# Postnatal epigenetic regulation of intestinal stem cells requires DNA methylation and is guided by the microbiome

**DOI:** 10.1186/s13059-015-0763-5

**Published:** 2015-09-30

**Authors:** Da-Hai Yu, Manasi Gadkari, Quan Zhou, Shiyan Yu, Nan Gao, Yongtao Guan, Deborah Schady, Tony N. Roshan, Miao-Hsueh Chen, Eleonora Laritsky, Zhongqi Ge, Hui Wang, Rui Chen, Caroline Westwater, Lynn Bry, Robert A. Waterland, Chelsea Moriarty, Cindy Hwang, Alton G. Swennes, Sean R. Moore, Lanlan Shen

**Affiliations:** Department of Pediatrics, Baylor College of Medicine, USDA/ARS Children’s Nutrition Research Center, 1100 Bates St., Ste. 8020, Houston, TX 77030 USA; Department of Biological Sciences, Rutgers University, Newark, NJ 07102 USA; Department of Molecular & Human Genetics, Baylor College of Medicine, Houston, TX 77030 USA; Department of Pathology, Texas Children’s Hospital, Baylor College of Medicine, Houston, TX 77030 USA; Department of Molecular and Human Genetics, Human Genome Center, Baylor College of Medicine, Houston, TX 77030 USA; Department of Oral Health Sciences, Medical University of South Carolina, Charleston, SC 29425 USA; Center for Clinical and Translational Metagenomics, Brigham & Women’s Hospital Harvard Digestive Diseases Center, Boston, MA 02115 USA; Center for Comparative Medicine and Department of Molecular Virology and Microbiology, Baylor College of Medicine, Houston, TX 77030 USA; Gastroenterology, Hepatology, & Nutrition, Center for Global Child Health, Cincinnati Children’s Hospital Medical Center, Cincinnati, OH 45229 USA

## Abstract

**Background:**

DNA methylation is an epigenetic mechanism central to development and maintenance of complex mammalian tissues, but our understanding of its role in intestinal development is limited.

**Results:**

We use whole genome bisulfite sequencing, and find that differentiation of mouse colonic intestinal stem cells to intestinal epithelium is not associated with major changes in DNA methylation. However, we detect extensive dynamic epigenetic changes in intestinal stem cells and their progeny during the suckling period, suggesting postnatal epigenetic development in this stem cell population. We find that postnatal DNA methylation increases at 3′ CpG islands (CGIs) correlate with transcriptional activation of glycosylation genes responsible for intestinal maturation. To directly test whether 3′ CGI methylation regulates transcription, we conditionally disrupted two major DNA methyltransferases, *Dnmt1* or *Dnmt3a*, in fetal and adult intestine. Deficiency of *Dnmt1* causes severe intestinal abnormalities in neonates and disrupts crypt homeostasis in adults, whereas *Dnmt3a* loss was compatible with intestinal development. These studies reveal that 3′ CGI methylation is functionally involved in the regulation of transcriptional activation in vivo, and that *Dnmt1* is a critical regulator of postnatal epigenetic changes in intestinal stem cells. Finally, we show that postnatal 3′ CGI methylation and associated gene activation in intestinal epithelial cells are significantly altered by germ-free conditions.

**Conclusions:**

Our results demonstrate that the suckling period is critical for epigenetic development of intestinal stem cells, with potential important implications for lifelong gut health, and that the gut microbiome guides and/or facilitates these postnatal epigenetic processes.

**Electronic supplementary material:**

The online version of this article (doi:10.1186/s13059-015-0763-5) contains supplementary material, which is available to authorized users.

## Background

The ontogeny of mammalian intestinal development encompasses three distinct phases: morphogenesis and cytodifferentiation during late gestation, the shift from intra- to extra-uterine environment at birth, and the transition from an exclusively milk diet rich in fat to a solid diet rich in carbohydrates at weaning. To meet the increased environmental and nutritional demands after birth, early postnatal life is a critical period during which the proliferative units of the intestinal epithelium known as crypts of Lieberkühn undergo extensive structural and functional maturation [1]. The intricate morphology, cellular composition and turnover rate of intestinal crypts are all controlled by multipotent intestinal stem cells (ISCs) located at the base of flask-shaped mucosal invaginations [2]. ISCs therefore constitute the ‘control center’ that regulates lifelong intestinal health and disease. Remarkably, however, while it has long been known that postnatal intestinal development is characterized by rapid growth and changes in brush border digestive functions [3], our understanding of postnatal development of ISCs is limited.

Recent technological developments enable the identification and isolation of live ISCs with high purity. Lgr5^+^ cells from mouse intestinal crypts were functionally validated as bona fide ISCs by lineage tracing studies [4]. With the sophistication of genetic studies that allow gene ablation in Lgr5^+^ ISCs, we are developing a broader appreciation of signaling pathways and transcriptional factors that control their early cell fate decisions [5]. Although the role of epigenetics in intestinal development has gained more attention recently [6–10], we still know little about the fundamental epigenetic mechanisms that control the origin, identity, and behavior of ISCs during development.

DNA methylation of cytosine in CpG dinucleotides is a well-established epigenetic mechanism critical for mammalian development. CpG density is extensively depleted in the mammalian genome; however, about 1 % of the genome escaped this CpG depletion, resulting in scattered regions of high CpG density termed CpG islands (CGIs). Interestingly, whereas most CpGs in the genome are methylated, significantly less methylation is observed at CGIs. CGI methylation appears to target specific regions such as promoters of X-linked genes on the inactive X chromosome in females, genomically imprinted loci, and genes associated with tissue-specific expression [11, 12]. Although DNA methylation is widely viewed as an epigenetic mark for gene silencing, we recently discovered that methylation at non-promoter CGIs, particularly at the 3′ end of genes, promotes human gene activation through a CTCF-dependent enhancer-blocking mechanism [13], underscoring the need for unbiased methods to study epigenetic regulation by DNA methylation during development.

The ontogenic periods, when developmentally programmed DNA methylation is being established, are vulnerable to environmental influences [14]. DNA methylation requires enzymes, DNA methyltransferases (DNMTs), and nutrition-dependent metabolic pathways that supply methyl groups [15–17]. It has become clear that postnatal establishment of gut microbiota plays a key role in several aspects of intestinal physiology, including morphological features [18, 19], altered glycosylation patterns [20–22], and stem cell activity [23–26]. Further, the intestinal microbiota has the capacity to produce folate and a variety of vitamins (i.e., B12 and B6) affecting host one-carbon metabolism [27, 28]. This is important because mammals are incapable of synthesizing folate and other B vitamins (which act as methyl donors and cofactors in biological methylation reactions) so they have to be obtained exogenously from diet and intestinal bacteria. Until now, little is known about the impact of gut microbiome on the host epigenome. In adult intestinal epithelial cells, methylation of the Toll-like receptor gene *TLR4* depends on intestinal commensal bacteria [29, 30], and DNA methylation in blood is associated with microbiota composition during pregnancy [31]. No previous studies, however, have examined how the early postnatal microbiome affects developmental epigenetics in host ISCs.

Here, employing genome-wide approaches, we show that ISCs undergo dynamic postnatal epigenetic changes during the suckling period. Using genetic approaches that permit conditional and inducible inactivation of Dnmts, we provide evidence that *Dnmt1* regulates postnatal epigenetic mechanisms, with a major impact on ISC function in both neonatal and adult intestine. Finally, we demonstrate that the postnatal gut microbiome is required to guide and/or enable these postnatal epigenetic processes.

## Results

### The DNA methylome changes more during postnatal ISC maturation than during differentiation of ISCs

We focused mainly on ISCs from the colon since this region of the gut is the primary site of several human diseases, including inflammatory bowel disease and cancer. Although Lgr5^+^ cells are known to exhibit stem cell properties before birth and drive the dynamics of developing crypts in the small intestine [32], their ontogeny has not been examined in the developing colon. We, therefore, characterized the temporal emergence of Lgr5^+^ colonic ISCs by fluorescence activated cell sorting (FACS) at embryonic and fetal stages using knock-in *Lgr5-EGFP-CreER* mice [4]. Lgr5-EGFP^+^ colonic ISCs are detectable at embryonic day 14.5 (E14.5) and become an appreciable subpopulation by E18.5 (Additional file 1: Figure S1).

**Fig. 1:**
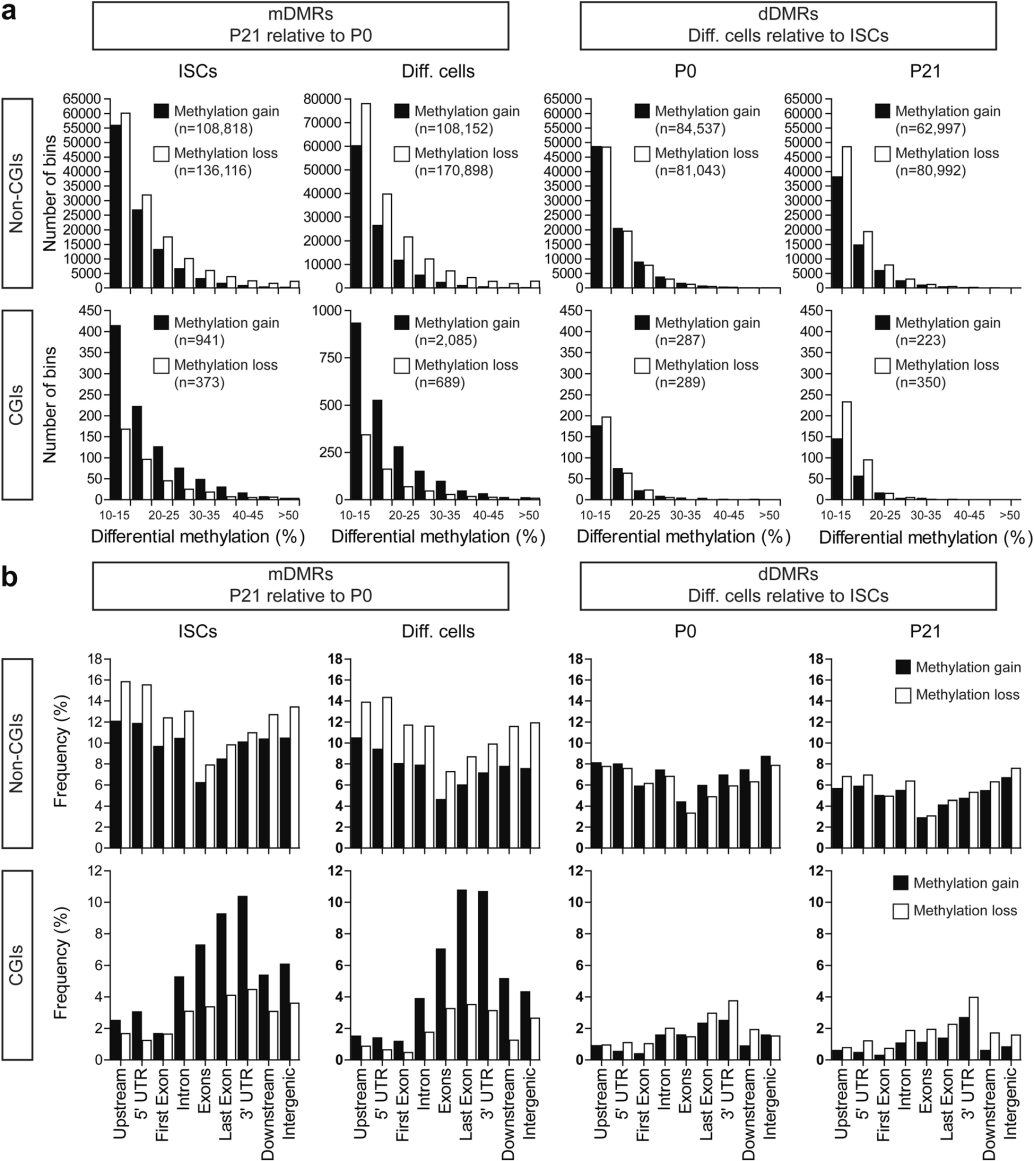
Unbiased analysis of DNA methylomes in colonic ISCs and their progeny during the suckling period. **a** Numbers of DMRs identified from the comparisons between either developmental stages (*mDMRs*, *left*) or differentiation states (*dDMRs*, *right*). Analyses were based on the association of DMRs with non-CGIs (*top*), and with CGIs (*bottom*). *Black bars* indicate methylation gains and *white bars* indicate methylation losses. **b** Distribution of mDMRs or dDMRs relative to gene region categories based on RefSeq annotation of all known genes. CGI-associated mDMRs are enriched in the gene body and 3′ end

To determine whether the ISC population undergoes epigenetic changes during early postnatal life, we performed unbiased DNA methylation mapping by whole-genome bisulfite sequencing (WGBS) [33] in Lgr5-EGFP^+^ colonic ISCs at birth [postnatal day 0 (P0)] and at the end of the suckling period (P21). Since colonic ISCs are continually undergoing differentiation to replace the short-lived population of differentiated epithelial cells, we analyzed the DNA methylomes of sorted epithelial cell adhesion molecule (EpCAM)-positive and Lgr5-enhanced green fluorescent protein (EGFP)-negative population (the descendants of ISCs) [34–36] to identify DNA methylation changes that correlate with differentiation (differentiated cells versus ISCs). We found that at both ages, global methylation levels were significantly lower in differentiated cells relative to ISCs (*P* < 0.0001; Additional file 1: Figure S2). Across the 25 million CpG sites analyzed, average CpG methylation levels were 73.9 % and 70.3 % at P0, and 73.1 % and 70.0 % at P21, for ISCs and differentiated cells, respectively. We compared specific genomic regions that underwent methylation changes either during maturation from P0 to P21 [maturation-associated differentially methylated regions (mDMRs)] or during differentiation from ISCs to epithelial cells [differentiation-associated differentially methylated regions (dDMRs)]. Our analysis revealed that there are more mDMRs than dDMRs, particularly at CGI-associated genomic regions (Fig. 1a). Interestingly, at non-CGIs, mDMRs frequently lose methylation, but at CGIs, mDMRs predominantly gain methylation (Fig. 1a). There was no significant enrichments of a gene region for non-CGI associated DMRs as compared to known genes, regardless of mDMR or dDMR, methylation gains or losses (Fig. 1b).  The pattern was completely different, however, at CGI-associated mDMRs; methylation gains at CGI mDMRs were strongly associated with the gene body (introns and exons, excluding the first and last exons) and 3′ end of genes (last exon and 3′ UTR) (Fig. 1b). Together, these results demonstrate that changes in genomic DNA methylation patterns are more dynamic during postnatal ISC maturation than during differentiation of ISCs to epithelial cells.

### 3′ CGI-associated mDMRs are functionally implicated in development and intestinal maturation

To evaluate the functional significance of mDMRs, we performed DAVID Gene Ontology (GO) analyses to identify statistically overrepresented biological processes (see Additional file 2: Tables S1 and S2 for lists of CGI and non-CGI mDMR genes). This analysis revealed multiple developmental processes significantly associated with mDMR genes showing methylation gains at non-promoter CGIs (Additional file 2: Table S3 contains a list of all significantly enriched GO terms). For example, genes with mDMRs in the gene body or 3′ CGI are enriched for embryonic organ development, intracellular signaling cascade, regulation of transcription, and cell morphogenesis involved in differentiation (Additional file 1: Figure S3). Of particular interest is the glycosphingolipid biosynthetic process, which is intimately linked to intestinal maturation by modulating mucus barrier function; glycosphingolipids may additionally modulate receptors for toxins, virus, and bacteria [37, 38]. Indeed, a careful examination of the gene list revealed a cluster of 3′ CGI genes responsible for glycan biosynthesis, including glycosidase (*Net37*), glycosyltransferases (*A3galt2*, *B4galnt1*, *B4galnt4*, and *Gal3st1*) and other related enzymes (*Fkrp* and *Phospho1*). When we compared the gene lists for 3′ CGI-associated mDMRs in ISCs and their differentiated progeny during the suckling period, we found, as expected, that most (60 %) of the mDMR genes in differentiated cells are found in ISCs (Additional file 1: Figure S4a). Since differentiated cells are more exposed to the intestinal lumen than are the ISCs in the crypts, mDMRs unique to differentiated cells may reflect responses to the very different luminal environment at P21 relative to P0. Not surprisingly, only 7 % (41/517) of mDMR genes in the differentiated cells overlap with P21 dDMR genes.

### Methylation gains at 3′ CGIs of glycosylation genes correlate tightly with transcriptional activation in the developing ISCs

To gain insight into the function of the methylation changes at mDMRs, we used RNA-Seq to comprehensively examine the relationship between ISC mDMRs and gene expression. We found that 3′ CGI methylation was positively correlated with gene expression (Additional file 1: Figure S4b), suggesting that postnatal establishment of DNA methylation in ISCs plays a functional role in intestinal maturation. To validate our genome-wide results, we focused on ten candidate genes, including five with mDMRs at non-CGIs and five with mDMRs at CGIs. All the non-CGI mDMR genes (*Rnf43*, *Zbtb22*, *Fam109a*, *Adamtsl5*, and *Mif*) showed DNA methylation loss in the ISCs during the suckling period. They were selected because their mDMRs are proximal to CGIs, in regions known as CGI shores, which have been shown to exhibit tissue-specific methylation correlated with gene expression [39]. Notably, *Rnf43* is a stem cell E3 ligase which acts as a negative regulator of Wnt signaling [40]. All the CGI mDMR genes (*B4galnt1*, *Net37*, *Lpar5*, *Fkrp*, and *Phospho1*) showed gain of methylation during ISC development. Among them, four (*B4galnt1*, *Net37*, *Fkrp*, and *Phospho1*) encode enzymes that affect glycosylation, and one (*Lpar5*) is involved in sodium and water absorption in the intestine [41].

**Fig. 2:**
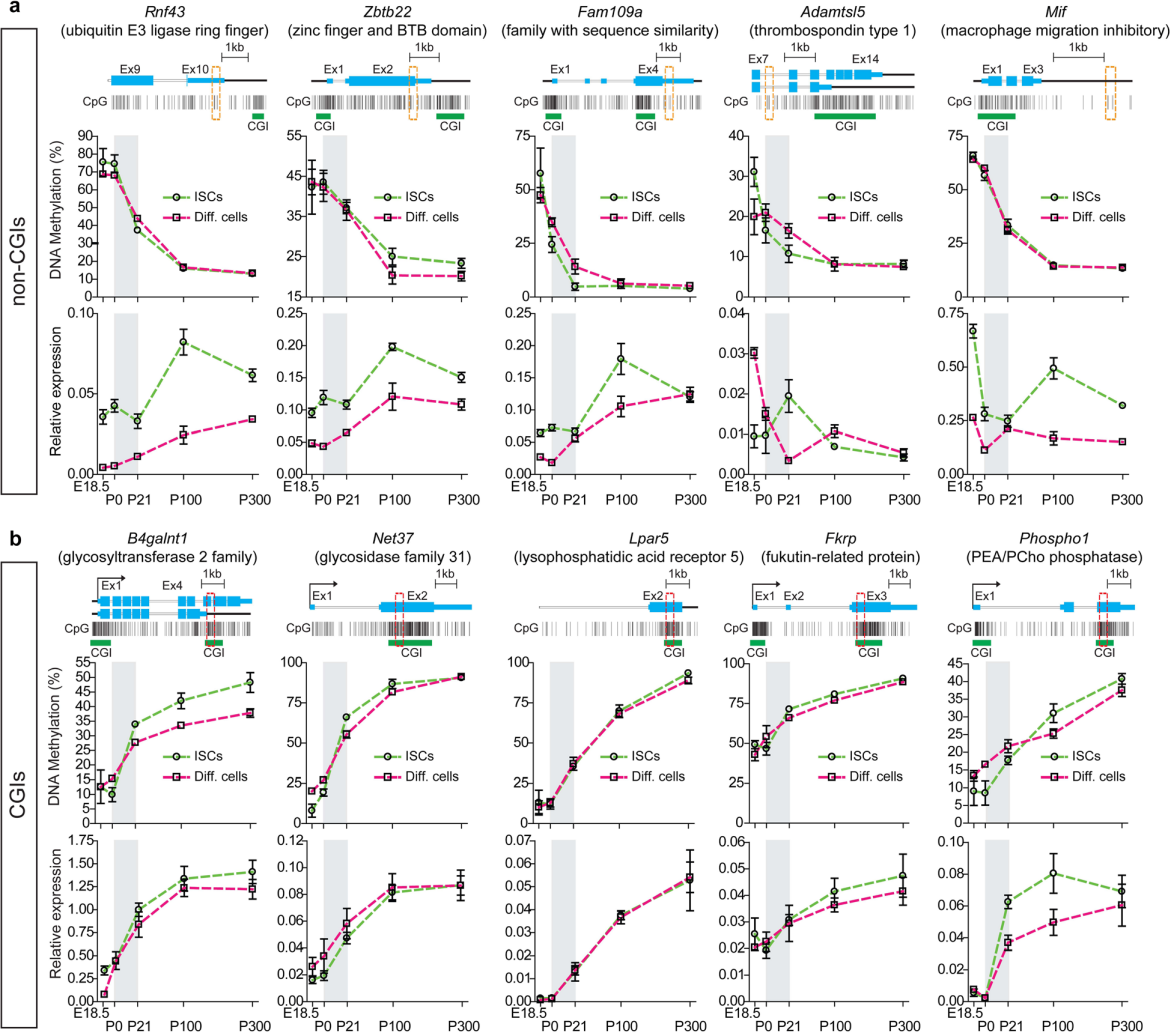
Temporal analysis of both DNA methylation and gene expression at identified candidate genes. **a** Non-CGI mDMR genes undergoing developmental loss of methylation. **b** 3′ CGI mDMR genes undergoing developmental gain of methylation. The suckling period is highlighted in *gray*. For each gene, exon–intron structure (including isoforms) and CpG map are shown on the *top. Thin blue bars* represent the 5′ or 3′ UTRs and *thick blue boxes* represent coding exons. Each vertical line represents a CpG site and *green bars* indicate CGIs. *Red dotted boxes* indicate the regions analyzed for CpG methylation. The gene expression is relative to *β-actin. Error bars* represent standard error of the mean of three to five biological replicates

To assess the correlation between DNA methylation and gene expression over an extended time-series, we analyzed DNA methylation and gene expression quantitatively in both ISCs and differentiated epithelial cells at E18.5, P0, P21, P100 (young adult), and P300 (old adult). In all cases, the results (Fig. 2) not only confirmed our WGBS findings but also demonstrated that ISC epigenetic regulation extends beyond the suckling period. Interestingly, we found no significant correlation between methylation changes at non-CGI genes and gene expression during the suckling period, but over the extended time course, expression increases lagged behind methylation decreases (Fig. 2a; Additional file 2: Table S4). Conversely, at all five of the 3′ CGI genes examined we found a positive temporal correlation between 3′ CGI methylation and transcriptional activation across development (Fig. 2b; Additional file 2: Table S4). To study at higher resolution the developmental dynamics of DNA methylation in these regions, we performed methylation analysis of 3′ CGI genes at P4, P7, P11, P16, and P18. The 3′ CGI methylation increases for several glycosylation genes (*B4galnt1*, *Net37*, and *Fkrp*) were most dramatic during the first week of postnatal life (Additional file 1: Figure S5), suggesting that some developmental signal coincident with parturition drives these changes. Most importantly, the nearly identical DNA methylation dynamics in ISCs and differentiated epithelial cells, from fetal to adult life (Fig. 2a, b), further support the conclusion from our genome-wide analyses that differentiation of ISCs does not entail widespread changes in DNA methylation.

### DNMT1-mediated DNA methylation is essential for postnatal intestinal development

**Fig. 3:**
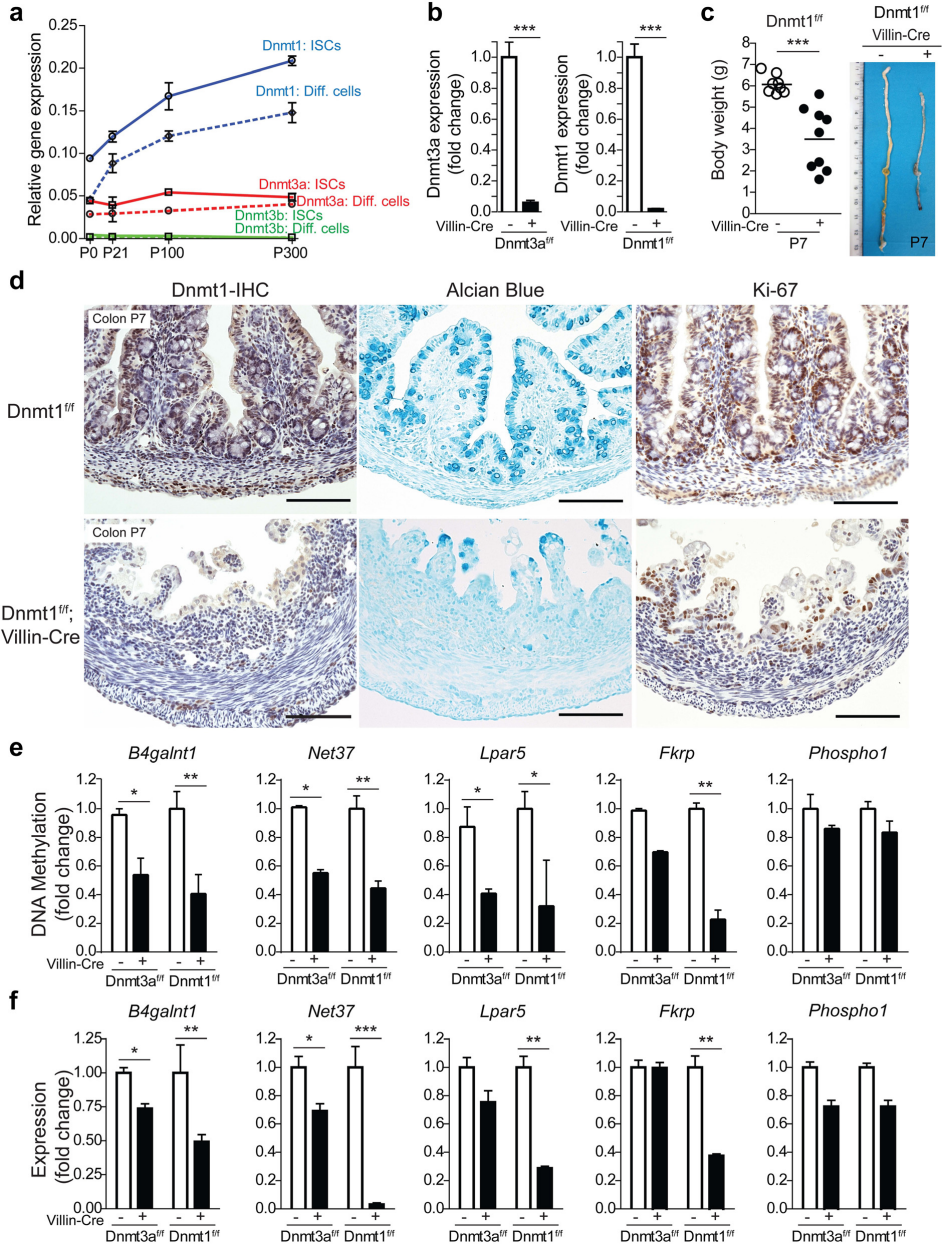
Deletion of *Dnmt3a* or *Dnmt1* in developing intestine. **a** mRNA expression analysis of three *Dnmts* in Lgr5^+^ ISCs and differentiated epithelial cells at various postnatal ages. **b** mRNA expression analysis confirms efficient epithelial cell specific ablation of *Dnmt3a* (*left*, by 90 %) or *Dnmt1* (*right*, by 95 %) in homozygous mutant mice. **c** Villin-Cre mediated *Dnmt1* deletion caused significant reductions in body weight (*left*) and intestinal length (*right*) at P7. **d** Immuno-histochemical staining for Dnmt1, Alcian blue and Ki-67 showed blunted villi, reduced goblet cells, and decreased proliferative cells in the colons of *Dnmt1* ^*f/f*^ *; Villin-Cre* mice. Scale bars, 100 μm. DNA methylation (**e**) and gene expression (**f**) analysis of 3′ CGI-associated genes in the colonic intestinal epithelium collected from *Dnmt3a* and *Dnmt1* homozygous mutant mice at P7, compared with control littermates. *Error bars* represent standard error of the mean of at least three replicate experiments. *P < 0.05, **P < 0.01, and ***P < 0.001 versus control (two-sided *t* test)

To further address the functional role of DNA methylation in intestinal development, we performed genetic studies by targeting genes responsible for the establishment and maintenance of DNA methylation. *Dnmt3a* and *Dnmt3b* are known as de novo methyltransferases (to establish methylation patterns during development) and *Dnmt1* is considered a maintenance methyltransferase (to propagate established methylation patterns in daughter cells during mitosis) [42]. By examining the mRNA expression patterns of these three *Dnmts* from P0 to P300, we found that *Dnmt3b* expression is essentially undetectable in either ISCs or differentiated epithelial cells (Fig. 3a, green lines). *Dnmt3a* expression is intermediate and relatively constant (Fig. 3a, red lines), whereas *Dnmt1* expression is the strongest in both ISCs and differentiated epithelial cells and increases progressively with age (Fig. 3a, blue lines). Based on these results, we focused our functional analyses on *Dnmt3a* and *Dnmt1*. We derived *Dnmt3a*^*f/f*^*; Villin-Cre* and *Dnmt1*^*f/f*^*; Villin-Cre* mouse lines to ablate individual *Dnmt* genes in an intestinal epithelial cell-specific fashion. In both *Dnmt3a* and *Dnmt1* homozygous mutant mice, we detected a nearly 90 % reduction in *Dnmt3a* and *Dnmt1* expression in neonatal intestines, respectively (Fig. 3b; Additional file 1: Figure S6). At P0, *Dnmt3a* and *Dnmt1* homozygous mutant pups were indistinguishable from their control littermates in terms of body weight and gross appearance. By P7, however, *Dnmt1* homozygous mutant mice showed significantly reduced body weight and shortened intestinal length compared with their littermate controls (Fig. 3c). Most (~80 %) of *Dnmt1* homozygous mutant mice died around the time of weaning (P21). Survivors had crypts containing cells that had escaped *Dnmt1* deletion. In contrast to the *Dnmt1* mutants, no obvious changes were detected in *Dnmt3a* homozygous mutant mice (Additional file 1: Figure S7). To better characterize the postnatal lethal phenotype of *Dnmt1* mutants, we performed histological and immunohistochemical analyses. These demonstrated that at P7, loss of Dnmt1 results in morphological changes characterized by villus-atrophy and vacuolated cells in both small intestine (Additional file 1: Figure S8) and colon (Fig. 3d). In addition, the *Dnmt1* mutant colons showed fewer goblet cells (indicated by Alcian blue staining), and a decrease in Ki-67^+^ proliferative cells (Fig. 3d). These alterations were not apparent at P0 or P3 (Additional file 1: Figure S9), indicating that the observed epithelial deterioration occurs postnatally. Notably, signs of severe mucosal damage, including epithelial pseudostratification and adenoma-like lesions, were occasionally observed in P7–P21 *Dnmt1* homozygous mutant mice (scored by pathologist; Additional file 1: Figure S8b). These histopathological changes potentially indicate indirect consequences of epithelial injury or inflammation.

To investigate the direct impact of *Dnmt* loss on postnatal epigenetic regulation of gene expression, we focused on the five previously characterized 3′ CGI mDMRs, and performed quantitative analyses of DNA methylation and gene expression in colonic epithelial cells at P7. *Dnmt3a* deletion caused significant methylation decreases for *B4galnt1*, *Net37*, and *Lpar5* (Fig. 3e; Additional file 1: Figure S10), but these were only modestly associated with expression (Fig. 3f). *Dnmt1* deletion, however, resulted in greater methylation decreases in four of the five genes (Fig. 3e; Additional file 1: Figure S10), causing stronger changes in expression (Fig. 3e). In all cases, loss of methylation was correlated with reduced expression, consistent with the notion that 3′ CGI methylation acts as a transcriptional activator. For *Net37*, the degree of expressional reduction far exceeded the alteration of methylation, reflecting broader impact of *Dnmt1* loss on additional regulatory regions, consistent with previous observations [7, 9]. Together, these results suggest a regulatory function of 3′ CGI methylation in postnatal mouse intestinal development, providing the first in vivo evidence to support the involvement of *Dnmt1* in controlling postnatal epigenetic regulation and epithelial maturation.

### *Dnmt1*-mediated DNA methylation is required for the control of adult ISC homeostasis

**Fig. 4:**
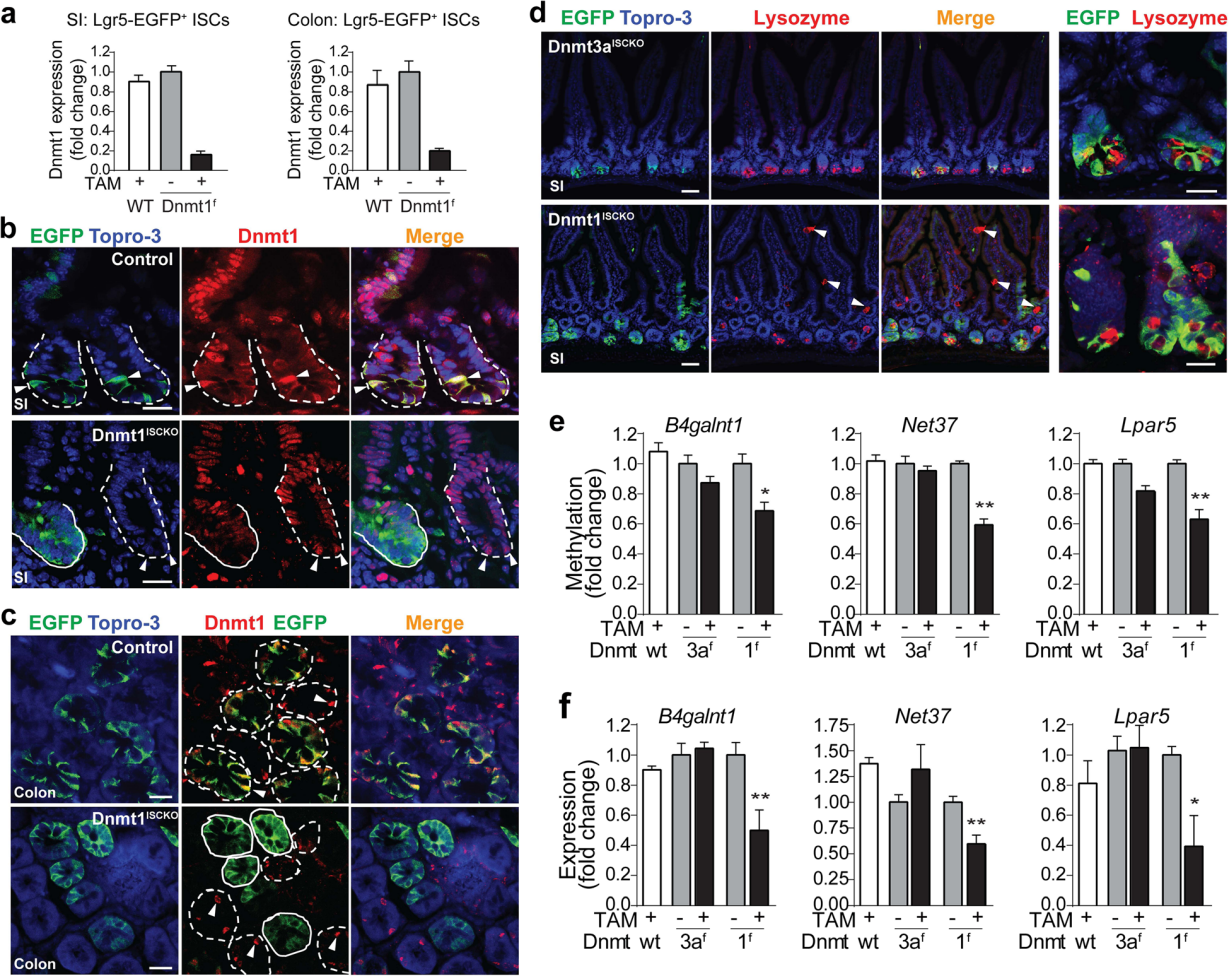
Deletion of *Dnmt1* in adult ISCs alters crypt homeostasis and postnatal epigenetic regulation. **a** mRNA expression analysis confirms decreased expression of *Dnmt1* in Lgr5-EGFP^+^ ISCs isolated from *Dnmt1* ^*ISCKO*^ mice, in both small intestine (*SI*) and colon. *TAM* tamoxifen, *WT* wild type. **b** Immunofluorescent analysis of ISCs (*EGFP*, *green*), Dnmt1 (*red*), and nuclei (*Topro-3*, *blue*) in small intestine crypts. In tamoxifen-treated control (*Dnmt-wild type; Lgr5-EGFP-CreER*) mice (*top panel*), *dotted lines* mark crypts, and ISCs (*green*) show strong Dnmt1 staining (indicated by *white arrowheads*). In *Dnmt1* ^*ISCKO*^ mice (*bottom panel*), the Lgr5-EGFP^+^ mutant crypt (outlined by *solid line*) shows decreased Dnmt1 staining and aggregation of CBCs, whereas an adjacent EGFP-negative crypt (outlined by *dashed line*) contains CBCs with wild-type *Dnmt1* and high expression of Dnmt1 (*white arrowheads*). Scale bars, 20 μm. **c** Immunofluorescent analysis of ISCs and Dnmt1 in control (*top panel*) and *Dnmt1* ^*ISCKO*^ (*bottom panel*) colons. Similar mosaic patterns of Dnmt1 deletion are observed in colon. Scale bars, 20 μm. **d** Immunofluorescent analysis of Paneth cells in *Dnmt3a* ^*ISCKO*^ and *Dnmt1* ^*ISCKO*^ mice. Paneth cells staining positive for lysozyme (*red*) are exclusively located at crypt bottoms in *Dnmt3a* ^*ISCKO*^ mice (*top panel*), whereas lysozyme^+^ cells (*arrowheads*) were detected at villus epithelia in *Dnmt1* ^*ISCKO*^ mice (*bottom panel*). Scale bars, 50 μm. *Far right panels* show a magnified view of Lgr5-EGFP^+^ ISCs for each genotype; the clear segregation between CBCs and Paneth cells is lost in the *Dnmt1* ^*ISCKO*^ mice. DNA methylation (**e**) and gene expression (**f**) analysis of 3′ CGI-associated genes in the colonic Lgr5^+^ ISCs collected from wild-type (*wt*) control, *Dnmt3a* ^*f/f*^; *Lgr5-EGFP-CreER* and *Dnmt1* ^*f/f*^; *Lgr5-EGFP-CreER* mice, with or without tamoxifen (*TAM*) administration. Error bars represent standard error of the mean of at least three replicate experiments. *P < 0.05, **P < 0.01, and ***P < 0.001 versus control (two-sided *t* test)

The data above on pan-intestinal epithelial cell deletion (by Villin-Cre) raised an intriguing possibility that *Dnmt1*-mediated postnatal epigenetic mechanisms could be involved in the regulation of adult ISC function. To test this, we derived a *Dnmt1*^*f/f*^*; Lgr5-EGFP-CreER* mouse line, in which deletion of *Dnmt1* can be induced in adult ISCs by tamoxifen (referred to hereafter as *Dnmt1*^*ISCKO*^). We used two types of controls: the *Dnmt-wild type; Lgr5-EGFP-CreER* mice with the same tamoxifen treatment, and the *Dnmt1*^*f/f*^*; Lgr5-EGFP-CreER* mice without tamoxifen treatment. Based on real-time RT-PCR analysis of *Dnmt1* expression in Lgr5-EGFP^+^ cells, we estimated that tamoxifen treatment induces around 80 % ablation of *Dnmt1* in both small intestine and colon (Fig. 4a). Using confocal immunofluorescent analyses for Dnmt1 and EGFP, we analyzed Dnmt1 protein expression in Lgr5^+^ crypt-based columnar (CBC) cells. Tamoxifen-injected wild-type control mice strongly expressed Dnmt1 in the Lgr5-EGFP^+^ CBCs (white arrowheads, top panel in Fig. 4b). Expression of Dnmt1 was also detected in villus epithelial cells, consistent with our previous real-time RT-PCR results (Fig. 3a). In small intestines of *Dnmt1*^*ISCKO*^ mice, co-staining of EGFP and Dnmt1 showed decreased Dnmt1 expression in Lgr5-EGFP^+^ ISCs (solid line marks the mutant crypt, Fig. 4b). It is known that Cre recombinase is expressed mosaically in the intestinal epithelium of *Lgr5-EGFP-CreER* mice [4]. Indeed, in the same *Dnmt1*^*ISCKO*^ mice, some Lgr5-EGFP-negative crypts, which are Dnmt1 wild type as a result of no recombination, retained strong Dnmt1 staining (dotted line circles the wild-type crypt with white arrowheads pointing to the Dnmt1^+^ cells, Fig. 4b, bottom panel). Similar mosaic deletion patterns were observed in colons of *Dnmt1*^*ISCKO*^ mice (solid circles indicate mutant crypts, and dashed circles indicate wild-type crypts with arrowheads pointing to Dnmt1^+^ cells, Fig. 4c). Therefore, our RT-PCR and immunostaining results for Lgr5+ cells suggest efficient but incomplete ablation of Dnmt1 from the adult ISCs. Notably, compared with the well-defined triangle shapes of wild-type CBCs (arrowheads in Fig. 4b), mutant CBCs in *Dnmt1*^*ISCKO*^ mouse crypts showed altered morphologies with most cells aggregating into clumps (solid circles, Fig. 4b; Additional file 1: Figure S11), suggesting dysregulated crypt homeostasis. Our observations on Paneth cells also supported this notion. In small intestines of *Dnmt1*^*ISCKO*^ mice, Lysozyme^+^ Paneth cells lost their clear compartmentalization with ISCs, and some were mislocalized in the villus epithelium (arrowheads, bottom panel in Fig. 4d). Normally, Paneth cells are localized in the crypt (top panel in Fig. 4d).

In parallel, we derived a *Dnmt3a*^*f/f*^; *Lgr5-EGFP-CreER* mouse line and applied the same strategy to evaluate the functional consequences of induced *Dnmt3a* deletion in adult ISCs (*Dnmt3a*^*ISCKO*^). After tamoxifen administration, we confirmed the high-efficiency of gene ablation in Lgr5-EGFP^+^ ISCs from *Dnmt3a*^*ISCKO*^ animals (Additional file 1: Figure S12a). In contrast to *Dnmt1*^*ISCKO*^, *Dnmt3a*^*ISCKO*^ small intestine and colon exhibited no obvious morphological abnormalities (Additional file 1: Figure S12b). Lysozyme staining revealed normal Paneth cell localization (top panel in Fig. 4d). Overall, these results were consistent with our observation in the *Dnmt3a*^*f/f*^; *Villin-cre* mice.

Finally, to determine whether dysregulation of postnatal epigenetic mechanisms might be involved in the observed phenotypic defects of *Dnmt1*-deficient ISCs, we analyzed DNA methylation and gene expression of three genes (*B4galnt1*, *Net37*, and *Lpar5*) that normally undergo increases in DNA methylation at 3′ CGIs. All three exhibited significantly reduced DNA methylation in the ISCs after disruption of *Dnmt1* but not *Dnmt3a* (Fig. 4e; Figure S13). As in the Villin-Cre experiments (Fig. 3e, f), hypomethylation at the 3′ CGIs led to reduced mRNA expression (Fig. 4f). These results provide further evidence that *Dnmt1* is a prominent player in control of the postnatally programmed DNA methylation in ISCs, while *Dnmt3a* is somewhat dispensable. Collectively, these data indicate that *Dnmt1*-mediated developmentally programmed epigenetic mechanisms are involved in the regulation of adult ISC function.

### Microbial exposure guides developmental epigenetics in the colonic epithelium

**Fig. 5:**
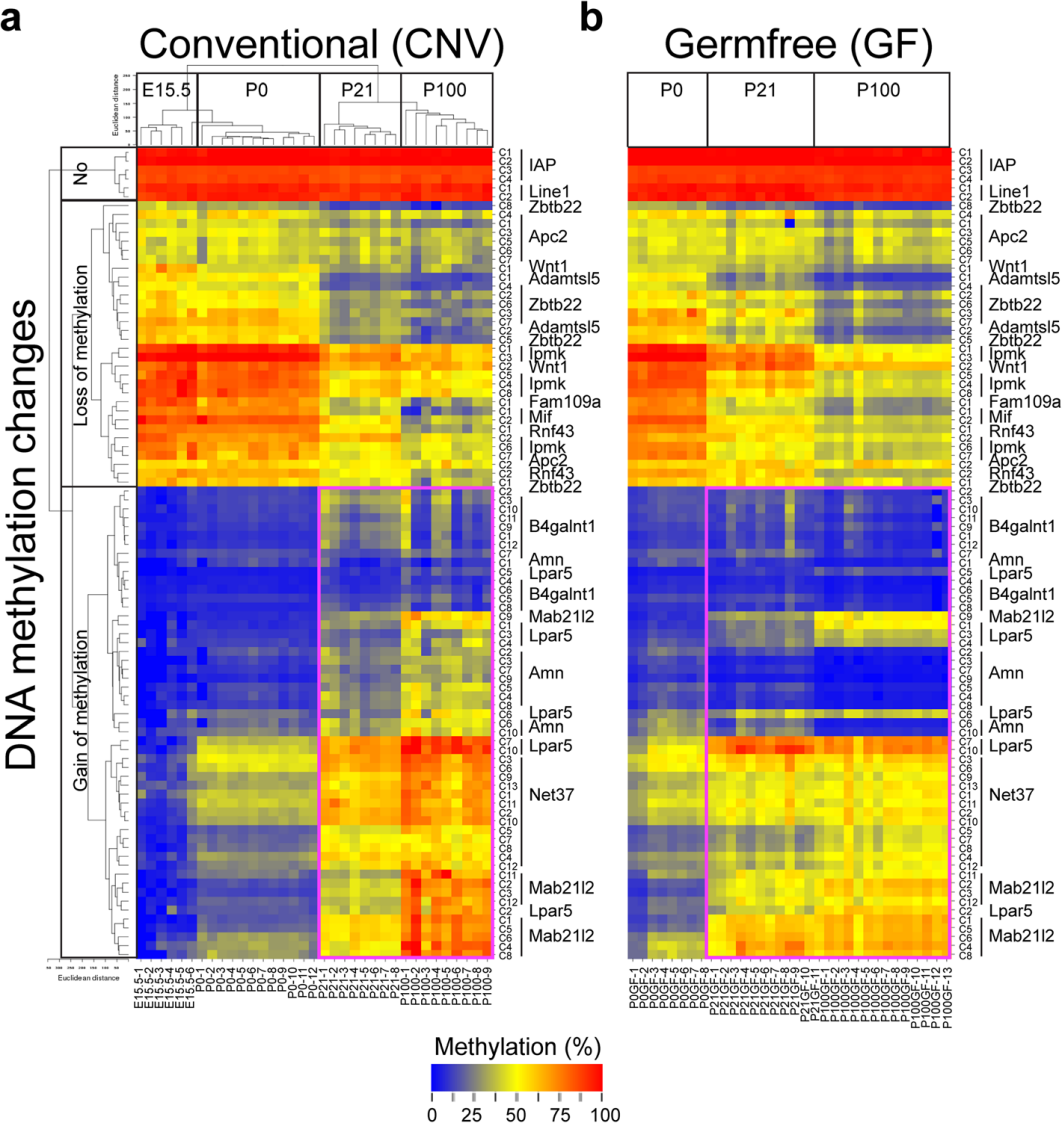
The germ-free condition affects developmentally programmed CGI methylation. **a** Unsupervised hierarchical clustering analysis on the basis of DNA methylation profiling in mouse colon under conventional (*CNV*) conditions. Each *row* indicates a CpG site and the corresponding gene name is indicated on the *right*. Each *column* indicates a sample analyzed and the corresponding age is indicated on the *bottom*. Methylation levels range from unmethylated (*blue*) to fully methylated (*red*), as indicated by the color legend at the bottom of the graphs. **b** DNA methylation profiling in mouse colon under germ-free (*GF*) conditions. The orders for CpG sites and age groups are pre-defined based on the conventional samples. *Purple boxes* highlight 3′ CGI-associated CpGs at which the methylation levels are significantly altered by germ-free conditions

Our discovery of postnatal epigenetic regulation in the ISC population highlights a potential critical ontogenic window in which the infant microbiome could influence intestinal development. To test this, we profiled DNA methylation in genetically identical C57 wild-type mice under either conventional (CNV) or germ-free (GF) conditions. We collected the colons at E15.5, P0, P21, and P100, and used the unfractionated tissues for methylation analysis. We quantitatively measured DNA methylation of over 90 CpG sites spanning 15 genes identified by WGBS as showing postnatal methylation changes in ISCs. In addition, to rule out the possibility that microbiome effects on DNA methylation were simply due to the changes of global methylation, we analyzed the methylation of two generic repetitive elements (*Line1* and *IAP*). Remarkably, in CNV mice, an unsupervised hierarchical clustering based purely on DNA methylation yielded a perfect correspondence with stage of development (Fig. 5a). In GF mice, however, these developmental changes in DNA methylation were markedly dysregulated, at both P21 and P100 (Fig. 5b). The most pronounced effect was in a large block of regions normally targeted for methylation increases from P0 to P21; in GF mice many of these changes did not occur, even by P100 (Fig. 5b, indicated by purple boxes). Additionally, we found similar methylation profiles of newborn mice (P0) in both CNV and GF conditions, consistent with these epigenetic changes being induced by colonization. It is worth mentioning that the macronutrient composition of the GF mouse diets was similar to that of the control CNV mouse diet (Additional file 2: Table S5). Further, we observed essentially the same results for GF mice housed at different facilities. Interestingly, we found significantly increased mRNA levels of *Dnmt1* in the GF mice at P21 and P100 (Additional file 1: Figure S14), indicating that the methylation defects under the GF condition are not due to insufficient Dnmt1 activity.

**Fig. 6:**
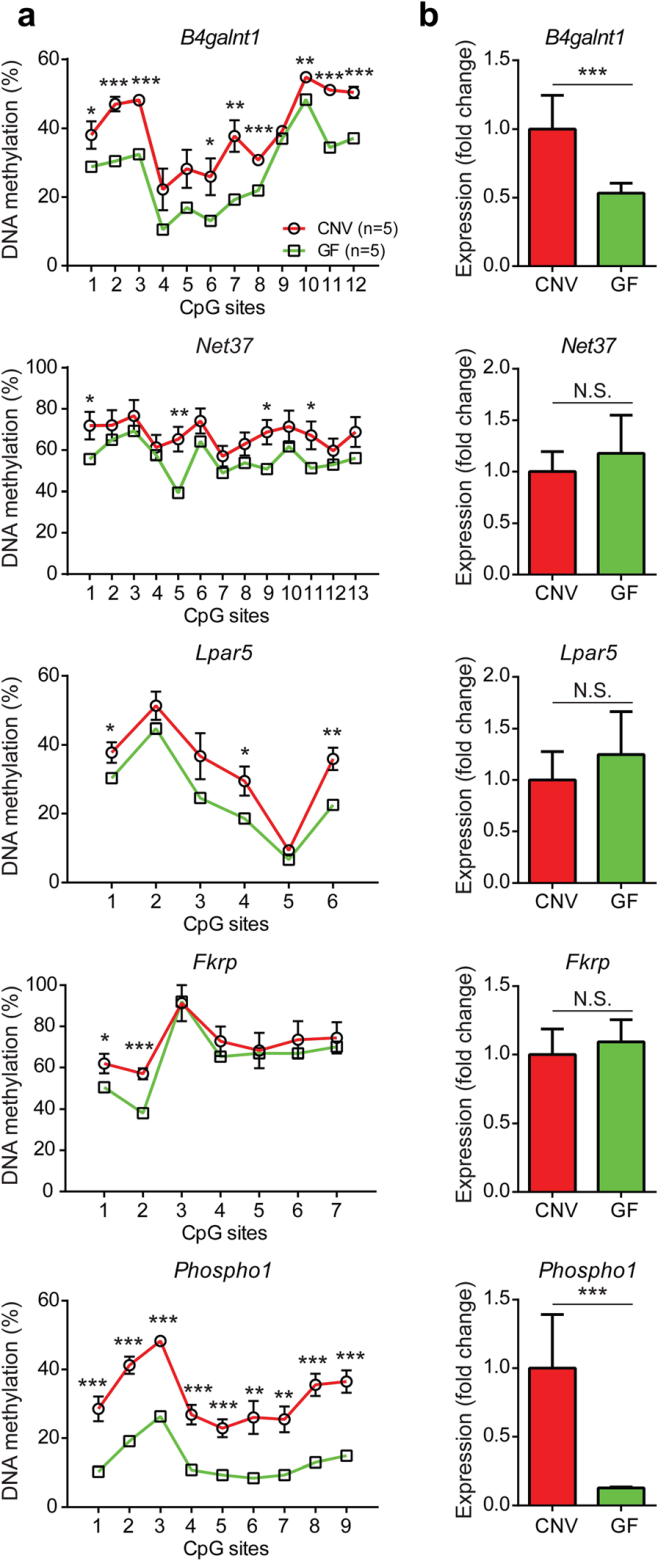
The germ-free condition affects developmentally programmed 3′ CGI methylation and transcription in intestinal epithelial cells. **a** DNA methylation status at multiple CpG sites of five 3′ CGI-associated genes are compared between two conditions: CNV (N = 5, *red*) and GF (N = 5, *green*). The* error bars* represent standard error of the mean. The *error bars* for the GF group are not showing as they are smaller than the symbols. **b** Gene expression analysis of corresponding 3′ CGI genes. *P < 0.05, **P < 0.01 and ***P < 0.001, *N.S.* not significant

To test whether the effects of the microbiome on DNA methylation might be mediated by altered cellular composition, and also to evaluate effects on gene expression, we obtained another cohort of mice at weaning under either CNV (N = 5) or GF (N = 5) conditions. Rather than studying whole colon, we isolated intestinal epithelial cells and assessed DNA methylation and expression of five 3′ CGI-associated genes. In agreement with our previous results, the GF condition significantly reduced the developmentally programmed methylation at all genes analyzed (Fig. 6a). We observed significantly reduced gene expression for the two genes with the greatest methylation decrements in GF mice (*B4galnt1* and *Phospho1*; Fig. 6b). Thus, the alterations in developmental epigenetics we observed in the whole colon of GF mice (Fig. 5b) occur within colonic epithelial cells.

Finally, to address the direct link between postnatal DNA methylation and the gut microbiome, we performed fecal microbiota transplant (FMT) experiments to conventionalize the GF mice at age P25. Based on our previous results, we examined 3′ CGI methylation of *B4galnt1* and *Phospho1* in colonic epithelial cells at P100 under FMT (N = 3) compared with GF (N = 3) and CNV (N = 2) conditions. As shown in Additional file 1: Figure S15, reestablishing commensal microbiota significantly increases DNA methylation at multiple CpG sites of 3′ CGIs. Consistently, restored DNA methylation at the 3′ CGI of the *Phospho1* gene in FMT mice leads to significantly increased gene expression.

## Discussion

Compared with the rapid recent progress in epigenome studies of embryonic and other somatic stem cells, epigenetic mechanisms in ISCs remain understudied. Recently, two genome-wide DNA methylation studies examined the role of DNA methylation in adult ISCs during differentiation, but yielded inconsistent results [7, 9]. Whereas one study showed limited DNA methylation dynamics during ISC differentiation [7], the second identified substantially more DMR regions, albeit with low levels of change [9]. Here, to address whether DNA methylation plays a regulatory role in ISC function, we chose to take a different approach, using an unbiased method to compare DNA methylomes in purified ISCs and their descendants at two distinct developmental stages (birth and weaning). Most notably, we found only minor methylation changes during differentiation of ISCs to epithelial cells, and that specific fractions of the genome undergo dynamic DNA methylation changes — apparent postnatal epigenetic regulation — within the ISC populations during the suckling period (i.e., from P0 to P21). Interestingly, a detailed characterization of these changes revealed continuous changes throughout intestinal maturation and into adulthood, supporting our earlier observations that postnatal epigenetic regulation proceeds well beyond infancy [43]. Thus, our results provide a framework for testing the role of DNA methylation ontogeny in the origin, identity, and behavior of ISCs across the developmental continuum.

Our results showed tight positive correlation of 3′ CGI methylation and gene expression throughout development. This (Fig. 2b) corroborates our previous reports that 3′ CGI methylation acts as a transcriptional activator during human embryonic stem cell differentiation [13], and is positively correlated with expression during murine hypothalamic development [44]. This is also consistent with our studies of conditional inactivation of Dnmt1, in which erasure of 3′ CGI methylation in ISCs resulted in decreased gene expression in vivo (Figs. 3 and 4). Mechanistically, it has been proposed that 3′ CGI methylation controls gene activation through a CTCF-dependent enhancer-blocking mechanism [13]. Although beyond the scope of this study, future studies will be necessary to reveal the exact mechanistic roles of epigenetic regulators, including CTCF, in the developmentally programmed transcriptional regulation of ISCs.

Conditional deletion of *Dnmts* in the intestine allowed us to dissect intrinsic mechanisms required for setting and maintaining developmentally programmed 3′ CGI methylation. Single knockout of *Dnmt3a* in intestinal epithelial cells caused significant decreases of 3′ CGI methylation, suggesting that *Dnmt3a* is involved in de novo methylation during early intestinal development. These reductions, however, were relatively modest and resulted in only slight transcriptional changes in associated genes, indicating that other de novo *Dnmt* genes might compensate for the loss of *Dnmt3a*. On the other hand, deletion of *Dnmt1* strongly decreased 3′ CGI methylation levels and led to concomitant decreases in transcription. Somewhat unexpectedly, when we induced *Dnmt3a* deletion by Lgr5-Cre in adolescent ISCs (when methylation gains at 3′ CGIs were still underway) we found no effects on de novo methylation. In contrast, after adolescent depletion of *Dnmt1*, we detected significantly reduced DNA methylation levels and mRNA expression in the ISCs. Taken together, our results indicate that *Dnmt1*, in addition to its dogmatic role as a maintenance methylase, also exhibits de novo methylation activity, as suggested by previous studies [45, 46].

Our studies demonstrated that the phenotypic consequences of intestine-specific *Dnmt1* deletion depend on the developmental stage at which the perturbation occurs. Compared with the effects of *Dnmt1* loss later in life, *Dnmt1* deletion during early postnatal life produced more striking morphological and functional changes, including severe postnatal growth retardation and development of adenomatous lesions in the intestine. Consistent with our findings, the recent study by Sheaffer et al. [9] showed that conditional disruption of *Dnmt1* by Villin-Cre in the adult intestine caused only modest changes in intestinal crypt homeostasis. To understand the contribution of DNA methylation to the observed defects in *Dnmt1* mutants, the previous study focused on identifying differential methylation during the transition from ISCs to differentiated epithelial cells [9]. Although our genome-wide analysis identified methylation changes in the differentiation axis similar to the previous report, we found substantially more profound methylation changes in the ISCs themselves during maturation from P0 to P21. Interestingly, a genome-wide study of other epigenetic marks also observed nearly identical histone marks (H3K4me2 and H3K27ac) and DNase I hypersensitivity in the adult ISCs and their progeny [8]. In light of these results, we suggest that the control of adult ISC function throughout life may be influenced by derangement of these postnatal maturational epigenetic processes. It remains to be determined, however, to what extent developmentally programmed epigenetic mechanisms contribute to the phenotypic defects seen in the *Dnmt1* mutant mice. Future studies with genome-wide analysis of *Dnmt1* and/or *Dnmt3a* mutants in both neonatal and adult ISCs should resolve this question.

Our discovery of coordinated developmental changes in 3′ CGI methylation and expression of genes encoding glycosylation enzymes highlights the potential pathophysiological relevance of developmental epigenetics in adult ISCs. Glycosphingolipids and membrane glycoproteins decorate the interior structures and outer surfaces of all living cells and, together with the secreted mucins, contribute to the glycocalyx of the intestinal epithelial surface. It has been postulated that the control mechanisms by which developmental changes in enterocyte surface glycosylation patterns are regulated may have a direct impact on host and age susceptibility to pathogenic bacterial infection, inflammatory bowel disease, and cancer metastasis [47, 48]. In support of this conjecture, our findings of postnatal epigenetic development at *B4galnt1*, which encodes beta 1,4-N-acetylgalactosaminyltransferase 1 and normally undergoes transcriptional activation by DNA methylation at 3′ CGIs, are of particular interest. *B4galnt1* regulates postnatal development of colonic epithelial cells through the biosynthetic pathway of Sd^a^ blood group antigen [49], and is implicated in inflammatory bowel disease, colorectal and other types of cancer [50, 51].

Lastly, our studies suggested an epigenetic paradigm for a symbiotic relationship between the host and commensal microbes in the intestine. Studies in germ-free mice revealed that the intestinal microflora profoundly impacts intestinal physiology and mucosal immune functions. For example, germ-free mice are deficient in mucosal surface area and exhibit reduced crypt renewal rate [23, 52]. *In vivo* studies demonstrated that the resident bacteria have a glycosylation-modulating effect on the intestinal cell surface [20, 53]. By comparing germ-free and conventional mice, we found that the developmental establishment of intestinal DNA methylation patterns, particularly at the 3′ CGIs of glycosylation genes, was substantially blunted in the absence of gut microbiota. Further, this was not due to impaired *Dnmt1* activity. Given that the gut microbiome synthesizes critical cofactors required for one carbon metabolism [27, 28], it could be proposed that the methylation deficits result from a general lack of these metabolites. The overall maintenance of DNA methylation at generic repeats (Line1 and IAP in Fig. 5), however, suggested that, rather than simply facilitating methylation, the microbiome played a more sophisticated instructive role. Moreover, our observations in fecal transplant mice supported the direct link between methylation and the microbiome, and indicated that different genomic regions could have different responsiveness to microbiota at distinct developmental stages (i.e., pre-weaning versus after-weaning). Clearly, future studies will be needed to determine how microbial–host metabolite interactions contribute to the epigenetic effects — for example, by supplementing GF mice with dietary folate and/or folate-producing bacterial strains. Given the profound effects of the gut microbiome in human health and disease, it will be important to determine whether specific bacterial species are involved and whether there is a critical developmental period for the microbiota to influence ISC developmental epigenetics. If so, this may open the possibility for developmentally targeted probiotic therapies to provide lifelong protection against intestinal disease.

## Conclusions

We have shown that ISCs undergo important postnatal epigenetic development. Our report sheds light on endogenous and exogenous signals that guide the DNA methylation machinery to orchestrate intestinal epigenomic development, with potential important implications for lifelong gut health.

## Materials and methods

### Experimental animals

The mouse lines have been described previously: *Lgr5-EGFP-CreER* (also known as *Lgr5*^*EGFP-IRES-CreERT2*^) mice (backcrossed to a pure C57BL/6 J background) expressing EGFP and Cre recombinase under the control of the endogenous *Lgr5* regulatory sequences [4], *Villin-Cre* (*Tg(Vil-cre)997Gum*) mice (on the C57BL/6 J background) expressing Cre recombinase under the control of mouse *Villin1* promoter [54], *Dnmt3a*^*f/f*^ (also known as *Dnmt3a*^*2lox/2lox*^) mice carrying floxed exon 19 [55–57], and *Dnmt1*^*f/f*^ (also known as *Dnmt1*^*2lox/2lox*^) mice carry carrying floxed exons 4–5 [56, 58] (both are in a pure 129 background). Germ-free mice (C57BL/6 J) were maintained in three independent gnotobiotic animal facilities at Medical University of South Carolina, Brigham and Women’s Hospital and Baylor College of Medicine. PCR genotyping assays, including primer sequences and PCR conditions, are summarized in Additional file 2: Table S6. All animal research was carried out in accordance with the NIH Guide for Care and Use of Laboratory Animals and approved by the Baylor College of Medicine Animal Care and Use Committee (Protocol AN-6775).

### Tamoxifen treatment

To induce ISC-specific knockout, adult *Dnmt3a*^*f/f*^*; Lgr5-EGFP-CreER* or *Dnmt1*^*f/f*^*; Lgr5-EGFP-CreER* mice were intraperitoneally injected every other day with tamoxifen (1 mg in corn oil × three times). We examined the small intestines and colons 7 and 14 days after initial tamoxifen injection.

### Isolation of Lgr5^+^ ISCs and differentiated epithelial cells

EGFP-labeled Lgr5^+^ ISCs (Lgr5-EGFP^+^) and EpCAM-labeled epithelial cells (Lgr5-EGFP^-^/EpCAM^+^) were isolated based on a previously published method [13]. For genome-wide studies, mice were pooled to obtain sufficient amounts of ISCs (500 ng genomic DNA). We crossed *Lgr5-EGFP-CreER* mice with wild-type C57 mice, pooled 16 litters to obtain P0 samples, and 7 Lgr5-EGFP^+^ offspring to obtain P21 samples. For candidate gene studies, all animals were individually analyzed, except that for the embryonic and P0 time-points, mice were pooled from one litter and used as one biological sample. Flow cytometric analysis and cell sorting were performed using EGFP and anti-EpCAM antibody (Biolegend) on a four-way MoFlo cell sorter (Beckman-Coulter).

### Whole-genome DNA methylation profiling by bisulfite-sequencing

WGBS was performed and analyzed according to a previously published protocol [33]. Briefly, sonicated, adaptor-ligated genomic DNA was treated with sodium bisulfite by the EZ DNA Methylation-Direct kit (Zymo Research). The bisulfite-modified DNA was amplified (18 cycles) using adaptor-specific primers and fragments of 200–500 bp were isolated. The quantity and size distribution of libraries were determined using the Pico Green fluorescence assay and the Agilent 2100 Bioanalyzer, respectively. Each library was sequenced as 100-bp paired-end reads with planned sequencing depth of ~1 billion reads and 20× coverage per sample (Additional file 2: Table S7). The standard Illumina pipeline was used to perform base calling. After removing adaptor sequences and low-quality tails, reads were mapped to the mouse reference genome (mm9) by Bismark [59]. All the Bisulfite-Seq data have been uploaded to the Gene Expression Omnibus (GEO) with accession number [GEO:GSE58532].

### Whole-genome transcriptional profiling by RNA-Seq

RNA-Seq libraries were prepared as described previously [60] and sequenced on the Illumina HiSeq 2000 instrument with planned sequencing depth of 60 million reads per sample. We used BOWTIE2 software [61], which uses the Burrows-Wheeler transform for efficient realignment of RNA sequences. Cufflinks [62] was used for counting functions, quality control summaries, and fragments per kilobase per million reads (FPKM). All RNA-Seq data have been uploaded to GEO with accession number [GEO:GSE64054].

### Quantitative DNA methylation analysis by bisulfite-pyrosequencing

Methylation at specific CpG sites was quantified by bisulfite-pyrosequencing using the PyroMark Q96 MD instrument [63]. Primer sequences and sequencing assays are summarized in Additional file 2: Table S8. For each assay, set-up included positive controls (*SssI*-treated genomic DNA) and negative controls (whole genome amplified genomic DNA), mixing experiments to rule out bias, and repeated experiments to assess reproducibility. Annealing temperatures were optimized to overcome PCR bias as previously reported [64].

### Quantitative gene expression analysis by real-time RT-PCR

TaqMan gene expression assays (Applied Biosystems) were used to quantify mRNA of target genes. Assays were designed to have primers/probes to span exon–exon junctions (Additional file 2: Table S9). All experiments were carried out in triplicate and relative gene expression was calculated by the ratio of the target genes to *β-actin* expression on an ABI StepOnePlus Detection System.

### Histology, immunohistochemistry and immunofluorescence assays

For histological analyses, mouse intestines were fixed in 4 % paraformaldehyde. Fixed tissues were paraffin-embedded, sectioned, and stained with haematoxylin and eosin according to standard laboratory protocols at the Cellular and Molecular Morphology Core at the Texas Medical Center Digestive Diseases Center. Procedures for immunohistochemistry and confocal immunofluorescent analyses have been described [65]. The primary antibodies were anti-DNMT1 (sc-20701, Santa Cruz for immunohistochemistry and ab87654, Abcam for immunofluorescence), GFP (ab6673, Abcam) and lysozyme (AR024-5R, Biogenex). 5′-Ethynyl-2′-deoxyuridine (EdU) staining was performed using the Click-iT® EdU Alexa Fluor® 555 imaging kit (Invitrogen) according to the manufacturer’s instructions.

### Alcian blue staining

Slides were de-waxed, rehydrated to water, and incubated in Alcian Blue solution (0.01 g/ml Alcian blue in 3 % acetic acid, pH 2.5) for 30 minutes. The slides were then washed briefly in water and mounted using Cytoseal 60 (Richard-Allan Scientific).

### Bioinformatic analysis

Our main analyses of WGBS focus on all 200-bp ‘bins’ in the genome at approximately nucleosomal resolution as previously described [33]. For bins containing at least two CpG sites and covered by at least ten reads, we calculated the methylation level as the percentage of all methylated CpG site counts within each bin. For gene category annotation, we used RefSeq downloaded from the UCSC genome database. For CGI annotation, a list of 23,021 mouse CGIs was identified based on a previous report [66]. The methylation level for a gene or CGI category was computed as the mean of methylation levels of associated bins.

The enrichment of GO annotation terms was analyzed using the DAVID Functional Annotation Tool [67]. We identified gene associated mDMRs with methylation changes greater than 15 %, and divided them into eight groups: (1) methylation gain at 5′ CGI (N = 316); (2) methylation gain at gene body or 3′ CGI (N = 583); (3) methylation loss at 5′ CGI (N = 239); (4) methylation loss at gene body or 3′ CGI (N = 348); (5) methylation gain at 5′ non-CGI (N = 323); (6) methylation gain at gene body or 3′ non-CGI (N = 900); (7) methylation loss at 5′ non-CGI (N = 513); (8) methylation loss at gene body or 3′ non-CGI (N = 1710).

### FMT experiments

At age P25, the GF mice were orally inoculated with freshly prepared fecal slurries from at least two CNV donor mice, and subsequently housed in the conventional animal facility. For DNA methylation and gene expression analysis, we isolated colonic epithelial cells from three groups of mice at P100 under either GF (N = 3), FMT (N = 3), or CNV (N = 2) conditions. All mice were housed in the animal facilities at Baylor College of Medicine.

### Statistical analysis

Statistical significance of global methylation differences was determined based on a test for proportions with the 200-bp bin as the unit of observation. Statistical significance of the overlap with each GO category was determined by Fisher’s exact test and the *P* values were adjusted by Bonferroni correction to control false discovery rate (FDR). For the comparisons of candidate genes, quantitative DNA methylation and expression results are expressed as mean ± standard error of the mean. For both DNA methylation and gene expression data, we confirmed the normality of data by Kolmogorov-Smirnov test. Student’s *t* tests with two-tailed distribution were used to determine the significance of difference. *P* < 0.05 was considered statistically significant. Age -adjusted regression analysis was performed to assess the statistical correlation between DNA methylation and gene expression. To account for multiple testing, we controlled FDR and reported FDR-adjusted *P* values using the Benjaimini-Hochberg procedure.

## Additional files

Additional file 1:
**Supplemental Figures S1–S15.** (PDF 2922 kb) Additional file 2:
**Supplemental Tables S1–S9.** (PDF 2694 kb)

## Abbreviations

bp: base pair
CBC: crypt-based columnar
CGI: CpG island
CNV: conventional
dDMR: differentiation-associated differentially methylated region
DNMT: DNA methyltransferase
E: embryonic day
EGFP: enhanced green fluorescent protein
EpCAM: epithelial cell adhesion molecule
FACS fluorescence activated cell sorting FDR: false discovery rate
FMT: fecal microbiota transplant
GEO: Gene Expression Omnibus
GF: germfree
GO: Gene Ontology
ISC: intestinal stem cell
mDMR: maturation-associated differentially methylated region
P: postnatal day
PCR: polymerase chain reaction
UTR: untranslated region
WGBS: whole-genome bisulfite sequencing
