# Supplementary material for: Postnatal epigenetic regulation of intestinal stem cells requires DNA methylation and is guided by the microbiome
Source: Genome Biol. 2015 Sep 30;16:211. doi: 10.1186/s13059-015-0763-5 (PMC4589031; doi:10.1186/s13059-015-0763-5)
Supplement: Additional file 1: — Supplemental Figures S1–S15. (PDF 2922 kb) [file 13059_2015_763_MOESM1_ESM.pdf]

Additional file 1

Supplemental Figures

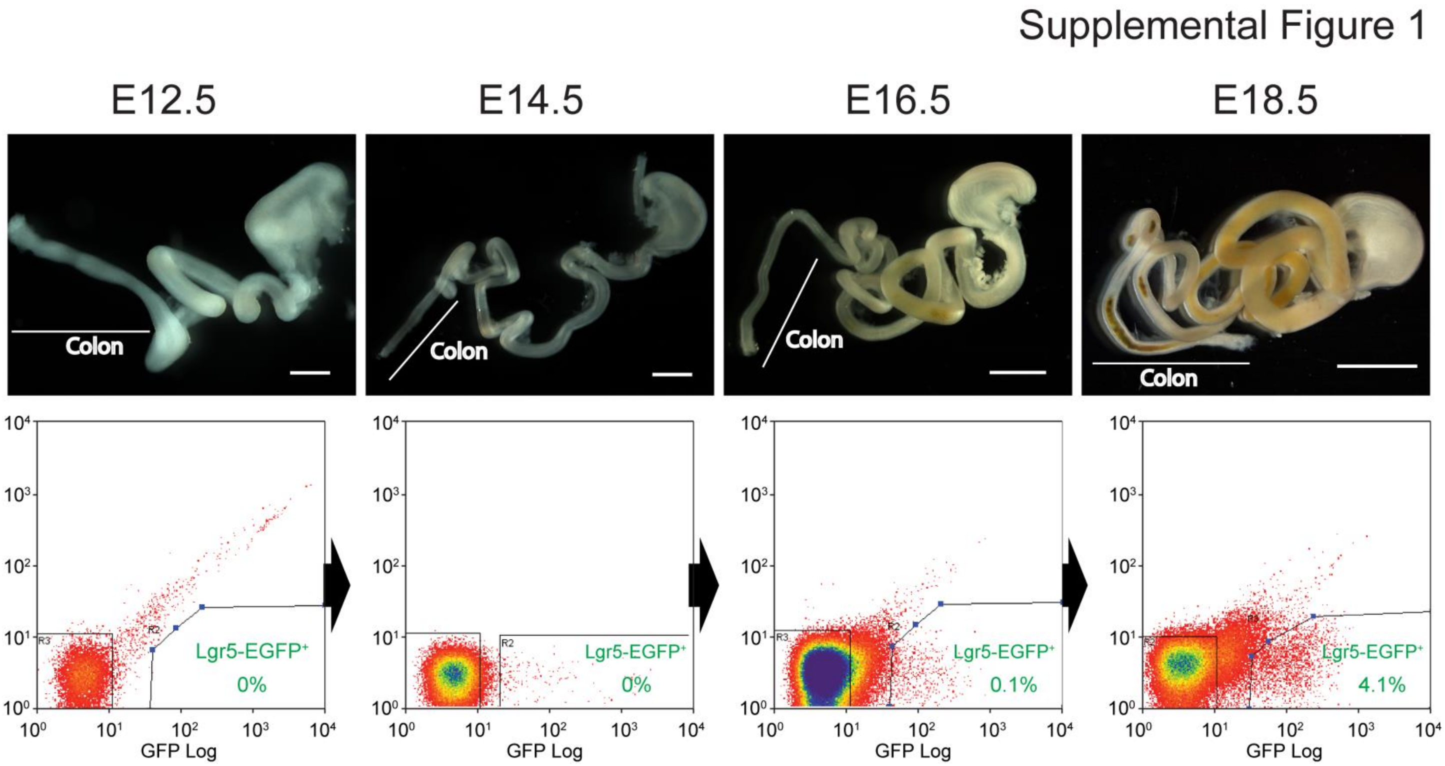

S1_Figure

The emergence of Lgr5^{+} colonic ISCs. The top panel shows representative dissections of the mouse GI tract at embryonic and fetal stages. Scale bars, 2000 µm. The bottom panel shows examples of fluorescence-activated cell sorting (FACS) of Lgr5^{+} colonic ISCs at corresponding stages. The percentages of Lgr5-eGFP^{+} cells were calculated based on sorted cells in the left and right gates.

Supplemental Figure 2

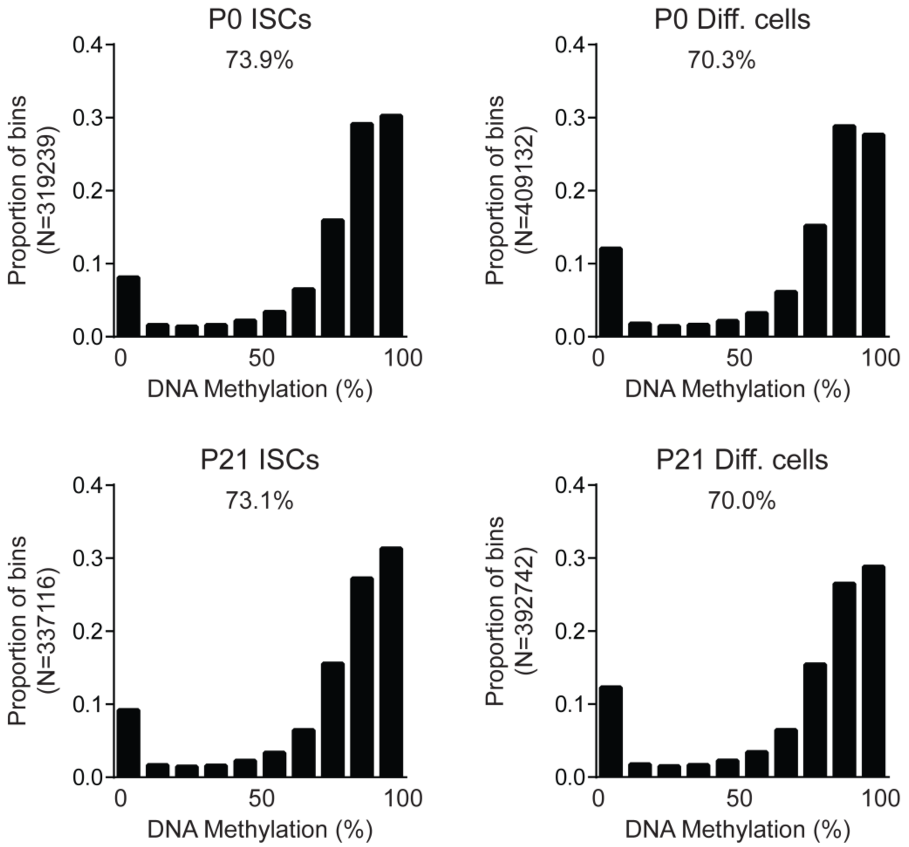

S2_Figure

Distribution of DNA methylation levels of individual informative 200bp genomic bins. The global methylation levels are indicated on top. We found that at both ages, global methylation levels were significantly lower in differentiated cells relative to ISCs (P<0.0001). Statistical significance of differences was determined based on test for proportions with unit of observation as the 200bp bin.

Supplemental Figure 3

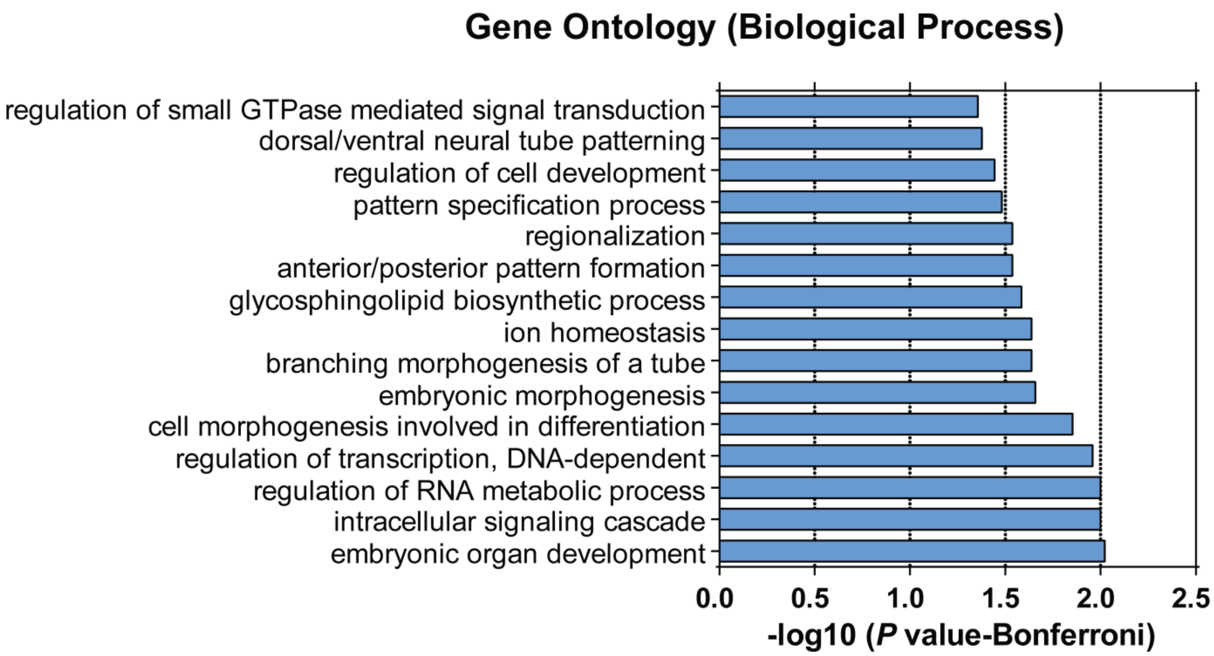

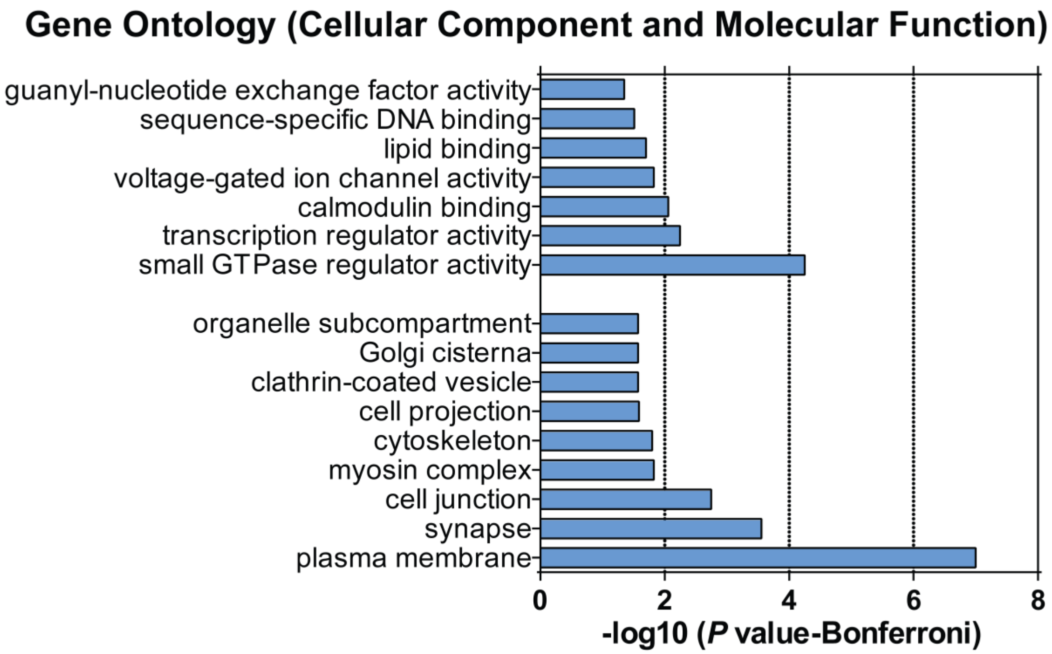

S3_Figure

GO analysis of genes associated with postnatal methylation gains at gene body or 3' CGIs. Bonferroni corrected *P*-values were –log10 transformed.

Supplemental Figure 4

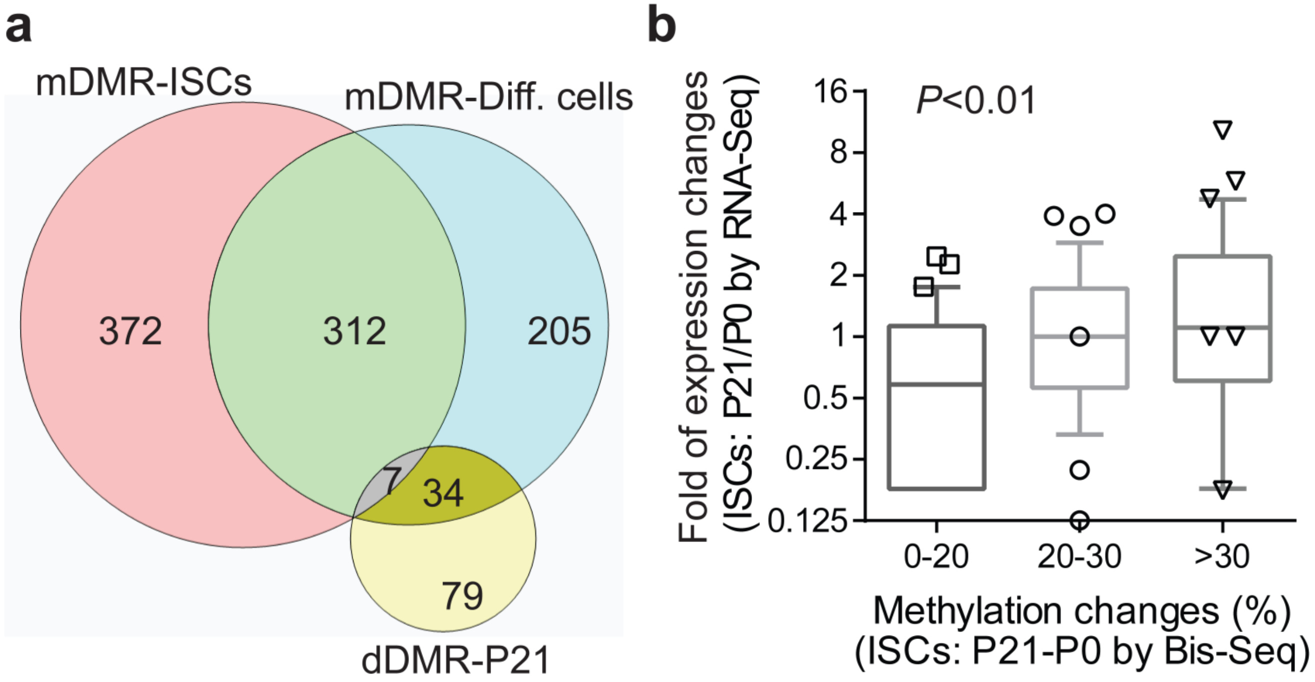

S4_Figure

(a) Comparison of CGI-associated mDMR genes found in ISCs versus those found in differentiated cells. (b) Expression changes of 3' CGI associated genes in ISCs during the suckling period. The genes are classified into three groups based on the degree of methylation changes (x-axis), and the box-and-whisker plots (10-90 percentile) compare the expression ratio (ISCs-P21/ISCs-P0) from RNA-Seq data among the three groups.

Supplemental Figure 5

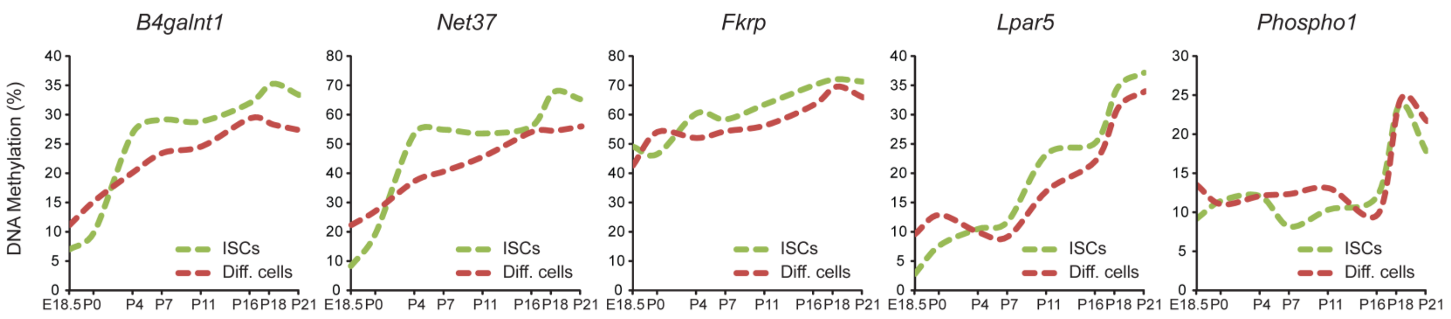

S5_Figure

Methylation profiling of 3' CGI genes at additional time-points during the suckling period.

Supplemental Figure 6

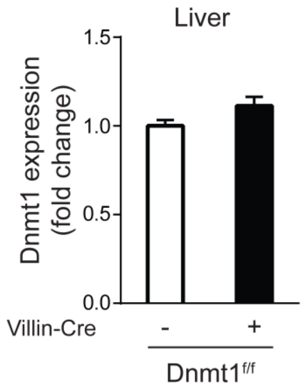

S6_Figure

Analysis of *Dnmt1* expression in liver tissues confirms that Villin-Cre mediated DNMT ablation is epithelial cell type specific.

Supplemental Figure 7

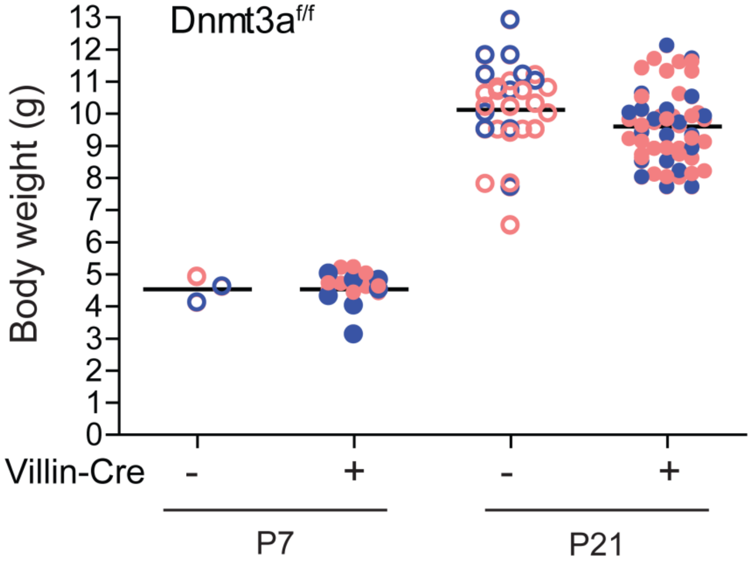

S7_Figure

Body weight of *Dnmt3a* mutant and control littermates at P7 and P21. Intestine-specific deletion was mediated by constitutive Villin-Cre transgene. Blue circles indicate males and red circles indicate female.

Supplemental Figure 8

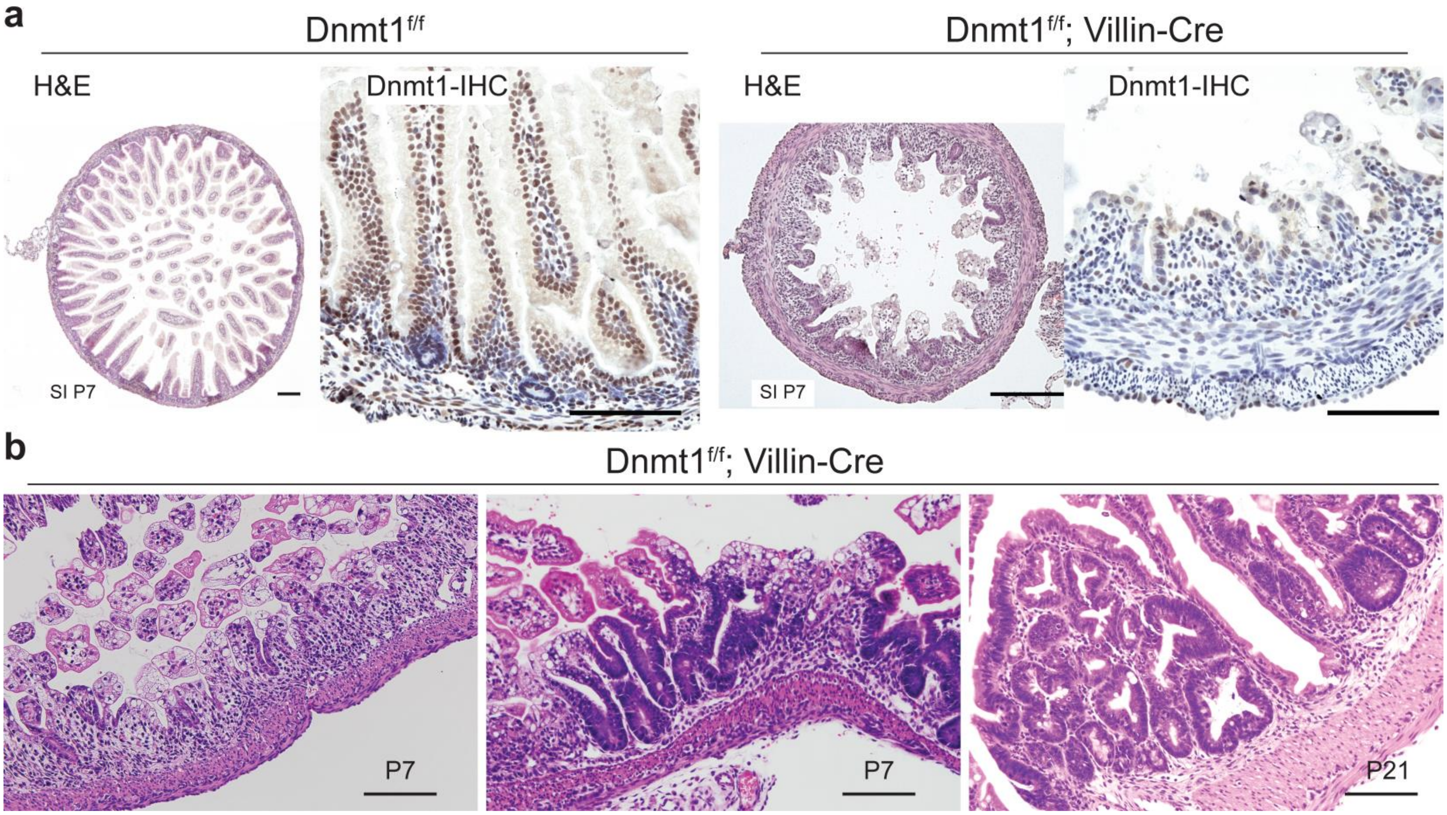

S8_Figure

Histology and immunohistochemical (IHC) analyses of small intestines with Villin-Cre mediated *Dnmt1* ablation. (a) Representative images of H&E (left) and Dnmt1 IHC (right) are shown for control (*Dnmt1*$^{f/f}$) and mutant (*Dnmt1*$^{f/f}$*; Villin-Cre*) mice at P7. **(b)** *Dnmt1* deletion in the developing small intestine causes various degrees of mucosal abnormality ranging from distorted and blunted villi to adenoma-like epithelial lesions in neonatal mice of P7 or older age (P21). Scale bars, 100 µm.

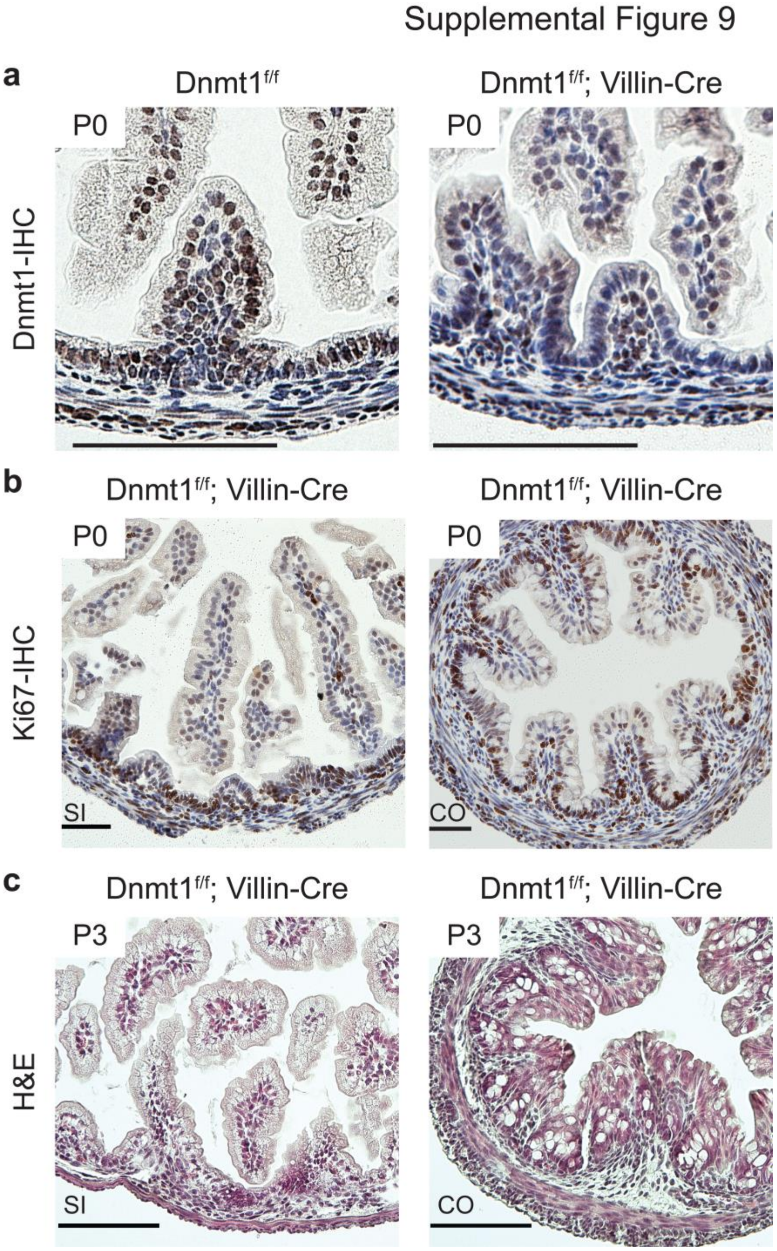

S9_Figure

Histology and immunohistochemical (IHC) analyses of Villin-Cre mediated *Dnmt1* mutant intestines at P0 (N=2) and P3 (N=2). (a) IHC staining for Dnmt1 in the control and mutant small intestines at P0. Epithelial loss of Dnmt1 expression was confirmed in the mutant mice. **(b)** IHC analysis of Ki67 in the *Dnmt1* mutant mice at P0. Normal morphologies and proliferation rates were found in the mutant small intestines (SI) and colons (CO). **(c)** Histological analysis suggested overall normal intestinal morphologies in *Dnmt1* mutant mice at P3. Scale bars, 100 μM.

Supplemental Figure 10

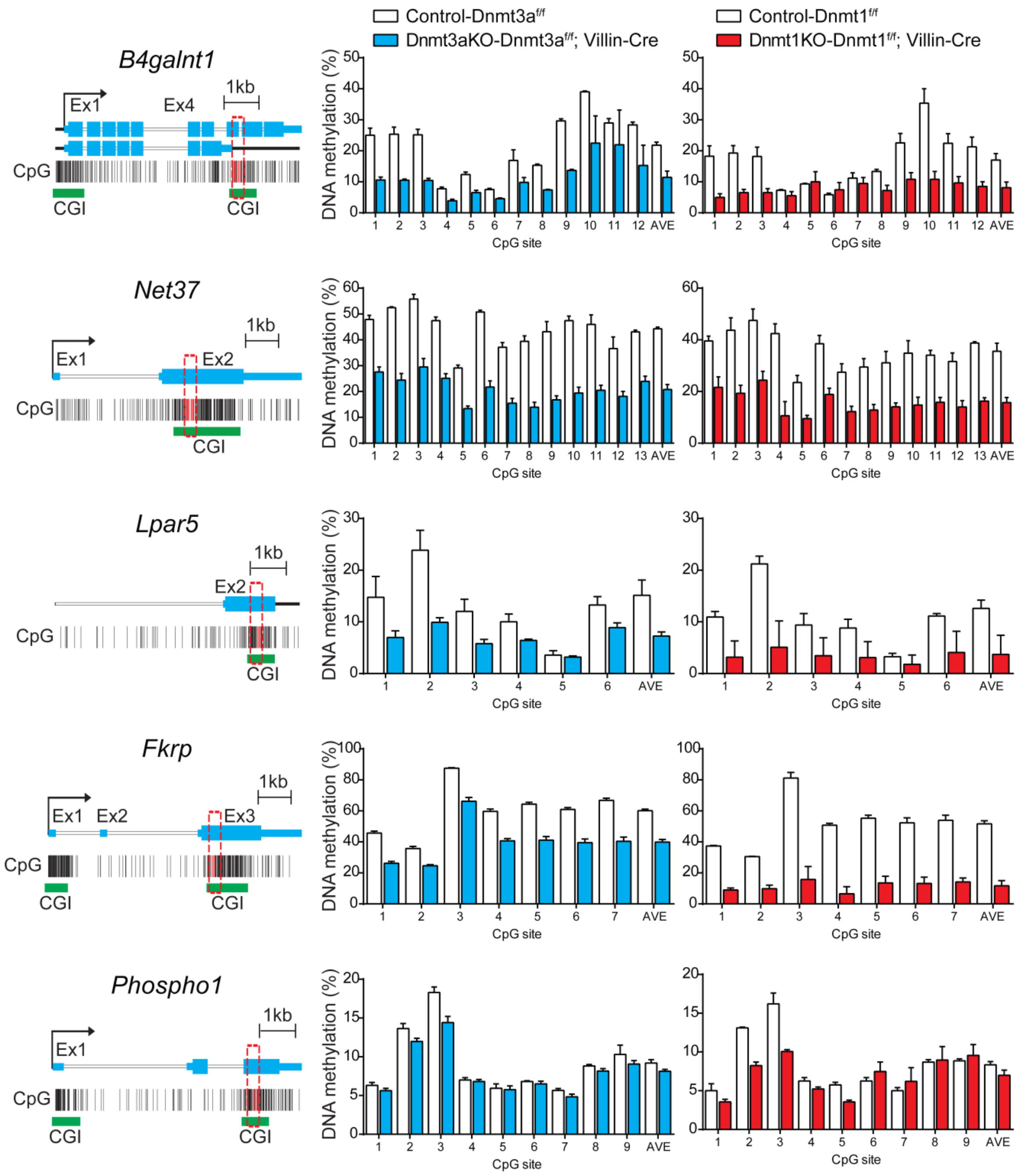

S10_Figure Quantitative DNA methylation analysis by bisulfite-pyrosequencing at single CpG sites in the intestinal epithelium from the control (*Dnmt*$^{f/f}$) and mutant (*Dnmt*$^{f/f}$;*Villin-Cre*) mice at P7. For each gene, CpG map is shown on the left. Each vertical line represents a CpG site and green bars indicate CGIs. Red dotted boxes indicate the bisulfite-pyrosequencing regions and CpG sites analyzed are highlighted in red. The levels of DNA methylation at individual CpG sites, as well as the average value of methylation at all CpG sites are shown for four groups of animals (legend indicated on the top).

Supplemental Figure 11

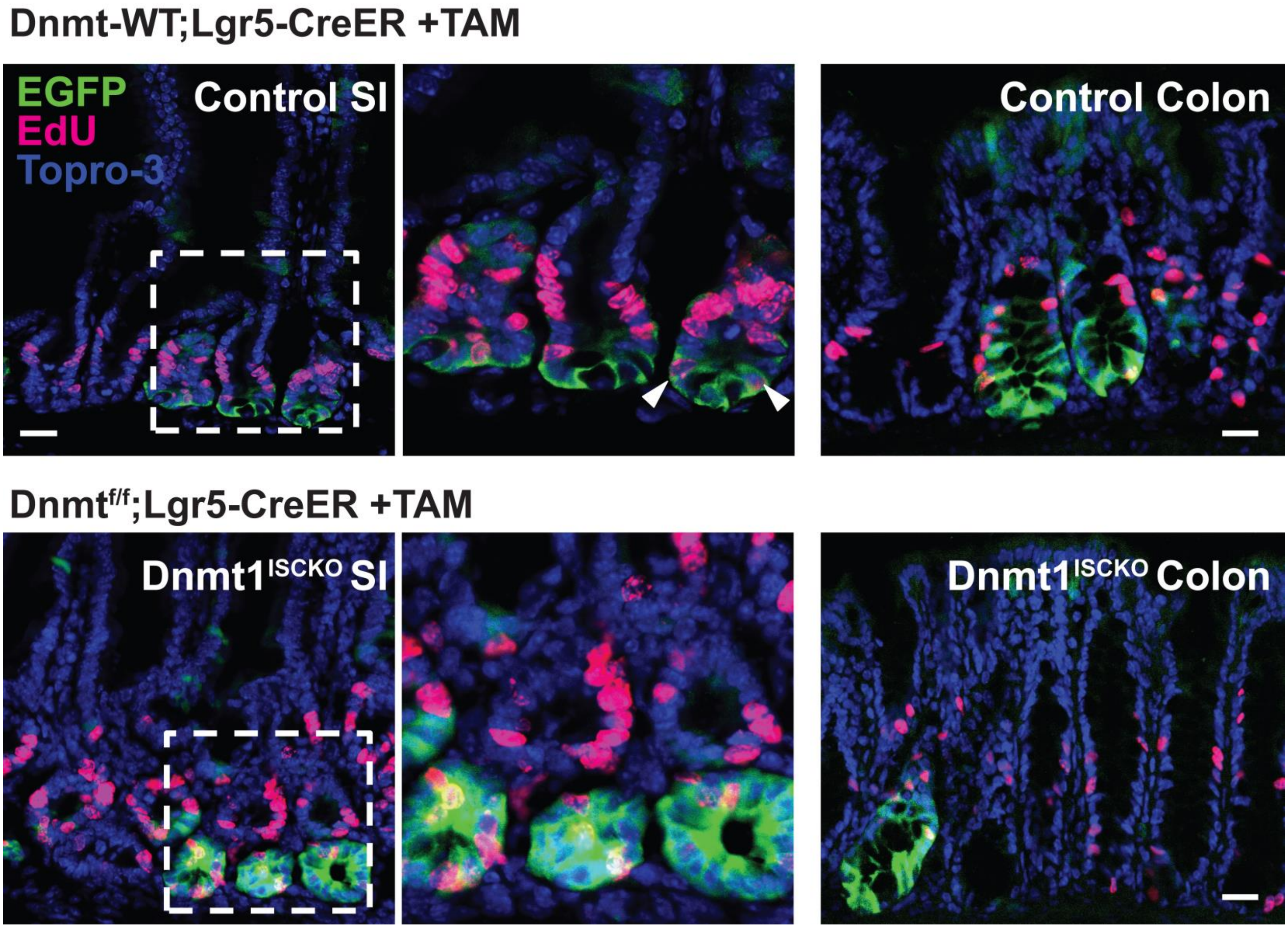

S11_Figure

Deletion of *Dnmt1* in adult ISCs alters morphology of CBCs in small intestine and colon.

Immunofluorescence analysis of ISCs (EGFP), proliferating cells (EdU) and nuclei (Topro-3) in the control and *Dnmt1^{ISCKO}* mice. Normal CBCs showed well-defined triangle shapes in both small intestine (arrowheads) and colon. Loss of Dnmt1 expression caused CBC aggregation into a closed ring pattern. EdU labeling showed slightly increased S-phase cells in the mutant crypts. For each SI section, there is a magnified view of the square area (middle panel).

Supplemental Figure 12

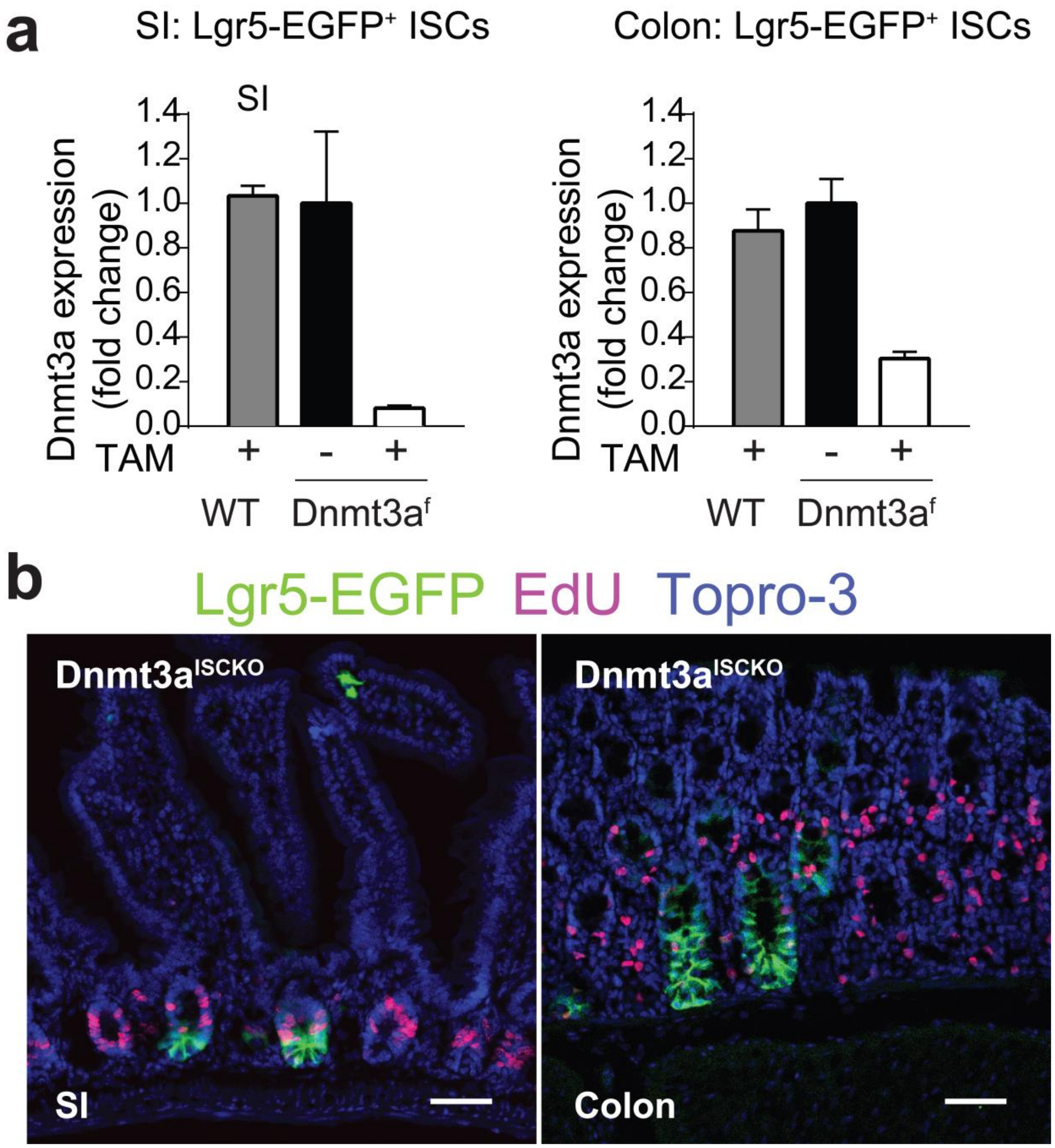

S12_Figure

Deletion of *Dnmt3a* in adult ISCs does not induce obvious phenotype. (a) After tamoxifen administration, RT-qPCR analysis confirms high efficiency of *Dnmt3a* deletion in isolated Lgr5-EGFP^{+} ISCs in both SI and colon. **(b)** Immunofluorescence analysis of ISCs (EGFP), proliferating cells (EdU) and nuclei (Topro-3) in the *Dnmt3a*^{ISCKO} mice shows an overall normal intestinal epithelial and crypt morphology, in both SI (left) and colon.

Supplemental Figure 13

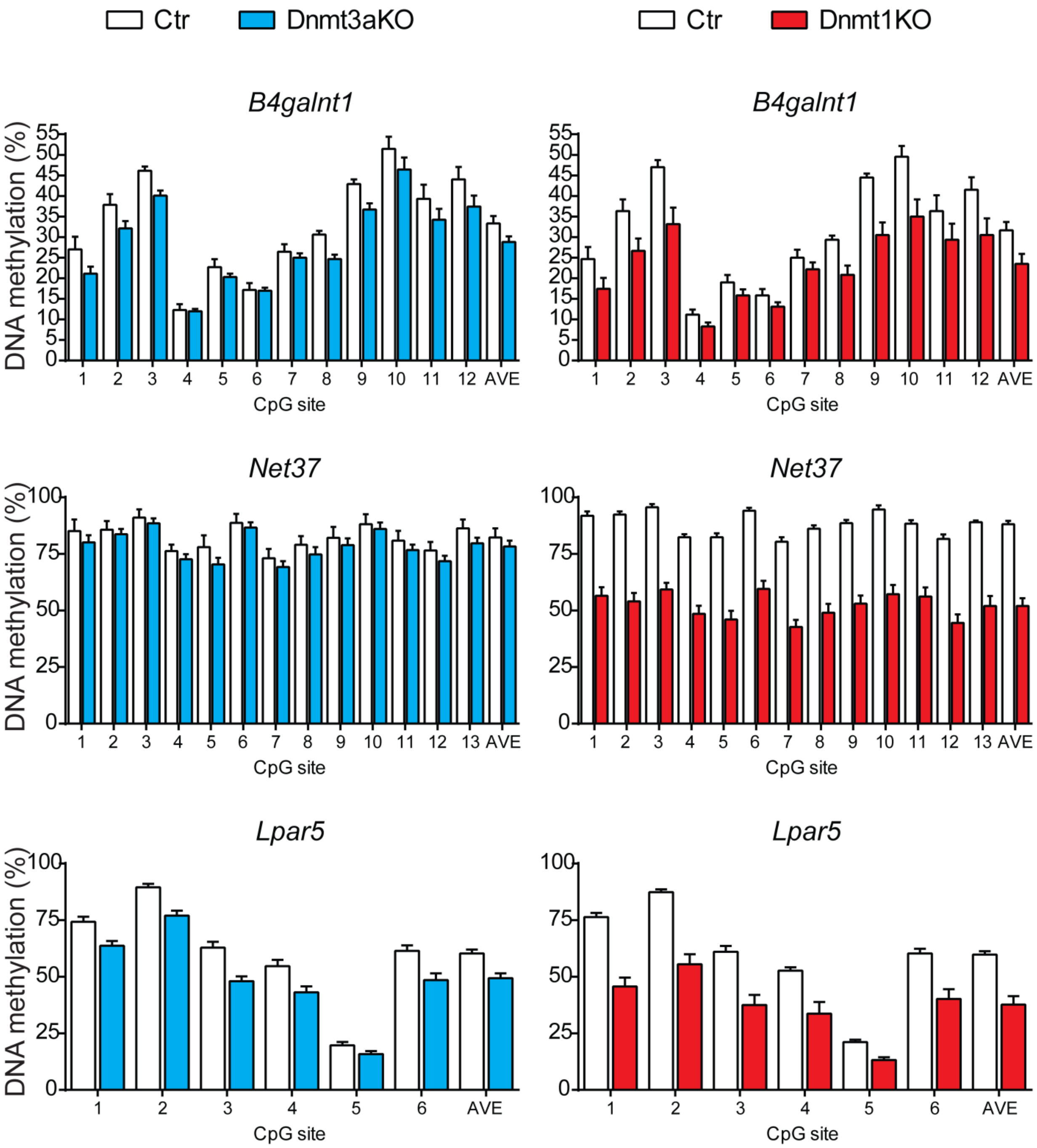

S13_Figure

Quantitative DNA methylation analysis by bisulfite-pyrosequencing at single CpG sites in the ISCs isolated from the control and *Dnmt*^{ISCKO} mice.

Supplemental Figure 14

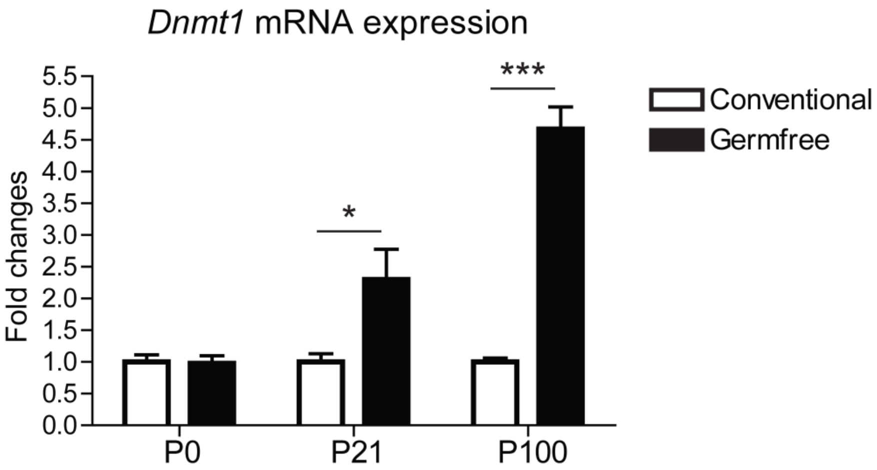

S14_Figure

mRNA expression of *Dnmt1* in mouse intestines under either conventional or germfree conditions at P21 and P100.

Supplemental Figure 15

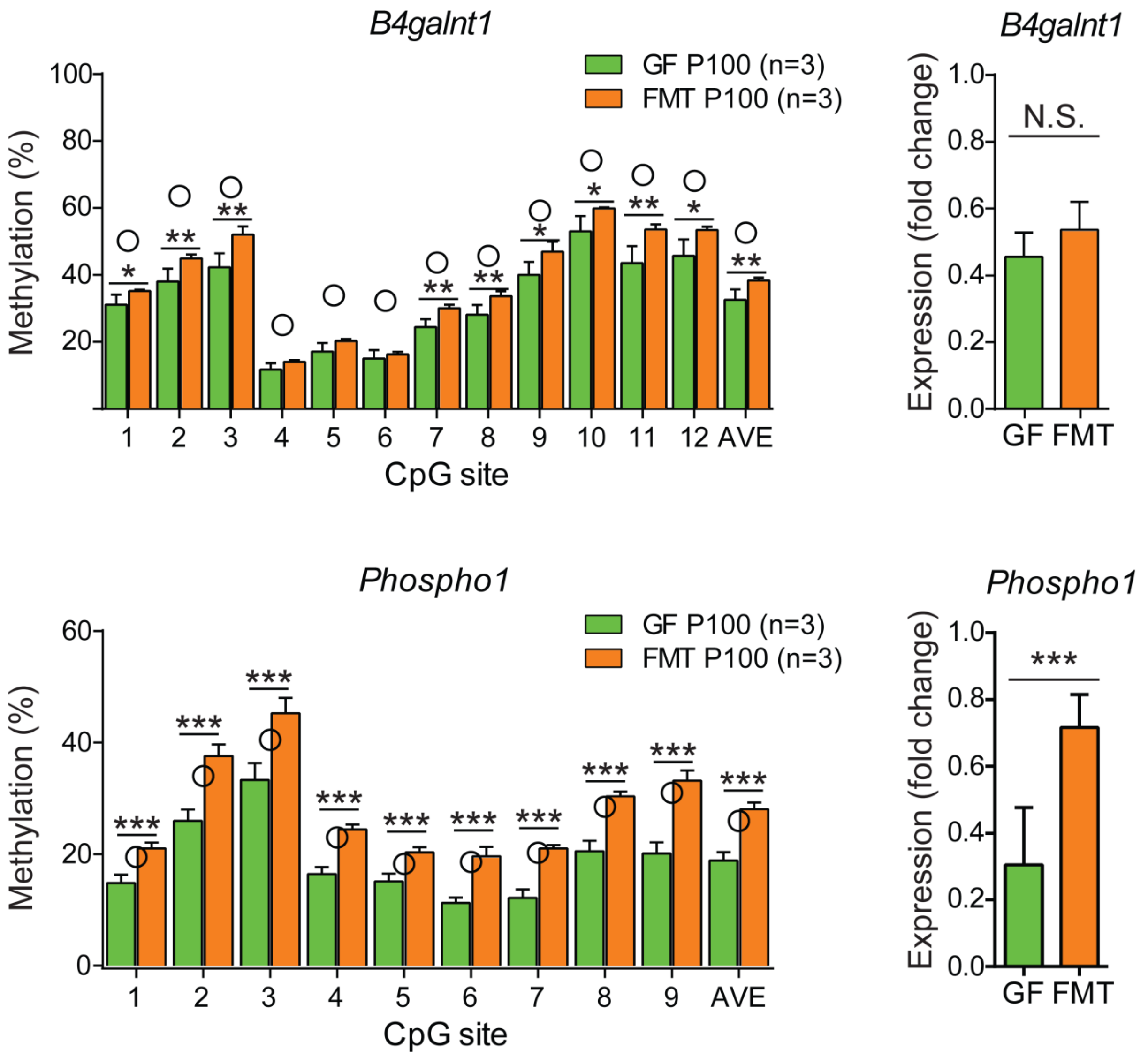

S15_Figure

Microbial colonization increases 3’ CGI methylation of *B4galnt1* (top) and *Phospho1* (bottom) genes. DNA methylation levels of colonic epithelial cells were compared between P100 mice under GF and FMT conditions. * *P*<0.05, ** *P*<0.01 and *** *P*<0.001. Empty circles indicate methylation levels in P100 CNV mice. At *B4galnt1*, FMT leads to modest, site-specific methylation increases that are not associated with expression change. For *Phospho1*, the increased 3’ CGI methylation in FMT mice leads to restored gene expression. Expression changes were relative to those under the CNV conditions.
