# Supplementary material for: Postnatal epigenetic regulation of intestinal stem cells requires DNA methylation and is guided by the microbiome
Source: Genome Biol. 2015 Sep 30;16:211. doi: 10.1186/s13059-015-0763-5 (PMC4589031; doi:10.1186/s13059-015-0763-5)
Supplement: Additional file 2: — Supplemental Tables S1–S9. (PDF 2694 kb) [file 13059_2015_763_MOESM2_ESM.pdf]

Additional file 2

Supplemental Tables

S1_Table. Lists of CGI mDMR genes

S2_Table. Lists of non-CGI mDMR genes

S3_Table. Significantly enriched GO categories associated with mDMR genes

S4_Table. Age-adjusted regression analysis of correlation between DNA methylation and gene expression

S5_Table. Composition of diets

S6_Table. Primers and PCR conditions for genotyping assays

S7_Table. Summary of WGBS results for genome-wide DNA methylation analysis

S8_Table. Bisulfite-pyrosequencing primers and sequenced regions for quantitative DNA methylation analysis

S9_Table. Real-time Taqman assays for gene expression analysis

Table S1. Lists of CGI mDMR genes

Group 1-mDMR genes associated with methylation gain at 5' CGI

							DNA Methylation Level (%)			
							ISCs		Diff. Cells	
Chr	Gene	Region	CGI	Start	End	#Bins	P0	P21	P0	P21
chr1	Gm973	Upstream	Y	59572108	59573107	1	23.7	66.7	20.9	64.2
chr11	Trim47	Upstream	Y	115971550	115972549	3	29.8	48.4	17.4	54.5
chr4	Marcksl1	Upstream	Y	129189825	129190824	2	29.0	62.9	19.6	55.7
chr15	Nrbp2	Upstream	Y	75920444	75921443	5	11.1	43.3	6.1	40.7
chr3	Mab21l2	Exon1	Y	86349503	86352205	8	24.2	75.8	37.0	70.5
chr4	Enho	Upstream	Y	41587336	41588335	3	39.9	56.9	0.2	31.0
chr15	Fam109b	Upstream	Y	82170609	82171608	3	42.0	59.0	28.4	58.6
chr8	Tradd	Exon1	Y	107783337	107783529	1	27.6	52.5	33.2	62.7
chr5	Ift81	5-UTR	Y	123061096	123064527	4	25.5	45.3	14.4	42.6
chr9	A730043L09Rik	5-UTR	Y	62091751	62091907	1	58.3	83.8	55.7	83.3
chr18	Camk4	Upstream	Y	33097695	33098694	2	27.4	52.1	18.2	45.5
chr2	Mvb12b	Upstream	Y	33743467	33744466	2	65.2	86.9	64.2	90.9
chr1	Gm973	5-UTR	Y	59573108	59573275	1	23.3	63.1	27.9	54.4
chr1	Gm973	Exon1	Y	59573108	59573362	1	23.3	63.1	27.9	54.4
chr8	Marveld3	Upstream	Y	112486106	112487105	2	22.6	53.6	0.6	26.0
chr16	Gp1bb	5-UTR	Y	18621674	18622496	4	24.8	58.4	21.2	46.0
chr16	Gp1bb	Exon1	Y	18621664	18622496	4	24.8	58.4	21.2	46.0
chr8	Cd97	Upstream	Y	86265211	86266210	2	9.4	31.6	7.9	32.5
chr16	Litaf	Upstream	Y	10993215	10994214	2	23.7	51.3	2.1	26.0
chr10	Kiss1r	Exon1	Y	79379716	79381662	5	1.3	70.8	23.4	47.2
chr7	Stx1b	Exon1	Y	134967774	134968045	2	23.3	41.5	7.2	30.8
chr16	Tiam1	Exon1	Y	89818206	89818597	2	3.6	33.9	1.9	25.4
chr9	Lysmd2	Upstream	Y	75472539	75473538	4	67.1	96.3	28.1	51.4
chr9	Rbp1	Exon1	Y	98325872	98326050	1	38.2	69.2	40.4	63.6
chr16	Gpr156	5-UTR	Y	37916582	37947809	3	13.2	37.6	0.2	23.3
chr6	Tfpi2	Exon1	Y	3918237	3918354	2	18.5	66.0	20.3	43.0
chr9	A730043L09Rik	Upstream	Y	62091908	62092907	1	25.8	56.2	23.5	45.9
chr8	Car7	Upstream	Y	107063707	107064706	2	11.5	40.3	19.5	41.3
chr2	Hoxd4	5-UTR	Y	74560035	74565335	8	33.4	72.0	47.1	68.7
chr2	Mir219-2	5-UTR	Y	29701151	29701247	1	53.5	82.0	56.4	77.7
chr2	Spef1	Exon1	Y	130997638	130997761	1	51.0	77.5	55.7	76.7
chr2	Cstad	Upstream	Y	30449564	30450563	2	2.9	29.1	11.0	31.4
chr2	Pltp	Upstream	Y	164683209	164684208	2	5.6	34.0	3.8	23.8
chr13	Irx4	5-UTR	Y	73397945	73402382	14	26.4	44.9	18.6	38.3
chr11	Shroom1	5-UTR	Y	53270707	53276756	4	40.2	56.4	8.3	27.8
chr17	Cldn6	5-UTR	Y	23816332	23818030	3	6.5	37.2	9.0	28.3
chr17	Cldn6	Exon1	Y	23816725	23816788	2	11.5	37.2	9.0	28.3
chr2	Chst14	Exon1	Y	118752233	118754319	5	43.9	72.9	17.2	36.2
chr7	Ccdc8	Exon1	Y	17579937	17581994	3	15.9	52.9	11.2	30.2
chr6	Eefsec	Upstream	Y	88396534	88397533	2	0.0	18.6	0.1	18.6
chr6	Ndnf	Upstream	Y	65620605	65621604	4	23.3	55.9	21.9	40.4
chr4	Foxe3	Upstream	Y	114598619	114599618	3	38.2	72.9	25.0	42.6
chr12	Clmn	Upstream	Y	106103287	106104286	3	53.2	79.4	60.7	77.7
chr10	Csrp2	Exon1	Y	110369011	110369123	2	29.5	58.0	33.3	50.2

chr7	Kcnq1ot1	5-UTR	Y	150399016	150482452	8	53.3	73.0	47.6	64.5
chr4	Ahdc1	5-UTR	Y	132567421	132617364	21	58.3	90.6	14.8	31.6
chr11	Myadml2	Exon1	Y	120508953	120509651	3	45.1	83.8	54.5	70.7
chr11	Hand1	Exon1	Y	57644746	57645649	5	31.2	53.1	25.3	41.3
chr18	Pcdhga8	5-UTR	Y	37885360	37885546	1	28.3	47.8	34.9	50.8
chr7	Nkx1-2	Upstream	Y	139791321	139792320	3	3.5	34.1	3.4	19.3
chr6	Hoxa2	Upstream	Y	52114831	52115830	3	51.0	90.8	51.6	67.4
chr2	Tspyl3	Exon1	Y	153048106	153051177	3	18.3	48.1	14.3	30.0
chr13	Mir1904	Upstream	Y	110693017	110694016	5	44.0	78.8	11.0	26.6
chr2	Epb4.1l1	Upstream	Y	156245788	156246787	4	54.3	91.1	25.7	41.3
chr5	Zfp316	5-UTR	Y	144026336	144031701	5	28.6	71.2	19.0	34.4
chr14	Zfhx2	5-UTR	Y	55694073	55710885	6	12.3	30.4	15.3	30.4
chr3	Mab21l1	Exon1	Y	55586432	55589209	8	21.0	55.9	34.3	49.4
chr10	Kiss1r	5-UTR	Y	79379716	79381418	4	1.3	64.1	16.1	30.6
chr10	1810043G02Rik	Upstream	Y	77440395	77441394	4	1.3	46.5	1.3	15.8
chr5	Htra3	Upstream	Y	36022432	36023431	2	59.1	74.8	58.9	73.3
chr15	Fam49b	Upstream	Y	63892011	63893010	3	37.4	75.3	23.7	38.0
chr7	Sct	Upstream	Y	148465031	148466030	2	15.2	38.0	8.0	21.9
chr8	B3gnt3	5-UTR	Y	74217783	74225699	4	0.9	35.6	16.7	30.6
chr9	Olfm2	Upstream	Y	20532659	20533658	2	11.3	45.4	16.5	30.0
chr7	Arhgef17	Exon1	Y	108077083	108080675	9	62.3	86.6	20.3	33.8
chr2	St6galnac4	Exon1	Y	32443101	32443201	1	6.0	27.5	17.5	30.8
chr7	Zbtb45	Exon1	Y	13592373	13593678	6	62.3	85.4	68.1	81.3
chr10	Bcr	Exon1	Y	74523641	74525055	7	6.4	41.9	5.2	18.3
chr7	Tmc5	5-UTR	Y	125740811	125766592	3	2.7	59.2	15.9	28.8
chr6	Hoxa3	Exon1	Y	52163039	52163066	1	0.7	25.6	1.8	14.7
chr6	Mgll	Upstream	Y	88673406	88674405	4	20.7	36.5	29.8	42.6
chr2	Tgif2	Upstream	Y	156664813	156665812	5	13.8	31.8	12.4	25.1
chr2	Hoxd3	5-UTR	Y	74550050	74582068	33	27.7	48.4	20.5	33.1
chr11	Rnd2	Exon1	Y	101330247	101330334	1	10.4	28.4	14.5	26.9
chr15	Pkdrej	Exon1	Y	85645106	85652163	4	4.8	38.9	13.7	25.9
chr12	Sstr1	Upstream	Y	59311791	59312790	2	17.8	37.1	16.7	28.9
chr8	Gm16793	Upstream	Y	36652075	36653074	5	16.1	37.1	42.8	54.7
chr9	Kbtbd13	Upstream	Y	65239460	65240459	2	35.5	77.9	37.0	48.8
chr6	Dennd2a	Upstream	Y	39507834	39508833	4	10.4	32.4	34.6	46.3
chr1	Itpkb	Exon1	Y	182262243	182264361	6	15.6	42.3	11.3	23.0
chr9	Mras	5-UTR	Y	99312005	99337131	4	69.0	86.6	20.3	31.6
chr11	Sox15	Exon1	Y	69468539	69469401	4	10.8	63.3	44.0	55.2
chr4	Sync	Exon1	Y	128970471	128971620	5	50.6	71.7	52.2	63.4
chr18	Pcdhga8	Exon1	Y	37885360	37887970	5	33.0	50.8	38.4	49.4
chr15	Acvrl1	Upstream	Y	100957953	100958952	1	18.8	52.8	32.8	43.8
chr17	Clic5	Upstream	Y	44324521	44325520	3	1.3	32.6	6.9	17.8
chr7	Kcnj11	5-UTR	Y	53355007	53356134	5	40.9	61.9	25.0	35.7
chr18	Zfp532	Exon1	Y	65739884	65740164	2	13.3	51.6	19.3	30.0
chr18	Zfp532	5-UTR	Y	65739884	65782651	8	13.3	51.6	5.2	15.6
chr6	Atp2b2	5-UTR	Y	113792325	113992074	16	8.0	38.7	22.8	33.2
chr6	Eno2	Upstream	Y	124719528	124720527	3	26.2	50.0	18.6	28.7
chr15	Apobec3	5-UTR	Y	79722838	79722946	1	11.8	44.8	17.8	27.9
chr15	Apobec3	Exon1	Y	79722838	79722999	1	11.8	44.8	17.8	27.9

chr1	B3gnt7	Upstream	Y	88198796	88199795	2	9.0	38.3	9.7	19.6
chr7	Catsperg1	Upstream	Y	29999053	30000052	1	9.0	27.2	9.1	18.7
chr9	Csrnp1	Exon1	Y	119893752	119893776	1	25.0	43.7	30.9	40.4
chr5	Cad	Upstream	Y	31356184	31357183	3	4.9	25.1	3.1	12.4
chr19	Fads3	Upstream	Y	10115038	10116037	4	58.0	77.6	37.3	46.2
chr15	Pvt1	Upstream	Y	61868542	61869541	4	37.9	63.1	30.3	39.2
chr1	Psen2	5-UTR	Y	182175886	182186431	5	28.1	44.2	19.2	27.9
chr9	Cgnl1	Upstream	Y	71619410	71620409	2	28.8	50.4	17.8	26.5
chr9	Phldb1	5-UTR	Y	44536163	44543281	9	21.0	44.4	5.3	13.9
chr9	Nfrkb	Upstream	Y	31192777	31193776	3	32.2	47.5	10.5	19.1
chr7	Fam53b	5-UTR	Y	139970897	140004879	6	14.3	65.2	39.2	47.8
chr10	Nfic	Upstream	Y	80889919	80890918	3	30.3	53.9	24.9	33.3
chr13	Atxn1	5-UTR	Y	45663787	46060360	12	0.3	29.8	30.4	38.8
chr11	Fzd2	Exon1	Y	102465745	102469372	12	5.5	26.3	5.4	13.7
chr7	Gramd1a	Upstream	Y	31936070	31937069	4	11.2	51.5	18.3	26.0
chr9	Fxyd2	Exon1	Y	45216189	45216227	1	60.0	83.3	70.3	78.0
chr5	Rilpl1	Upstream	Y	124981401	124982400	3	26.6	51.4	16.2	23.8
chr17	Caskin1	Upstream	Y	24624728	24625727	4	36.7	66.7	14.3	21.9
chr2	4933433C11Rik	Exon1	Y	25068809	25069103	1	60.8	76.0	66.2	73.7
chr6	Scnn1a	Exon1	Y	125271358	125272392	4	6.7	38.6	35.1	42.5
chr12	4921506M07Rik	Exon1	Y	58676789	58677673	5	5.3	23.7	6.7	13.9
chr13	Irx4	Upstream	Y	73396945	73397944	3	43.1	68.3	36.3	43.3
chr7	Fancf	Exon1	Y	59115949	59117637	7	0.0	46.2	20.4	27.3
chr15	Bai1	Exon1	Y	74359360	74360370	5	10.8	28.4	14.5	21.4
chr17	Prss41	5-UTR	Y	23981105	23981123	1	0.0	15.4	5.1	11.9
chr14	Stk24	Upstream	Y	121778453	121779452	5	1.7	23.9	2.5	9.3
chr8	Hsbp1	Upstream	Y	121867438	121868437	3	0.4	24.0	22.7	29.3
chr11	Rasl10b	Upstream	Y	83222574	83223573	5	8.7	40.9	7.6	14.2
chr11	2810433D01Rik	Upstream	Y	102485731	102486730	2	62.7	80.9	70.7	77.2
chr10	H2afy2	5-UTR	Y	61220644	61246612	6	4.5	32.4	2.6	9.1
chr3	Rhoc	5-UTR	Y	104591952	104594814	4	26.1	48.2	11.9	18.3
chr3	Ccna1	Upstream	Y	54858978	54859977	5	16.7	33.9	2.9	9.4
chr6	Slc41a3	5-UTR	Y	90554874	90561902	3	8.6	24.3	7.8	14.2
chr2	Fam78a	Exon1	Y	31938104	31939225	6	4.5	23.0	12.2	18.6
chr2	Smox	5-UTR	Y	131317598	131337745	5	18.2	41.5	4.3	10.6
chr4	Fam110b	5-UTR	Y	5571326	5725730	6	6.7	25.5	4.6	10.5
chr2	Nebl	Upstream	Y	17652696	17653695	3	7.1	23.3	2.8	8.7
chr11	Morc2a	5-UTR	Y	3549497	3550382	3	36.0	86.9	24.6	30.4
chr11	Morc2a	Exon1	Y	3549497	3550149	3	36.0	86.9	24.6	30.4
chr14	Gm15217	Exon1	Y	46999954	47000061	1	47.8	64.0	49.9	55.6
chr10	Csrp2	5-UTR	Y	110357232	110369011	4	10.4	34.1	9.7	15.5
chr17	Mllt1	Upstream	Y	57074812	57075811	4	1.0	46.5	15.8	21.6
chr11	Phospho1	Upstream	Y	95684814	95685813	5	4.9	29.4	9.4	15.1
chr13	Smad5	Upstream	Y	56803371	56804370	4	1.8	17.2	6.8	12.5
chr14	Slc39a14	Upstream	Y	70751232	70752231	2	13.6	34.8	4.2	9.9
chr2	Pamr1	Upstream	Y	102389178	102390177	2	22.7	39.7	10.7	15.9
chr15	Prph	Upstream	Y	98884605	98885604	2	11.9	27.1	8.2	13.3
chr15	D730005E14Rik	5-UTR	Y	79719962	79723568	3	11.5	30.7	12.1	17.3
chr19	Hps6	Exon1	Y	46077968	46080663	6	66.7	85.7	27.2	32.3

chr8	Pcnxl2	Upstream	Y	128422194	128423193	2	17.6	41.7	14.8	19.9
chr7	Ifitm10	5-UTR	Y	149557006	149558164	5	24.3	59.6	18.2	23.1
chr2	Prdm11	5-UTR	Y	92854041	92886301	14	0.0	37.9	12.3	17.3
chr16	Bace2	Upstream	Y	97577335	97578334	2	17.8	38.6	14.8	19.6
chr11	Metrnl	Upstream	Y	121562741	121563740	2	8.9	27.8	7.4	12.2
chr16	Mir193b	Upstream	Y	13448616	13449615	2	7.7	51.0	13.6	18.4
chr5	Tbc1d1	5-UTR	Y	64551450	64564718	5	0.3	44.4	18.3	23.0
chr9	Eepd1	Exon1	Y	25289866	25290904	2	45.0	61.4	62.6	67.1
chr16	Tiam1	5-UTR	Y	89898813	89974944	13	47.6	68.3	60.0	64.5
chr19	Peli3	5-UTR	Y	4941912	4943092	4	50.0	71.4	14.1	18.5
chr17	Prss41	Exon1	Y	23981044	23981123	2	1.7	30.4	15.1	19.4
chr9	Clk3	Upstream	Y	57613668	57614667	5	49.3	64.8	16.5	20.8
chr15	Plec	Upstream	Y	76061809	76062808	3	15.1	33.6	14.1	18.4
chr1	Syt2	5-UTR	Y	136543258	136637428	12	4.8	26.8	18.5	22.8
chr2	Eef1a2	Upstream	Y	180891721	180892720	2	3.2	42.5	37.9	42.1
chr1	Nos1ap	Upstream	Y	172519981	172520980	3	3.9	21.0	4.5	8.6
chr7	Lipe	Upstream	Y	26181007	26182006	5	13.0	43.7	42.1	46.2
chr14	Sox21	Exon1	Y	118632456	118636252	14	0.3	29.6	8.0	11.9
chr17	Cbs	5-UTR	Y	31769973	31774150	5	12.8	37.1	3.4	7.3
chr9	Mpi	Upstream	Y	57400560	57401559	3	28.0	50.1	49.8	53.7
chr7	Kcnc1	Upstream	Y	53650867	53651866	4	4.7	27.8	7.9	11.8
chr12	Rps6kl1	5-UTR	Y	86490839	86492214	3	13.8	29.9	6.9	10.7
chr7	Mir293	Upstream	Y	3219344	3220343	2	23.8	38.9	26.4	30.2
chr7	Zfp94	5-UTR	Y	25096499	25101685	3	19.7	40.3	18.4	22.2
chr1	Slc45a3	5-UTR	Y	133859492	133873517	6	23.3	61.7	8.0	11.8
chr17	Syngr3	Upstream	Y	24826895	24827894	2	54.7	70.9	28.8	32.5
chr3	Gm9833	Upstream	Y	10087277	10088276	3	58.7	76.7	58.9	62.5
chr2	Gata5	Upstream	Y	180069385	180070384	3	25.8	42.6	11.2	14.7
chr11	E130012A19Rik	Exon1	Y	97488701	97491030	10	10.3	26.4	3.9	7.4
chr11	Zkscan17	Upstream	Y	59320143	59321142	2	16.8	32.8	5.8	9.3
chr7	Doc2a	Upstream	Y	133990067	133991066	2	33.8	55.6	43.2	46.6
chr10	Mier2	Upstream	Y	79017837	79018836	4	6.5	45.6	27.3	30.7
chr9	Zfp280d	Upstream	Y	72121706	72122705	2	39.9	83.7	39.4	42.7
chr6	Mira	Upstream	Y	52165288	52166287	4	11.2	28.2	12.9	16.2
chr5	Stag3	5-UTR	Y	138721737	138723102	3	38.4	88.0	41.5	44.8
chr2	Rapgef1	Upstream	Y	29474240	29475239	3	24.6	58.4	40.7	43.9
chr19	Gldc	Upstream	Y	30249932	30250931	2	0.5	21.1	5.3	8.5
chr7	Pwwp2b	Upstream	Y	146433381	146434380	5	0.0	15.5	3.1	6.3
chr13	Pom121l2	Upstream	Y	22072063	22073062	1	0.0	20.8	11.9	15.1
chr12	Gpr135	Upstream	Y	73171979	73172978	3	53.1	90.0	30.9	34.0
chr16	2510009E07Rik	Upstream	Y	21694739	21695738	5	5.2	24.8	4.2	7.3
chr6	Gxylt2	Upstream	Y	100653728	100654727	2	34.1	73.1	34.1	37.1
chr11	Prss38	Upstream	Y	59189156	59190155	1	55.9	71.8	69.0	72.0
chr1	Phlda3	Exon1	Y	137662662	137663471	5	15.6	49.4	4.0	7.0
chr17	Tdrd6	Exon1	Y	43761056	43767248	7	32.3	52.6	14.2	17.1
chr15	Mgat3	Upstream	Y	80003151	80004150	4	35.5	53.3	28.4	31.3
chr4	Npr2	Exon1	Y	43644807	43645723	4	14.9	49.1	44.5	47.5
chr11	Kat2a	Upstream	Y	100573782	100574781	2	0.7	38.2	32.0	34.7
chr7	Ggn	Exon1	Y	29955877	29958119	11	0.8	21.6	25.8	28.5

chr1	4930444P10Rik	Upstream	Y	16083123	16084122	3	10.3	28.6	6.0	8.7
chr19	Add3	5-UTR	Y	53217246	53291310	7	23.3	43.1	12.1	14.8
chr15	Trps1	5-UTR	Y	50678487	50721587	11	0.1	15.9	5.1	7.7
chr4	Nmnat1	5-UTR	Y	148848652	148859251	5	32.8	70.1	58.5	61.1
chr12	Mta1	Upstream	Y	114335489	114336488	5	24.6	56.9	14.3	16.8
chr2	Gpr176	Upstream	Y	118199156	118200155	5	25.7	59.0	14.1	16.5
chr8	Zfp358	Upstream	Y	3492138	3493137	2	16.5	46.6	18.1	20.5
chr9	Snx19	Exon1	Y	30234914	30236841	2	19.9	44.5	24.2	26.6
chr1	Dnm3	Upstream	Y	164408166	164409165	2	25.0	49.1	28.7	31.1
chr4	Lzic	Upstream	Y	148858442	148859441	3	5.1	23.6	7.7	10.0
chr6	Cecr6	Upstream	Y	120443826	120444825	3	0.7	24.6	8.3	10.6
chr11	Slc35e4	Exon1	Y	3812572	3814667	10	0.7	18.0	9.3	11.5
chr5	Gpc2	Upstream	Y	138721166	138722165	5	28.9	44.5	24.9	27.1
chr7	Mir291b	5-UTR	Y	3219483	3219561	1	19.3	36.9	19.0	21.2
chr7	Zfp583	Upstream	Y	6282037	6283036	3	21.6	59.1	15.6	17.8
chr7	Chst8	5-UTR	Y	35533211	35597730	9	0.6	76.0	29.6	31.7
chr6	Scnn1a	5-UTR	Y	125271358	125271898	2	6.7	21.9	16.7	18.7
chr6	Reep1	Upstream	Y	71656675	71657674	4	16.7	44.1	40.5	42.5
chr11	Sphk1	Upstream	Y	116392225	116393224	4	34.1	50.3	24.8	26.7
chr1	Lemd1	5-UTR	Y	134088013	134124798	4	1.0	18.6	11.1	13.0
chr19	Sgms1	5-UTR	Y	32234655	32462944	10	1.2	25.7	1.3	3.0
chr3	Phf17	5-UTR	Y	41359656	41385142	19	21.6	38.9	13.0	14.6
chr15	Kdelr3	Upstream	Y	79345838	79346837	3	2.2	28.9	9.7	11.2
chr2	Patl2	5-UTR	Y	121955253	122011925	5	0.2	17.8	15.7	17.2
chr4	Cep85	Upstream	Y	133743001	133744000	3	4.4	20.7	3.3	4.6
chr13	Dcdc2a	Upstream	Y	25146873	25147872	5	0.4	17.1	6.4	7.7
chr2	Olfm1	Upstream	Y	28047613	28048612	3	9.6	27.3	23.3	24.6
chr4	Tmeff1	Upstream	Y	48597065	48598064	4	3.5	42.1	1.2	2.2
chr2	Vsx1	Upstream	Y	150514874	150515873	2	12.1	41.3	46.5	47.5
chr2	Eif3m	Upstream	Y	104857185	104858184	3	0.0	36.7	0.1	1.0
chr11	Map3k14	5-UTR	Y	103103685	103128715	6	41.8	60.3	42.1	42.9
chr2	Vps16	Upstream	Y	130249056	130250055	5	46.5	94.1	24.9	25.8
chr9	Gpr62	Exon1	Y	106366291	106368271	8	26.0	43.1	9.5	10.4
chr2	Cdh4	Upstream	Y	179176183	179177182	4	17.6	34.5	15.4	16.3
chr4	Fndc5	Upstream	Y	128813304	128814303	5	50.0	71.4	73.3	74.1
chr16	Snn	5-UTR	Y	11066391	11072411	5	12.8	30.8	0.6	1.3
chr10	Cdh23	5-UTR	Y	60120082	60159238	14	9.3	40.3	11.8	12.5
chr15	Tubgcp6	Upstream	Y	88953581	88954580	3	1.4	17.7	12.0	12.7
chr8	4930444A02Rik	Upstream	Y	27104594	27105593	2	19.1	35.4	17.1	17.7
chr2	Prrx2	Upstream	Y	30699887	30700886	4	43.9	70.0	34.0	34.6
chr9	4933433G15Rik	5-UTR	Y	75257991	75258573	2	7.0	27.1	9.2	9.7
chr9	Mapk6	Upstream	Y	75257822	75258821	3	7.0	27.1	6.2	6.7
chr11	Rasl10a	Upstream	Y	4957131	4958130	3	21.1	41.5	11.0	11.4
chr11	Mmgt2	5-UTR	Y	62462166	62478329	4	0.0	29.8	21.9	22.3
chr11	Doc2b	Upstream	Y	75609560	75610559	5	41.5	69.9	44.1	44.4
chr12	BC022687	Upstream	Y	114046186	114047185	3	5.3	23.8	20.6	21.0
chr11	Gcgr	5-UTR	Y	120392041	120396070	3	9.4	26.5	4.8	5.2
chr15	11-Mar	Upstream	Y	26237827	26238826	4	8.3	42.6	1.4	1.7
chr11	Cog1	Upstream	Y	113509843	113510842	4	5.5	22.7	30.6	31.0

chr13	Prr7	5-UTR	Y	55565628	55573385	11	3.5	18.9	9.5	9.8
chr2	Ocstamp	Exon1	Y	165222721	165223720	3	64.0	86.5	78.6	78.8
chr5	Kcnk3	Upstream	Y	30889543	30890542	4	0.0	31.0	3.5	3.7
chr1	Abl2	Upstream	Y	158487918	158488917	3	1.1	27.1	0.6	0.8
chr4	Atg4c	5-UTR	Y	98860625	98879326	6	60.9	93.0	38.0	38.1
chr8	Smpd3	5-UTR	Y	108789820	108861888	8	5.0	22.6	17.7	17.8
chr13	Ddx46	Upstream	Y	55735388	55736387	2	28.1	43.5	10.9	10.9
chr2	Rims4	Upstream	Y	163744420	163745419	5	11.1	52.7	53.5	53.4
chr11	Coa3	Upstream	Y	101140263	101141262	4	1.6	30.9	27.3	27.2
chr13	Spock1	Upstream	Y	58009694	58010693	2	0.3	22.6	14.9	14.7
chr7	Smg1	Upstream	Y	125387152	125388151	5	0.0	16.7	0.7	0.5
chr14	Entpd4	Upstream	Y	69954208	69955207	3	3.0	26.8	2.0	1.7
chr2	Hrh3	Upstream	Y	179839113	179840112	5	5.0	20.1	11.7	11.4
chr4	Adprhl2	Upstream	Y	125998948	125999947	4	35.3	50.3	48.1	47.7
chr16	Col8a1	5-UTR	Y	57632752	57754850	3	0.0	47.6	62.9	62.4
chr14	5031414D18Rik	5-UTR	Y	75415834	75431710	4	17.4	34.1	7.9	7.2
chr5	Hopx	Upstream	Y	77544149	77545148	1	4.3	29.6	12.3	11.5
chr13	Ror2	Upstream	Y	53381479	53382478	3	3.0	29.1	20.2	19.5
chr13	Arsb	Upstream	Y	94540634	94541633	3	0.9	26.1	1.1	0.4
chr17	Jmjd8	Upstream	Y	25964988	25965987	3	0.0	23.5	17.4	16.5
chr10	Dram1	Upstream	Y	87819821	87820820	3	25.3	60.8	20.1	19.2
chr10	Pdxk	Upstream	Y	77927694	77928693	3	1.1	21.5	15.5	14.4
chr16	Tango2	5-UTR	Y	18324506	18344025	4	0.0	32.1	17.1	16.0
chr10	Hcfc2	Upstream	Y	82160752	82161751	2	33.7	50.0	19.6	18.4
chr19	Fbxw4	Upstream	Y	45734684	45735683	3	1.2	26.3	19.0	17.8
chr7	Ceacam9	Exon1	Y	17308974	17309333	2	55.0	70.4	61.9	60.5
chr7	Snrpn	5-UTR	Y	67133491	67285105	6	34.9	50.6	42.0	40.5
chr7	Thrsp	Upstream	Y	104566021	104567020	1	19.8	39.1	42.5	41.0
chr4	Ccnl2	Upstream	Y	155185598	155186597	4	27.5	80.0	21.3	19.8
chr4	Astn2	Upstream	Y	66065518	66066517	3	2.7	22.1	16.7	15.1
chr17	Atp6v1g2	5-UTR	Y	35373541	35373708	2	0.7	16.3	3.8	2.2
chr9	Zmynd10	Exon1	Y	107449641	107449879	2	2.6	28.9	30.7	28.9
chr1	Rps6kc1	Upstream	Y	192735650	192736649	3	1.2	25.0	7.7	5.7
chr2	Eid1	Upstream	Y	125497836	125498835	3	29.3	58.2	52.5	50.3
chr11	Cntd1	5-UTR	Y	101140517	101140619	2	2.7	22.3	22.9	20.5
chr11	Cntd1	Exon1	Y	101140517	101140794	2	2.7	22.3	22.9	20.5
chr18	Slc25a46	Upstream	Y	31769557	31770556	2	24.5	52.6	20.3	17.8
chr15	Dmc1	5-UTR	Y	79432882	79435539	4	24.8	42.3	27.5	24.9
chr1	Irs1	Exon1	Y	82283352	82288014	14	22.5	51.6	40.8	38.0
chr14	4931414P19Rik	5-UTR	Y	55214554	55224745	5	0.3	29.7	12.1	9.1
chr15	Bik	5-UTR	Y	83357292	83371729	4	0.8	38.7	11.6	8.2
chr5	Limch1	Upstream	Y	67136079	67137078	4	50.3	89.7	7.6	3.8
chr9	Dclk3	Upstream	Y	111340585	111341584	4	64.8	79.8	41.6	37.2
chr3	Efna3	Upstream	Y	89126802	89127801	5	9.0	51.9	36.9	32.2
chr3	St6galnac3	Upstream	Y	153388098	153389097	2	0.0	19.5	11.5	6.6
chr4	Mecr	Upstream	Y	131398386	131399385	2	20.1	42.3	23.3	18.2
chr11	Cnot8	Upstream	Y	57916655	57917654	5	38.3	80.4	29.7	24.1
chr7	Gsg1l	Upstream	Y	133225926	133226925	4	25.0	45.5	32.9	27.2
chr7	Rasl2-9	Upstream	Y	5077553	5078552	3	15.4	53.4	18.9	13.2

chr4	Eps15	Upstream	Y	108951880	108952879	2	35.0	54.8	22.1	15.0
chr6	Gpr37	Exon1	Y	25638113	25639980	8	3.2	27.9	14.3	7.2
chr15	Tnrc6b	5-UTR	Y	80541743	80590015	6	7.8	41.3	8.3	1.1
chr12	Ralgapa1	Upstream	Y	56922155	56923154	2	22.6	43.7	20.5	12.5
chr8	Cdh15	Exon1	Y	125372274	125372429	2	10.2	27.3	14.6	6.4
chr7	Tgfb1	Upstream	Y	26471021	26472020	5	14.6	40.5	45.3	37.0
chr15	Zfp641	Upstream	Y	98126515	98127514	3	50.8	68.9	64.1	55.8
chr16	Paxbp1	Upstream	Y	91044625	91045624	5	0.0	15.1	9.4	1.1
chr5	Fras1	Upstream	Y	96801974	96802973	3	0.6	19.5	10.3	1.5
chr14	Atg14	Upstream	Y	48188110	48189109	2	36.6	36.5	0.5	76.5
chr14	Uggt2	Upstream	Y	119498657	119499656	3	73.8	84.6	24.3	83.3
chr8	Tppp3	Upstream	Y	107995323	107996322	2	75.0	85.0	0.2	48.3
chr4	Ptprf	Upstream	Y	117964003	117965002	2	78.5	88.3	42.7	90.8
chr4	Xkr8	Upstream	Y	132288462	132289461	2	93.5	93.4	48.4	94.0
chr8	Map10	Upstream	Y	128192718	128193717	3	91.7	97.1	60.3	98.8
chr19	Ehbp1l1	Exon1	Y	5725917	5726317	2	62.7	64.0	21.4	56.7
chr5	Shh	Upstream	Y	28793642	28794641	1	14.8	17.6	3.5	36.3
chr5	Sirt4	5-UTR	Y	115933121	115934493	3	35.6	27.2	0.4	31.9
chr13	Nsd1	Upstream	Y	55310143	55311142	1	67.8	74.6	53.2	84.3
chr2	Gm13889	Upstream	Y	93797258	93798257	3	50.0	49.4	21.8	51.1
chr11	Fbxo39	5-UTR	Y	72127946	72130325	2	56.0	69.5	33.6	62.7
chr14	Slc7a7	5-UTR	Y	55027842	55036539	3	63.0	74.5	49.6	77.0
chr13	Dbn1	Upstream	Y	55589438	55590437	2	44.6	41.2	21.1	48.3
chr6	Hoxa2	5-UTR	Y	52114644	52114830	1	53.3	53.2	33.9	60.3
chr6	Arl8b	Upstream	Y	108732053	108733052	2	0.4	0.5	0.1	26.4
chr15	Asap1	Upstream	Y	64214482	64215481	2	24.6	31.0	11.7	37.4
chr9	Ube2q2	Upstream	Y	54996176	54997175	4	24.9	33.5	12.9	38.6
chr11	Rasgef1c	5-UTR	Y	49715337	49770541	6	80.8	74.6	39.7	65.1
chr14	Adcy4	Upstream	Y	56402857	56403856	2	37.5	49.1	38.5	64.0
chr8	Bean1	Upstream	Y	106693413	106694412	3	34.4	28.2	32.7	57.0
chr8	B3gnt3	Exon1	Y	74217216	74217798	4	77.5	80.3	52.5	76.1
chr4	Hes5	Upstream	Y	154334032	154335031	5	68.6	61.0	25.6	48.1
chr8	1700067K01Rik	Exon1	Y	86525605	86525804	2	79.8	93.2	71.8	94.3
chr8	Stox2	5-UTR	Y	48288608	48437702	14	56.3	67.7	10.3	32.5
chr9	Ddx43	Upstream	Y	78242584	78243583	3	28.1	33.9	17.5	38.9
chr17	Gm7325	5-UTR	Y	45738621	45739051	1	49.2	49.2	39.3	60.6
chr19	Apba1	Exon1	Y	23967225	23968510	7	76.0	71.4	32.1	53.2
chr15	Fmnl3	Upstream	Y	99200898	99201897	2	45.8	45.7	18.6	39.6
chr13	Nsd1	Exon1	Y	55311143	55311236	1	74.6	88.5	70.9	91.9
chr11	Shroom1	Exon1	Y	53276725	53277695	6	52.0	51.4	6.8	26.8
chr11	Dnaic2	Upstream	Y	114587726	114588725	2	19.0	30.4	11.2	30.7
chr10	Atcay	Exon1	Y	80693237	80693524	2	22.2	28.3	14.9	34.4
chr6	Hoxa2	Exon1	Y	52114265	52114830	3	54.4	54.4	35.3	54.7
chr4	Dnajc11	Upstream	Y	151306829	151307828	4	46.9	42.6	16.7	36.0
chr19	Tex40	Upstream	Y	6999871	7000870	4	18.5	29.5	16.9	36.3
chr17	Pacsin1	Upstream	Y	27791627	27792626	4	63.0	70.0	17.5	36.8
chr15	Zfp641	5-UTR	Y	98124235	98126514	4	0.7	3.0	29.7	48.3
chr12	Sptb	5-UTR	Y	77750912	77811534	9	44.3	53.8	22.6	41.1
chr11	Sec24a	Upstream	Y	51570337	51571336	2	2.7	0.0	2.1	20.4

chr16	Heg1	Upstream	Y	33683552	33684551	2	24.4	34.6	14.3	32.4
chr5	Pds5b	Upstream	Y	151475402	151476401	3	23.4	18.2	0.6	18.5
chr14	Amer2	Upstream	Y	60996123	60997122	3	44.9	40.2	16.0	33.7
chr8	Ccdc110	Exon1	Y	47020372	47020518	1	50.4	53.6	41.3	58.9
chr18	Elac1	Upstream	Y	73914134	73915133	4	76.6	70.5	57.2	73.5
chr3	Gm9833	5-UTR	Y	10088277	10089618	1	36.2	41.2	27.0	43.2
chr15	Nr4a1	Upstream	Y	101096277	101097276	4	29.9	25.9	12.5	28.6
chr8	Bean1	5-UTR	Y	106694413	106705630	7	48.1	59.4	12.7	28.6
chr10	Slc2a12	Upstream	Y	22363817	22364816	1	22.8	36.6	19.5	35.4
chr17	Prss41	Upstream	Y	23981124	23982123	1	11.7	24.9	18.5	34.4
chr5	9530036O11Rik	5-UTR	Y	28793524	28793888	2	8.0	11.3	2.8	18.6
chr4	Msantd3	Upstream	Y	48551818	48552817	2	20.5	28.3	25.9	41.6
chr11	Cuedc1	5-UTR	Y	87912648	87983334	14	24.2	38.6	15.6	31.3
chr4	B930041F14Rik	Upstream	Y	155067451	155068450	3	1.7	0.9	1.0	16.6
chr4	Gm13238	Upstream	Y	145426122	145427121	1	44.0	52.8	36.5	52.0
chr4	Fbxo10	Upstream	Y	45097477	45098476	2	24.7	28.0	12.4	27.7
chr4	Glis1	Upstream	Y	107106324	107107323	4	30.9	37.8	7.6	22.9
chr3	Zfp704	Upstream	Y	9610086	9611085	3	7.3	13.9	2.8	18.0
chr7	Ccdc114	Exon1	Y	53184437	53184626	1	45.0	53.1	44.5	59.8

Group 2-mDMR genes associated with methylation gain at gene body and 3' CGI

							DNA Methylation Level (%)			
							ISCs		Diff. Cells	
**Chr**	**Gene**	**Region**	**CGI**	**Start**	**End**	**#Bins**	**P0**	**P21**	**P0**	**P21**
chr12	Gsc	Downstrea	Y	105708419	105709418	1	18.4	57.1	8.8	62.8
chr11	Trim7	3-UTR	Y	48663492	48663697	1	38.7	69.8	20.8	69.6
chr8	Slc9a5	Intron1	Y	107873473	107877322	2	44.8	76.4	25.8	72.1
chr18	Pcdhga1	3-UTR	Y	37999784	38001524	4	8.3	50.0	15.3	61.5
chr18	Pcdhga3	3-UTR	Y	37999784	38001526	4	8.3	50.0	15.3	61.5
chr18	Pcdhga8	3-UTR	Y	37999784	38001526	4	8.3	50.0	15.3	61.5
chr18	Pcdhgb4	3-UTR	Y	37999784	38001524	4	8.3	50.0	15.3	61.5
chr19	Kank1	Intron4	Y	25453021	25483575	3	4.7	46.2	2.8	48.5
chr15	Baiap2l2	Intron8	Y	79101083	79101631	2	12.9	56.6	12.8	58.2
chr13	Rgs14	Intron7	Y	55481053	55481365	1	25.4	61.7	26.5	69.3
chr18	Pde6a	Exon10	Y	61416638	61416703	1	44.9	69.0	40.3	82.8
chr11	Kdm6b	LastExon	Y	69212020	69213351	4	51.7	81.5	42.5	84.0
chr11	BC096441	Intron12	Y	69508944	69509097	1	11.9	33.3	8.0	47.8
chr2	Hoxd4	Exon4	Y	74564933	74565762	4	18.1	73.0	26.5	66.0
chr11	Kdm6b	3-UTR	Y	69212020	69213327	3	51.7	81.5	43.6	83.1
chr8	Mcf2l	Intron1	Y	12916055	12963163	2	19.6	66.5	19.4	58.8
chr4	Rcc2	Intron2	Y	140258351	140263865	5	4.5	38.8	2.1	40.8
chr8	Jak3	Intron6	Y	74203744	74204333	3	75.3	93.4	35.7	74.1
chr8	Zdhhc1	Downstrea	Y	107995325	107996324	3	58.4	85.0	17.4	55.6
chr10	B4galnt1	Exon7	Y	126607034	126607224	1	32.4	77.3	38.1	75.4
chr18	Pcdhga1	Downstrea	Y	38001525	38002524	1	8.3	50.0	16.3	53.2
chr18	Pcdhga3	Downstrea	Y	38001527	38002526	1	8.3	50.0	16.3	53.2
chr18	Pcdhga8	Downstrea	Y	38001527	38002526	1	8.3	50.0	16.3	53.2
chr18	Pcdhga9	Downstrea	Y	38001518	38002517	1	8.3	50.0	16.3	53.2
chr18	Pcdhgb4	Downstrea	Y	38001525	38002524	1	8.3	50.0	16.3	53.2

chr13	Pde4d		Intron8	Y	110693898	110724075	2	44.0	78.8	44.0	80.4
chr7	Capns1		Exon8	Y	30977886	30977919	1	68.2	87.5	50.2	86.6
chr4	AI464131		LastExon	Y	41442634	41446723	12	24.1	71.3	34.2	70.4
chr11	Nags		Intron5	Y	102009296	102009405	1	46.9	76.8	39.7	75.6
chr7	Capns1		Intron8	Y	30977612	30977885	1	60.9	77.6	50.0	85.9
chr12	Mycn		LastExon	Y	12942899	12944416	4	38.8	82.6	38.4	74.2
chr16		5-Sep	LastExon	Y	18621904	18622889	2	24.8	79.4	33.2	68.6
chr16		5-Sep	3-UTR	Y	18621904	18622832	2	24.8	79.4	33.2	68.6
chr16	Gp1bb		3-UTR	Y	18620412	18620814	2	46.1	95.5	47.5	82.7
chr17	Rab11fip3		Intron14	Y	26173628	26204526	1	33.7	56.0	22.1	56.6
chr11	Shroom1		LastExon	Y	53280265	53281257	3	8.3	24.0	7.2	40.8
chr11	Arhgap27		Exon6	Y	103195572	103195677	1	29.4	64.3	26.5	59.9
chr2	Proser2		LastExon	Y	6019546	6022406	6	51.9	90.2	46.1	79.4
chr7	Phldb3		Intron3	Y	25398033	25401850	1	22.2	72.9	32.5	65.0
chr3	Lrba		Intron40	Y	86346634	86409791	9	24.2	75.8	38.1	70.5
chr5	Card11		LastExon	Y	141348953	141349483	2	16.7	59.7	23.2	55.4
chr16	Scarf2		Intron9	Y	17805018	17806441	2	20.0	46.6	22.7	54.6
chr18	Pcdhga1		LastExon	Y	37999557	38001524	5	49.5	69.1	38.9	70.7
chr18	Pcdhga3		LastExon	Y	37999557	38001526	5	49.5	69.1	38.9	70.7
chr18	Pcdhga8		LastExon	Y	37999557	38001526	5	49.5	69.1	38.9	70.7
chr18	Pcdhgb4		LastExon	Y	37999557	38001524	5	49.5	69.1	38.9	70.7
chr8	Isyna1		Downstrea	Y	73121190	73122189	4	71.8	88.0	43.1	74.8
chr9	Dixdc1		Intron19	Y	50519078	50535890	3	36.3	68.4	35.5	66.8
chr17	Stap2		Intron6	Y	56139439	56139509	1	64.4	91.6	55.2	86.4
chr9	P4htm		Intron7	Y	108486164	108499229	1	26.8	69.1	36.5	67.4
chr7	Pkp3		Intron10	Y	148274824	148275143	1	60.2	85.5	49.1	80.0
chr3	Npr1		Intron21	Y	90267933	90268716	2	53.2	76.6	49.2	80.0
chr2	Sp5		3-UTR	Y	70315227	70315783	2	4.5	44.2	5.5	36.0
chr16	Tiam1		Intron12	Y	89813373	89822122	3	3.6	33.9	7.0	37.2
chr8	Marveld3		LastExon	Y	112471809	112472562	4	32.3	68.2	42.5	72.7
chr2	Bmp2		Intron2	Y	133380505	133386609	2	12.3	40.4	20.0	50.0
chr8	Klf2		LastExon	Y	74844643	74845553	2	46.8	69.7	41.9	71.7
chr11	Trim7		LastExon	Y	48662980	48663697	4	55.1	78.2	52.5	81.5
chr15	Baiap2l2		Exon8	Y	79101632	79101748	1	9.6	32.2	7.6	36.5
chr17	Cldn6		Intron2	Y	23816789	23818014	1	11.5	37.2	15.4	44.3
chr15	Copz1		Downstrea	Y	103130296	103131295	2	35.9	69.8	44.9	73.7
chr15	Baiap2l2		Intron7	Y	79092396	79100935	3	41.4	86.1	19.5	48.3
chr9	Icam5		3-UTR	Y	20843345	20843480	1	29.4	61.7	34.0	62.7
chr11	Plekhh3		Intron3	Y	101025786	101025972	1	53.2	75.0	49.3	77.9
chr11	Rnd2		Intron2	Y	101330335	101331326	1	42.5	80.0	40.2	68.6
chr12	Rapgef5		Intron12	Y	118927156	118927620	2	42.5	69.1	38.8	67.1
chr5	Ift81		Intron18	Y	123061117	123064446	4	25.5	45.3	14.4	42.6
chr17	Prr18		3-UTR	Y	8533872	8536978	8	70.5	86.2	17.3	45.5
chr6	Wipf3		Intron4	Y	54435927	54437901	1	15.5	58.9	13.3	41.5
chr18	Jakmip2		Intron20	Y	43741985	43750201	1	20.0	52.2	43.3	71.4
chr4	Arhgef19		Intron4	Y	140803146	140803292	1	32.4	71.9	40.2	68.3
chr7	Phldb3		Intron2	Y	25397699	25397894	1	29.5	46.5	23.8	51.8
chr10	Cdk19		Intron2	Y	40114207	40154109	2	42.5	75.6	37.0	65.0
chr2	Nr1h3		Exon4	Y	91030619	91030798	1	39.8	62.4	41.0	69.0

chr19	Rps6ka4	Intron6	Y	6905938	6906257	2	50.3	79.4	44.8	72.7
chr12	Amn	Intron3	Y	112510154	112512353	3	42.1	61.8	33.7	61.4
chr11	Gal3st1	LastExon	Y	3897929	3899331	7	56.0	78.3	53.6	81.2
chr10	B4galnt1	Intron8	Y	126607225	126607646	2	16.0	77.3	45.1	72.0
chr13	Pde4d	Exon7	Y	110693785	110693897	2	44.0	78.8	26.4	52.8
chr7	Dmpk	Intron14	Y	19677604	19677891	2	51.9	71.2	47.3	73.7
chr8	Jak3	Exon4	Y	74203101	74203246	1	67.8	87.2	67.6	94.0
chr8	Nfix	Intron8	Y	87251867	87295682	5	7.2	35.3	14.9	41.2
chr19	Pcsk5	Intron1	Y	17508266	17510942	1	37.1	66.7	37.5	63.6
chr19	2700081O15Rik	Intron4	Y	7496953	7497132	1	62.6	87.9	62.8	88.9
chr11	Epn3	Intron1	Y	94352794	94352884	1	31.7	68.3	38.7	64.6
chr1	Nrp2	Intron15	Y	62832876	62859107	3	19.0	40.9	13.6	39.4
chr9	Igdcc4	Intron6	Y	64970614	64971582	3	39.3	84.6	55.2	80.9
chr1	G0s2	LastExon	Y	195098354	195098996	4	7.7	59.3	45.4	70.9
chr17	Stap2	Exon6	Y	56139510	56139579	1	36.0	54.8	28.7	54.1
chr6	Tspan33	Downstrea	Y	29668559	29669558	2	35.2	61.1	45.2	70.1
chr18	Pde6a	Intron10	Y	61414101	61416637	1	23.2	60.0	31.8	56.6
chr2	Lzts3	LastExon	Y	130458575	130461200	4	57.9	76.7	51.4	76.1
chr11	Ccr10	Intron1	Y	101035993	101036713	1	57.1	80.6	50.0	74.6
chr4	Trim14	Downstrea	Y	46518719	46519718	2	14.0	41.2	20.7	45.2
chr19	Efemp2	Intron2	Y	5475179	5475407	1	39.5	66.7	39.5	64.0
chr8	Zfpm1	3-UTR	Y	124861092	124861147	1	55.8	88.1	61.1	85.3
chr18	Ctif	Intron3	Y	75631675	75679392	4	11.9	38.8	34.4	58.2
chr11	Tlcd2	Intron1	Y	75281880	75282058	1	29.4	70.2	33.0	56.8
chr9	Cacna2d2	3-UTR	Y	107429796	107431674	1	49.4	80.8	48.2	71.9
chr10	Phactr2	Intron12	Y	13011769	13193861	11	72.2	93.4	12.8	36.2
chr5	Shh	Intron2	Y	28788124	28793000	16	3.5	44.4	5.0	28.4
chr11	Phospho1	Intron2	Y	95690090	95691864	3	45.4	79.8	26.4	49.4
chr16	Gpr156	Intron1	Y	37916702	37947453	3	13.2	37.6	0.2	23.3
chr8	Fbxl8	LastExon	Y	107791910	107793226	7	72.6	88.5	58.8	81.8
chr2	Spef1	3-UTR	Y	130995997	130997418	2	26.5	51.7	16.8	39.7
chr2	Hoxd3	Intron2	Y	74582613	74584378	2	19.0	60.9	25.0	47.7
chr4	1300002K09Rik	Exon6	Y	45881949	45882077	1	36.5	69.3	43.4	65.9
chr9	Cacna2d2	LastExon	Y	107429652	107431674	2	37.1	65.1	34.8	57.3
chr7	Rcn3	Intron5	Y	52344071	52346810	2	56.0	82.6	65.4	87.9
chr7	Bcl3	Exon2	Y	20394845	20395012	1	59.5	83.1	61.1	83.4
chr15	Hdac7	Exon12	Y	97633114	97633247	1	50.0	81.6	50.5	72.5
chr2	Acvr1c	Intron8	Y	58168435	58210018	7	4.0	30.0	13.2	35.0
chr11	Slc16a11	Exon3	Y	70028786	70029553	4	58.4	81.9	63.1	84.8
chr7	Tmc5	Intron1	Y	125741059	125743697	2	4.5	59.2	23.1	44.9
chr2	Myo3a	Intron13	Y	22255339	22261740	3	14.8	43.3	6.6	28.2
chr14	Opn4	LastExon	Y	35403804	35404516	3	51.3	75.3	53.9	75.5
chr15	Trio	Exon45	Y	27785820	27785989	1	48.6	68.1	46.8	68.4
chr2	Lsm14b	Intron1	Y	179760018	179761339	5	54.2	76.7	19.4	40.6
chr19	Pdzd7	Intron15	Y	45115194	45119907	1	63.3	84.3	64.2	85.1
chr8	Klf2	3-UTR	Y	74844819	74845553	1	46.8	73.0	47.2	68.0
chr17	Ntn3	Exon2	Y	24344338	24344388	1	68.2	84.6	66.7	87.5
chr17	Gm7325	LastExon	Y	45737916	45738634	3	27.4	45.2	18.3	39.0
chr5	Tbx3	LastExon	Y	120132640	120134610	3	36.7	60.3	27.6	48.2

chr6	Dctn1	Intron5	Y	83133951	83136421	3	25.9	43.8	23.6	44.1
chr9	Icam5	LastExon	Y	20843082	20843480	2	29.4	61.7	21.5	41.8
chr17	Tap1	Intron1	Y	34325351	34326122	1	39.2	56.6	36.7	57.0
chr6	Tfpi2	Intron4	Y	3918051	3918236	1	27.1	49.6	34.4	54.6
chr7	Dmpk	Exon13	Y	19677557	19677603	1	42.1	77.5	47.9	67.8
chr4	Ahdc1	Intron2	Y	132568203	132610927	18	58.3	90.6	17.4	37.3
chr13	Irx4	Intron1	Y	73398325	73402295	13	26.4	44.9	18.6	38.3
chr11	Plekhh3	Exon3	Y	101025973	101026105	1	70.5	93.5	71.6	91.2
chr7	B4galnt4	Intron1	Y	148247324	148249735	3	54.8	80.3	27.5	47.1
chr3	Sema4a	LastExon	Y	88239884	88241174	4	38.3	74.3	51.7	71.2
chr10	Abca7	Exon13	Y	79465576	79465797	1	55.9	79.9	61.8	81.3
chr11	Shroom1	Intron1	Y	53270844	53276724	4	40.2	56.4	8.3	27.8
chr11	Tmem88	LastExon	Y	69210018	69211383	3	35.9	56.3	34.9	54.4
chr2	Mir219-2	Downstrea	Y	29700151	29701150	2	53.5	82.0	61.5	81.0
chr14	Slc7a7	Intron11	Y	55035608	55036493	2	63.0	79.9	54.2	73.6
chr7	Dock1	Intron27	Y	142067999	142182204	9	43.0	67.5	22.5	41.8
chr11	Kdm6b	Downstrea	Y	69211020	69212019	5	31.6	56.3	28.6	47.8
chr14	Scara5	Exon3	Y	66349358	66350032	3	40.9	56.8	50.2	69.3
chr4	Ephb2	Intron13	Y	136252070	136326870	4	15.0	79.6	40.9	60.0
chr11	Myo1d	Intron6	Y	80415309	80451375	2	45.1	66.5	45.7	64.8
chr2	Hoxd4	Intron2	Y	74560689	74561388	1	39.3	84.7	55.6	74.6
chr2	Hoxd4	Exon2	Y	74561389	74561424	1	39.3	84.7	55.6	74.6
chr2	Urm1	Downstrea	Y	29700517	29701516	4	53.5	75.9	46.6	65.3
chr11	Tmc8	Intron3	Y	117644858	117645084	1	17.1	39.9	21.2	39.9
chr5	Stx1a	Intron6	Y	135518088	135518462	1	38.1	63.0	29.1	47.6
chr11	Hand1	Intron1	Y	57643209	57644745	2	37.2	61.8	39.5	58.0
chr5	Atraid	LastExon	Y	31356636	31356996	2	4.9	25.1	5.7	24.0
chr5	Atraid	3-UTR	Y	31356741	31356996	2	4.9	25.1	5.7	24.0
chr8	Ssbp4	LastExon	Y	73121389	73121667	2	71.8	88.0	63.8	82.1
chr8	Ssbp4	3-UTR	Y	73121389	73121637	2	71.8	88.0	63.8	82.1
chr11	Lsmd1	Downstrea	Y	69210174	69211173	1	36.5	56.3	34.5	52.7
chr11	Tmem88	3-UTR	Y	69210018	69211113	2	36.5	56.3	34.5	52.7
chr6	Bcl2l14	Downstrea	Y	134388743	134389742	2	58.6	77.0	47.4	65.5
chr16	Gp1bb	LastExon	Y	18620412	18621425	6	32.1	95.5	16.7	34.8
chr11	Socs3	LastExon	Y	117827401	117829632	5	28.7	58.5	26.1	44.0
chr17	Caskin1	Intron18	Y	24642966	24643467	1	62.2	77.5	46.7	64.5
chr10	Nfic	Exon3	Y	80870300	80870424	1	11.3	32.8	16.9	34.5
chr5	Fbrsl1	Intron1	Y	110792899	110799968	1	15.0	33.3	0.0	17.4
chr6	Lpar5	LastExon	Y	125031155	125032490	6	41.0	65.7	35.9	53.2
chr11	Nags	Intron4	Y	102008859	102009123	1	10.6	67.2	35.5	52.8
chr9	Nrgn	Intron2	Y	37353303	37353532	1	23.5	48.8	28.2	45.5
chr2	Hoxd4	Intron3	Y	74561425	74563519	5	48.7	71.6	50.2	67.4
chr2	Fbn1	Intron64	Y	125305009	125331175	2	2.1	30.1	4.1	21.3
chr18	Reep5	Intron3	Y	34516885	34532058	1	35.3	81.8	43.3	60.4
chr17	Kremen2	3-UTR	Y	23878166	23878727	1	61.4	80.6	67.2	84.3
chr15	Vdr	Intron3	Y	97689681	97697547	3	42.4	67.3	65.9	82.9
chr9	Rasl12	Intron1	Y	65246475	65248725	1	12.7	42.3	19.9	36.9
chr6	2700086A05Rik	Intron2	Y	52162487	52163031	3	0.6	31.8	2.7	19.6
chr6	2700086A05Rik	3-UTR	Y	52163032	52163596	3	0.7	36.5	2.5	19.5

chr11	Camta2	Exon2	Y	70484051	70484238	1	36.9	69.1	45.6	62.5
chr7	Igf1r	Intron1	Y	75097839	75148695	9	57.2	75.2	31.6	48.3
chr4	Prdm16	Intron15	Y	153850796	153902690	2	21.0	51.6	25.6	42.2
chr4	Lactbl1	3-UTR	Y	136194000	136194025	1	14.3	37.5	11.5	27.9
chr4	Bmp8b	Intron1	Y	122782919	122791810	2	44.0	75.1	62.2	78.5
chr11	Cntnap1	Exon4	Y	101039550	101039753	1	56.8	74.6	57.1	73.5
chr11	Nos2	Intron2	Y	78735880	78742075	5	3.8	25.0	5.8	22.1
chr6	Magi1	Intron22	Y	93765820	93893197	2	0.0	20.9	0.6	16.7
chr6	Magi1	Intron21	Y	93765820	93863317	2	0.0	20.9	0.6	16.7
chr9	Ppm1m	Intron9	Y	106100556	106101300	4	1.8	58.2	14.8	30.9
chr13	Nrn1	Intron1	Y	36818726	36821975	4	18.9	36.2	21.0	36.9
chr19	Tmem132a	Intron2	Y	10934246	10934613	1	53.3	72.7	66.7	82.6
chr6	AI854703	Intron1	Y	48578569	48579126	1	29.2	50.0	31.9	47.8
chr17	H2-Q8	Exon2	Y	35562516	35562791	1	24.9	53.5	27.4	43.3
chr7	Chst8	LastExon	Y	35459487	35461263	6	11.7	50.0	19.3	35.0
chr7	Bloc1s3	3-UTR	Y	20091153	20092362	1	64.1	87.3	72.7	88.3
chr2	Foxa2	LastExon	Y	147868614	147870560	8	4.6	47.1	12.4	27.8
chr7	1-Sep	Intron1	Y	134358359	134358462	2	27.4	57.6	41.0	56.4
chr12	Atl1	Intron2	Y	71008980	71026928	5	5.3	43.4	20.9	36.2
chr16	Liph	Intron7	Y	21976408	21981408	1	23.1	43.8	29.3	44.7
chr5	Zfp316	Intron6	Y	144026813	144031529	5	28.6	71.2	19.0	34.4
chr11	Cuedc1	Intron1	Y	87912846	87983066	13	21.2	38.6	14.2	29.5
chr9	Barx2	Intron3	Y	31666629	31720489	2	13.7	33.5	14.9	30.2
chr14	Zfhx2	Intron9	Y	55694122	55710844	6	12.3	30.4	15.3	30.4
chr7	Kcnq1	Intron11	Y	150447282	150544614	12	53.3	73.0	52.1	67.2
chr11	Pdlim4	Intron6	Y	53877289	53882341	2	55.1	76.1	39.1	54.1
chr8	Tradd	LastExon	Y	107782475	107783195	4	72.5	89.9	73.9	88.9
chr7	Arhgap33	Exon20	Y	31319122	31319231	2	17.5	33.3	18.4	33.3
chr4	Gm12992	Intron4	Y	131471437	131475682	6	32.9	51.4	35.0	49.7
chr4	Gm12992	Intron2	Y	131471437	131475682	6	32.9	51.4	35.0	49.7
chr17	Npw	Downstrea	Y	24793275	24794274	1	40.5	55.9	35.6	50.2
chr17	Kremen2	LastExon	Y	23878166	23878938	2	61.4	80.6	63.7	78.2
chr5	Ankrd13a	Intron8	Y	115246925	115247861	2	50.5	93.9	45.1	59.5
chr1	Phlda3	Intron1	Y	137663472	137665054	1	15.6	49.4	18.9	33.3
chr1	Phlda3	3-UTR	Y	137663404	137665711	1	15.6	49.4	18.9	33.3
chr2	Gm17762	Downstrea	Y	17952205	17953204	1	11.5	36.2	12.4	26.8
chr17	Tmem151b	Intron2	Y	45684151	45686231	5	35.1	51.1	28.5	42.9
chr7	Myh14	Intron28	Y	51893834	51894312	2	55.6	96.3	64.0	78.3
chr12	Cyp46a1	Intron1	Y	109572775	109580532	3	31.2	50.8	31.6	45.9
chr7	Bcl3	Intron2	Y	20394609	20394844	1	46.5	71.3	47.5	61.8
chr19	Entpd7	LastExon	Y	43804188	43808343	2	55.8	76.1	61.9	76.1
chr17	Ccdc64b	Intron2	Y	23799030	23802354	1	70.9	89.8	73.4	87.6
chr3	Nbea	Intron17	Y	55527572	55590362	13	20.4	55.2	30.6	44.7
chr7	Atp4a	Intron11	Y	31502903	31503997	1	47.1	63.2	37.1	51.1
chr11	Slc16a11	Intron3	Y	70028601	70028785	1	67.5	87.4	70.1	84.1
chr8	B3gnt3	Intron2	Y	74217799	74225616	4	0.9	35.6	16.7	30.6
chr19	Ltbp3	Intron2	Y	5742563	5745439	2	50.0	69.9	46.6	60.5
chr3	4931419H13Rik	Intron1	Y	54859359	54862196	3	16.7	33.9	4.8	18.5
chr11	Sfi1	Intron31	Y	3087414	3088943	2	46.6	88.8	71.4	85.1

chr5	Mnx1	Intron1	Y	29800772	29801295	3	41.3	75.7	58.9	72.4
chr4	Cela2a	Intron3	Y	141375043	141377239	1	58.8	75.7	75.9	89.5
chr3	Gria2	Intron14	Y	80545121	80605566	6	21.3	37.4	16.2	29.7
chr19	Cyp26c1	Intron2	Y	37761152	37761608	2	6.0	54.6	20.4	33.9
chr12	Mboat2	Intron1	Y	25516640	25563055	4	7.6	25.4	4.5	18.0
chr9	Rbp1	Intron1	Y	98325471	98325871	2	31.4	62.6	34.0	47.5
chr6	Plekha5	Intron3	Y	140375077	140473385	2	48.2	67.2	42.0	55.4
chr5	Mnx1	LastExon	Y	29800363	29800771	3	45.3	76.1	51.4	64.7
chr4	Prdm16	Intron16	Y	153903041	154010831	13	30.0	47.5	19.8	33.0
chr2	Rapgef4	Intron3	Y	71872177	71883158	2	25.9	84.8	68.8	81.9
chr2	Hoxd3	Intron1	Y	74550374	74581984	32	27.7	48.4	21.2	34.3
chr5	Sirt4	Exon2	Y	115930240	115930534	1	8.0	25.8	9.2	22.2
chr7	Zfp575	Intron1	Y	25371158	25371672	2	63.4	81.8	66.5	79.3
chr19	Pdzd7	Exon14	Y	45115053	45115193	1	42.5	61.8	50.0	62.8
chr15	Krt77	Downstrea	Y	101689287	101690286	2	7.3	28.7	11.1	23.8
chr7	Mylpf	Downstrea	Y	134357802	134358801	4	16.4	43.0	33.8	46.5
chr5	Mlxipl	Exon13	Y	135609864	135609991	1	0.4	17.9	2.4	15.0
chr1	Aff3	Exon10	Y	38266398	38267492	4	47.0	68.1	54.8	67.4
chr17	Tnfrsf21	Intron1	Y	43154042	43174543	7	49.6	85.0	30.4	42.9
chr19	Rps6ka4	Intron15	Y	6913858	6913949	1	62.8	97.7	75.0	87.5
chr4	Foxo6	3-UTR	Y	119939683	119940521	4	24.7	41.8	29.8	42.3
chr11	Tmc8	Intron9	Y	117648183	117651529	2	40.3	59.1	35.0	47.4
chr6	Rab11fip5	Intron5	Y	85298986	85324091	2	70.8	86.3	34.0	46.3
chr13	Irx4	Intron2	Y	73402428	73402905	2	22.4	62.3	46.9	59.2
chr14	Extl3	Intron4	Y	65685648	65694420	2	49.0	64.5	59.8	72.1
chr4	Espn	Intron6	Y	151503748	151504877	3	17.8	43.3	23.0	35.2
chr9	Barx2	Intron2	Y	31661783	31666315	1	67.4	86.4	74.3	86.3
chr1	Glb1l	Exon14	Y	75205631	75205797	1	6.2	24.6	6.9	18.9
chr10	D630037F22Rik	Intron11	Y	55809646	55843316	6	0.5	15.7	4.0	15.9
chr2	Itpka	Exon2	Y	119575098	119575314	1	61.8	82.1	66.6	78.5
chr11	4933422H20Rik	Exon2	Y	115302395	115303059	2	50.0	78.4	62.8	74.2
chr1	Ihh	Intron1	Y	74993322	74994877	3	22.9	56.0	12.3	23.5
chr15	Galr3	Downstrea	Y	78873989	78874988	1	39.3	74.6	44.1	55.3
chr15	Pde1b	Intron2	Y	103334015	103350379	3	58.0	73.9	36.6	47.9
chr7	Sptbn4	Intron5	Y	28147488	28149089	2	4.3	33.8	36.0	47.2
chr2	Sp5	LastExon	Y	70314081	70315783	8	3.5	34.2	2.0	13.2
chr13	Irx4	Exon4	Y	73404952	73405280	2	24.2	61.6	40.0	51.1
chr19	Ccdc86	Downstrea	Y	11014971	11015970	5	1.3	21.1	9.8	20.8
chr9	Epor	Exon2	Y	21764968	21765055	1	18.1	51.2	27.7	38.7
chr6	Zfp467	LastExon	Y	48386612	48389356	8	70.7	90.6	76.0	87.0
chr6	Zfp467	Intron1	Y	48377879	48391945	9	70.7	90.6	76.0	87.0
chr9	Barx2	Exon2	Y	31666316	31666628	2	13.7	37.3	20.2	31.1
chr7	Bloc1s3	LastExon	Y	20091153	20092959	5	32.4	50.5	18.2	29.1
chr8	Cacna1a	Intron37	Y	87138691	87141690	3	41.4	56.5	44.6	55.3
chr6	Hoxa7	Downstrea	Y	52164623	52165622	2	12.8	34.6	14.9	25.7
chr6	Hoxa7	3-UTR	Y	52165623	52165715	1	12.8	34.6	14.9	25.7
chr8	6430548M08Rik	Intron12	Y	122684313	122685114	2	71.8	88.0	77.9	88.6
chr3	Polr3c	Exon9	Y	96523384	96523488	1	63.2	87.0	70.7	81.2
chr3	Ccdc109b	Intron7	Y	129637619	129672867	5	6.7	64.5	37.9	48.3

chr5	Drc1	Intron3	Y	30644973	30647772	1	6.0	25.1	3.2	13.5
chr11	Fam171a2	Intron1	Y	102300224	102300745	1	53.3	73.0	49.3	59.7
chr2	St6galnac6	Intron1	Y	32455357	32467743	11	11.8	37.5	10.5	20.8
chr6	Lrrc23	LastExon	Y	124719881	124720181	2	26.2	50.0	18.6	28.7
chr6	Lrrc23	3-UTR	Y	124719881	124720619	2	26.2	50.0	18.6	28.7
chr9	Igdcc3	Intron2	Y	64992369	65025201	6	62.3	83.1	20.6	30.8
chr4	Agrn	Intron34	Y	155559245	155569352	2	53.3	70.8	60.8	70.7
chr17	Haao	Intron9	Y	84242615	84245976	1	74.3	90.1	79.7	89.6
chr11	Abi3	LastExon	Y	95691386	95694190	8	45.4	79.8	17.0	26.7
chr11	Abi3	3-UTR	Y	95691386	95694026	8	45.4	79.8	17.0	26.7
chr7	Dgat2	Intron7	Y	106318337	106330901	2	3.8	24.3	15.2	24.9
chr6	Zfp467	3-UTR	Y	48386612	48389282	7	70.7	90.6	76.4	85.9
chr15	Apobec3	Intron1	Y	79723000	79725840	2	11.5	45.1	16.5	26.0
chr14	Zmiz1	Intron6	Y	26401160	26455358	11	13.8	28.9	4.7	14.3
chr12	Hif1a	Intron1	Y	75009312	75027543	4	3.9	19.5	0.8	10.3
chr11	Phf23	Exon2	Y	69811248	69811340	1	43.8	62.6	45.1	54.5
chr18	Gm9926	3-UTR	Y	66666928	66671391	2	25.4	49.7	39.9	49.3
chr3	Gm21949	Intron5	Y	68299180	68400071	7	11.1	71.4	13.8	23.2
chr3	Schip1	Intron2	Y	68299180	68400071	7	11.1	71.4	13.8	23.2
chr15	Cacnb3	Intron1	Y	98462952	98469918	12	4.5	22.0	11.2	20.5
chr19	Entpd7	3-UTR	Y	43804426	43808343	1	51.8	73.8	64.0	73.1
chr11	E130012A19Rik	3-UTR	Y	97488701	97489485	2	10.3	26.4	12.9	21.9
chr1	Psen2	Intron10	Y	182175889	182186353	5	28.1	44.2	19.2	27.9
chr9	Phldb1	Intron21	Y	44536184	44543207	9	21.0	44.4	5.3	13.9
chr10	Mmp11	Intron7	Y	75391255	75395078	2	5.2	29.6	6.4	15.0
chr7	Fam53b	Intron4	Y	139971069	140004748	6	14.3	65.2	39.2	47.8
chr17	Arhgdig	Exon4	Y	26337136	26337315	1	76.5	91.7	80.8	89.3
chr11	Myo1d	Intron1	Y	80297885	80370935	1	75.9	92.6	68.9	77.4
chr11	Ebf1	Intron6	Y	44456972	44682593	4	45.0	60.4	38.9	47.4
chr10	Foxo3	Intron2	Y	41917708	41994621	3	1.7	16.8	7.4	15.8
chr7	Ryr1	Intron57	Y	29855716	29856587	2	30.6	53.5	31.2	39.6
chr8	Ctxn1	3-UTR	Y	4257646	4258380	2	19.3	66.1	17.2	25.6
chr16	Tiam1	Intron27	Y	89956353	89974828	6	14.8	45.8	15.7	24.0
chr5	Setd1b	3-UTR	Y	123616016	123618639	2	71.2	88.6	79.8	88.0
chr9	Mon1a	Intron3	Y	107802847	107803525	1	30.3	54.5	45.4	53.5
chr7	Bnip3	Intron5	Y	146090512	146101045	2	0.0	26.4	1.9	10.0
chr7	Prkcdbp	Intron1	Y	112629734	112630289	2	13.0	34.7	8.9	17.0
chr4	Runx3	Intron5	Y	134727174	134731214	1	35.6	67.9	49.5	57.4
chr11	Hcrt	Intron1	Y	100623480	100624134	1	6.7	36.5	11.6	19.4
chr4	Ptch2	Intron1	Y	116769033	116769451	1	0.0	16.2	4.7	12.5
chr10	Hmga2	Intron2	Y	119811757	119899719	6	21.6	64.8	45.8	53.6
chr3	Lmna	Intron7	Y	88289070	88290125	1	57.1	73.3	67.3	75.0
chr1	Obsl1	Intron5	Y	75484638	75485163	2	3.2	70.0	30.7	38.3
chr4	Nkain1	Intron1	Y	130125913	130160561	5	0.0	16.9	7.7	15.3
chr11	Myh3	Intron7	Y	66897361	66897576	1	48.5	64.6	55.0	62.5
chr3	Ankrd35	Exon9	Y	96487106	96489097	3	14.4	35.3	17.8	25.4
chr16	Liph	Intron6	Y	21974221	21976305	1	51.1	70.8	52.1	59.5
chr11	Gfap	Intron5	Y	102754901	102755691	2	58.5	96.2	52.8	60.1
chr8	Pard6a	Intron1	Y	108225218	108226073	5	2.5	19.9	5.8	13.1

chr5	Tmub1	LastExon	Y	23951281	23952091	4	42.2	57.8	52.3	59.6
chr12	Rtn1	Intron6	Y	73324527	73404643	8	0.5	22.3	23.2	30.4
chr16	Sdf2l1	Intron1	Y	17130882	17131689	1	3.4	18.8	3.7	10.9
chr8	Klhl36	Exon2	Y	122393522	122394592	5	62.1	86.5	79.6	86.7
chr4	Lactbl1	Downstrea	Y	136194026	136195025	2	40.4	59.5	22.6	29.7
chr13	Cd83	Intron2	Y	43880837	43892901	2	37.0	56.6	40.6	47.6
chr7	Sergef	Intron1	Y	53698906	53771037	2	69.8	87.8	79.0	86.1
chr7	Lrrc4b	LastExon	Y	51716379	51718714	10	1.3	39.4	31.3	38.3
chr11	4933439C10Rik	Intron1	Y	59320389	59320692	1	16.8	32.8	10.9	17.9
chr9	Klhl31	Intron2	Y	77498983	77502933	2	36.4	60.5	22.4	29.3
chr14	Slain1	Intron5	Y	104094541	104101733	1	78.4	97.8	84.3	91.0
chr15	Syngr1	Intron1	Y	79921923	79940873	6	38.9	54.9	34.7	41.5
chr7	Rgma	Intron1	Y	80520775	80536266	8	8.8	26.3	5.2	11.9
chr10	H2afy2	Intron8	Y	61220703	61246530	6	4.5	32.4	2.6	9.1
chr9	Rassf1	Intron1	Y	107454178	107456468	4	4.6	27.8	13.5	20.1
chr7	Wnt11	Intron1	Y	105987701	105994883	4	12.5	27.9	32.1	38.7
chr7	Art1	Exon2	Y	109255181	109255946	3	42.4	67.8	24.5	31.0
chr17	H2-Q7	Exon2	Y	35576854	35577129	1	42.7	61.5	50.5	57.0
chr17	H2-Q9	Exon2	Y	35576854	35577129	1	42.7	61.5	50.5	57.0
chr3	Rhoc	Intron1	Y	104592002	104594806	4	26.1	48.2	11.9	18.3
chr9	Eomes	Intron1	Y	118388665	118389584	2	8.3	23.7	8.4	14.9
chr6	Slc41a3	Intron1	Y	90555003	90561872	3	8.6	24.3	7.8	14.2
chr4	Draxin	LastExon	Y	147472546	147476691	3	7.1	24.3	2.3	8.7
chr17	Map4k3	Intron33	Y	81080266	81127133	5	9.4	52.4	16.5	22.8
chr17	Tulp1	LastExon	Y	28488464	28488947	4	0.0	24.3	13.2	19.5
chr7	Mir291a	Downstrea	Y	3219002	3220001	4	17.5	34.1	19.9	26.1
chr2	Smox	Intron1	Y	131317773	131337719	5	18.2	41.5	4.3	10.6
chr3	Tnik	Exon11	Y	28476291	28476495	1	27.4	61.5	38.9	45.1
chr8	Zfpm1	LastExon	Y	124859332	124861147	10	55.8	85.8	34.2	40.4
chr11	Prkca	Intron12	Y	107875696	107915288	3	18.8	44.6	36.2	42.4
chr7	Lmo1	Intron3	Y	116287184	116313496	4	8.7	38.1	13.4	19.3
chr2	Rbpjl	LastExon	Y	164239870	164240948	2	58.1	92.2	55.4	61.3
chr15	Csnk1e	Intron9	Y	79260596	79269201	2	49.8	66.8	50.8	56.7
chr10	Csrp2	Intron1	Y	110357311	110369010	4	10.4	34.1	9.7	15.5
chr10	Abca7	Intron14	Y	79465798	79465876	1	45.2	64.8	66.2	72.0
chr7	Cd37	Intron3	Y	52491014	52491484	1	20.3	43.2	26.4	32.1
chr1	Tmem198	Exon2	Y	75479144	75479719	3	48.0	68.2	45.8	51.4
chr7	Gm10046	Intron1	Y	28550803	28554258	2	22.5	56.7	46.2	51.8
chr16	Ece2	Exon2	Y	20630425	20630511	1	13.1	28.2	17.2	22.6
chr14	Rem2	Exon3	Y	55097791	55097998	1	65.3	87.0	85.0	90.4
chr10	Gm10754	Intron1	Y	97145293	97215139	7	75.8	91.7	83.1	88.5
chr11	Hoxb2	3-UTR	Y	96214944	96215328	1	27.5	49.1	49.4	54.7
chr9	Epor	Intron2	Y	21764293	21764967	1	26.7	62.5	46.6	51.9
chr12	Bcl11b	Intron2	Y	109204098	109227674	7	1.6	40.5	29.3	34.5
chr14	Abcc4	Intron28	Y	119053597	119067568	2	49.0	73.7	64.2	69.3
chr5	Rilpl1	Intron6	Y	124971411	124980683	3	36.4	89.3	37.4	42.4
chr8	Nfix	Intron9	Y	87296215	87323896	19	19.2	50.6	6.8	11.8
chr7	Trpm1	LastExon	Y	71412846	71414645	5	34.3	77.2	31.4	36.4
chr11	Rai1	Intron2	Y	59953873	59998597	16	39.5	55.0	19.9	24.9

chr2	Prdm11	Intron6	Y	92854047	92886135	14	0.0	37.9	12.3	17.3
chr5	Card11	Intron13	Y	141366024	141366939	2	66.9	84.1	71.4	76.1
chr11	Col23a1	Intron1	Y	51103890	51130397	3	5.2	24.5	6.4	11.1
chr9	Mras	Intron5	Y	99312022	99336728	3	69.0	86.6	27.0	31.6
chr3	Gipc2	Downstrea	Y	151755805	151756804	1	51.9	70.0	58.5	63.1
chr8	Kbtbd11	LastExon	Y	15026651	15033332	12	20.0	59.1	7.7	12.3
chr7	Kcnj11	Intron1	Y	53355023	53355997	4	40.9	61.9	31.1	35.7
chr2	Gata3	Intron4	Y	9796553	9798978	3	16.1	40.4	19.5	24.0
chr5	Mnx1	3-UTR	Y	29800363	29800408	1	57.4	83.3	60.7	65.2
chr4	Epha10	Intron8	Y	124583277	124589250	4	3.0	32.5	24.0	28.5
chr17	Cpne5	Intron20	Y	29363179	29374557	2	23.5	52.6	17.6	22.0
chr7	Sprn	LastExon	Y	147336527	147339571	4	6.3	30.9	19.5	23.8
chr19	Peli3	Intron6	Y	4941913	4942937	4	50.0	71.4	14.1	18.5
chr1	Neurl3	Intron2	Y	36325555	36326057	3	72.2	94.9	80.7	84.7
chr2	Necab3	Intron12	Y	154382818	154384473	2	9.8	29.3	14.6	18.6
chr6	Prrt3	LastExon	Y	113444089	113446059	7	17.4	34.5	16.4	20.5
chr3	1110032F04Rik	3-UTR	Y	68674374	68676085	3	2.1	18.9	16.3	20.3
chr17	Cbs	Intron16	Y	31770100	31774027	5	12.8	37.1	3.4	7.3
chr19	Add3	Intron1	Y	53217580	53291290	6	23.3	43.1	14.4	18.3
chr12	Rps6kl1	Intron10	Y	86490859	86492112	3	13.8	29.9	6.9	10.7
chr11	Kcnip1	Intron7	Y	33545628	33892748	11	2.2	30.7	28.2	32.0
chr7	Mir292	Downstrea	Y	3219272	3220271	2	23.8	38.9	26.4	30.2
chr5	Sgsm1	Intron23	Y	113717920	113739567	8	8.1	46.4	35.8	39.6
chr5	Sgsm1	Intron16	Y	113717920	113739567	8	8.1	46.4	35.8	39.6
chr7	Zfp94	Intron5	Y	25099431	25101537	3	19.7	40.3	18.4	22.2
chr1	Syt2	Intron2	Y	136560576	136637411	8	1.1	31.0	26.0	29.8
chr3	Celsr2	Intron33	Y	108210316	108215102	1	72.7	89.5	73.2	76.9
chr1	Slc45a3	Intron1	Y	133859630	133873294	6	23.3	61.7	8.0	11.8
chr9	Zfp872	Intron1	Y	21992676	22001510	2	9.6	30.8	11.8	15.5
chr5	Pdx1	Intron1	Y	148082224	148085955	2	10.5	26.2	23.1	26.8
chr5	Kcnk3	LastExon	Y	30924264	30927643	6	15.8	32.5	12.3	15.8
chr4	Ror1	Intron1	Y	99768915	99975484	3	5.2	20.7	2.6	6.1
chr1	Pam	Intron24	Y	99872483	99873433	1	3.8	25.5	2.5	6.0
chr7	B4galnt4	Intron16	Y	148256841	148256940	1	29.9	49.3	24.4	27.9
chr7	Tbc1d17	Downstrea	Y	52095146	52096145	4	1.6	63.7	22.7	26.1
chr1	Kcnj10	LastExon	Y	174299052	174304216	6	40.7	66.5	61.2	64.6
chr7	Tfpt	Intron5	Y	3580689	3581153	2	2.3	24.6	24.5	27.8
chr6	Hoxa7	LastExon	Y	52165623	52166029	3	11.2	28.2	12.9	16.2
chr11	Rasl10a	LastExon	Y	4959801	4960386	2	11.0	31.5	23.0	26.3
chr5	Stag3	Intron1	Y	138721883	138723092	2	56.6	88.0	61.1	64.3
chr19	Sgms1	Intron5	Y	32234854	32297998	3	1.2	22.4	2.4	5.6
chr7	Taf10	Intron3	Y	112892007	112892343	3	7.4	29.8	20.2	23.4
chr7	Ilk	Downstrea	Y	112891440	112892439	3	7.4	29.8	20.2	23.4
chr11	Mmp28	Intron7	Y	83265242	83276249	2	3.2	59.0	19.1	22.2
chr15	Trps1	Intron5	Y	50678489	50721149	9	0.1	18.8	6.1	9.2
chr8	Ctxn1	LastExon	Y	4257646	4258647	4	19.3	66.1	6.0	9.1
chr3	Rarres1	Intron5	Y	67299734	67319082	3	27.9	49.4	30.3	33.3
chr7	Fbl	Intron8	Y	28964063	28964149	1	31.8	51.3	52.1	55.0
chr7	Fbl	Exon7	Y	28963917	28964062	1	31.8	51.3	52.1	55.0

chr4	Epb4.1	Intron20	Y	131563354	131626542	8	14.1	33.7	7.0	10.0
chr4	A3galt2	LastExon	Y	128444229	128446542	4	64.3	81.7	81.8	84.6
chr19	Men1	LastExon	Y	6339687	6340894	5	10.4	28.8	30.8	33.6
chr7	Il4i1	LastExon	Y	52094953	52096179	5	1.6	63.7	18.3	21.0
chr7	Nup62-il4i1	LastExon	Y	52094953	52096173	5	1.6	63.7	18.3	21.0
chr4	Espn	Intron8	Y	151505404	151507032	6	3.6	20.2	6.1	8.8
chr9	2600006L11Rik	Intron1	Y	63928644	63934090	4	13.9	29.0	19.0	21.7
chr7	Rasip1	Intron5	Y	52888260	52890186	1	74.3	90.6	86.9	89.6
chr19	Neurl1a	Exon2	Y	47314840	47315161	2	41.3	58.2	45.7	48.3
chr8	Lonrf1	Intron11	Y	37299162	37311620	5	3.7	25.4	1.4	4.0
chr4	Fam110b	LastExon	Y	5725416	5727091	5	12.4	59.4	46.1	48.7
chr19	Syt7	Intron3	Y	10492533	10500788	7	10.8	28.5	23.0	25.6
chr4	Nmnat1	Intron4	Y	148848705	148859217	5	32.8	70.1	58.5	61.1
chr8	4933416M07Rik	Intron1	Y	28253834	28253950	2	25.8	85.9	89.0	91.4
chr6	Hoxa9	LastExon	Y	52173096	52174481	2	3.6	21.6	12.9	15.3
chr9	Twf2	Intron1	Y	106105616	106109219	2	0.3	27.6	11.3	13.7
chr7	Chst8	Intron3	Y	35533297	35597364	8	0.6	76.0	33.8	36.2
chr14	Xkr6	Intron1	Y	64226122	64417043	8	43.0	62.6	28.4	30.7
chr7	Mir291b	Downstrea	Y	3219562	3220561	1	19.3	36.9	19.0	21.2
chr7	4732471J01Rik	Intron2	Y	26181409	26181708	2	29.6	57.2	60.0	62.2
chr8	Evi5l	Intron9	Y	4193218	4193305	1	17.2	32.5	24.9	27.1
chr17	Ddah2	Intron3	Y	35197648	35197777	1	61.6	83.0	76.6	78.7
chr7	Rhcg	Intron10	Y	86752618	86762225	2	7.4	24.5	13.5	15.5
chr15	Pdxp	Intron1	Y	78744962	78748559	2	16.7	32.8	11.2	13.1
chr1	Lemd1	Intron1	Y	134088067	134124760	4	1.0	18.6	11.1	13.0
chr9	Slc36a4	Intron1	Y	15514282	15524076	4	17.9	34.4	11.6	13.4
chr7	Grik5	Downstrea	Y	25793870	25794869	2	6.7	30.1	6.1	7.9
chr3	Tnik	Intron12	Y	28476496	28492943	1	41.0	60.0	57.0	58.7
chr7	Sptbn4	Intron18	Y	28179324	28182969	6	10.5	38.0	18.3	20.0
chr10	Cdh23	Intron68	Y	60149852	60159047	7	6.8	40.3	19.0	20.8
chr9	Il10ra	Intron1	Y	45064525	45068435	4	46.7	71.7	73.1	74.8
chr15	Cela1	Exon5	Y	100515565	100515665	1	34.0	51.2	44.9	46.6
chr5	Pxn	Intron1	Y	115956878	115994351	8	54.5	84.3	51.1	52.8
chr3	Phf17	Intron1	Y	41359742	41385116	19	21.6	38.9	13.0	14.6
chr7	Ccdc155	Intron19	Y	52455756	52459961	2	1.6	23.8	24.9	26.3
chr7	Klf13	Intron1	Y	71036684	71082852	12	0.7	17.5	5.5	7.0
chr15	Wnt7b	Intron3	Y	85389560	85411809	21	3.1	33.6	7.6	9.0
chr9	Sema3b	LastExon	Y	107500446	107501506	3	63.3	88.1	79.3	80.7
chr7	Ric8	Exon2	Y	148043881	148044471	3	9.3	37.0	31.9	33.2
chr5	Srrm4	Intron12	Y	116932546	117041342	7	66.1	95.7	49.3	50.5
chr9	Bmper	Intron2	Y	23029288	23058815	1	10.9	28.0	26.6	27.8
chr12	Hs1bp3	Intron1	Y	8320304	8324590	3	1.2	16.2	9.6	10.7
chr9	Plekho2	Intron5	Y	65420937	65427744	3	9.4	35.8	20.4	21.6
chr15	Hdac10	Intron1	Y	88953910	88953994	2	2.5	34.5	17.9	19.0
chr6	Iqsec3	Exon10	Y	121362526	121363634	6	0.4	19.3	6.1	7.2
chr7	B4galnt4	Exon16	Y	148256941	148257013	1	46.3	74.7	61.2	62.3
chr3	Atp8b2	Intron26	Y	89762972	89764996	4	3.6	27.3	6.3	7.4
chr6	Sema4f	Intron13	Y	82887207	82889454	2	2.9	44.3	36.3	37.3
chr4	Astn2	Intron7	Y	65242840	65305731	5	0.9	24.0	37.7	38.8

chr11	Gas2l1	Intron1	Y	4956693	4962157	16	21.5	46.5	18.2	19.2
chr11	Map3k14	Intron15	Y	103103704	103128651	6	41.8	60.3	42.1	42.9
chr9	Rab6b	Intron1	Y	103014557	103042711	6	0.4	19.6	27.8	28.7
chr13	Prr7	Intron1	Y	55565776	55573148	10	3.5	18.9	10.6	11.4
chr16	Snn	Intron1	Y	11066541	11072324	5	12.8	30.8	0.6	1.3
chr7	4930583K01Rik	Intron1	Y	125387480	125388241	3	0.0	16.7	0.2	0.8
chr16	Tmem44	Intron2	Y	30529657	30537539	2	11.6	27.7	13.3	14.0
chr5	Art3	Exon3	Y	92821495	92822203	4	27.6	43.4	32.2	32.8
chr13	Dusp22	Intron1	Y	30752016	30760673	3	8.3	26.2	1.2	1.7
chr8	Rasa3	Intron23	Y	13631893	13677392	7	30.2	45.8	30.4	30.9
chr7	Abcc8	Intron38	Y	53433954	53435171	3	0.8	29.7	8.9	9.4
chr5	Gm9899	Intron3	Y	30885314	30890743	6	0.0	31.0	2.3	2.8
chr5	Ccdc60	Intron6	Y	116590860	116596130	2	45.7	61.1	65.8	66.3
chr11	Mmgt2	Intron1	Y	62462359	62478171	4	0.0	29.8	21.9	22.3
chr11	Gcgr	Intron1	Y	120392148	120395993	3	9.4	26.5	4.8	5.2
chr8	Jph3	Intron1	Y	124254905	124276866	6	1.4	28.1	18.4	18.7
chr5	Cxcl1	Intron2	Y	91320620	91320734	1	34.8	56.5	44.4	44.7
chr9	Fam212a	Intron1	Y	107887396	107888027	3	5.5	23.6	4.7	4.9
chr7	Crtc3	Intron14	Y	87822331	87833492	5	1.0	64.5	10.5	10.7
chr11	Tnfrsf13b	Downstrea	Y	60961145	60962144	2	20.3	37.9	45.1	45.3
chr9	S1pr5	LastExon	Y	21047361	21049647	8	1.2	19.5	1.8	2.0
chr8	Smpd3	Intron8	Y	108812120	108861711	8	5.0	22.6	17.7	17.8
chr5	Tbc1d1	Intron1	Y	64551745	64564626	4	0.3	44.4	22.9	23.0
chr8	Ddx19b	Intron11	Y	113547915	113555521	6	45.1	93.9	18.8	18.8
chr15	Kcns2	LastExon	Y	34768205	34773162	3	41.2	58.6	28.0	28.1
chr11	Ankrd13b	Intron15	Y	77291281	77302940	3	19.9	37.0	15.4	15.4
chr1	Gm216	LastExon	Y	74414967	74415895	4	58.9	76.9	58.0	58.0
chr1	2010300C02Rik	Intron2	Y	37670327	37671325	2	8.6	39.3	25.6	25.5
chr5	Mospd3	LastExon	Y	138037875	138038281	3	14.3	34.2	17.0	16.9
chr5	Mospd3	3-UTR	Y	138037875	138038249	2	14.3	34.2	17.0	16.9
chr4	Atg4c	Intron1	Y	98861144	98879267	4	60.9	93.0	63.2	63.1
chr19	Wdr96	Intron10	Y	47827563	47830491	2	57.2	77.3	70.0	69.9
chr11	E130309D14Rik	Intron3	Y	74448791	74451459	3	39.3	62.5	49.4	49.3
chr4	Ttpa	Intron1	Y	19935791	19941777	3	7.6	29.8	15.0	14.7
chr1	5730559C18Rik	Intron1	Y	138110996	138112332	1	38.5	55.8	58.9	58.6
chr19	Rbp4	Intron2	Y	38192975	38198518	4	62.5	92.2	78.9	78.6
chr10	Tbc1d30	Intron5	Y	120726042	120731644	1	62.5	85.0	83.3	83.0
chr15	Zfp251	LastExon	Y	76682574	76685044	3	69.3	85.6	90.0	89.7
chr1	Epb4.1l5	Exon23	Y	121539054	121539235	2	62.3	78.3	65.7	65.3
chr16	Col8a1	Intron3	Y	57678569	57754752	3	0.0	47.6	62.9	62.4
chr4	Gabrd	Intron8	Y	154763156	154772003	3	0.2	53.6	37.9	37.4
chr5	Rph3a	Intron20	Y	121436523	121458992	2	31.1	56.5	38.1	37.6
chr16	Vwa5b2	Intron6	Y	20591624	20594252	3	15.2	42.6	26.9	26.4
chr19	Gpr137	Intron6	Y	7014973	7016413	7	11.4	34.4	4.5	3.9
chr8	Zdhhc2	Intron1	Y	41509524	41531131	5	22.7	77.3	32.1	31.4
chr14	5031414D18Rik	Intron1	Y	75415974	75431678	4	17.4	34.1	7.9	7.2
chr15	Maff	LastExon	Y	79187851	79189506	6	44.4	68.5	22.5	21.9
chr6	Wdr54	Exon8	Y	83105643	83105865	2	19.7	41.6	23.9	23.1
chr19	Unc93b1	Intron7	Y	3942819	3943528	4	10.0	39.6	24.1	23.2

chr14	Gm16677	Intron2	Y	69950660	69954998	2	5.5	40.0	3.8	2.8
chr9	Kank2	Intron3	Y	21574571	21577188	1	69.9	86.2	82.0	81.0
chr15	Maff	3-UTR	Y	79188286	79189506	4	44.4	68.5	33.7	32.7
chr5	Agap3	Intron1	Y	23958353	23982195	13	18.6	43.1	6.8	5.7
chr18	Tnfaip8	Intron1	Y	50213274	50249853	4	49.5	65.4	51.1	50.1
chr16	Tango2	Intron8	Y	18324544	18343987	4	0.0	32.1	17.1	16.0
chr17	Sox8	LastExon	Y	25702838	25705026	5	46.9	64.8	48.4	47.1
chr11	Rab11fip4	Intron3	Y	79434058	79494289	9	1.0	29.9	11.1	9.8
chr17	Pi16	Intron1	Y	29456337	29459130	1	20.0	36.6	28.2	26.8
chr7	Snrpn	Intron9	Y	67144300	67277135	6	34.9	50.6	42.0	40.5
chr7	Syt3	Intron9	Y	51651406	51654455	1	58.0	73.6	76.5	75.0
chr7	B4galnt4	Intron14	Y	148254661	148256309	3	0.0	46.5	30.9	29.3
chr11	Kcnab3	Intron1	Y	69140426	69141621	1	10.6	27.3	16.9	15.3
chr17	Dlgap1	Intron8	Y	71115430	71136127	1	55.1	72.8	67.9	66.3
chr15	Scn8a	Intron11	Y	100805148	100814119	2	10.1	27.5	12.4	10.5
chr3	Gpr88	3-UTR	Y	115952572	115954423	2	0.0	17.8	14.1	12.2
chr5	Katnal1	Intron2	Y	149690561	149700454	3	23.5	41.9	32.9	30.9
chr11	Myh3	Exon7	Y	66897577	66897669	1	9.9	25.9	26.4	24.4
chr7	Gsg1l	Intron6	Y	133164186	133225373	5	8.6	37.2	45.5	43.1
chr10	Derl3	Exon2	Y	75356515	75356588	1	25.8	43.4	46.4	44.0
chr13	Mcidas	Intron3	Y	113784657	113787050	2	6.9	27.5	6.9	4.2
chr3	Frem2	Intron8	Y	53329874	53339086	1	21.0	38.0	23.6	20.8
chr18	Rnf165	Intron7	Y	77723204	77803780	8	9.2	48.6	38.6	35.7
chr17	Plcl2	Intron5	Y	50807434	50827036	2	69.2	87.3	79.2	76.2
chr11	Abcc3	Intron5	Y	94213541	94216326	1	39.1	63.5	60.3	57.3
chr11	Rufy1	Exon16	Y	50235132	50235305	1	27.3	51.7	32.0	28.9
chr2	Gata3	Exon3	Y	9796016	9796552	3	26.5	45.6	38.8	35.5
chr15	Arhgap39	Intron1	Y	76555607	76555752	1	48.1	63.5	54.1	50.7
chr15	Bik	Intron1	Y	83357367	83371724	4	0.8	38.7	11.6	8.2
chr10	Hkdc1	Intron11	Y	61866396	61868172	2	55.7	79.1	71.5	68.0
chr14	4931414P19Rik	Intron6	Y	55214789	55224639	4	0.3	29.7	15.1	11.4
chr17	Nfkbil1	Intron1	Y	35357922	35358081	1	77.5	96.0	98.9	95.0
chr9	Stac	Intron10	Y	111537590	111592501	2	10.0	39.5	19.8	15.9
chr12	Asb2	Intron3	Y	104563825	104568554	2	3.5	28.6	26.7	22.7
chr11	Gltpd2	Downstrea	Y	70334239	70335238	1	70.0	86.4	74.1	69.6
chr9	Pcbp4	Downstrea	Y	106366343	106367342	3	24.3	67.9	18.8	14.2
chr17	Pisd-ps2	Intron7	Y	3081035	3083967	3	12.0	35.1	35.9	31.3
chr1	Sema4c	Intron8	Y	36609976	36610068	1	72.4	100.0	92.7	87.9
chr7	B9d2	LastExon	Y	26471000	26471577	3	25.4	57.9	59.3	54.3
chr15	Sult4a1	Intron6	Y	83922851	83935860	4	10.4	44.1	49.1	44.0
chr7	Numbl	Intron1	Y	28043981	28047186	3	4.7	30.0	12.0	6.9
chr9	Pml	Exon7	Y	58094782	58095245	2	20.0	41.0	28.5	23.0
chr17	Pglyrp2	Intron1	Y	32550420	32553822	4	13.1	49.4	33.7	28.1
chr15	Grasp	Intron7	Y	101061483	101061947	2	55.8	79.5	73.9	68.2
chr11	Coro6	LastExon	Y	77282881	77283003	2	55.2	77.5	75.2	69.0
chr10	Gna15	Exon4	Y	80975156	80975310	1	17.1	33.4	30.6	24.2
chr15	Aqp5	Intron1	Y	99422066	99423092	1	44.7	62.5	55.6	48.9
chr5	BC037034	Intron9	Y	138704205	138704294	2	8.5	37.7	38.7	32.0
chr15	Tnrc6b	Intron1	Y	80541919	80577835	6	7.8	41.3	8.3	1.1

chr7	Slc8a2	Intron3	Y	16726518	16730279	2	66.7	89.9	74.1	66.4
chr11	Aatk	Intron7	Y	119875049	119876772	3	56.0	75.6	55.4	47.6
chr4	Tnfrsf25	Intron6	Y	151493453	151493619	1	30.8	63.6	52.4	44.4
chr7	Lrfn1	Intron4	Y	29245083	29251607	2	54.3	75.5	82.3	74.4
chr15	Itgb7	Intron11	Y	102054004	102054732	1	29.8	51.2	48.2	40.0
chr17	Tmem63b	Intron6	Y	45800619	45801692	1	51.7	67.2	62.5	54.2
chr14	Bmp1	Intron18	Y	70908722	70911387	1	50.0	73.1	63.0	54.5
chr9	Gpr62	3-UTR	Y	106366291	106366981	2	24.3	67.9	36.0	27.0
chr11	1700051A21Rik	Intron1	Y	72081028	72081382	2	17.5	37.0	24.2	15.1
chr9	Rbpms2	Intron1	Y	65478705	65497155	4	55.1	65.5	19.1	65.3
chr11	Epn3	LastExon	Y	94350913	94352793	4	66.7	78.6	30.6	71.1
chr5	Gm16065	Intron1	Y	120136064	120138232	5	55.0	52.4	31.2	71.0
chr4	Ttc34	Intron1	Y	154230553	154232069	2	2.3	6.2	0.4	38.3
chr10	Adamtsl5	Intron1	Y	79804202	79804333	1	60.0	70.5	46.6	84.0
chr8	Hsf4	Intron4	Y	107795013	107795273	1	41.5	50.0	25.9	63.1
chr18	Siglec15	Intron4	Y	78245612	78245968	2	39.1	40.8	2.3	38.5
chr2	Xkr7	Intron1	Y	152858335	152878449	4	51.9	54.9	29.7	64.6
chr6	Clec2l	Intron1	Y	38613416	38623441	3	38.3	24.8	0.7	34.6
chr11	Epn3	3-UTR	Y	94350913	94352464	2	66.7	78.6	39.3	72.9
chr6	Tcf7l1	Intron10	Y	72599198	72738204	5	74.3	75.5	27.6	60.8
chr5	Dnah10	Intron1	Y	125205751	125207197	2	63.5	66.9	41.2	73.6
chr5	Sirt4	Intron4	Y	115933128	115934340	3	35.6	27.2	0.4	31.9
chr10	Cnn2	Intron1	Y	79451486	79454104	6	46.3	47.2	46.1	77.5
chr4	Ptchd2	Intron19	Y	147644706	147645549	2	28.8	36.9	38.3	68.8
chr18	Pcdhga9	LastExon	Y	37999557	38001517	4	90.6	88.1	50.1	79.4
chr11	Fbxo39	Intron1	Y	72128038	72130246	2	56.0	69.5	33.6	62.7
chr4	Prkcz	Intron14	Y	154675125	154728762	7	60.4	70.3	48.0	76.6
chr8	Jak3	3-UTR	Y	74212047	74214476	10	63.1	67.3	38.0	66.7
chr7	Bcl3	Exon4	Y	20396669	20396757	1	18.4	32.6	12.9	41.0
chr8	Jak3	Intron24	Y	74212062	74214070	8	63.1	64.7	32.1	59.8
chr18	Dsg2	Intron13	Y	20754583	20756839	1	72.0	80.0	67.4	95.1
chr7	Eid2b	3-UTR	Y	29063256	29065148	2	95.4	93.7	66.1	93.7
chr15	Baiap2l2	Intron4	Y	79090205	79091503	2	70.8	82.4	57.2	84.3
chr6	Prdm5	Intron1	Y	65729188	65744293	2	19.2	29.8	12.5	39.3
chr11	Nr1d1	Intron1	Y	98629734	98630362	2	21.0	27.0	15.0	41.4
chr5	Ncf1	Intron2	Y	134698046	134698551	3	53.0	52.4	24.9	51.0
chr1	Ugt1a6b	Intron2	Y	90004375	90111618	6	71.2	77.3	33.4	59.2
chr1	Ugt1a10	Intron1	Y	89952912	90111618	6	71.2	77.3	33.4	59.2
chr1	Ugt1a5	Intron1	Y	90063479	90111618	6	71.2	77.3	33.4	59.2
chr11	Rasgef1c	Intron1	Y	49715470	49770535	6	80.8	74.6	39.7	65.1
chr14	Ripk3	Downstrea	Y	56402832	56403831	3	37.5	49.1	38.5	64.0
chr19	Gal	Intron4	Y	3413350	3414031	1	39.6	37.5	22.4	47.1
chr7	Fkrp	LastExon	Y	17394616	17397325	9	90.6	84.5	52.0	76.6
chr4	Lrp8	Intron4	Y	107515950	107520045	7	74.7	83.1	45.0	69.5
chr7	Tnnt1	Intron3	Y	4459215	4460075	4	65.3	79.9	60.0	84.5
chr6	Dctn1	Exon5	Y	83136422	83136439	1	67.2	77.0	46.6	71.0
chr8	Lrrc25	Downstrea	Y	73144750	73145749	2	51.7	65.3	40.7	64.1
chr7	Phox2a	Intron1	Y	108967229	108969294	3	29.1	32.5	22.8	46.1
chr16	Liph	Exon6	Y	21976306	21976407	1	29.6	44.0	24.8	48.1

chr6	Dnah6	Intron71	Y	73142808	73142893	1	58.9	50.8	54.9	78.0
chr8	Zfhx3	Intron1	Y	111239231	111316101	5	90.0	93.1	69.1	92.0
chr15	Hdac7	Intron12	Y	97632982	97633113	1	55.0	61.8	53.0	75.8
chr2	Spef1	LastExon	Y	130995997	130997526	3	26.5	41.0	18.1	40.8
chr7	Trim72	LastExon	Y	135153401	135154907	4	79.8	80.9	56.9	79.5
chr2	Psd4	Intron15	Y	24260972	24261206	1	44.0	52.9	40.3	62.7
chr8	Stox2	Intron4	Y	48288628	48437384	14	56.3	67.7	10.3	32.5
chr16	Tmem191c	Exon6	Y	17277621	17277685	1	17.4	32.0	13.4	35.5
chr5	Tfr2	LastExon	Y	138028057	138028709	2	26.9	32.0	41.8	63.4
chr19	Map4k2	Intron4	Y	6341968	6342254	1	72.7	87.0	58.6	80.0
chr12	Pigh	Intron3	Y	80186844	80190426	2	19.6	17.2	0.3	21.6
chr17	Gm7325	Intron1	Y	45738635	45738959	1	49.2	49.2	39.3	60.6
chr1	Des	Intron1	Y	75357568	75358732	2	52.9	43.8	43.4	64.6
chr5	Zfp316	Intron1	Y	144017245	144024471	1	77.3	69.0	64.3	85.5
chr11	Cntnap1	Exon7	Y	101041624	101041885	1	57.1	70.9	54.0	74.7
chr4	1300002K09Rik	Intron7	Y	45882078	45882431	2	71.1	77.7	64.3	84.7
chr15	Chadl	Intron4	Y	81525674	81526279	1	75.9	82.1	66.4	86.6
chr7	Slc17a7	Intron9	Y	52427561	52428261	1	21.4	13.9	14.8	35.0
chr17	Usp49	Exon2	Y	47808993	47810367	8	83.4	90.8	54.1	74.3
chr1	Lefty1	Intron3	Y	182867386	182867742	1	53.1	66.7	29.2	49.2
chr2	Pax8	Intron10	Y	24300252	24330592	7	40.6	43.6	43.0	63.0
chr17	4930539E08Rik	Exon10	Y	29046237	29046817	3	39.6	42.0	18.7	38.5
chr11	Krt12	Intron2	Y	99278280	99279228	1	58.1	62.1	47.2	67.0
chr6	Slc6a11	Intron1	Y	114081512	114084592	2	43.1	42.5	23.4	43.2
chr7	Myo7a	Intron33	Y	105232177	105233893	2	51.9	63.5	43.3	63.1
chr4	Ttll10	Intron1	Y	155409561	155409854	1	63.7	76.3	58.2	77.8
chr6	Hoxa3	LastExon	Y	52119061	52120741	6	76.5	88.2	69.5	89.1
chr8	Acta1	Intron4	Y	126416895	126417045	1	33.7	38.1	22.7	42.3
chr19	Rab1b	Downstre	Y	5098207	5099206	5	30.0	41.3	18.0	37.5
chr19	Kcnk4	LastExon	Y	7000180	7000865	3	18.5	29.5	16.9	36.3
chr8	Foxf1	Intron1	Y	123609275	123610618	1	24.0	27.6	14.7	34.0
chr3	Gipc2	Intron2	Y	151765665	151770904	7	61.6	73.7	58.2	77.3
chr5	Sirt4	Exon3	Y	115932633	115933127	3	68.1	80.4	65.9	84.9
chr11	Tbkbp1	Intron3	Y	97000905	97007613	1	46.6	60.7	61.1	80.0
chr6	Eva1a	LastExon	Y	82041788	82043093	3	49.7	55.5	38.4	57.3
chr17	Anks1	Exon11	Y	28144794	28145377	2	68.8	80.8	64.3	83.1
chr15	C1ql4	LastExon	Y	98915185	98915364	1	31.3	40.8	25.6	44.4
chr17	Efna5	Intron4	Y	63000484	63230354	9	91.9	92.0	26.4	45.1
chr15	Zfp641	Intron5	Y	98124260	98126485	4	0.7	3.0	29.7	48.3
chr6	2010107G12Rik	Intron1	Y	34896227	34903764	3	14.5	28.3	5.7	24.3
chr4	Tmeff1	Exon4	Y	48630107	48630203	1	90.5	94.1	74.9	93.5
chr7	Myh14	Intron31	Y	51896550	51898833	2	74.4	72.7	57.2	75.6
chr17	Notch4	Intron27	Y	34723155	34723471	1	8.3	15.2	2.9	21.2
chr12	Wdr25	Intron2	Y	110136936	110218900	3	17.1	28.6	16.0	34.2
chr10	Dos	Intron1	Y	79594997	79595749	1	90.0	82.1	79.4	97.5
chr4	Arhgef19	Exon4	Y	140803293	140803401	1	29.3	44.1	30.1	48.2
chr12	Hhipl1	Intron8	Y	109565419	109565877	2	70.0	76.7	62.2	80.1
chr8	Gpr124	LastExon	Y	28231238	28233908	7	67.3	64.1	53.8	71.2
chr14	Comtd1	LastExon	Y	22665083	22666649	2	37.4	42.6	22.0	39.3

chr17	Abcg8	Intron5	Y	85091957	85092050	1	80.5	93.0	75.5	92.7
chr5	Tmem184a	Exon4	Y	140283748	140283823	1	45.5	56.8	54.0	71.2
chr14	Fam124a	Intron3	Y	63206724	63224709	1	66.4	73.6	51.6	68.6
chr8	B3gnt3	Intron1	Y	74217055	74217215	1	82.3	94.0	72.0	89.0
chr17	Angptl4	Intron6	Y	33917839	33918007	1	51.1	41.7	40.0	56.9
chr18	Arap3	Intron17	Y	38145187	38147208	1	75.8	69.0	73.5	90.4
chr4	A3galt2	3-UTR	Y	128444917	128446542	1	64.3	76.9	73.2	90.0
chr17	Prrt1	Intron1	Y	34766650	34767577	2	72.5	84.5	67.3	84.1
chr2	C330006A16Rik	LastExon	Y	25992328	25994433	4	90.6	92.9	72.9	89.6
chr7	Irgq	3-UTR	Y	25319507	25323619	1	79.5	81.1	69.3	86.1
chr15	Olfr279	Downstrea	Y	98328838	98329837	1	84.4	86.8	77.9	94.6
chr13	Spata31d1d	LastExon	Y	59827286	59830778	3	50.6	43.5	23.1	39.8
chr15	Cacng2	LastExon	Y	77824053	77826114	4	84.3	75.2	63.7	80.4
chr12	Adssl1	Intron1	Y	113858522	113866511	2	19.0	26.9	9.0	25.7
chr10	Tmprss9	Intron10	Y	80353242	80354901	1	73.8	78.6	63.9	80.5
chr11	Hdac5	Exon25	Y	102086048	102086269	2	7.5	19.0	14.6	31.2
chr7	Ryr1	Intron93	Y	29890076	29894218	1	10.6	21.2	10.4	26.9
chr10	Hcn2	Intron3	Y	79189054	79191637	1	50.0	48.4	49.2	65.6
chr15	Letmd1	Intron2	Y	100300268	100302090	1	19.8	25.4	14.1	30.5
chr16	Rwdd2b	Exon2	Y	87437309	87437376	1	80.4	87.0	74.7	91.1
chr9	Pth1r	Intron9	Y	110630010	110630181	1	93.1	88.9	77.4	93.8
chr11	Fdxr	Downstrea	Y	115128339	115129338	3	32.0	33.3	12.7	29.0
chr10	Cradd	Intron1	Y	94638613	94785219	6	81.2	90.1	52.4	68.7
chr7	Sbk1	Intron3	Y	133434760	133435338	1	81.8	85.4	68.6	84.9
chr11	Sarm1	Intron4	Y	78297159	78300724	1	4.5	18.5	13.5	29.7
chr8	Palm3	Intron2	Y	86548300	86550092	2	19.7	19.1	13.7	29.9
chr6	Hoxa3	Downstrea	Y	52118061	52119060	3	78.3	85.2	68.9	85.0
chr11	Myo15	Intron23	Y	60310071	60310419	1	57.7	53.7	58.6	74.7
chr1	Rassf5	Intron5	Y	133108956	133141265	3	70.4	75.0	51.1	67.2
chr9	Keap1	Exon4	Y	21041515	21042200	4	73.9	80.4	40.6	56.7
chr4	Trabd2b	Intron1	Y	114079525	114081497	2	33.4	45.7	32.5	48.6
chr9	Igdcc4	Intron7	Y	64971997	64972418	1	70.8	82.5	61.9	78.0
chr5	Crmp1	Intron1	Y	37633765	37656470	12	31.3	29.0	15.3	31.4
chr5	Cit	Intron8	Y	116336817	116358646	4	29.1	40.8	27.3	43.2
chr1	Kiss1	Downstrea	Y	135226300	135227299	1	15.0	19.8	17.4	33.3
chr19	Kcnk4	3-UTR	Y	7000180	7000472	2	18.5	29.5	16.2	32.1
chr7	Bcl3	Exon3	Y	20396406	20396483	1	60.4	74.4	62.5	78.3
chr17	Pi16	Intron3	Y	29462928	29463218	1	73.9	74.4	67.6	83.4
chr6	Fam19a1	Intron2	Y	96065749	96473328	6	44.5	42.8	31.0	46.9
chr8	Thap11	3-UTR	Y	108380179	108380850	1	32.3	24.5	17.2	33.1
chr11	Arhgap27	Exon3	Y	103194885	103194962	1	64.8	77.6	57.4	73.2
chr19	Kcnk4	Intron1	Y	7000866	7001580	3	20.9	33.3	18.6	34.5
chr1	A630095N17Rik	LastExon	Y	75216670	75217148	2	63.8	66.0	53.3	69.1
chr1	A630095N17Rik	3-UTR	Y	75216670	75217034	2	63.8	66.0	53.3	69.1
chr12	Dlk1	Intron2	Y	110691937	110693194	2	31.8	40.6	25.2	41.0
chr6	Mical3	Intron6	Y	120907812	120908258	1	85.3	100.0	84.2	100.0
chr17	Plekhh2	Intron2	Y	84921166	84946802	2	89.2	92.0	76.7	92.4
chr4	Epha10	Intron9	Y	124589310	124589426	1	66.1	68.6	56.7	72.4
chr4	Chd5	Intron7	Y	151733921	151734672	1	35.8	30.8	30.0	45.6

chr5	Ncor2	Intron30	Y	125536246	125546209	7	87.1	95.7	54.6	70.1
chr1	Zfp281	LastExon	Y	138521849	138526968	6	46.7	50.3	24.3	39.9
chr18	Wnt8a	Exon2	Y	34702498	34702636	1	50.0	53.8	47.6	63.2
chr12	Syt16	Intron1	Y	75098851	75230358	3	40.0	43.2	24.6	40.1
chr18	Egr1	LastExon	Y	35022119	35024610	2	12.6	27.5	14.3	29.5
chr9	Osbpl10	Exon5	Y	115125667	115126147	2	25.3	17.1	22.5	37.6
chr7	Shank2	Intron1	Y	151361862	151365659	1	71.3	85.6	62.7	77.8
chr1	Zdbf2	3-UTR	Y	63356520	63361149	1	72.3	71.1	46.2	61.3
chr9	Cyp1a2	Intron5	Y	57528919	57529508	1	70.8	84.9	70.5	85.6
chr2	Rtn4rl2	Downstrea	Y	84711103	84712102	3	24.9	30.8	21.7	36.7
chr7	Dact3	Intron3	Y	17468860	17470429	1	72.6	80.7	64.4	79.4
chr6	Hoxa3	Intron1	Y	52120742	52122119	2	85.7	90.6	75.6	90.6

Group 3-mDMR genes associated with methylation loss at 5' CGI

							DNA Methylation Level (%)			
							ISCs		Diff. Cells	
**Chr**	**Gene**	**Region**	**CGI**	**Start**	**End**	**#Bins**	**P0**	**P21**	**P0**	**P21**
chr5	Klhl8	Upstream	Y	104340249	104341248	3	46.1	26.9	45.3	1.0
chr6	Lpar5	5-UTR	Y	125017938	125031335	2	61.3	10.2	77.0	34.7
chr9	Mir425	5-UTR	Y	108471108	108471192	1	57.6	2.4	53.8	15.5
chr3	Glrb	Upstream	Y	80717547	80718546	4	60.9	33.3	36.8	0.7
chr9	1600029D21Rik	Exon1	Y	50307200	50307404	2	69.5	52.7	76.5	41.5
chr9	2310014F07Rik	Exon1	Y	50307031	50307275	1	71.0	45.7	75.3	41.5
chr18	Proc	5-UTR	Y	32295585	32299224	1	65.0	15.2	37.8	10.4
chr18	Proc	Exon1	Y	32299164	32299224	1	65.0	15.2	37.8	10.4
chr14	Klhl33	Exon1	Y	51512050	51512930	3	58.6	37.5	83.6	56.6
chr14	Haus4	Upstream	Y	55173199	55174198	4	26.9	11.3	29.9	3.2
chr13	Tmem174	5-UTR	Y	99407276	99407365	1	77.8	48.9	82.8	57.9
chr7	Oscar	Exon1	Y	3562671	3562952	1	58.1	30.1	59.7	35.0
chr19	Bloc1s2	Upstream	Y	44220937	44221936	2	33.6	10.3	35.9	12.6
chr4	Pank4	Upstream	Y	154337242	154338241	3	54.4	33.6	60.8	37.5
chr11	Sfi1	Exon1	Y	3032219	3032330	1	82.5	60.2	80.2	58.2
chr9	Vstm5	Upstream	Y	15042489	15043488	3	20.1	0.0	36.4	15.5
chr9	Acat1	Upstream	Y	53418456	53419455	2	26.3	7.3	30.6	11.0
chr4	Tex10	Upstream	Y	48486295	48487294	2	37.1	6.6	25.3	5.9
chr6	Slc41a3	Upstream	Y	90553874	90554873	3	66.7	33.3	19.1	0.4
chr9	Dalrd3	Upstream	Y	108471223	108472222	2	23.5	0.5	23.1	4.4
chr15	A4galt	Upstream	Y	83082205	83083204	4	55.0	34.4	50.3	32.1
chr3	Ppa2	Upstream	Y	132972080	132973079	3	53.2	31.0	34.4	16.2
chr13	Txndc5	Upstream	Y	38620330	38621329	4	23.4	8.4	21.6	4.8
chr19	Neat1	5-UTR	Y	5842302	5845478	4	28.4	0.8	20.7	4.0
chr10	Fam26f	5-UTR	Y	33847716	33847778	1	69.2	46.9	74.3	57.8
chr18	Proc	Upstream	Y	32299225	32300224	2	43.8	2.9	22.9	6.5
chr8	Syce2	Upstream	Y	87395010	87396009	3	62.0	45.0	42.6	27.4
chr13	Cltb	Exon1	Y	54700083	54700194	1	32.8	8.1	20.6	6.0
chr6	Crbn	Upstream	Y	106750082	106751081	2	29.3	2.2	16.2	2.0
chr17	Twsg1	Upstream	Y	66300528	66301527	3	21.6	0.6	16.9	3.0
chr6	Mkrn1	Upstream	Y	39370369	39371368	3	55.0	40.0	14.1	0.6
chr2	Lcmt2	Exon1	Y	120963028	120966434	8	47.1	9.9	15.2	1.7

chr2	Lrrc26	Upstream	Y	25144431	25145430	1	33.3	0.0	13.0	0.0
chr14	Gjb2	5-UTR	Y	57719587	57723539	7	33.7	8.3	20.0	7.1
chr9	Usp19	Upstream	Y	108392007	108393006	4	42.9	23.7	30.0	17.7
chr17	Gtf2a1l	5-UTR	Y	89068000	89068050	1	46.6	18.5	28.6	16.4
chr17	Gtf2a1l	Exon1	Y	89068000	89068071	2	46.6	18.5	28.6	16.4
chr8	Bcar1	Upstream	Y	114267750	114268749	4	33.4	14.7	21.3	9.2
chr17	Rftn1	Upstream	Y	50329823	50330822	4	42.3	16.4	24.9	13.1
chr17	Slc3a1	Exon1	Y	85427687	85428198	1	84.3	61.4	73.9	63.2
chr15	Racgap1	Upstream	Y	99482088	99483087	2	33.3	12.0	10.5	0.0
chr7	Bbc3	Upstream	Y	16893932	16894931	5	39.0	18.5	21.5	11.1
chr7	Rras2	Upstream	Y	121261296	121262295	3	17.3	2.0	13.1	3.1
chr17	Clip4	5-UTR	Y	72119031	72139222	8	66.8	35.8	34.2	24.4
chr17	Srrm2	5-UTR	Y	23940154	23945078	8	31.9	13.3	18.6	8.9
chr10	Fam26f	Upstream	Y	33847779	33848778	1	79.5	47.8	76.2	66.7
chr16	Htr1f	5-UTR	Y	64926754	65105610	3	59.3	26.6	27.5	18.0
chr11	Ube2z	Upstream	Y	95926679	95927678	4	32.7	14.7	9.7	0.6
chr2	Dhrs9	Exon1	Y	69218519	69218688	1	42.9	11.3	20.8	12.1
chr15	Lima1	5-UTR	Y	99674225	99705887	4	35.9	7.3	29.5	20.8
chr12	Acot5	Upstream	Y	85409275	85410274	1	29.4	5.4	20.0	11.6
chr18	Mbd2	Upstream	Y	70726946	70727945	2	68.2	16.7	27.2	19.0
chr18	2700062C07Rik	Upstream	Y	24628372	24629371	2	58.7	26.7	32.0	24.4
chr11	Cenpv	Upstream	Y	62352764	62353763	2	18.5	1.4	8.6	1.1
chr14	Nisch	Upstream	Y	32020013	32021012	2	44.1	11.7	17.6	10.2
chr7	Sephs2	Exon1	Y	134415395	134417573	5	33.9	12.9	13.8	6.5
chr15	Krt4	Exon1	Y	101754613	101755166	3	94.8	56.3	65.3	58.0
chr7	Homer2	Upstream	Y	88851812	88852811	5	50.4	31.4	27.7	20.8
chr11	Mfsd6l	Exon1	Y	68369688	68371747	3	25.6	2.1	10.1	3.3
chr12	Crip2	Upstream	Y	114377447	114378446	3	35.5	16.2	8.4	1.7
chr2	Rbm45	Upstream	Y	76207041	76208040	2	60.0	28.0	25.6	18.9
chr8	Mbtps1	Upstream	Y	122082662	122083661	3	45.4	16.7	18.3	11.9
chr11	Kif19a	Upstream	Y	114625703	114626702	4	50.0	19.3	20.5	14.1
chr2	Dhrs9	5-UTR	Y	69218519	69230966	2	33.2	9.6	16.1	9.7
chr4	Serinc2	Upstream	Y	129952831	129953830	3	23.3	8.0	13.9	7.7
chr4	Zc3h12a	5-UTR	Y	124804293	124805125	4	22.5	1.0	7.0	1.3
chr11	Hoxb5	Exon1	Y	96164826	96165489	4	37.3	13.0	31.0	25.4
chr2	Hoxd10	Exon1	Y	74529948	74530781	4	33.2	14.1	21.4	15.8
chr2	Asb6	Upstream	Y	30683821	30684820	2	45.0	21.7	27.7	22.2
chr10	Map2k2	Upstream	Y	80567692	80568691	3	44.4	24.0	10.6	5.2
chr1	Bend6	5-UTR	Y	33940406	33964466	5	34.9	3.0	7.9	2.6
chr6	Ggct	Upstream	Y	54942862	54943861	3	24.4	7.9	12.5	7.3
chr14	Fam167a	Upstream	Y	64054231	64055230	4	74.0	35.8	48.2	43.0
chr2	Cables2	Upstream	Y	180008171	180009170	4	35.0	0.3	7.0	1.8
chr15	2310069G16Rik	Upstream	Y	44618309	44619308	2	69.2	46.5	47.3	42.3
chr6	Mrpl53	Upstream	Y	83058102	83059101	2	25.0	7.9	13.8	9.1
chr5	Gnai1	Upstream	Y	17866232	17867231	3	16.9	1.7	7.5	2.7
chr6	Pole4	Upstream	Y	82602860	82603859	3	29.6	11.4	6.2	1.5
chr15	Scrib	Upstream	Y	75900161	75901160	4	85.3	69.5	34.9	30.2
chr10	Fam26f	Exon1	Y	33847197	33847778	4	62.4	46.9	29.9	25.3
chr2	Mcts2	5-UTR	Y	152512884	152513006	1	71.7	47.6	63.6	59.1

chr2	Mcts2	Exon1	Y	152512884	152513678	1	71.7	47.6	63.6	59.1
chr17	Cdc42ep3	5-UTR	Y	79734830	79754431	8	57.1	38.9	40.7	36.3
chr6	Dfna5	Upstream	Y	50211769	50212768	3	17.9	0.9	8.9	4.5
chr17	2310061I04Rik	Upstream	Y	36034324	36035323	4	57.8	29.6	13.0	8.7
chr9	Igdcc4	Upstream	Y	64948302	64949301	4	72.0	25.7	32.5	28.2
chr6	Csgalnact2	Upstream	Y	118089159	118090158	2	66.1	0.4	32.1	28.0
chr3	Lamtor5	Upstream	Y	107080776	107081775	1	47.5	25.3	26.8	22.9
chr11	Krt20	Exon1	Y	99299001	99299467	3	85.8	53.7	60.2	56.3
chr14	Akap11	Upstream	Y	78936668	78937667	4	72.4	38.1	31.6	27.8
chr5	Rac1	Upstream	Y	144288862	144289861	4	25.8	6.6	8.9	5.3
chr3	Chd1l	Upstream	Y	97414114	97415113	2	48.1	12.4	23.0	19.4
chr11	Exoc7	Upstream	Y	116168053	116169052	3	52.1	6.3	34.8	31.2
chr11	Olfr317	Exon1	Y	58545598	58546725	2	89.8	73.7	73.6	70.2
chr7	Efcab4a	5-UTR	Y	148646993	148649309	7	20.5	5.5	4.4	1.2
chr2	Ncoa3	Upstream	Y	165817137	165818136	3	57.6	37.7	41.7	38.7
chr12	Insm2	Exon1	Y	56699904	56703004	12	54.1	37.2	8.2	5.5
chr13	Ell2	Upstream	Y	75843932	75844931	3	37.8	14.9	10.8	8.1
chr8	Pgbd5	5-UTR	Y	126908509	126957836	5	74.8	29.2	22.1	19.5
chr4	AA415398	Upstream	Y	119211375	119212374	5	25.5	8.3	5.4	2.7
chr4	Foxj3	Upstream	Y	119211293	119212292	5	25.5	8.3	5.4	2.7
chr7	Eid2b	Exon1	Y	29062725	29065148	5	95.4	47.1	26.8	24.3
chr8	Agpat5	Upstream	Y	18845279	18846278	3	23.9	3.2	3.8	1.5
chr13	Kdm1b	5-UTR	Y	47138868	47143284	7	16.5	0.0	3.7	1.7
chr13	Nhlrc1	Upstream	Y	47110220	47111219	2	58.5	37.0	31.1	29.1
chr18	Pcdhb11	Exon1	Y	37581072	37584686	4	86.0	53.1	64.2	62.2
chr7	Stx4a	Upstream	Y	134984322	134985321	5	24.9	7.5	6.9	5.1
chr3	Slc27a3	Upstream	Y	90193850	90194849	3	72.0	48.4	3.5	1.8
chr4	Foxd2	Exon1	Y	114578885	114581503	11	20.0	4.3	2.5	0.9
chr10	Txnrd1	5-UTR	Y	82296696	82335714	12	54.4	29.8	24.9	23.4
chr5	Caln1	5-UTR	Y	130845328	130890697	3	96.1	77.4	90.7	89.2
chr2	Lamc3	Upstream	Y	31741801	31742800	2	55.2	18.5	24.8	23.4
chr17	Apobec2	Upstream	Y	48572054	48573053	2	83.8	63.2	66.9	65.5
chr8	Coprs	Upstream	Y	13890272	13891271	3	43.7	25.3	16.9	15.5
chr16	5-Sep	Upstream	Y	18630032	18631031	4	45.7	4.8	10.3	9.0
chr17	Rab31	Upstream	Y	66122093	66123092	2	19.4	1.6	1.4	0.2
chr15	Pacsin2	Upstream	Y	83295037	83296036	3	42.5	26.4	27.4	26.3
chr10	Gipc3	Upstream	Y	80806012	80807011	3	53.8	22.7	2.5	1.5
chr11	Hoxb5	5-UTR	Y	96164826	96164927	1	28.0	12.6	23.4	22.4
chr9	Klhl40	Exon1	Y	121686725	121688045	7	75.9	53.3	33.4	32.4
chr12	Atg2b	Upstream	Y	106923452	106924451	5	24.8	9.3	4.6	3.6
chr9	Csk	Upstream	Y	57492988	57493987	5	68.2	31.5	15.7	14.8
chr16	Ppl	Upstream	Y	5132575	5133574	4	45.2	23.1	25.3	24.4
chr1	Dis3l2	Upstream	Y	88599379	88600378	3	39.3	9.2	8.8	7.9
chr1	Sned1	Upstream	Y	95131474	95132473	3	76.9	3.1	1.7	0.9
chr6	Wdr54	Upstream	Y	83106374	83107373	3	23.5	4.2	8.1	7.3
chr8	Jph3	Exon1	Y	124276867	124277644	4	65.0	39.3	51.4	50.7
chr17	Fem1a	Exon1	Y	56396216	56403031	7	45.2	18.9	17.1	16.4
chr11	Tbc1d16	5-UTR	Y	119071997	119089813	9	93.0	42.7	23.4	22.7
chr9	Cmc1	Upstream	Y	118059315	118060314	2	32.6	15.1	14.4	13.7

chr1	Pigm	Exon1	Y	174306662	174314230	6	56.6	16.4	11.7	11.2
chr9	Nckipsd	Upstream	Y	108709711	108710710	3	56.1	24.6	22.9	22.4
chr9	2410012M07Rik	Upstream	Y	98764186	98765185	1	40.9	20.0	42.2	41.9
chr8	Mfap3l	5-UTR	Y	63111657	63135388	6	93.3	62.3	30.9	30.7
chr13	Arhgef28	5-UTR	Y	98915540	98976120	5	83.9	38.9	33.0	32.9
chr8	4930432K21Rik	5-UTR	Y	86671937	86672756	3	75.4	44.4	42.9	42.8
chr8	4930432K21Rik	Exon1	Y	86671937	86672297	3	75.4	44.4	42.9	42.8
chr5	Nptx2	Upstream	Y	145305756	145306755	4	42.1	24.9	21.2	21.2
chr11	Smcr7	5-UTR	Y	60541900	60543790	4	26.0	9.1	3.8	3.9
chr18	Adrb2	Upstream	Y	62339636	62340635	4	47.6	19.2	9.0	9.2
chr8	Cc2d1a	Upstream	Y	86671653	86672652	5	75.4	33.4	25.8	25.9
chr5	Amz1	5-UTR	Y	141200081	141217237	5	95.3	47.6	23.5	23.7
chr17	Mrps34	Upstream	Y	25031065	25032064	4	18.8	0.0	2.2	2.4
chr17	Ubxn6	Upstream	Y	56214413	56215412	2	28.6	12.0	16.3	16.5
chr7	Dhx32	5-UTR	Y	140951386	140968486	8	93.3	62.2	27.3	27.6
chr4	Mdn1	Upstream	Y	32743094	32744093	3	21.6	0.1	0.2	0.5
chr10	Dapk3	Upstream	Y	80644752	80645751	3	49.8	15.9	13.5	13.8
chr10	Aes	Upstream	Y	81021189	81022188	3	16.7	1.7	2.2	2.6
chr8	Cln8	5-UTR	Y	14888536	14894687	7	46.9	1.0	27.3	27.6
chr12	Itpk1	Upstream	Y	103943080	103944079	5	36.8	15.8	7.2	7.5
chr17	2810468N07Rik	Upstream	Y	25706756	25707755	5	44.1	0.0	13.5	14.0
chr13	Elmo1	5-UTR	Y	20182376	20277295	8	87.6	48.6	13.9	14.4
chr1	Ankzf1	Upstream	Y	75187734	75188733	5	47.6	25.9	12.6	13.2
chr7	Nfkbid	Upstream	Y	31207323	31208322	5	41.7	21.1	0.3	0.9
chr6	Wnt5b	5-UTR	Y	119398234	119494365	23	21.7	4.8	2.8	3.5
chr1	Slco5a1	Exon1	Y	12979661	12981216	6	56.7	24.9	20.0	20.6
chr6	Rad18	Upstream	Y	112646665	112647664	2	24.3	0.7	0.4	1.1
chr8	Dda1	Upstream	Y	73992093	73993092	2	44.4	21.3	14.3	15.0
chr2	Prrc2b	Upstream	Y	32005668	32006667	4	21.3	0.4	1.3	2.0
chr1	Paqr8	5-UTR	Y	20880703	20924704	7	24.3	0.9	15.9	16.7
chr7	Lrfn3	5-UTR	Y	31145818	31147791	2	88.0	55.6	57.4	58.2
chr11	Cdk5r1	Exon1	Y	80290548	80294681	6	86.4	34.1	23.4	24.3
chr7	Tsku	5-UTR	Y	105501633	105509838	9	40.4	18.8	5.6	6.6
chr7	Mef2a	Upstream	Y	74517745	74518744	5	18.2	0.3	4.5	5.6
chr5	Mtus2	5-UTR	Y	148768896	148887974	6	48.5	11.5	14.9	16.0
chr15	Zfp623	5-UTR	Y	75771382	75777626	5	22.9	1.0	3.7	4.8
chr6	Wnt7a	Upstream	Y	91361364	91362363	5	55.7	30.7	29.0	30.2
chr10	BC048403	Upstream	Y	121175993	121176992	3	39.5	16.8	10.5	11.8
chr15	Trmt12	Exon1	Y	58704204	58708336	5	40.9	21.5	15.6	16.9
chr19	Erlin1	Upstream	Y	44144276	44145275	3	64.1	32.5	20.9	22.3
chr5	Rgs12	5-UTR	Y	35292097	35307523	4	37.2	2.4	1.5	2.9
chr7	Zfp536	5-UTR	Y	38355009	38554771	13	24.2	1.5	30.9	32.4
chr16	B3galt5	5-UTR	Y	96457408	96536775	5	86.8	50.8	22.3	23.8
chr13	Nhlrc1	Exon1	Y	47107926	47110219	6	72.6	37.2	26.0	27.8
chr16	Hmox2	5-UTR	Y	4726361	4762594	7	24.4	1.2	3.8	5.6
chr1	Atg9a	5-UTR	Y	75186980	75188497	4	47.6	25.9	15.6	17.5
chr6	Vopp1	Upstream	Y	57775120	57776119	3	77.9	43.4	0.3	2.6
chr8	Afg3l1	Upstream	Y	126000762	126001761	4	33.7	13.8	9.0	11.4
chr8	Thap11	Exon1	Y	108379003	108380850	7	32.3	14.9	3.2	5.7

chr13	Nkd2	Upstream	Y	73985080	73986079	3	58.7	42.6	26.3	28.8
chr1	Cryba2	Upstream	Y	74939710	74940709	3	32.0	16.8	9.0	11.6
chr15	Pdxp	Upstream	Y	78743349	78744348	4	43.9	16.1	33.3	35.9
chr13	4833422C13Rik	5-UTR	Y	91881027	91881477	3	20.0	3.3	4.6	7.2
chr19	Dagla	5-UTR	Y	10346079	10379367	6	63.2	40.4	15.7	18.6
chr13	Rnf144b	5-UTR	Y	47218089	47302754	9	85.6	36.6	19.8	22.8
chr4	Zbtb48	Upstream	Y	151401781	151402780	4	29.3	13.1	6.1	9.2
chr13	Gprin1	5-UTR	Y	54841821	54851030	5	64.6	37.2	32.5	35.6
chr18	Pcdhac1	Exon1	Y	37249790	37252225	4	53.8	36.4	39.3	42.6
chr11	Dlx4	Upstream	Y	95007116	95008115	4	23.5	1.0	15.4	18.7
chr18	Cxxc5	5-UTR	Y	35989472	36018201	20	25.1	6.9	21.2	24.6
chr7	Clns1a	Upstream	Y	104844167	104845166	3	74.3	38.9	34.0	37.6
chr5	Ythdc1	Upstream	Y	87232515	87233514	3	17.2	0.3	4.5	8.2
chr18	Kcng2	5-UTR	Y	80519875	80560993	12	66.8	51.8	28.9	32.6
chr9	Ttc12	Upstream	Y	49294331	49295330	2	27.4	8.3	13.3	17.1
chr5	Gltp	Upstream	Y	115140945	115141944	4	76.0	30.4	22.1	25.8
chr16	Thpo	5-UTR	Y	20729150	20734584	14	61.5	46.1	24.8	28.7
chr6	Fbln2	Exon1	Y	91183057	91184345	4	89.1	57.0	64.6	68.6
chr15	Mtss1	Upstream	Y	58913582	58914581	4	21.3	1.7	3.5	7.4
chr17	Tbc1d24	5-UTR	Y	24323114	24342507	4	31.6	7.5	13.6	17.6
chr7	Chid1	Upstream	Y	148725757	148726756	3	56.3	18.8	18.6	22.7
chr9	Dock3	Upstream	Y	107134241	107135240	3	57.8	22.8	54.8	58.8
chr19	Dock8	Upstream	Y	25073019	25074018	4	65.8	47.5	20.3	24.3
chr2	Epb4.1l1	5-UTR	Y	156246788	156319653	14	66.8	48.3	13.6	17.6
chr17	Nrxn1	5-UTR	Y	91488067	91492142	4	44.7	5.3	51.4	55.7
chr3	Rarres1	Upstream	Y	67319446	67320445	2	56.0	22.9	23.3	27.7
chr10	Fam211b	Upstream	Y	75023076	75024075	3	30.0	2.8	26.9	31.4
chr11	Rara	5-UTR	Y	98799010	98811931	7	67.4	49.6	19.6	24.1
chr15	Kctd17	Upstream	Y	78258058	78259057	2	84.3	45.9	44.6	49.1
chr5	Chst12	5-UTR	Y	140981563	140999573	5	44.8	28.6	18.3	22.9
chr11	Foxi1	Exon1	Y	34107468	34108089	4	85.3	58.6	63.9	69.0
chr17	Fkbp5	Upstream	Y	28623095	28624094	3	31.0	3.7	19.2	24.4
chr4	Bach2	5-UTR	Y	32504410	32588599	2	46.3	28.6	26.6	31.8
chr9	Prss50	Upstream	Y	110759471	110760470	2	62.5	45.9	39.3	44.9
chr12	Tmem121	Upstream	Y	114423114	114424113	4	37.6	14.2	24.2	30.1
chr8	Plekhg4	Upstream	Y	107898281	107899280	4	94.1	71.2	51.6	57.5
chr2	Ppp1r3d	Exon1	Y	178145911	178149168	7	68.3	41.1	25.2	31.3
chr6	H1fx	Upstream	Y	87931477	87932476	5	27.3	11.9	3.9	10.1
chr11	Wscd1	5-UTR	Y	71564205	71573350	6	26.9	2.8	4.0	10.4
chr11	Grb10	5-UTR	Y	11870620	11937423	15	66.6	45.1	39.5	46.3
chr10	Celf5	Upstream	Y	80945455	80946454	2	55.6	27.4	23.9	30.7
chr11	Nsf	Upstream	Y	103815371	103816370	2	25.0	1.2	9.6	16.5
chr11	Mafg	Upstream	Y	120494862	120495861	4	53.1	18.9	17.3	24.3
chr18	Mapk4	5-UTR	Y	74130091	74224603	8	31.0	2.3	11.8	18.9
chr4	Tmem215	5-UTR	Y	40419213	40420957	6	18.4	1.4	6.3	14.1
chr4	Tmem215	Exon1	Y	40419213	40420637	6	18.4	1.4	6.3	14.1
chr6	Slc35b4	Upstream	Y	34127055	34128054	2	20.3	0.0	0.7	8.5
chr2	Hoxd12	Upstream	Y	74512070	74513069	2	26.6	5.0	14.3	22.4
chr1	Agap1	Upstream	Y	91350386	91351385	5	71.4	30.1	10.0	18.6

chr17	Tbc1d5	Upstream	Y	51318675	51319674	3	45.0	7.2	0.5	9.2
chr14	Dzip1	Upstream	Y	119322390	119323389	2	76.8	56.0	52.8	61.5
chr2	Nr4a2	Upstream	Y	56976415	56977414	4	52.1	29.4	16.8	26.2
chr11	Col1a1	Exon1	Y	94797584	94797758	2	83.0	65.1	46.6	56.1
chr4	Slc46a2	Exon1	Y	59926662	59927928	5	83.1	61.1	31.4	41.1
chr5	Steap4	5-UTR	Y	7960472	7975440	3	0.0	0.4	33.6	1.0
chr13	Ninj1	Upstream	Y	49281916	49282915	4	0.0	1.2	27.1	1.1
chr7	Zkscan2	5-UTR	Y	130643482	130643963	2	13.6	12.1	39.0	18.9
chr9	Eepd1	Upstream	Y	25288182	25289181	2	61.5	71.4	77.1	60.7

Group 4-mDMR genes associated with methylation loss at gene body and 3' CGI

							DNA Methylation Level (%)			
							ISCs		Diff. Cells	
Chr	Gene	Region	CGI	Start	End	#Bins	P0	P21	P0	P21
chr2	St6galnac4	Intron5	Y	32451322	32452533	1	74.2	58.8	84.9	25.3
chr13	Cltb	Exon2	Y	54700405	54700522	1	77.8	23.1	63.1	15.2
chr13	Itga2	Intron28	Y	115676898	115690622	3	61.5	3.2	49.6	3.2
chr7	Nlrp6	Intron1	Y	148106986	148107539	1	19.0	1.6	44.8	0.0
chr6	2310001H17Rik	Intron2	Y	129183172	129187756	3	55.8	24.6	65.6	21.3
chr6	Lpar5	Intron1	Y	125017995	125031154	2	61.3	10.2	77.0	34.7
chr6	Cand2	Exon9	Y	115741687	115743189	6	91.7	41.7	85.5	45.1
chr9	Mir191	Downstrea	Y	108470724	108471723	3	51.9	9.7	55.1	17.9
chr13	Cltb	Intron3	Y	54700523	54708004	1	77.4	41.4	80.8	43.8
chr5	Setd8	Intron5	Y	124901383	124907067	3	84.9	34.7	70.8	33.9
chr8	3010033K07Rik	Intron1	Y	111077524	111108055	2	79.6	63.2	85.7	50.1
chr9	Celsr3	Intron24	Y	108745632	108745874	1	37.5	15.4	42.3	7.8
chr17	Tmem151b	Intron1	Y	45682904	45683709	3	55.1	33.7	61.2	27.6
chr7	Lipt2	LastExon	Y	107308684	107309441	1	54.8	33.1	67.8	36.3
chr11	Hoxb4	3-UTR	Y	96181643	96182952	4	48.3	19.4	57.1	28.8
chr16	Htr1f	Intron2	Y	65074056	65105450	2	59.3	26.6	53.9	25.9
chr18	Proc	Intron8	Y	32295606	32299163	1	65.0	15.2	37.8	10.4
chr12	Cdc42bpb	Intron36	Y	112583835	112615307	5	22.3	6.8	31.4	5.2
chr9	Mir425	Downstrea	Y	108471193	108472192	3	34.9	1.2	33.3	8.1
chr15	Srebf2	Intron2	Y	82000585	82001531	1	64.8	16.9	54.2	29.5
chr11	Nek8	Intron7	Y	77982951	77983879	1	29.2	1.2	24.4	0.6
chr19	Pkd2l1	Downstrea	Y	44221127	44222126	2	33.6	10.3	35.9	12.6
chr16	Ephb3	Intron3	Y	21215071	21217268	1	25.6	3.0	28.2	5.5
chr10	Tcf3	Intron3	Y	79876083	79877906	1	90.9	75.0	76.5	54.1
chr2	Tgm2	Intron12	Y	157968911	157972026	2	72.7	0.0	42.7	20.3
chr11	Fbf1	Intron5	Y	116007326	116007749	1	95.2	59.5	88.4	66.3
chr8	4930567H12Rik	Intron2	Y	128109075	128140001	3	49.1	28.9	49.2	27.3
chr2	Cep250	Exon28	Y	155815952	155818510	3	41.8	26.7	47.1	25.5
chr10	Ipmk	LastExon	Y	70843953	70848633	3	47.0	12.1	29.6	8.4
chr7	Kcnq1	Intron1	Y	150293647	150335402	2	21.5	4.7	22.1	1.5
chr15	Myc	LastExon	Y	61820901	61821916	2	80.9	56.9	78.9	58.7
chr15	Panx2	Intron1	Y	88890730	88897987	4	37.1	7.5	25.0	5.3
chr9	1810026J23Rik	3-UTR	Y	21398283	21400414	1	54.1	24.2	53.3	33.7
chr11	Hoxb4	LastExon	Y	96181344	96182952	5	48.3	13.2	39.7	20.4
chr11	Hoxb5	Intron1	Y	96165490	96166203	2	67.6	39.5	57.3	38.5

chr16	Alg1	Intron1	Y	5233951	5235058	2	40.0	7.8	19.5	1.2
chr7	Lmtk3	Intron12	Y	53050932	53053568	4	24.2	7.0	22.7	5.0
chr11	Sfi1	Intron2	Y	3032331	3032889	4	72.6	55.6	72.4	54.8
chr12	6030440G07Rik	Intron1	Y	112232918	112251099	2	50.7	14.8	23.5	6.6
chr10	Zc3h12d	Intron3	Y	7573126	7582183	1	79.0	59.0	80.5	63.6
chr11	Kdm6b	Intron7	Y	69215166	69215260	1	66.1	36.0	63.7	47.1
chr14	Gjb2	Intron1	Y	57719609	57723305	6	33.7	8.3	23.2	7.1
chr1	Tor3a	Intron3	Y	158596909	158599501	2	67.5	36.8	56.1	40.1
chr11	Hoxb6	Intron1	Y	96160906	96161982	4	42.8	18.1	43.1	27.6
chr11	Hoxb7	LastExon	Y	96150691	96151477	2	71.5	30.2	54.4	39.1
chr8	Snai3	Intron2	Y	124980629	124984438	2	52.7	0.0	23.4	8.2
chr11	Mpo	Intron12	Y	87616252	87616899	1	43.4	26.7	45.0	30.2
chr7	Hirip3	Exon2	Y	134006332	134006440	1	25.0	8.3	21.4	6.8
chr11	Sfi1	Exon17	Y	3045244	3045325	1	50.4	32.7	49.4	34.9
chr11	Acsf2	Intron15	Y	94434543	94462956	2	36.4	11.1	21.2	7.0
chr7	Trpm4	Exon12	Y	52572376	52572505	1	85.9	69.1	86.6	72.6
chr2	Zfp385b	Intron7	Y	77341740	77557507	2	85.9	62.1	82.5	69.1
chr1	Gpa33	Intron1	Y	168060736	168076769	2	48.2	32.1	40.4	26.9
chr5	6030443J06Rik	Intron1	Y	22056291	22059576	3	96.9	47.0	62.7	49.4
chr8	Kcnk1	Intron1	Y	128519715	128548911	6	58.3	35.0	30.2	17.1
chr11	Hoxb6	LastExon	Y	96161983	96162883	3	53.3	26.6	49.4	36.6
chr19	Banf1	Intron1	Y	5365159	5365817	2	34.1	8.0	17.9	5.2
chr10	Btbd11	Intron3	Y	85024455	85061469	5	20.9	0.0	24.0	11.3
chr2	St6galnac6	Intron2	Y	32467924	32470300	2	100.0	49.7	65.1	52.7
chr6	Plxnd1	Intron35	Y	115930773	115943506	3	18.2	2.0	19.3	6.9
chr17	Neu1	Intron2	Y	35069054	35069510	1	29.3	10.6	32.8	20.4
chr11	Kdm6b	Exon5	Y	69214765	69214879	1	28.5	9.8	20.2	8.2
chr5	Pkd2	Intron3	Y	104906196	104907809	1	91.7	69.0	87.8	76.1
chr17	Srrm2	Intron1	Y	23940366	23945032	7	31.9	13.3	22.2	11.0
chr7	Zfp580	3-UTR	Y	5004938	5005325	2	20.7	2.2	24.6	13.5
chr11	Hoxb6	3-UTR	Y	96162243	96162883	2	63.9	34.2	50.2	39.1
chr10	Spatc1l	LastExon	Y	76032411	76032945	3	69.6	25.7	31.4	20.3
chr11	Sfi1	Exon27	Y	3077430	3077524	1	78.5	52.1	73.9	62.9
chr17	Wdr46	Exon3	Y	34078351	34078463	1	32.2	16.9	27.7	16.9
chr11	Gm12216	Intron3	Y	53663613	53672614	2	62.4	27.0	27.4	16.5
chr2	Pxmp4	Intron3	Y	154422001	154429252	1	18.6	0.5	13.4	2.8
chr2	Rin2	Exon8	Y	145685750	145686910	5	28.7	4.5	12.9	2.4
chr13	Cltb	Intron2	Y	54700195	54700404	1	26.8	2.3	12.0	1.7
chr10	Cpm	Intron2	Y	117066940	117096823	3	41.9	24.2	38.4	28.3
chr19	Pik3ap1	Intron16	Y	41450756	41459446	3	78.6	3.8	35.2	25.3
chr17	Clip4	Intron1	Y	72119251	72139207	8	66.8	35.8	34.2	24.4
chr18	Fam69c	Exon2	Y	84899422	84900081	3	28.0	6.3	47.7	38.1
chr15	Gramd4	3-UTR	Y	85965885	85968066	2	76.2	55.8	50.5	41.1
chr15	Gramd4	LastExon	Y	85965780	85968066	3	76.2	55.8	50.5	41.1
chr4	Foxd2	3-UTR	Y	114578885	114579947	2	20.0	4.3	10.5	1.4
chr12	Insm2	3-UTR	Y	56701942	56703004	2	54.1	37.2	41.5	32.4
chr19	E030044B06Rik	Intron1	Y	40942931	40950788	5	81.0	23.1	21.8	12.9
chr8	4931428F04Rik	Downstrea	Y	107803309	107804308	2	60.7	26.8	61.5	52.7
chr8	Nol3	Exon2	Y	107803316	107803675	2	60.7	26.8	61.5	52.7

chr13	Isl1	Intron2	Y	117091987	117093253	1	73.0	41.9	53.2	44.4
chr15	Lima1	Intron10	Y	99674255	99705763	4	35.9	7.3	29.5	20.8
chr2	Rapgef4	Intron1	Y	71819409	71869089	5	52.0	33.9	41.0	32.4
chr17	Atat1	Intron1	Y	36034861	36035201	2	57.8	29.6	25.9	17.3
chr13	Ero1lb	Intron1	Y	12658446	12667086	3	58.5	21.2	29.4	21.0
chr2	4930526D03Rik	3-UTR	Y	181431500	181431574	1	79.2	63.0	75.0	66.7
chr14	Dupd1	Intron2	Y	22506058	22522101	2	96.7	55.1	54.9	46.6
chr11	Abi3	Exon2	Y	95694954	95695114	1	23.5	5.2	18.2	10.0
chr6	Casc1	Intron7	Y	145134413	145139877	3	70.0	48.4	64.2	56.0
chr19	Sh3pxd2a	Intron7	Y	47361209	47388524	4	96.0	45.7	75.2	67.1
chr7	Lgals7	Downstrea	Y	29651304	29652303	2	46.6	20.0	53.1	45.0
chr17	Neu1	Intron4	Y	35071131	35071227	1	64.8	42.6	67.9	59.9
chr4	Pex14	Intron8	Y	148450177	148473880	5	76.9	22.5	28.0	20.5
chr2	Gm14164	Intron1	Y	152171463	152187534	2	90.6	70.0	72.0	64.4
chr5	Foxk1	Intron2	Y	142911281	142924640	3	39.0	13.8	26.1	18.7
chr2	Tmem210	Downstrea	Y	25144708	25145707	3	33.3	0.3	7.7	0.3
chr14	Shisa2	Intron1	Y	60245238	60248471	4	74.6	26.4	31.8	24.5
chr7	Setd1a	Exon17	Y	134942605	134942742	1	88.0	72.0	84.6	77.4
chr15	Tbc1d22a	Intron1	Y	86045067	86065159	3	25.6	5.1	10.4	3.2
chr7	Arrb1	Intron1	Y	106684309	106730770	4	24.4	4.5	15.5	8.4
chr8	Zfp276	Intron1	Y	125778824	125779606	3	56.3	39.4	22.0	14.9
chr1	Ppfia4	LastExon	Y	136193360	136195405	3	39.1	22.9	44.9	37.8
chr11	Slc5a10	Intron6	Y	61492159	61517752	8	41.2	12.1	13.6	6.8
chr9	Lamb2	LastExon	Y	108392615	108392861	2	29.2	12.4	18.1	11.5
chr7	Kcnk6	3-UTR	Y	30006947	30010054	1	18.4	0.0	18.0	11.5
chr7	Ube2m	Downstrea	Y	13619469	13620468	5	23.8	0.7	7.7	1.3
chr2	Dhrs9	Intron1	Y	69218689	69230908	2	33.2	9.6	16.1	9.7
chr18	St8sia5	LastExon	Y	77493105	77494189	3	61.8	41.0	42.6	36.3
chr17	Cdc42ep3	Intron1	Y	79735057	79754156	7	57.1	38.9	47.4	41.4
chr4	Zc3h12a	Intron5	Y	124804327	124805010	4	22.5	1.0	7.0	1.3
chr2	Pabpc1l	Intron1	Y	163851410	163853210	1	96.6	69.0	86.0	80.4
chr5	A930011G23Rik	Intron2	Y	99806195	99987910	3	62.0	36.9	49.4	44.0
chr1	Bend6	Intron6	Y	33940494	33964410	5	34.9	3.0	7.9	2.6
chr6	Gsg1	LastExon	Y	135187348	135187763	3	72.5	53.0	74.2	69.0
chr4	Gpr153	3-UTR	Y	151657699	151659446	3	57.1	30.4	31.8	26.6
chr1	Ppfia4	3-UTR	Y	136193360	136195654	4	39.1	22.9	34.0	28.8
chr19	Mlana	Intron2	Y	29774661	29779109	1	98.6	83.3	97.6	92.6
chr11	Hdac5	Intron25	Y	102079802	102086047	4	24.2	3.6	13.1	8.1
chr8	Gdf15	Intron1	Y	73154074	73155231	3	24.6	1.7	13.0	8.1
chr4	Yrdc	Intron2	Y	124529052	124529143	1	32.5	16.1	29.1	24.3
chr4	Mxra8	Intron1	Y	155214000	155214886	1	25.9	9.5	18.2	13.3
chr19	Pitx3	Exon2	Y	46211855	46211984	2	58.3	18.8	16.7	11.8
chr2	H13	Intron4	Y	152511923	152514398	3	69.0	52.2	58.2	53.5
chr7	Gng8	Intron1	Y	17477706	17477798	1	35.8	12.5	24.7	20.0
chr15	Naprt1	Intron12	Y	75724278	75724446	2	21.2	0.5	9.6	5.0
chr15	Puf60	LastExon	Y	75900612	75901057	2	85.3	69.5	74.7	70.4
chr1	Ctdsp1	Intron5	Y	74441307	74441410	2	87.1	66.5	83.4	79.2
chr11	1810043H04Rik	Intron2	Y	119960573	119961487	4	29.0	6.4	8.9	4.7
chr9	1810026J23Rik	LastExon	Y	21397594	21400414	6	27.7	12.4	11.6	7.5

chr5	Fbxl13	Intron14	Y	21091112	21120467	2	72.7	51.6	72.1	68.0
chr1	4933424G06Rik	Intron8	Y	36803417	36814634	2	58.5	24.2	56.6	52.6
chr6	Ccdc142	3-UTR	Y	83058372	83059115	4	25.0	5.3	10.0	6.0
chr6	Ccdc142	LastExon	Y	83058085	83059115	4	25.0	5.3	10.0	6.0
chr1	Mir26b	Downstrea	Y	74440969	74441968	4	87.1	66.5	85.6	81.7
chr7	Tarsl2	Exon11	Y	72822882	72823044	1	77.0	60.9	64.1	60.3
chr9	Gramd1b	Intron18	Y	40141241	40220881	3	51.6	34.3	51.2	47.4
chr11	A030009H04Rik	3-UTR	Y	69154661	69156149	2	87.8	64.3	62.8	59.2
chr19	1700092M07Rik	LastExon	Y	8815465	8815715	2	28.7	11.1	13.0	9.4
chr11	Krt222	Intron4	Y	99100069	99101806	1	42.0	25.5	35.2	31.9
chr7	Zfp580	LastExon	Y	5004407	5005325	6	20.7	2.2	10.8	7.5
chr8	Pgbd5	Intron6	Y	126908523	126957698	4	74.8	29.2	27.5	24.3
chr7	Efcab4a	Intron1	Y	148647089	148649282	7	20.5	5.5	4.4	1.2
chr19	Arl2	Intron4	Y	6137841	6141036	4	40.2	24.5	12.7	9.6
chr2	Rnf114	Intron3	Y	167329102	167332546	5	61.5	21.8	11.0	7.9
chr11	Osbp2	Intron12	Y	3626579	3719839	13	48.5	27.6	14.1	11.1
chr7	Ric8	Intron2	Y	148043792	148043880	1	21.7	0.0	5.0	1.9
chr1	Faim3	Intron5	Y	132774482	132774759	1	42.9	22.2	27.0	24.0
chr14	Saysd1	Intron1	Y	20896867	20902099	2	19.0	3.3	5.2	2.2
chr1	Myeov2	Intron2	Y	94536337	94538456	4	28.2	5.2	7.0	4.2
chr15	Sybu	Intron7	Y	44579945	44619134	6	69.2	38.1	23.8	21.3
chr13	Kdm1b	Intron1	Y	47139040	47143271	6	16.5	0.0	4.3	1.9
chr2	1700101E01Rik	Intron5	Y	28841422	28910333	5	40.7	8.2	8.7	6.3
chr10	Lrp11	Intron1	Y	7310412	7315901	2	17.9	1.6	3.5	1.1
chr15	1700001L05Rik	Intron3	Y	83195941	83197419	2	45.3	27.5	26.4	24.0
chr6	Kcna1	LastExon	Y	126586481	126593904	10	52.2	36.8	26.0	23.7
chr4	Yrdc	Exon2	Y	124529144	124529263	1	53.6	28.9	31.1	28.8
chr19	Yif1a	Intron1	Y	5088749	5089418	2	23.9	5.4	10.5	8.3
chr3	Sema6c	3-UTR	Y	94977211	94977972	4	27.1	11.5	9.9	7.8
chr14	Gja3	LastExon	Y	57653297	57655767	5	92.9	77.1	76.7	74.8
chr10	Pip5k1c	3-UTR	Y	80780514	80782719	2	25.1	7.8	9.1	7.2
chr10	Pip5k1c	LastExon	Y	80780511	80782719	2	25.1	7.8	9.1	7.2
chr11	Sdk2	Intron44	Y	113804593	113927116	7	85.3	54.8	31.9	30.0
chr4	Gpr153	LastExon	Y	151656967	151659446	7	57.1	15.2	16.1	14.2
chr2	Map1a	Exon4	Y	121125005	121133108	2	94.6	78.8	83.7	82.0
chr12	Bcap29	Intron6	Y	32315759	32319127	3	82.5	61.7	59.6	58.0
chr10	Txnrd1	Intron1	Y	82296777	82335682	12	54.4	29.8	24.9	23.4
chr7	Rsph6a	Exon2	Y	19650656	19651420	4	28.3	9.7	27.8	26.3
chr5	Caln1	Intron1	Y	130845505	130890626	3	96.1	77.4	90.7	89.2
chr1	Hs6st1	Intron1	Y	36126030	36160357	4	90.4	0.0	24.0	22.5
chr9	Dock3	Intron52	Y	107081496	107133802	6	83.3	66.7	70.6	69.1
chr7	Scn1b	Intron5	Y	31910241	31911684	5	21.5	4.7	17.4	15.9
chr11	Flt4	Intron14	Y	49448298	49448394	1	35.0	17.2	33.6	32.2
chr3	Slitrk3	Downstrea	Y	72851047	72852046	2	78.3	58.6	73.7	72.5
chr2	1700003F12Rik	Downstrea	Y	154375785	154376784	2	87.5	55.4	61.3	60.1
chr11	Rgs9	Intron18	Y	109142860	109159251	6	85.4	67.4	69.6	68.6
chr6	1700003E16Rik	Intron1	Y	83106585	83110905	2	23.5	7.7	12.0	10.9
chr6	Il17re	LastExon	Y	113419551	113420745	4	62.0	46.2	34.3	33.2
chr13	Gfod1	LastExon	Y	43290888	43296613	6	87.9	63.7	72.8	71.9

chr14	Ltb4r2	LastExon	Y	56380745	56382066	7	57.5	39.4	11.3	10.3
chr11	Skap1	Intron4	Y	96402801	96563922	4	87.3	67.2	91.2	90.3
chr7	Gys1	Intron3	Y	52693664	52694848	2	71.9	56.8	70.7	69.9
chr10	Dapk3	Intron7	Y	80654657	80655041	2	97.6	78.7	65.4	64.6
chr7	Zfp768	LastExon	Y	134486309	134488389	6	68.8	53.1	86.9	86.1
chr10	Eef2	Intron2	Y	80640625	80640797	2	81.3	41.9	44.5	43.7
chr11	Tbc1d16	Intron11	Y	119072055	119089709	8	93.0	42.7	26.6	25.9
chr17	Wdr46	Intron4	Y	34078464	34078577	1	69.4	51.3	55.2	54.7
chr17	Guca1b	Intron1	Y	47522646	47526058	1	66.3	50.5	49.5	49.1
chr1	Rgs20	Intron5	Y	5011119	5060090	6	27.3	9.3	11.5	11.2
chr7	Kcnk6	Intron2	Y	30010887	30017180	2	33.7	8.9	25.5	25.2
chr8	Mfap3l	Intron1	Y	63111856	63135256	6	93.3	62.3	30.9	30.7
chr15	Zfp385a	LastExon	Y	103144326	103145656	6	38.9	21.0	32.1	32.0
chr13	Arhgef28	Intron35	Y	98915551	98976027	5	83.9	38.9	33.0	32.9
chr17	Cyp4f40	Intron11	Y	32812932	32813247	1	75.9	54.9	67.2	67.1
chr7	Zfp771	Intron2	Y	134388649	134397318	3	86.3	40.4	37.8	37.9
chr8	Atmin	Intron1	Y	119467651	119476773	4	19.6	3.9	0.2	0.3
chr2	Chrm4	LastExon	Y	91767374	91769986	6	70.4	40.3	51.4	51.6
chr18	Synpo	LastExon	Y	60753644	60756142	5	27.5	2.4	19.2	19.4
chr10	Eef2	Downstrea	Y	80645255	80646254	5	49.8	9.6	8.2	8.4
chr10	E030030I06Rik	Intron1	Y	21834155	21868294	7	63.2	30.9	35.8	36.1
chr8	Upf3a	Intron2	Y	13786254	13787364	2	61.4	2.5	2.0	2.3
chr7	Dhx32	Intron11	Y	140951597	140968284	7	93.3	62.2	31.8	32.2
chr6	Wnt5b	Intron4	Y	119398291	119493998	22	21.7	4.8	3.0	3.5
chr8	Cln8	Intron1	Y	14889119	14894571	5	62.6	1.3	38.0	38.5
chr2	Kcnb1	Intron1	Y	166931860	167013556	3	88.6	32.6	31.9	32.5
chr11	Trpv3	Intron5	Y	73092573	73093263	2	52.3	17.1	47.2	47.8
chr17	Cyp4f40	Downstrea	Y	32813426	32814425	1	16.1	0.0	1.0	1.6
chr5	Rell1	Intron6	Y	64329273	64359884	4	47.5	0.0	46.5	47.1
chr13	Elmo1	Intron1	Y	20182641	20277222	7	87.6	48.6	16.1	16.7
chr7	Irgq	Intron2	Y	25316935	25318284	2	91.6	50.6	48.5	49.1
chr8	Shcbp1	Intron12	Y	4768312	4779420	4	86.9	45.6	45.1	45.7
chr13	Fam65b	Intron16	Y	24802161	24805490	1	33.3	11.9	11.1	11.8
chr11	Rab40b	Intron5	Y	121224879	121249311	3	53.6	30.0	14.9	15.7
chr10	Gng7	Intron3	Y	80460790	80477600	8	31.6	14.3	29.7	30.5
chr1	Rims1	Intron31	Y	22733850	22812399	2	55.3	37.5	35.8	36.6
chr9	Abcg4	Intron13	Y	44090005	44095298	1	38.6	19.5	32.5	33.3
chr8	Rnf166	Intron5	Y	124994257	124999762	5	17.7	2.6	3.0	3.9
chr10	D630013N20Rik	Downstrea	Y	70062797	70063796	3	44.0	21.7	0.6	1.6
chr8	Eps15l1	Intron23	Y	74924133	74945303	4	20.0	3.4	0.9	1.8
chr13	Acot12	Intron1	Y	91881266	91889792	3	20.0	3.3	13.8	14.7
chr2	Vstm2l	Intron3	Y	157762978	157769592	6	31.0	6.0	3.8	4.7
chr1	Sctr	Intron1	Y	121903909	121918734	3	41.6	5.6	30.5	31.5
chr18	Pdgfrb	Intron6	Y	61224834	61225368	1	30.3	11.0	15.4	16.4
chr18	Nrg2	Intron9	Y	36212606	36356081	12	30.0	4.3	16.8	17.8
chr3	Sema6c	LastExon	Y	94976177	94977972	9	27.1	11.5	15.1	16.5
chr5	Rgs12	Intron1	Y	35292174	35307421	4	37.2	2.4	1.5	2.9
chr7	Tsku	Intron1	Y	105501641	105509787	8	40.4	18.8	6.3	7.7
chr8	Cacna1a	Intron19	Y	87083590	87089846	3	67.2	45.1	40.8	42.2

chr15	Zfp623	Intron1	Y	75771590	75777552	4	22.9	1.3	4.5	6.0
chr14	Synpo2l	LastExon	Y	21478640	21481687	6	41.8	15.8	8.5	10.0
chr2	Dok5	Intron1	Y	170557689	170626349	7	94.2	69.5	69.0	70.6
chr16	Hmox2	Intron1	Y	4726472	4762558	7	24.4	1.2	3.8	5.6
chr5	Tchp	Intron10	Y	115168470	115169587	2	84.2	67.8	79.7	81.6
chr17	Dusp1	Intron2	Y	26644150	26644518	2	19.6	2.1	17.4	19.3
chr1	Syt2	LastExon	Y	136644052	136645994	2	57.8	40.7	33.8	35.9
chr10	Ptprb	Intron1	Y	115738736	115739163	2	23.8	8.3	18.9	21.0
chr5	Mxd4	Intron4	Y	34526993	34529977	4	46.4	26.6	13.8	15.9
chr10	Inhbe	LastExon	Y	126786458	126788067	3	24.3	6.5	11.7	13.9
chr7	Spns1	Exon10	Y	133520188	133520253	1	66.2	48.7	54.9	57.1
chr2	Myh7b	Intron29	Y	155455417	155455993	3	100.0	61.9	56.0	58.6
chr1	Kcnh1	Intron1	Y	194014969	194045214	3	26.5	9.1	6.9	9.5
chr16	Rtn4r	Intron1	Y	18127996	18150824	5	62.2	34.5	20.3	23.0
chr14	Sall2	LastExon	Y	52930852	52935338	3	92.7	63.3	62.2	65.0
chr13	Shc3	Intron11	Y	51611965	51662339	3	56.5	38.8	16.0	18.9
chr2	Myl9	LastExon	Y	156606726	156607393	2	72.9	38.7	50.3	53.3
chr19	Dagla	Intron19	Y	10346122	10379310	6	63.2	40.4	15.7	18.6
chr13	Rnf144b	Intron1	Y	47218243	47302728	9	85.6	36.6	19.8	22.8
chr13	Gprin1	Intron2	Y	54843470	54850930	5	64.6	37.2	32.5	35.6
chr7	Adck4	LastExon	Y	28042242	28042968	3	30.8	14.8	40.2	43.5
chr10	Ppm1h	Intron1	Y	122116487	122219238	9	45.5	19.0	41.4	44.7
chr18	Cxxc5	Intron1	Y	35989641	36018046	19	25.1	6.9	16.8	20.2
chr7	Gm2115	Intron1	Y	91677700	91724214	2	33.3	12.0	13.6	17.1
chr15	Srebf2	Intron3	Y	82001714	82003182	1	44.4	24.2	43.7	47.2
chr4	Heyl	Intron1	Y	122911190	122917373	8	80.6	46.9	15.5	19.1
chr1	Rassf5	Intron4	Y	133078832	133108833	2	24.3	9.2	20.7	24.3
chr7	Ifitm10	Exon2	Y	149556763	149557215	2	95.1	59.6	68.1	71.8
chr4	Gm13032	Intron2	Y	140370183	140372610	3	28.0	7.6	9.4	13.1
chr4	Prdm16	Intron6	Y	153708562	153709201	3	73.0	57.9	52.1	55.9
chr4	Tas1r1	LastExon	Y	151402023	151403106	3	29.3	13.1	8.1	12.1
chr17	Tbc1d24	Intron9	Y	24326999	24342418	4	31.6	7.5	13.6	17.6
chr7	Rtn2	LastExon	Y	19881065	19881513	3	56.5	0.4	11.1	15.2
chr7	Gpr123	Intron7	Y	147059957	147060865	1	68.2	50.0	50.6	54.7
chr2	Epb4.1l1	Intron1	Y	156247036	156319639	13	66.8	48.3	14.9	19.3
chr8	Pkn1	Downstrea	Y	86192661	86193660	4	61.1	43.3	54.8	59.3
chr11	Rara	Intron1	Y	98799194	98811558	6	67.4	49.6	19.6	24.1
chr13	Habp4	Intron1	Y	64263643	64271321	6	62.1	19.5	36.2	40.7
chr5	Smarcd3	Downstrea	Y	24097440	24098439	2	70.2	53.3	49.0	53.5
chr5	Chst12	Intron1	Y	140981651	140999494	5	44.8	28.6	18.3	22.9
chr16	Thpo	Intron5	Y	20729291	20734334	13	61.5	46.1	26.5	31.3
chr4	Bach2	Intron1	Y	32504559	32588587	2	46.3	28.6	26.6	31.8
chr7	Slco3a1	Intron10	Y	81649529	81699296	4	49.2	30.2	22.7	28.0
chr5	Igfbp7	Intron5	Y	77830605	77836564	1	38.9	23.7	31.4	37.0
chr7	Zfp128	Intron1	Y	13466710	13469991	2	28.6	2.0	13.7	19.4
chr1	Mroh2a	Exon33	Y	90151665	90151868	1	90.9	75.8	84.8	90.6
chr17	Neu1	Exon3	Y	35070948	35071130	1	70.2	54.0	59.6	65.6
chr11	Wscd1	Intron1	Y	71564328	71573079	6	26.9	2.8	4.0	10.4
chr8	Gdf1	LastExon	Y	72854598	72855491	7	65.5	34.8	64.2	70.7

chr1	Fcrlb	LastExon	Y	172837404	172837834	2	30.2	7.3	13.4	20.2
chr8	Rln3	Intron1	Y	86567231	86568668	1	20.0	4.3	13.2	20.4
chr10	Nfic	Intron8	Y	80883544	80889820	6	85.1	41.7	29.1	36.4
chr10	Jsrp1	Intron4	Y	80273312	80274811	1	65.3	49.0	42.7	50.0
chr7	Mypop	Intron2	Y	19577970	19585839	2	80.4	50.9	39.5	47.0
chr15	Zfat	Intron15	Y	68056506	68090229	4	21.9	1.4	10.7	18.2
chr8	Upf1	Downstre	Y	72854421	72855420	5	65.5	34.8	59.9	67.4
chr14	Sorbs3	Intron20	Y	70603224	70607330	6	44.6	18.7	16.0	23.4
chr9	Tpbg	LastExon	Y	85737258	85740662	9	91.8	55.7	31.1	38.6
chr10	Anks1b	Intron24	Y	90429138	90432548	4	56.8	25.0	10.4	17.9
chr17	Memo1	Intron8	Y	74657846	74694031	6	87.2	62.0	30.1	37.7
chr15	Gramd4	Intron1	Y	85888262	85921742	10	17.5	2.5	0.8	8.5
chr11	Grb10	Intron16	Y	11887678	11937194	14	66.6	45.1	42.8	50.8
chr11	Gucy2e	Intron9	Y	69041139	69041544	1	45.0	25.8	39.4	47.5
chr6	Gm5577	Intron1	Y	87931879	87932953	3	27.3	11.9	5.0	13.3
chr5	Kcnh2	Intron14	Y	23854360	23857025	5	72.1	48.4	24.7	33.1
chr4	Slc6a9	Exon12	Y	117540364	117540534	1	57.6	34.8	43.4	51.9
chr16	Gp5	3-UTR	Y	30307771	30308236	1	85.2	57.1	62.7	71.4
chr17	Tulp1	Intron10	Y	28499937	28500630	2	60.1	43.1	30.1	38.8
chr15	Pdgfb	Intron6	Y	79835996	79844365	11	77.0	46.4	10.0	18.9
chr10	Txnrd1	Intron2	Y	82323376	82337357	5	90.4	66.6	54.4	63.8
chr8	Use1	Intron5	Y	73892011	73892448	1	85.0	68.8	73.1	82.6
chr2	Mapk8ip1	Intron11	Y	92231299	92241185	7	68.2	3.6	8.9	18.5
chr18	Lims2	Intron1	Y	32091205	32101393	7	95.6	65.1	46.0	55.9
chr12	Begain	LastExon	Y	110270392	110272561	8	86.4	54.9	53.3	63.2
chr11	Ccdc40	Intron13	Y	119113331	119114563	1	33.3	20.8	52.2	46.4
chr8	Plekha2	Intron9	Y	26174459	26182470	2	24.1	12.1	51.7	14.4
chr8	Abhd8	Intron1	Y	73981277	73981936	2	8.9	0.0	47.7	0.0
chr9	Ap1m2	Intron10	Y	21114237	21116650	2	0.6	0.3	44.0	0.0
chr18	Zfp608	Intron8	Y	55106463	55147264	4	0.0	0.0	38.2	0.0
chr15	Smpd5	Intron1	Y	76125722	76125819	1	40.0	41.9	80.6	46.5
chr5	Steap4	Intron1	Y	7960542	7975438	3	0.0	0.4	33.6	1.0
chr7	Fam71e1	Intron4	Y	51755809	51756337	1	34.1	38.1	57.1	28.6
chr4	Fndc5	Intron1	Y	128814408	128815934	3	13.2	23.8	31.7	5.2
chr11	Rilp	Intron4	Y	75324510	75324790	1	27.3	34.9	53.3	28.8
chr7	B9d2	3-UTR	Y	26471314	26471577	2	25.4	32.2	51.8	28.1
chr5	Diablo	Exon4	Y	123973264	123973390	1	25.0	35.1	47.1	26.3
chr2	Kcnk15	Intron1	Y	163679826	163683918	3	9.3	6.9	25.0	5.1
chr16	Ildr1	Intron2	Y	36708507	36709564	1	61.1	58.2	77.0	58.7
chr7	Mamstr	Exon8	Y	52899889	52899943	1	6.0	0.0	21.5	3.7
chr8	Zswim4	Downstre	Y	86733841	86734840	4	3.0	17.4	37.2	20.0
chr2	Slc12a5	Exon24	Y	164822610	164822743	1	17.8	25.2	36.0	19.2
chr13	Nxnl2	Intron1	Y	51266994	51269993	2	3.8	2.7	20.6	3.9
chr5	Mtus2	Intron9	Y	149100665	149107058	1	59.4	69.2	79.1	63.4
chr2	Pigu	Downstre	Y	155102988	155103987	2	24.8	31.2	54.4	39.1
chr2	Map1lc3a	Intron3	Y	155102941	155103210	3	12.4	15.6	36.3	21.0
chr10	Amh	Downstre	Y	80270394	80271393	5	8.0	11.6	23.5	8.3
chr10	Jsrp1	Downstre	Y	80270241	80271240	5	8.0	11.6	23.5	8.3

Table S2. Lists of non-CGI mDMR genes

Group 5-mDMR genes associated with methylation gain at 5' non-CGI

							DNA Methylation Level (%)			
							ISCs		Diff. Cells	
**Chr**	**Gene**	**Region**	**CGI**	**Start**	**End**	**#Bins**	**P0**	**P21**	**P0**	**P21**
chr11	Snf8	Upstream	N	95895231	95896230	3	36.8	68.1	48.9	97.2
chr6	Lyrm5	Upstream	N	145158654	145159653	2	22.6	77.6	5.3	53.0
chr17	Zfp13	Upstream	N	23736455	23737454	5	0.0	38.2	6.3	52.0
chr15	Cpq	Upstream	N	33011884	33012883	5	51.4	95.5	5.1	49.8
chr19	Slc22a20	Upstream	N	5986144	5987143	5	39.1	86.8	44.3	88.8
chr3	Sv2a	Upstream	N	95984150	95985149	4	54.0	72.5	42.5	87.0
chr5	Pla2g1b	Upstream	N	115915275	115916274	4	2.5	47.9	46.3	90.6
chr17	T2	Upstream	N	8564261	8565260	5	40.2	55.7	13.2	57.4
chr18	Slc39a6	Upstream	N	24762319	24763318	2	10.5	44.6	14.7	58.8
chr12	Myt1l	Upstream	N	30212249	30213248	5	29.0	57.1	31.2	74.4
chr17	C2	Upstream	N	35019046	35020045	5	32.5	56.5	4.7	47.3
chr12	Matn3	Upstream	N	8953735	8954734	3	9.6	71.9	33.1	75.2
chr4	C8b	Upstream	N	104437922	104438921	5	20.7	89.4	49.3	91.4
chr10	Trappc3l	Upstream	N	33756403	33757402	5	63.6	80.2	43.5	84.2
chr5	Pex1	Upstream	N	3595066	3596065	3	3.0	64.9	4.0	44.5
chr8	Arl2bp	Upstream	N	97189655	97190654	3	3.8	39.1	5.7	44.2
chr9	1110036E04Rik	Upstream	N	63896636	63897635	5	22.9	64.5	24.1	62.2
chr1	Tmem14a	Upstream	N	21207712	21208711	5	58.7	77.6	34.8	72.7
chr6	Tmem168	Upstream	N	13558064	13559063	2	17.0	64.6	19.9	56.3
chr9	Med17	Upstream	N	15084312	15085311	2	1.1	41.7	29.7	65.5
chr17	Lix1	Upstream	N	17538650	17539649	5	14.8	37.0	27.3	63.0
chr2	Otor	Upstream	N	142903228	142904227	5	54.5	73.3	26.7	62.3
chr8	Zfhx3	Upstream	N	111237544	111238543	3	22.0	63.3	21.3	56.8
chr9	Igdcc3	Upstream	N	64987996	64988995	2	22.0	45.8	27.9	62.7
chr4	Susd1	Upstream	N	59451506	59452505	4	28.3	49.2	15.2	49.6
chr9	Gramd1b	Upstream	N	40263350	40264349	1	39.7	71.5	40.8	75.2
chr15	Krt83	Upstream	N	101269236	101270235	3	45.0	84.9	41.7	75.9
chr5	Sds	Upstream	N	120925556	120926555	5	10.0	42.5	14.0	47.9
chr18	Snora74a	Upstream	N	35715684	35716683	5	46.5	68.8	27.4	60.7
chr10	Rab21	Upstream	N	114752648	114753647	2	74.2	92.9	55.4	88.5
chr11	Sat2	Upstream	N	69434611	69435610	5	3.6	27.1	4.1	36.5
chr12	Pfn4	Upstream	N	4775101	4776100	2	42.5	90.5	63.3	95.7
chr6	Mrps35	Upstream	N	146990292	146991291	4	30.8	69.0	56.0	88.2
chr5	Cad	Upstream	N	31356184	31357183	2	52.6	87.1	54.6	86.7
chr14	Sap18	Upstream	N	58416026	58417025	3	16.4	45.6	19.6	51.5
chr11	Abr	Upstream	N	76391230	76392229	5	16.9	41.9	15.3	47.1
chr10	Rmst	Upstream	N	91627924	91628923	5	25.5	55.2	19.1	50.3
chr1	4930487H11Rik	Upstream	N	62841307	62842306	4	25.2	56.8	24.3	54.6
chr3	Fabp12	Upstream	N	10301184	10302183	5	70.5	88.2	56.7	86.9
chr7	Aldh1a3	Upstream	N	73572364	73573363	1	54.0	72.1	44.5	74.6
chr2	Gm757	Upstream	N	25092308	25093307	5	39.2	58.5	44.0	74.1
chr6	St3gal5	Upstream	N	72046607	72047606	2	40.6	76.2	43.4	73.1
chr9	Lztfl1	Upstream	N	123626676	123627675	2	18.2	51.3	21.4	50.7
chr10	Tle2	Upstream	N	81037032	81038031	4	17.1	64.2	48.8	78.2

chr5	Mmd2	Upstream	N	143084707	143085706	3	56.8	85.4	56.2	85.4
chr11	Tbcd	Upstream	N	121312263	121313262	3	8.2	53.8	26.7	55.8
chr11	Plscr3	Upstream	N	69658874	69659873	3	27.3	50.8	29.4	58.1
chr6	Slc6a6	Upstream	N	91633061	91634060	2	35.0	59.2	42.5	71.0
chr15	Nfam1	Upstream	N	82863828	82864827	5	36.5	66.0	25.7	54.2
chr1	Inha	Upstream	N	75502652	75503651	2	59.2	76.7	48.7	77.1
chr6	Hoxa3	Upstream	N	52163067	52164066	3	16.9	56.8	5.4	33.7
chr10	Itga7	Upstream	N	128369869	128370868	4	39.1	70.3	43.2	71.5
chr19	Golga7b	Upstream	N	42321068	42322067	5	11.6	67.1	23.7	51.9
chr13	1110007C09Rik	Upstream	N	49311396	49312395	3	42.0	75.3	60.2	88.2
chr2	Snph	Upstream	N	151458270	151459269	3	64.0	87.2	60.2	88.0
chr18	Kcng2	Upstream	N	80560994	80561993	5	61.9	77.2	52.4	79.9
chr12	Prox2	Upstream	N	86447382	86448381	5	31.9	59.3	33.5	60.4
chr18	Ctif	Upstream	N	75857351	75858350	2	24.5	55.6	21.1	47.9
chr10	Tnfaip3	Upstream	N	18735217	18736216	3	13.1	29.5	13.1	39.7
chr17	Smoc2	Upstream	N	14415513	14416512	2	7.4	25.8	13.9	40.3
chr17	1600014C23Rik	Upstream	N	45870794	45871793	2	65.2	84.7	57.6	84.0
chr10	Rap1b	Upstream	N	117283031	117284030	2	67.5	83.3	59.5	85.4
chr17	Ly6g6d	Upstream	N	35211410	35212409	5	50.0	95.0	62.1	88.0
chr1	Gm216	Upstream	N	74407682	74408681	5	47.1	91.1	61.8	87.6
chr3	Clca3	Upstream	N	144695741	144696740	5	31.8	52.1	38.1	63.5
chr13	Cplx2	Upstream	N	54471713	54472712	4	7.9	57.4	25.4	50.7
chr12	Plekhh1	Upstream	N	80129150	80130149	2	4.5	37.0	14.3	39.0
chr4	Enho	Upstream	N	41587336	41588335	2	37.9	54.2	34.4	59.1
chr18	Wnt8a	Upstream	N	34700982	34701981	5	33.0	49.8	32.0	56.5
chr18	Phax	Upstream	N	56721223	56722222	2	21.7	57.7	27.9	52.1
chr18	A830052D11Rik	Upstream	N	32537939	32538938	5	5.0	57.7	36.8	60.9
chr1	Vangl2	Upstream	N	173957400	173958399	3	27.9	53.3	39.4	63.5
chr14	Gjb2	Upstream	N	57723540	57724539	2	17.0	38.2	34.2	58.2
chr3	Spag17	Upstream	N	99688340	99689339	5	2.7	29.4	4.1	28.0
chr18	Ticam2	Upstream	N	46734188	46735187	4	34.6	66.3	37.9	61.5
chr11	Kcnip1	Upstream	N	33893194	33894193	5	43.9	60.9	35.5	59.0
chr5	Gpr81	Upstream	N	124330030	124331029	5	61.0	77.9	48.1	71.5
chr16	Gp1bb	Upstream	N	18622497	18623496	3	66.3	89.1	63.8	87.2
chr7	Gm7092	Upstream	N	26400912	26401911	5	39.5	61.2	65.5	88.8
chr17	Angptl4	Upstream	N	33918521	33919520	3	48.4	85.7	70.8	94.0
chr17	Mocs1	Upstream	N	49566689	49567688	4	61.5	96.9	43.4	66.6
chr6	Il5ra	Upstream	N	106699032	106700031	5	29.5	53.7	39.4	62.5
chr13	Pitx1	Upstream	N	55932787	55933786	1	64.0	85.7	51.9	75.0
chr16	Bach1	Upstream	N	87698199	87699198	2	36.0	64.3	31.4	54.3
chr4	Zfp37	Upstream	N	61869581	61870580	3	8.2	33.3	15.6	38.5
chr9	Dyx1c1	Upstream	N	72805592	72806591	3	20.3	71.9	46.7	69.4
chr1	Capn8	Upstream	N	184494138	184495137	5	25.6	57.7	52.0	74.6
chr19	Loxl4	Upstream	N	42687297	42688296	2	37.1	52.8	36.0	58.5
chr4	Heyl	Upstream	N	122909799	122910798	4	58.5	78.3	45.9	68.1
chr7	Mapk3	Upstream	N	133902140	133903139	2	63.2	100.0	58.4	80.7
chr5	Zfand2a	Upstream	N	139960446	139961445	3	75.9	94.6	71.1	93.3
chr2	Wfdc13	Upstream	N	164509607	164510606	5	65.8	82.6	63.8	85.9
chr9	2310014F07Rik	Upstream	N	50312911	50313910	5	16.3	37.4	18.2	40.2

chr13	Rnf182	Upstream	N	43710166	43711165	4	27.6	47.9	31.4	53.3
chr2	5430417L22Rik	Upstream	N	118570498	118571497	1	11.9	32.2	13.0	34.7
chr5	Pomp	Upstream	N	148671204	148672203	3	3.4	45.4	14.4	36.1
chr6	Clstn3	Upstream	N	124414803	124415802	5	29.6	86.3	22.0	43.6
chr9	Gm10684	Upstream	N	44943690	44944689	5	32.5	57.8	36.3	57.6
chr14	Mir3075	Upstream	N	26352925	26353924	5	41.4	59.0	56.8	78.1
chr2	Clic3	Upstream	N	25311363	25312362	5	26.9	64.0	30.1	51.3
chr4	Pdzk1ip1	Upstream	N	114760313	114761312	5	41.2	67.8	42.9	64.0
chr5	Nyap1	Upstream	N	138181227	138182226	4	39.8	69.5	38.1	59.2
chr8	Pde4c	Upstream	N	73246963	73247962	5	56.1	79.1	50.2	71.3
chr4	Cdh17	Upstream	N	11684304	11685303	5	13.3	36.3	44.2	65.1
chr18	Zfp532	Upstream	N	65738884	65739883	5	55.2	73.6	61.1	82.0
chr3	Cd160	Upstream	N	96633275	96634274	5	65.8	83.6	51.1	71.6
chr17	Gm14873	Upstream	N	47838946	47839945	5	76.9	92.7	61.6	82.1
chr2	Zfp133-ps	Upstream	N	144284016	144285015	3	36.8	63.8	69.5	89.9
chr9	Col7a1	Upstream	N	108854790	108855789	5	26.8	75.7	28.7	49.0
chr11	St6galnac1	Upstream	N	116636822	116637821	5	61.6	79.2	48.0	68.1
chr13	Gm11346	Upstream	N	24696637	24697636	3	32.0	66.9	48.7	68.7
chr3	Nudt17	Upstream	N	96512484	96513483	4	72.0	100.0	80.0	100.0
chr3	Fgg	Upstream	N	82810818	82811817	5	23.7	48.1	30.1	50.1
chr3	Arhgef2	Upstream	N	88419129	88420128	5	32.0	76.9	50.5	70.5
chr3	Lmo4	Upstream	N	143868220	143869219	1	18.4	42.9	10.7	30.6
chr4	Gm11413	Upstream	N	83036573	83037572	5	51.3	76.9	53.8	73.7
chr16	Gm16863	Upstream	N	21296946	21297945	5	57.1	79.7	52.5	72.4
chr2	Mga	Upstream	N	119721964	119722963	3	49.3	67.2	42.0	61.8
chr1	Itpkb	Upstream	N	182259607	182260606	5	16.8	62.6	41.0	60.7
chr11	Tmem88	Upstream	N	69211737	69212736	4	33.3	60.3	42.5	62.2
chr7	Ttll13	Upstream	N	87390262	87391261	3	35.0	100.0	24.5	44.1
chr14	Adam2	Upstream	N	66696571	66697570	5	69.9	85.2	59.8	79.3
chr9	Tipin	Upstream	N	64128414	64129413	3	41.6	63.6	46.6	66.1
chr18	4930426D05Rik	Upstream	N	21809066	21810065	4	13.4	57.4	34.5	54.0
chr6	Eif4e3	Upstream	N	99616766	99617765	2	56.0	73.6	51.8	71.3
chr16	Zdhhc8	Upstream	N	18235230	18236229	1	31.7	53.6	24.9	44.2
chr7	Olfr681	Upstream	N	112268973	112269972	5	55.7	95.5	77.8	97.0
chr1	Kdm5b	Upstream	N	136455755	136456754	3	64.0	81.0	62.1	81.2
chr9	Cln6	Upstream	N	62685594	62686593	3	13.2	43.8	18.2	37.2
chr10	Ikzf4	Upstream	N	128083050	128084049	3	30.8	46.7	30.9	49.9
chr15	Ank	Upstream	N	27395432	27396431	1	32.9	55.1	35.6	54.6
chr7	Insc	Upstream	N	121888280	121889279	4	34.6	76.7	41.0	59.9
chr12	F730035M05Rik	Upstream	N	71327828	71328827	1	21.0	46.8	13.4	32.3
chr9	Ppp2r1b	Upstream	N	50664040	50665039	2	64.4	86.0	66.1	84.9
chr8	Nanos3	Upstream	N	86700452	86701451	5	69.2	87.3	64.7	83.5
chr1	Sec16b	Upstream	N	159435927	159436926	5	72.4	92.0	61.3	80.0
chr7	Car11	Upstream	N	52954337	52955336	2	60.0	90.1	64.2	82.8
chr2	Lrrn4	Upstream	N	132706599	132707598	5	23.8	46.2	22.6	41.2
chr9	Ooep	Upstream	N	78226396	78227395	5	62.6	85.2	64.5	83.0
chr1	Fzd7	Upstream	N	59537991	59538990	1	7.4	28.8	9.9	28.4
chr3	Synpo2	Upstream	N	122939068	122940067	3	13.5	40.3	24.0	42.4
chr18	Osbpl1a	Upstream	N	13100351	13101350	4	45.3	77.0	56.3	74.7

chr10	Gdf11	Upstream	N	128328775	128329774	1	36.9	66.4	36.1	54.5
chr10	9-Mar	Upstream	N	126497241	126498240	1	32.0	48.8	41.9	60.3
chr1	Agxt	Upstream	N	95030817	95031816	5	69.8	86.2	66.2	84.6
chr1	Gm5415	Upstream	N	32604139	32605138	5	81.0	97.1	81.8	100.0
chr6	Lrtm2	Upstream	N	119280785	119281784	3	4.3	55.1	23.7	41.8
chr2	Fam65c	Upstream	N	167836094	167837093	5	43.2	76.6	51.7	69.9
chr1	Hhat	Upstream	N	194597414	194598413	4	74.0	100.0	73.1	91.2
chr2	Gad2	Upstream	N	22476847	22477846	3	9.5	30.2	9.2	27.3
chr7	Alg8	Upstream	N	104519127	104520126	3	9.0	26.3	5.1	23.2
chr6	Mpp6	Upstream	N	50059240	50060239	1	14.3	33.3	24.4	42.5
chr5	2210019I11Rik	Upstream	N	148083098	148084097	5	60.7	77.0	59.5	77.2
chr19	Kat5	Upstream	N	5610095	5611094	3	52.8	75.9	59.0	76.6
chr12	Flrt2	Upstream	N	96929436	96930435	3	31.2	63.2	35.0	52.6
chr4	Map3k6	Upstream	N	132795733	132796732	3	63.4	82.9	62.7	80.2
chr3	Abca4	Upstream	N	121746378	121747377	5	48.7	66.5	49.6	67.1
chr7	Kcnq1ot1	Upstream	N	150482453	150483452	4	25.7	62.9	42.9	60.3
chr17	Zfp870	Upstream	N	33028549	33029548	5	0.6	47.6	41.6	58.9
chr4	Aqp7	Upstream	N	40995170	40996169	5	72.0	89.9	70.8	88.1
chr4	Gpx7	Upstream	N	108079319	108080318	4	35.6	63.1	48.4	65.6
chr13	Pfkp	Upstream	N	6647971	6648970	3	32.5	49.5	34.3	51.5
chr7	Olfr555	Upstream	N	109806337	109807336	5	66.0	81.7	64.3	81.3
chr14	Stc1	Upstream	N	69646346	69647345	5	38.0	76.3	18.1	35.0
chr1	Gm16880	Upstream	N	138591788	138592787	5	39.5	70.5	65.3	82.1
chr17	Mmp25	Upstream	N	23782237	23783236	3	37.5	52.9	45.0	61.9
chr4	Melk	Upstream	N	44312789	44313788	4	43.3	68.4	44.5	61.3
chr15	Ppp1r1a	Upstream	N	103368424	103369423	4	34.2	70.6	52.8	69.6
chr7	Stx1b	Upstream	N	134968046	134969045	4	60.5	78.2	58.1	74.8
chr7	Cyp2a22	Upstream	N	27724406	27725405	5	69.9	87.5	74.1	90.6
chr4	Mxra8	Upstream	N	155212789	155213788	5	20.8	64.4	53.7	70.2
chr13	Irx4	Upstream	N	73396945	73397944	2	56.9	77.1	49.0	65.5
chr10	H2afy2	Upstream	N	61246613	61247612	2	38.8	66.4	34.8	51.1
chr3	Pdzk1	Upstream	N	96632988	96633987	5	61.8	79.8	48.5	64.7
chr2	Ppp1r16b	Upstream	N	158491469	158492468	5	41.6	74.3	43.9	59.9
chr12	Serpina1b	Upstream	N	104976400	104977399	5	31.5	61.9	28.8	44.8
chr11	Pik3r5	Upstream	N	68244627	68245626	5	33.8	62.4	35.8	51.7
chr11	4930405P13Rik	Upstream	N	88766242	88767241	5	56.3	72.9	58.5	74.4
chr7	Erf	Upstream	N	26035778	26036777	1	18.9	48.8	26.6	42.4
chr2	Tmem189	Upstream	N	167487045	167488044	1	63.3	82.5	58.7	74.5
chr5	Tmem33	Upstream	N	67650891	67651890	4	39.0	77.7	19.4	35.1
chr19	Plgrkt	Upstream	N	29436362	29437361	2	21.3	42.5	30.1	45.8
chr4	Olfr275	Upstream	N	52837271	52838270	5	75.2	95.2	81.6	97.3
chr17	Calm2	Upstream	N	87846276	87847275	2	37.9	67.8	36.3	52.0
chr16	Lca5l	Upstream	N	96413865	96414864	3	43.1	64.7	39.4	55.1
chr10	Fam162b	Upstream	N	51310267	51311266	4	47.3	62.9	62.5	78.1
chr11	Gm12228	Upstream	N	54632421	54633420	5	48.0	74.1	53.0	68.6
chr2	1700017J07Rik	Upstream	N	168802769	168803768	5	39.5	62.1	42.8	58.3
chr5	Slc46a3	Upstream	N	148706379	148707378	3	39.1	62.6	35.5	50.9
chr8	Zfp868	Upstream	N	72149448	72150447	4	49.9	76.1	41.0	56.4
chr15	Fam109b	Upstream	N	82170609	82171608	2	39.8	62.1	42.0	57.3

chr4	Slc30a2	Upstream	N	133897961	133898960	4	44.3	76.6	48.7	63.8
chr6	Zc3hav1l	Upstream	N	38249260	38250259	3	40.9	69.2	55.6	70.7
chr17	Tulp1	Exon1	N	28502016	28502088	2	45.9	66.7	29.0	76.5
chr2	Zcchc3	Exon1	N	152237692	152240780	8	13.2	57.2	15.4	60.0
chr4	Mxra8	Exon1	N	155213789	155213999	2	9.5	51.1	12.5	51.3
chr4	Gm8439	Exon1	N	120261350	120261457	2	28.4	61.6	23.5	61.7
chr3	Mab21l2	Exon1	N	86349503	86352205	5	46.2	77.5	43.6	81.8
chr13	Pik3r1	Exon1	N	102462138	102462602	3	25.4	59.2	12.6	48.3
chr2	Plcb2	Exon1	N	118553974	118554174	2	31.6	62.1	35.0	69.4
chr7	Upk1a	Exon1	N	31397406	31397493	2	38.5	63.2	17.2	50.0
chr16	Tnp2	Exon1	N	10788358	10788748	3	45.8	61.4	42.8	75.6
chr11	Hes7	Exon1	N	68933955	68934017	2	8.8	32.0	4.9	37.6
chr14	Slc39a2	Exon1	N	52513285	52513589	2	18.7	61.2	23.5	55.4
chr19	Pkd2l1	Exon1	N	44266530	44266932	2	61.9	85.7	53.6	84.6
chr14	Kcnk16	Exon1	N	21088172	21088384	1	21.5	48.6	24.3	54.6
chr17	Lta	Exon1	N	35342104	35342296	1	50.0	81.3	52.2	80.9
chr7	Qprt	Exon1	N	134265487	134265543	2	27.8	57.7	22.8	51.1
chr6	Iqsec1	Exon1	N	90759762	90760117	2	59.0	78.0	49.4	75.9
chr2	Spint3	Exon1	N	164398720	164398956	2	45.8	67.3	43.5	68.7
chr11	Nlk	Exon1	N	78509311	78510927	5	70.1	100.0	69.7	94.3
chr2	Sh2d3c	Exon1	N	32576575	32576715	1	50.0	87.0	64.8	88.9
chr17	Myom1	Exon1	N	71368897	71368991	1	17.6	33.1	14.8	38.7
chr4	Trim63	Exon1	N	133871035	133871297	2	49.4	74.0	40.4	64.0
chr7	Plin1	Exon1	N	86877628	86877662	1	58.3	86.4	69.4	92.2
chr1	Hdac4	Exon1	N	94044833	94044970	2	60.0	78.7	53.2	75.0
chr2	Myo3b	Exon1	N	69877183	69877209	2	48.3	90.3	57.8	79.5
chr16	4932438H23Rik	Exon1	N	91069277	91069390	1	32.0	56.1	38.6	60.2
chr11	Sowaha	Exon1	N	53290080	53293697	8	40.0	88.8	63.4	85.0
chr2	Ttc30b	Exon1	N	75773907	75776519	12	80.0	96.9	59.6	80.7
chr14	Fam107a	Exon1	N	9141999	9142290	2	19.5	66.1	24.7	45.7
chr9	Rbp1	Exon1	N	98323380	98325470	8	10.2	65.6	38.4	58.9
chr7	Arhgef17	Exon1	N	108077083	108080675	8	64.9	89.1	65.2	85.3
chr9	Pou2af1	Exon1	N	51021795	51021906	2	30.9	72.0	38.4	58.1
chr5	Pilrb2	Exon1	N	138312797	138312986	2	63.3	90.0	49.6	69.3
chr5	Sparcl1	Exon1	N	104542396	104543107	4	73.8	89.7	69.1	88.6
chr5	Fbxo24	Exon1	N	138066167	138066306	1	37.7	85.1	64.6	83.5
chr15	Amhr2	Exon1	N	102275798	102275953	2	19.2	41.4	46.2	64.5
chr1	Arl4c	Exon1	N	90594801	90598766	12	26.8	47.3	28.3	46.6
chr6	St7	Exon1	N	17699216	17699614	3	41.2	56.7	41.2	59.5
chr13	Foxq1	Exon1	N	31650039	31652843	3	24.4	42.3	22.0	40.0
chr17	Gm7325	Exon1	N	45738960	45739051	1	18.3	36.7	19.7	37.7
chr12	Tgfb3	Exon1	N	87418531	87419991	5	34.3	57.0	36.5	54.5
chr16	Tvp23a	Exon1	N	10447023	10447443	3	15.1	33.7	16.4	34.3
chr3	Cd160	Exon1	N	96633146	96633274	2	67.1	86.3	62.3	80.0
chr11	Abi3	Exon1	N	95703723	95703790	1	23.9	59.2	31.5	49.1
chr11	Gngt2	Exon1	N	95703609	95703740	1	23.9	59.2	31.5	49.1
chr6	Asb4	Exon1	N	5333386	5333405	1	38.5	57.8	33.3	50.9
chr13	Homer1	Exon1	N	94074450	94075257	2	7.5	35.0	17.4	34.1
chr9	Fut4	Exon1	N	14552903	14556566	11	64.8	81.0	61.8	77.9

chr4	Hpdl	Exon1	N	116492512	116494113	4	65.3	82.9	65.2	81.0
chr1	Cfc1	Exon1	N	34592493	34592800	2	20.5	45.5	36.4	52.1
chr10	Ado	Exon1	N	67007259	67011703	16	58.1	80.5	59.7	75.2
chr4	1700013G24Rik	Exon1	N	137009211	137009270	1	48.6	64.0	54.7	70.2
chr17	Pou5f1	Exon1	N	35642977	35643428	3	63.7	84.1	69.5	84.9
chr2	Gm826	Exon1	N	160152832	160153230	2	25.8	69.5	20.2	35.5
chr13	Spin1	Exon1	N	51228168	51228230	2	80.0	100.0	82.9	98.0
chr2	Duoxa2	Exon1	N	122125884	122125941	1	30.0	95.0	32.2	85.2
chr1	Igsf9	Exon1	N	174414412	174414677	2	52.3	88.6	47.3	89.6
chr2	Slc20a1	Exon1	N	129025185	129025800	3	21.3	73.9	33.2	73.1
chr7	Trpm4	Exon1	N	52559979	52560084	1	50.0	65.9	50.7	88.9
chr6	Itpr1	Exon1	N	108164197	108164272	1	17.4	53.6	14.5	50.5
chr11	Ehbp1	Exon1	N	21907086	21907200	1	58.7	77.4	46.0	81.0
chr1	4930487H11Rik	Exon1	N	62840970	62841045	1	36.7	82.7	37.9	72.6
chr1	Cnot11	Exon1	N	39594261	39594425	1	49.3	68.0	32.8	67.1
chr2	Mdk	Exon1	N	91770975	91771136	2	11.1	36.1	12.0	45.4
chr12	Serpina3h	Exon1	N	105488350	105488967	3	52.1	96.2	13.5	46.7
chr7	Ino80e	Exon1	N	134000740	134000856	1	5.7	24.1	10.1	41.5
chr11	Cbx8	Exon1	N	118901246	118901312	1	42.2	68.1	45.9	77.2
chr17	Tiam2	Exon1	N	3412632	3412750	1	39.3	64.7	39.5	70.0
chr9	Bcl9l	Exon1	N	44307531	44307632	2	47.5	71.8	49.2	79.8
chr6	Pcyox1	Exon1	N	86341670	86341822	1	38.6	60.4	34.6	65.2
chr9	Rbp2	Exon1	N	98399135	98399313	1	33.3	64.5	35.1	62.9
chr16	Ttc3	Exon1	N	94605519	94605673	2	34.2	50.0	24.0	50.0
chr2	Tasp1	Exon1	N	139683438	139683511	1	79.2	95.7	69.8	95.1
chr3	Pygo2	Exon1	N	89235049	89235098	2	27.9	54.5	10.5	35.6
chr17	Lta	Exon1	N	35341320	35341419	1	68.1	97.5	69.7	94.2
chr13	Irx4	Exon1	N	73402296	73402427	2	47.8	83.7	60.5	84.9
chr9	Apoa5	Exon1	N	46077164	46077220	2	43.7	67.5	71.3	95.2
chr15	A4galt	Exon1	N	83060944	83061087	2	61.6	83.0	59.3	82.1
chr2	1700109F18Rik	Exon1	N	74552328	74552458	2	16.2	40.3	18.8	40.4
chr4	Epb4.1l4b	Exon1	N	57077671	57077735	1	53.4	74.2	47.9	69.4
chr17	Epb4.1l3	Exon1	N	69557199	69557416	2	55.3	72.4	55.5	77.0
chr17	Ltb	Exon1	N	35331995	35332310	1	40.6	65.8	37.6	59.0
chr2	Myo3b	Exon1	N	69877355	69877397	1	42.7	61.2	43.2	64.2
chr7	D430042O09Rik	Exon1	N	132871823	132871878	1	64.4	85.2	60.3	80.6
chr6	Klra1	Exon1	N	130322839	130322942	1	32.4	50.0	35.6	55.6
chr1	Nsl1	Exon1	N	192889054	192889132	1	27.1	60.0	41.5	61.3
chr11	Gm525	Exon1	N	88936303	88936403	1	6.7	34.6	15.2	34.8
chr5	Agfg2	Exon1	N	138105444	138105600	1	50.0	84.1	48.5	67.7
chr3	Cttnbp2nl	Exon1	N	104810866	104810973	1	29.8	52.0	22.7	41.5
chr2	Gm16119	Exon1	N	181315148	181315333	2	47.5	90.2	22.2	41.0
chr12	Frmd6	Exon1	N	71964864	71965129	3	71.6	93.5	74.1	92.4
chr10	Il23a	Exon1	N	127734134	127734280	1	57.1	74.2	62.2	80.4
chr16	Rcan1	Exon1	N	92396111	92396270	1	55.9	79.5	62.7	81.0
chr9	Chrna5	Exon1	N	54845819	54845970	1	29.0	62.7	37.5	55.7
chr19	1700055N04Rik	Exon1	N	3963839	3964001	1	62.8	79.3	64.2	82.4
chr12	Trim9	Exon1	N	71349252	71349414	1	46.3	64.7	40.6	58.6
chr4	Tox	Exon1	N	6615966	6616117	1	50.6	67.5	54.1	71.7

chr15	A330009N23Rik	Exon1	N	101053912	101054064	2	26.2	50.2	20.6	38.1
chr4	Rere	Exon1	N	149780305	149780773	4	64.6	81.0	60.7	78.1
chr12	Dnmt3a	Exon1	N	3835027	3835260	2	32.6	61.2	40.5	57.8
chr19	5830416P10Rik	Exon1	N	53519740	53519834	1	21.3	41.6	19.1	36.4
chr19	Snx15	Exon1	N	6120486	6120743	1	39.2	80.7	42.2	59.4
chr12	Grhl1	Exon1	N	25260779	25260965	1	35.1	83.3	55.9	72.8
chr7	Lrrc28	Exon1	N	74674472	74674631	1	60.0	80.2	66.1	82.7
chr15	Yaf2	Exon1	N	93116841	93116993	1	40.9	65.1	49.6	66.2
chr9	Snx22	Exon1	N	65915320	65915387	1	47.6	65.8	43.6	59.8
chr7	Gas2	Exon1	N	59143282	59143446	2	51.2	90.6	61.4	77.5
chr6	Pianp	Exon1	N	124948936	124948995	2	31.6	53.4	36.8	52.7
chr17	Rrp1b	Exon1	N	32182874	32182956	1	79.4	96.3	79.6	95.5
chr19	4930526L06Rik	Exon1	N	11367130	11367302	1	47.3	80.5	56.9	72.7
chr19	Lipa	5-UTR	N	34599333	34601964	10	10.8	93.8	9.7	60.3
chr11	Brip1	5-UTR	N	86012467	86014695	9	61.9	100.0	50.0	100.0
chr3	Mab21l2	5-UTR	N	86351614	86352205	1	52.6	73.9	39.3	87.1
chr11	Fbxo48	5-UTR	N	16851413	16853378	7	9.3	46.6	3.8	48.7
chr12	Rps6kl1	5-UTR	N	86490839	86492214	4	16.7	68.4	27.3	72.1
chr1	4930487H11Rik	5-UTR	N	62841127	62841306	1	26.6	81.6	42.4	81.3
chr4	Gm8439	5-UTR	N	120261350	120261409	1	28.4	61.6	23.5	61.7
chr11	Hes7	5-UTR	N	68933955	68933975	1	7.4	35.7	3.1	41.1
chr7	Mir675	5-UTR	N	149762969	149763052	1	64.4	81.9	51.1	87.2
chr14	Slc39a2	5-UTR	N	52513285	52513474	1	6.0	45.8	7.0	41.7
chr9	Upk2	5-UTR	N	44262790	44262850	1	23.8	55.2	34.4	67.6
chr9	Bcl9l	5-UTR	N	44307219	44307606	1	47.5	71.8	49.2	79.8
chr2	Mir130a	5-UTR	N	84581272	84581335	1	65.7	89.1	63.2	93.2
chr5	2210019I11Rik	5-UTR	N	148082796	148083097	2	46.2	70.9	40.4	69.5
chr7	Qprt	5-UTR	N	134265500	134265543	1	27.8	57.7	22.8	51.1
chr14	Ndrg2	5-UTR	N	52531216	52533163	6	30.6	53.3	37.2	65.0
chr1	Ormdl1	5-UTR	N	53353939	53355822	7	34.2	56.8	71.0	97.6
chr9	Nek11	5-UTR	N	105295352	105297617	10	9.8	50.3	29.2	55.4
chr3	Spry1	5-UTR	N	37538872	37541531	5	13.7	34.5	16.5	42.6
chr16	Mir193b	5-UTR	N	13449616	13449694	1	73.6	89.6	63.0	88.9
chr13	Irx4	5-UTR	N	73397945	73402382	9	26.5	61.5	32.6	57.9
chr5	Gm16063	5-UTR	N	120075951	120076039	1	23.8	62.1	31.1	56.1
chr17	Cldn6	5-UTR	N	23816332	23818030	5	53.2	80.0	55.0	79.8
chr2	Sh2d3c	5-UTR	N	32576575	32576678	1	50.0	87.0	64.8	88.9
chr1	Gm16880	5-UTR	N	138592788	138592968	1	15.7	45.3	9.9	33.8
chr9	Fbxl12	5-UTR	N	20443643	20449211	16	46.4	65.5	28.2	51.9
chr10	Ascc1	5-UTR	N	59465553	59467566	6	7.0	52.7	34.2	57.9
chr2	Bmi1	5-UTR	N	18598645	18603438	15	34.3	58.7	49.4	73.0
chr4	AW011738	5-UTR	N	155577393	155580137	8	53.2	69.9	55.4	79.0
chr9	Stt3a	5-UTR	N	36572727	36575163	9	45.7	61.4	61.6	84.8
chr17	Lta	5-UTR	N	35341599	35342296	3	35.6	64.2	34.6	57.7
chr6	Vwf	5-UTR	N	125502966	125504470	8	51.8	68.1	39.6	62.4
chr7	Serpinh1	5-UTR	N	106497932	106501749	16	50.8	85.2	62.6	84.6
chr3	Ankrd50	5-UTR	N	38382125	38383738	5	45.0	63.8	47.5	69.5
chr1	Mir30a	5-UTR	N	23279108	23279178	1	29.3	50.0	29.9	51.9
chr1	Hdac4	5-UTR	N	94044855	94044970	1	60.0	78.7	53.2	75.0

chr2	Myo3b	5-UTR	N	69877183	69877202	1	48.3	90.3	57.8	79.5
chr7	Hrc	5-UTR	N	52590639	52590796	1	44.9	68.0	56.3	77.8
chr11	Atp6v0a1	5-UTR	N	100870766	100873134	8	34.0	60.3	27.3	48.8
chr11	Gm12228	5-UTR	N	54633421	54633563	1	24.1	66.1	35.8	57.1
chr2	Pacsin3	5-UTR	N	91096970	91100364	13	47.9	76.8	50.7	71.5
chr9	Rbp1	5-UTR	N	98323380	98325397	8	10.2	65.6	38.4	58.9
chr15	Fam109b	5-UTR	N	82171609	82173711	8	34.8	79.3	52.0	72.3
chr4	Trim63	5-UTR	N	133871035	133871138	1	49.4	74.0	50.3	70.4
chr6	Ccdc37	5-UTR	N	90377484	90378474	4	8.3	37.9	27.1	47.2
chr12	Tgfb3	5-UTR	N	87418883	87419991	3	28.4	56.8	31.0	51.0
chr9	Pou2af1	5-UTR	N	51021795	51021890	1	30.9	72.0	38.4	58.1
chr2	Gdf5	5-UTR	N	155770792	155771100	2	59.7	92.0	64.5	84.1
chr14	Cdh24	5-UTR	N	55258382	55260201	6	53.6	78.0	53.4	72.8
chr15	Nrbp2	5-UTR	N	75920055	75920443	2	61.3	84.8	65.4	84.6
chr5	Fbxo24	5-UTR	N	138066170	138066306	1	37.7	85.1	64.6	83.5
chr17	Kremen2	5-UTR	N	23881993	23882796	2	13.6	29.5	16.3	34.9
chr5	AI839979	5-UTR	N	31873557	31873770	1	9.7	26.8	2.8	21.1
chr6	St7	5-UTR	N	17699216	17699592	2	41.2	56.7	41.2	59.5
chr19	Slc22a6	5-UTR	N	8692486	8692785	1	38.6	63.3	39.7	57.9
chr4	Eno1	5-UTR	N	149611306	149613590	8	8.8	38.8	14.8	32.9
chr17	Gm7325	5-UTR	N	45738621	45739051	2	18.3	36.7	19.7	37.7
chr16	Tvp23a	5-UTR	N	10447139	10447443	2	15.1	36.5	16.4	34.3
chr5	Sult1b1	5-UTR	N	87964110	87967220	15	35.7	83.3	0.0	17.6
chr17	Gm6623	5-UTR	N	36315722	36318309	9	34.5	55.7	38.3	55.4
chr1	Pkhd1	5-UTR	N	20605405	20608138	14	24.4	42.0	30.5	47.3
chr4	Ccdc28b	5-UTR	N	129300008	129301152	6	36.4	57.6	27.7	44.5
chr4	Angptl7	5-UTR	N	147874399	147874571	1	53.0	78.4	67.6	84.2
chr4	Myom3	5-UTR	N	135315630	135318434	13	61.6	82.7	64.0	80.4
chr2	Sec16a	5-UTR	N	26297522	26300736	13	31.4	51.6	39.5	55.0
chr2	Cubn	5-UTR	N	13413441	13413503	1	22.2	39.0	24.3	39.6
chr5	2310034O05Rik	5-UTR	N	100639711	100640046	2	58.6	81.6	63.2	78.3
chr14	Pbrm1	5-UTR	N	31832324	31838698	29	40.1	63.9	40.2	55.2

Group 6-mDMR genes associated with methylation gain at gene body and 3' non-CGI

							DNA Methylation Level (%)			
							ISCs		Diff. Cells	
Chr	Gene	Region	CGI	Start	End	#Bins	P0	P21	P0	P21
chr4	Cdkn2c	LastExon	N	109333481	109334231	4	37.3	86.7	19.9	82.9
chr1	Dtl	LastExon	N	193361244	193363220	10	29.0	45.0	39.5	83.2
chr5	Atraid	LastExon	N	31356636	31356996	1	28.1	79.2	34.3	78.0
chr2	Myo3a	LastExon	N	22473569	22473772	2	21.5	46.8	14.2	57.1
chr16	5-Sep	LastExon	N	18621904	18622889	3	66.3	85.5	49.7	87.3
chr11	Arl5c	LastExon	N	97850894	97851653	5	36.6	70.9	28.9	64.1
chr11	Vat1	LastExon	N	101320062	101321590	8	33.5	57.2	33.2	65.4
chr5	Drc1	LastExon	N	30668777	30668993	2	20.0	48.2	22.9	54.6
chr4	Cdkn2b	LastExon	N	88952198	88953223	3	49.2	92.5	54.1	85.6
chr17	H2-Ke2	LastExon	N	34075854	34076056	2	36.1	57.4	35.8	67.2
chr1	Fzd5	LastExon	N	64777132	64783427	21	37.4	58.1	28.9	58.5
chr2	Sp5	LastExon	N	70314081	70315783	1	24.0	69.7	19.5	48.9

chr19	Arl3	LastExon	N	46605599	46605876	2	52.7	80.0	47.9	77.1
chr5	Card11	LastExon	N	141348953	141349483	1	22.9	59.8	30.1	59.1
chr18	Dsc3	LastExon	N	20119431	20122070	13	57.6	91.7	62.5	90.7
chr10	Map3k5	LastExon	N	19861329	19862556	7	53.3	75.6	49.9	77.8
chr15	Foxh1	LastExon	N	76498654	76499570	3	42.5	74.5	47.6	75.5
chr1	Fam5c	LastExon	N	148748131	148749602	8	53.4	75.9	60.6	87.6
chr19	Rab3il1	LastExon	N	10108947	10110076	6	9.1	62.9	60.3	86.9
chr4	Cga	LastExon	N	34854315	34854623	2	58.0	88.1	62.3	88.6
chr11	Wbp2	LastExon	N	115939887	115940910	6	62.2	86.9	58.6	84.1
chr3	Vps45	LastExon	N	95803755	95804074	2	43.3	65.1	47.0	72.5
chr2	Ccdc32	LastExon	N	118843515	118844900	7	72.5	93.6	69.2	93.7
chr15	Kifc2	LastExon	N	76497634	76498626	6	49.7	77.6	49.9	73.7
chr11	Zfp735	LastExon	N	73523972	73527310	11	48.7	66.7	34.6	57.1
chr9	Dnm2	LastExon	N	21311867	21312203	2	21.6	43.2	24.5	47.0
chr9	Mmp13	LastExon	N	7282018	7283333	7	75.6	91.9	73.9	96.2
chr11	Rhbdd3	LastExon	N	5005803	5006094	3	66.7	92.3	73.7	95.9
chr9	Gnai2	LastExon	N	107516469	107517429	5	62.5	82.3	57.4	79.6
chr17	Cd2ap	LastExon	N	42929900	42933318	16	65.0	88.0	64.9	87.0
chr9	Lars2	LastExon	N	123370617	123371782	4	31.9	53.4	41.0	62.9
chr2	Hoxd8	LastExon	N	74544571	74545364	3	26.2	53.1	30.8	52.4
chr12	A230065H16Rik	LastExon	N	112649935	112650288	2	69.0	85.8	57.2	78.7
chr11	Krt20	LastExon	N	99289717	99290392	4	20.6	54.1	24.7	46.1
chr14	Il17rb	LastExon	N	30809354	30810375	5	64.1	93.7	68.3	89.4
chr2	Pdhx	LastExon	N	102861213	102862426	6	64.9	83.3	62.5	83.4
chr10	Shmt2	LastExon	N	126954179	126954972	5	40.2	76.6	50.3	70.8
chr2	Foxa2	LastExon	N	147868614	147870560	3	30.4	84.3	29.7	50.0
chr7	Tmc7	LastExon	N	125679358	125681731	12	41.2	62.5	70.3	90.6
chr6	Ddx47	LastExon	N	134973297	134973794	3	27.7	50.1	29.6	49.8
chr7	1600016N20Rik	LastExon	N	148395942	148396103	2	71.5	87.4	71.3	91.3
chr13	Tnpo1	LastExon	N	99612036	99614575	13	76.2	92.7	76.5	96.4
chr7	Msx3	LastExon	N	147232056	147234039	7	58.8	75.5	50.3	70.0
chr11	Kdm6b	LastExon	N	69212020	69213351	5	33.3	60.3	42.5	62.2
chr5	Tbx3	LastExon	N	120132640	120134610	8	56.3	75.4	35.6	55.2
chr8	Aktip	LastExon	N	93647398	93648577	7	62.4	78.3	60.2	79.8
chr9	Chrna3	LastExon	N	54859150	54860736	8	61.5	86.7	65.3	84.8
chr3	Slc50a1	LastExon	N	89072168	89072520	2	67.9	87.1	58.9	78.4
chr7	Rplp2	LastExon	N	148637131	148637241	2	33.6	62.7	54.8	74.1
chr11	Cd7	LastExon	N	120898063	120898464	2	49.0	83.6	61.2	80.4
chr3	Spry1	LastExon	N	37541477	37543521	11	20.1	62.6	34.8	53.3
chr1	2010300C02Rik	LastExon	N	37668521	37669241	4	29.4	58.0	36.6	55.1
chr17	Vmn2r94	LastExon	N	18380534	18381465	5	58.6	76.6	51.2	69.3
chr3	Dkk2	LastExon	N	131840834	131843268	12	77.7	96.1	76.7	94.8
chr5	Vmn2r12	LastExon	N	109514868	109515730	4	60.9	95.7	77.5	95.5
chr7	Prkcdbp	LastExon	N	112629130	112629733	3	49.7	71.3	58.7	76.6
chr2	BC052040	LastExon	N	115602638	115604504	10	45.6	96.3	48.0	65.8
chr9	Rwdd2a	LastExon	N	86467581	86468506	4	79.2	95.1	66.9	84.6
chr9	Sema7a	LastExon	N	57809047	57810672	8	51.9	94.6	58.8	76.4
chr6	Nxph1	LastExon	N	9197085	9198578	8	67.4	86.1	67.9	85.5
chr8	Hpgd	LastExon	N	58798855	58799843	5	65.5	88.4	63.1	80.4

chr19	Klc2	LastExon	N	5107746	5108688	4	13.5	31.7	41.0	58.1
chr4	Eps15	LastExon	N	109057924	109060421	13	80.8	97.6	78.7	95.8
chr8	Nwd1	LastExon	N	75235492	75238645	15	52.2	70.9	67.2	83.5
chr1	BC026585	LastExon	N	159418574	159418864	2	66.6	84.4	79.9	96.0
chr11	Ace	LastExon	N	105850116	105851278	4	36.7	58.1	36.4	52.4
chr8	Nfix	LastExon	N	87231498	87232576	6	0.4	44.3	3.6	19.5
chr17	2300002M23Rik	LastExon	N	35704779	35705890	6	21.0	40.4	27.2	42.7
chr8	Ccl17	LastExon	N	97335660	97335936	2	64.5	80.6	67.0	82.4
chr8	Gtf2e2	LastExon	N	34886925	34887645	4	12.5	61.2	21.2	36.6
chr19	Lipa	Intron9	N	34599337	34601855	10	10.8	93.8	9.7	60.3
chr6	Lrp6	Intron9	N	134417718	134418660	5	42.9	90.0	40.9	75.0
chr16	5-Sep	Intron9	N	18625349	18625437	1	24.2	90.9	49.3	80.5
chr15	Col22a1	Intron9	N	71646429	71650460	20	55.0	77.1	53.8	85.0
chr18	Zfp608	Intron9	N	55148361	55149481	7	14.3	42.5	13.9	40.6
chr9	Vwa5a	Intron9	N	38535428	38535578	1	41.2	58.1	37.8	63.1
chr8	Nfix	Intron9	N	87296215	87323896	120	45.5	69.3	44.4	69.5
chr7	Capns1	Intron9	N	30977920	30978896	3	31.7	56.6	32.1	56.7
chr4	Tmco4	Intron9	N	138575586	138575960	2	48.8	65.1	46.3	69.7
chr17	Efhb	Intron9	N	53585181	53588709	18	72.2	90.7	70.8	93.7
chr4	Espn	Intron9	N	151507066	151508269	6	60.4	75.7	52.0	73.2
chr17	Catsperd	Intron9	N	56790317	56791217	5	28.2	69.0	48.0	69.2
chr17	Rgs11	Intron9	N	26342721	26344243	8	50.8	81.5	63.7	84.3
chr8	Ces1a	Intron9	N	95557932	95559850	10	3.1	19.1	8.8	29.3
chr6	Dqx1	Intron9	N	83014638	83014787	1	10.4	45.5	24.4	43.7
chr7	Adck4	Intron9	N	28027313	28028321	5	46.8	63.4	47.1	66.3
chr11	Rufy1	Intron9	N	50219986	50221324	7	39.9	65.2	68.6	87.4
chr4	Rap1gap	Intron9	N	137272489	137273312	4	40.2	70.4	44.6	63.4
chr17	Kremen2	Intron9	N	23882071	23882705	2	13.6	29.5	16.3	34.9
chr8	Tsnaxip1	Intron9	N	108365706	108365781	1	75.0	96.3	82.1	100.0
chr4	Aldh4a1	Intron9	N	139196602	139197991	7	73.4	88.5	57.8	75.4
chr1	Mdm4	Intron9	N	134909289	134915353	29	64.0	93.5	75.3	92.9
chr5	Cep135	Intron9	N	77038795	77040674	9	19.4	39.5	30.1	46.6
chr11	Mapt	Intron9	N	104179564	104182664	13	57.1	86.7	61.9	77.7
chr8	Tmem184c	Intron9	N	80130317	80134001	17	52.8	69.9	71.1	86.7
chr5	Ppargc1a	Intron9	N	51886045	51886908	4	53.8	85.4	59.4	74.6
chr11	Gabra1	Intron8	N	41989514	41993061	14	57.6	74.5	27.6	75.0
chr8	Col4a2	Intron8	N	11402235	11402983	4	18.8	63.7	51.1	93.7
chr12	Ifrd1	Intron8	N	40940723	40942776	10	41.3	59.6	34.3	68.2
chr15	Baiap2l2	Intron8	N	79101083	79101631	1	14.2	55.9	14.2	43.0
chr10	Syne1	Intron8	N	5184208	5184979	4	32.1	94.2	32.7	60.7
chr12	Ptgr2	Intron8	N	85649383	85651868	12	47.5	64.5	41.0	68.5
chr11	Slc22a4	Intron8	N	53819051	53821269	11	59.5	90.1	58.9	85.6
chr6	Ppp1r9a	Intron8	N	5061054	5063600	12	11.9	51.3	39.0	65.6
chr7	Trpm4	Intron8	N	52564601	52565646	5	35.0	58.3	59.6	85.6
chr19	Rtn3	Intron8	N	7542358	7557428	70	50.3	74.1	39.9	64.7
chr17	4930539E08Rik	Intron8	N	29042441	29042635	1	49.7	73.4	49.7	74.1
chr14	Sh3bp5	Intron8	N	32247332	32248996	4	51.3	68.7	42.9	66.1
chr6	Mpp6	Intron8	N	50128520	50130174	8	39.3	67.4	59.5	81.2
chr5	Ppargc1a	Intron8	N	51881485	51885839	20	75.6	90.9	58.3	79.3

chr1	Lamc2	Intron8	N	154980879	154981906	5	51.6	76.1	46.5	67.3
chr12	G2e3	Intron8	N	52462035	52464172	11	80.2	97.6	75.9	96.0
chr9	Ecsit	Intron8	N	21888653	21889800	4	77.3	97.8	70.1	90.0
chr7	Capns1	Intron8	N	30977612	30977885	1	78.0	97.1	73.4	92.1
chr10	Mfsd12	Intron8	N	80825773	80826297	3	35.7	55.0	40.5	59.1
chr5	Tmem184a	Intron8	N	140289147	140290006	4	21.0	45.0	7.6	25.3
chr3	Gucy1b3	Intron8	N	81848168	81849320	6	67.0	88.5	64.4	81.0
chr4	Serinc2	Intron8	N	129941696	129942312	1	27.9	49.4	20.0	35.8
chr4	Adamtsl1	Intron8	N	85873959	85878456	22	66.1	84.6	67.4	82.9
chr2	Lrp2	Intron77	N	69389010	69390409	7	61.2	76.7	78.4	94.3
chr1	Ush2a	Intron70	N	190786819	190788717	9	62.7	83.9	64.6	87.6
chr9	Zfp810	Intron7	N	22110047	22111992	8	12.1	29.0	16.2	57.2
chr15	Naga	Intron7	N	82167372	82167816	2	44.2	84.0	28.6	68.3
chr15	Cyb5r3	Intron7	N	82992735	82996504	18	48.6	93.2	54.1	93.8
chr8	Mau2	Intron7	N	72547597	72549726	11	32.0	95.8	33.9	73.0
chr2	Sh2d3c	Intron7	N	32605206	32606471	6	57.9	77.1	36.9	73.0
chr1	Espnl	Intron7	N	93238521	93239117	3	61.7	88.9	57.1	91.4
chr19	Sufu	Intron7	N	46527806	46529224	7	36.7	82.0	49.7	81.5
chr6	Scnn1a	Intron7	N	125289242	125292619	17	61.4	93.1	60.9	91.5
chr19	Aldh3b2	Intron7	N	3979617	3980005	2	50.0	70.2	54.7	83.3
chr15	Galnt6	Intron7	N	100534014	100534541	2	58.5	75.9	49.1	76.9
chr1	Susd4	Intron7	N	184822397	184825329	15	43.2	66.2	66.8	94.2
chr8	Frem3	Intron7	N	83214721	83219025	21	72.5	87.9	62.5	89.2
chr17	BC004004	Intron7	N	29433635	29435586	10	36.2	66.1	37.5	63.2
chr7	Trim21	Intron7	N	109712855	109713902	6	27.3	44.7	28.7	53.2
chr11	Larp1	Intron7	N	57856409	57857407	5	66.0	83.7	70.2	92.4
chr5	Setd8	Intron7	N	124909975	124910496	3	28.8	43.8	60.9	82.9
chr5	Stim2	Intron7	N	54496638	54500941	22	55.3	71.1	55.1	76.9
chr11	Aatf	Intron7	N	84284789	84286553	9	24.5	76.8	53.7	73.5
chr3	Larp7	Intron7	N	127249322	127249393	1	53.1	79.5	61.9	80.9
chr2	1700019L03Rik	Intron7	N	32636316	32636873	3	22.7	45.6	53.5	71.5
chr5	Sult1b1	Intron7	N	87964122	87967129	15	35.7	83.3	0.0	17.6
chr11	Galk1	Intron7	N	115872531	115873827	5	27.8	53.6	18.9	36.5
chr17	Rgs11	Intron7	N	26341873	26342052	1	51.6	73.1	59.0	76.4
chr6	Atg7	Intron7	N	114634540	114636091	8	41.9	64.5	51.8	68.6
chr14	Lmo7	Intron7	N	102280219	102283378	16	44.0	75.2	53.9	70.6
chr10	Ikzf4	Intron7	N	128081025	128082776	8	66.1	83.7	67.0	83.0
chr1	Ncstn	Intron7	N	174000280	174001381	6	75.0	90.8	69.9	85.8
chr4	Serinc2	Intron7	N	129940906	129941504	3	75.6	91.0	75.7	91.4
chr8	Gtf2e2	Intron7	N	34886532	34886924	2	60.8	78.6	71.3	86.8
chr8	Itfg1	Intron7	N	88262125	88264126	10	75.1	94.3	78.3	93.8
chr2	Scn1a	Intron7	N	66134933	66137680	14	64.5	80.2	66.9	81.9
chr5	Gm15800	Intron67	N	121808344	121809305	5	65.0	90.5	74.9	94.9
chr1	Pkhd1	Intron66	N	20605487	20607991	13	28.4	50.3	35.4	54.5
chr4	Vps13d	Intron64	N	144763062	144764711	8	63.0	83.9	79.1	94.2
chr5	Atraid	Intron6	N	31356432	31356635	1	28.1	79.2	34.3	78.0
chr17	Tfeb	Intron6	N	47925243	47925857	3	56.4	75.3	36.7	69.8
chr13	Mef2c	Intron6	N	83772845	83775024	10	54.3	86.4	60.7	92.8
chr16	Usp16	Intron6	N	87470108	87470598	2	64.3	94.2	66.1	96.2

chr2	Ubox5	Intron6	N	130454372	130455651	5	0.0	34.0	48.1	77.0
chr10	Specc1l	Intron6	N	74720687	74721712	5	44.0	73.7	46.3	75.1
chr16	Liph	Intron6	N	21974221	21976305	9	55.9	73.0	48.7	76.0
chr16	Tbx1	Intron6	N	18585230	18586628	4	25.8	63.0	30.8	57.6
chr15	Slc45a2	Intron6	N	10955689	10957469	9	70.4	87.0	69.8	93.4
chr2	1700001O22Rik	Intron6	N	30656522	30659069	7	74.4	92.7	71.9	94.5
chr7	Myh14	Intron6	N	51869101	51869662	3	48.5	67.9	44.6	67.0
chr18	Cndp2	Intron6	N	84841551	84844368	14	50.8	82.5	48.2	70.2
chr4	Ccdc24	Intron6	N	117544466	117544593	1	48.0	78.6	41.9	63.2
chr4	Rcc2	Intron6	N	140268590	140269313	4	51.5	85.8	64.2	85.4
chr11	Bahcc1	Intron6	N	120134738	120135007	1	73.0	88.9	71.4	92.5
chr11	Spns3	Intron6	N	72350183	72350672	2	65.0	85.4	59.3	80.0
chr4	Ttll10	Intron6	N	155419000	155419433	2	43.5	59.3	39.0	59.2
chr14	9930012K11Rik	Intron6	N	70557462	70559138	6	22.6	58.0	51.8	70.9
chr17	Snx9	Intron6	N	5902515	5904888	12	43.7	71.7	66.7	85.2
chr18	Dnajc18	Intron6	N	35856778	35860457	18	75.8	94.0	68.6	87.1
chr13	Slc34a1	Intron6	N	55504141	55504265	1	68.8	87.2	67.6	86.0
chr16	Urb1	Intron6	N	90760188	90760635	2	57.7	73.6	55.4	73.6
chr16	Bcl6	Intron6	N	23973306	23974898	8	64.6	88.5	73.1	91.2
chr11	Cpsf4l	Intron6	N	113570169	113571135	6	56.8	77.9	48.8	66.8
chr12	Rad51b	Intron6	N	80426074	80428207	11	40.0	85.9	55.7	73.5
chr5	Noa1	Intron6	N	77736743	77737931	5	43.9	67.9	43.8	61.3
chr2	Tyro3	Intron6	N	119631467	119633570	11	63.8	82.7	63.1	80.4
chr2	Dnm1	Intron6	N	32171431	32172417	5	14.3	40.9	21.7	39.0
chr1	Farp2	Intron6	N	95464092	95465657	8	28.7	49.0	46.0	63.3
chr2	Nebl	Intron6	N	17651091	17652419	4	2.8	22.2	8.3	24.8
chr8	Tpte	Intron6	N	23416367	23417407	4	72.0	89.6	73.8	89.5
chr2	Fmn1	Intron6	N	113366099	113369469	17	78.4	94.7	83.2	98.9
chr2	Nr4a2	Intron6	N	56964853	56965361	3	36.8	57.0	36.0	51.4
chr11	Aftph	Intron6	N	20609750	20612166	12	65.8	83.9	64.0	79.1
chr8	Acta1	Intron6	N	126417610	126418321	4	48.6	71.6	51.6	66.8
chr11	Galk1	Intron6	N	115871997	115872340	2	62.5	80.6	72.7	87.8
chr15	Vps13b	Intron57	N	35844706	35846801	10	60.2	77.9	74.5	90.2
chr11	Zzef1	Intron54	N	72736684	72738138	7	32.1	84.6	59.6	79.3
chr7	Serpinh1	Intron5	N	106500378	106501562	4	31.8	84.5	31.2	78.1
chr6	Styk1	Intron5	N	131257173	131259900	14	6.1	59.4	58.2	96.9
chr9	Smad3	Intron5	N	63511523	63513954	12	23.9	51.4	34.1	68.7
chr1	Lamc2	Intron5	N	154977281	154977779	2	36.5	70.4	35.0	68.6
chr7	Ccdc61	Intron5	N	19477933	19478346	1	30.7	57.5	29.9	60.6
chr2	Hoxd4	Intron5	N	74565763	74566318	2	14.9	86.9	23.6	53.6
chr10	2610008E11Rik	Intron5	N	78557303	78560202	13	43.2	80.0	43.8	72.6
chr5	Rell1	Intron5	N	64327334	64329044	9	57.5	77.1	53.0	80.8
chr3	Ppm1j	Intron5	N	104587046	104587551	3	69.4	84.9	56.6	84.4
chr6	Pon3	Intron5	N	5186999	5190812	19	81.3	96.8	68.9	96.4
chr2	Dnm1	Intron5	N	32170868	32171318	2	61.3	96.3	51.2	78.6
chr4	Cd164l2	Intron5	N	132779663	132780166	3	17.1	66.7	31.3	57.1
chr6	E230016M11Rik	Intron5	N	67027360	67028220	4	48.0	68.5	49.9	75.6
chr6	Ica1	Intron5	N	8606409	8608219	8	51.0	74.7	54.4	79.0
chr11	Gm11978	Intron5	N	6559649	6560086	2	42.9	61.4	52.2	76.3

chr19	Rab1b	Intron5	N	5105251	5106924	6	3.3	24.4	29.8	53.5
chr14	Ecd	Intron5	N	21148212	21149175	5	72.5	88.5	66.8	90.2
chr6	Foxp1	Intron5	N	98894715	98895340	3	48.2	64.5	51.4	74.4
chr8	Nfix	Intron5	N	87250505	87251022	3	60.0	75.5	63.2	86.0
chr5	Cad	Intron5	N	31361496	31361805	2	55.6	71.1	54.1	75.6
chr5	Stard13	Intron5	N	151849554	151850166	3	9.7	41.7	25.6	46.3
chr2	Prdm11	Intron5	N	92853574	92853921	2	49.9	80.7	66.7	86.9
chr17	Mocs1	Intron5	N	49588487	49588759	1	58.9	78.0	61.5	80.9
chr10	Ube2d1	Intron5	N	70724864	70724946	1	78.8	97.4	79.4	98.4
chr1	Nr5a2	Intron5	N	138840006	138841535	8	54.3	71.8	53.1	71.9
chr6	Slc41a3	Intron5	N	90583750	90585243	7	69.4	86.1	74.8	93.5
chr6	Cpa2	Intron5	N	30497207	30499241	10	71.4	89.4	71.1	89.7
chr16	Pla1a	Intron5	N	38407846	38409680	9	67.4	90.8	62.5	81.0
chr19	Slc1a1	Intron5	N	28970617	28971996	7	52.4	83.3	62.1	80.7
chr1	Stau2	Intron5	N	16359968	16364754	23	71.2	87.0	64.2	82.6
chr13	Tspan17	Intron5	N	54896540	54896771	1	41.1	56.7	42.7	60.6
chr2	Nr4a2	Intron5	N	56963178	56963986	3	29.7	58.4	36.8	54.3
chr4	Ccdc28b	Intron5	N	129300032	129301057	6	36.4	57.6	27.7	44.5
chr14	Tinf2	Intron5	N	56299545	56299631	1	73.0	93.8	70.4	87.0
chr2	Neb	Intron5	N	52004798	52005109	2	66.7	90.9	80.0	95.7
chr9	Gsta4	Intron5	N	78053861	78055607	9	69.3	87.0	76.0	91.6
chr17	Prss22	Intron5	N	24134305	24134889	3	64.0	91.1	62.1	77.5
chr15	Cela1	Intron5	N	100515137	100515564	2	48.7	66.0	44.3	59.4
chr1	Ncf2	Intron5	N	154671455	154673011	8	76.3	96.9	79.8	94.9
chr1	Tpr	Intron49	N	152293372	152294641	6	8.7	62.5	11.1	82.6
chr15	Lrrk2	Intron48	N	91641576	91642622	5	44.2	78.8	38.2	54.0
chr13	Lyst	Intron45	N	13839070	13844209	24	68.9	91.1	72.1	89.2
chr5	Zan	Intron42	N	137858408	137860270	9	37.5	55.1	61.0	79.9
chr5	Rbm48	Intron4	N	3595446	3595965	2	3.0	64.9	4.0	44.5
chr2	Gad2	Intron4	N	22480552	22484910	22	46.0	82.4	33.9	68.1
chr2	7-Mar	Intron4	N	60070425	60071955	8	40.8	70.7	49.5	83.7
chr2	Nr4a2	Intron4	N	56962689	56963047	2	52.6	70.6	37.5	71.4
chr9	Tcaim	Intron4	N	122723707	122727891	21	56.5	75.0	60.4	92.2
chr2	Gm14318	Intron4	N	180074072	180074512	2	65.0	88.6	53.2	83.9
chr11	Ddc	Intron4	N	11722297	11724851	13	56.3	83.7	51.8	82.5
chr9	Trf	Intron4	N	103117588	103118143	3	56.5	76.0	47.8	78.3
chr2	A730036I17Rik	Intron4	N	129059522	129060680	6	55.5	80.8	45.9	75.4
chr19	Cyb561a3	Intron4	N	10656987	10659671	12	17.9	33.6	67.0	94.9
chr7	Xylt1	Intron4	N	124735561	124737050	7	78.9	94.1	57.6	85.0
chr5	Ociad2	Intron4	N	73720560	73723342	14	14.3	40.1	11.5	38.9
chr2	Rbbp9	Intron4	N	144374883	144376415	6	70.1	92.3	64.8	91.3
chr9	Dis3l	Intron4	N	64158153	64158248	1	43.9	63.2	46.7	71.4
chr18	Poli	Intron4	N	70681596	70682355	4	48.0	65.1	46.9	71.4
chr6	Stra8	Intron4	N	34880968	34882997	10	43.8	91.6	73.7	97.1
chr2	Slc5a12	Intron4	N	110450350	110456830	27	66.6	91.1	69.5	92.2
chr4	Skint4	Intron4	N	111792723	111797167	19	40.0	71.3	31.3	53.8
chr2	A430105I19Rik	Intron4	N	118584380	118584936	3	61.2	82.8	56.7	79.1
chr3	Fam160a1	Intron4	N	85477402	85479992	13	65.2	94.2	63.6	85.4
chr1	Ccdc19	Intron4	N	174462357	174462661	1	65.7	85.7	65.8	86.5

chr11	Smtnl2	Intron4	N	72215030	72215926	4	44.1	64.3	45.8	66.3
chr17	Zdhhc14	Intron4	N	5712526	5714968	12	65.6	81.5	61.8	81.3
chr10	Arid3a	Intron4	N	79409303	79411604	12	71.9	90.4	53.8	72.9
chr2	Slc52a3	Intron4	N	151833341	151833681	2	33.3	55.3	44.8	63.9
chr14	Pdlim2	Intron4	N	70567852	70571026	16	58.1	88.1	58.7	77.7
chr4	Rcc2	Intron4	N	140266392	140268045	8	69.7	88.2	71.0	90.0
chr10	Midn	Intron4	N	79614469	79616335	9	7.1	52.1	6.0	24.9
chr6	Gstk1	Intron4	N	42197584	42197732	1	44.4	72.5	56.8	75.0
chr16	Mfi2	Intron4	N	31884030	31885029	3	9.8	41.2	17.0	35.1
chr17	2310061I04Rik	Intron4	N	36032318	36032615	1	45.5	80.3	56.2	74.0
chr11	Spag9	Intron4	N	93904981	93909721	23	37.3	62.1	44.9	62.3
chr6	Nr2c2	Intron4	N	92099799	92102308	13	73.7	88.8	72.3	89.5
chr12	Dnajc27	Intron4	N	4096378	4097223	4	70.0	86.4	72.9	90.0
chr15	Arhgap39	Intron4	N	76557259	76557832	3	61.0	79.3	48.8	65.8
chr11	Nos2	Intron4	N	78743373	78745081	9	64.3	88.0	68.4	85.4
chr3	Adam15	Intron4	N	89144860	89145429	3	68.8	84.4	69.6	86.5
chr15	Smpd5	Intron4	N	76126415	76126507	2	70.8	86.1	67.3	82.8
chr6	Clec4f	Intron4	N	83603295	83604676	7	60.4	77.2	80.4	95.7
chr11	Abca9	Intron37	N	110024693	110027210	13	50.0	87.9	51.8	94.3
chr1	Nbeal1	Intron35	N	60334096	60334756	3	84.6	100.0	77.7	93.0
chr7	Muc6	Intron33	N	148838209	148841146	14	72.0	87.7	55.1	82.3
chr1	Kif21b	Intron33	N	138068907	138069379	2	48.6	69.9	33.9	58.3
chr19	Pcnxl3	Intron33	N	5687219	5687450	1	51.4	69.7	55.8	75.9
chr1	Cps1	Intron33	N	67264501	67267076	13	61.9	79.0	56.3	75.0
chr6	Itpr2	Intron32	N	146293753	146295589	9	25.0	45.7	42.6	75.8
chr1	Kif21b	Intron32	N	138068283	138068735	2	55.7	74.3	51.2	66.5
chr17	Dnah8	Intron31	N	30849332	30849978	3	45.4	62.8	46.0	66.5
chr13	Dip2c	Intron30	N	9634137	9636340	11	76.9	96.8	70.2	85.2
chr4	Grhl3	Intron3	N	135104414	135105153	4	28.1	51.4	21.2	62.4
chr5	Upk3b	Intron3	N	136516110	136517631	8	66.6	88.0	47.3	88.3
chr1	Mettl21c	Intron3	N	44070619	44074030	17	34.1	52.5	15.8	53.7
chr6	Atp6v0e2	Intron3	N	48490166	48490426	2	52.5	67.7	38.5	74.3
chr5	Gm3716	Intron3	N	65001395	65001751	2	14.9	48.7	13.9	47.4
chr2	Cacnb4	Intron3	N	52305566	52307388	9	43.0	64.6	55.2	87.5
chr7	Svip	Intron3	N	59259356	59261107	7	40.8	70.3	50.9	80.8
chr17	Mllt4	Intron3	N	13944400	13945938	8	21.2	75.4	65.9	95.5
chr9	Limd1	Intron3	N	123419694	123420333	3	22.6	51.6	24.8	54.2
chr13	Phactr1	Intron3	N	43228430	43229295	4	49.2	80.1	53.7	81.4
chr2	Slc20a1	Intron3	N	129026060	129026235	1	66.1	81.5	46.2	72.9
chr9	Keap1	Intron3	N	21036347	21037831	5	48.9	67.3	48.6	75.2
chr11	Trim65	Intron3	N	115988930	115989011	1	43.9	63.3	33.9	60.2
chr19	Scd2	Intron3	N	44372661	44374098	7	30.7	50.1	19.8	45.0
chr1	Igfn1	Intron3	N	137852435	137853274	4	74.6	89.7	52.6	77.6
chr18	Spink3	Intron3	N	43894889	43896748	8	23.8	56.0	8.9	33.4
chr2	4932418E24Rik	Intron3	N	26131788	26131936	1	46.5	76.0	51.1	75.0
chr5	Pdgfra	Intron3	N	75559878	75562535	13	70.4	89.5	72.1	95.4
chr17	Lta	Intron3	N	35341608	35342103	3	35.6	64.2	34.6	57.7
chr13	Elmo1	Intron3	N	20300044	20301007	5	64.0	96.8	62.6	84.8
chr19	Snx15	Intron3	N	6121523	6121782	1	12.5	44.2	18.1	39.2

chr8	Gas8	Intron3	N	126047036	126047977	5	56.5	87.2	71.5	92.6
chr9	Trim71	Intron3	N	114434240	114471422	180	37.1	67.4	44.2	64.7
chr7	Arnt2	Intron3	N	91411054	91411688	3	31.8	76.9	79.6	100.0
chr18	Svil	Intron3	N	5049493	5054015	23	65.3	81.6	67.0	87.0
chr5	Khk	Intron3	N	31229530	31230815	6	70.0	100.0	77.0	96.4
chr6	Gstk1	Intron3	N	42196939	42197482	3	45.4	65.1	45.3	64.3
chr11	Wipf2	Intron3	N	98752147	98752809	3	64.8	84.1	67.7	86.5
chr17	Soga2	Intron3	N	66695726	66697582	9	61.6	78.7	61.1	79.6
chr11	Havcr1	Intron3	N	46566145	46569630	17	59.5	74.9	74.5	93.0
chr1	Serpinb5	Intron3	N	108768965	108771619	13	73.2	90.7	74.6	92.9
chr14	Bcl2l2	Intron3	N	55503651	55504220	3	61.3	77.8	63.2	81.3
chr2	Yme1l1	Intron3	N	23018184	23020018	9	58.1	77.7	64.6	82.6
chr9	Icam1	Intron3	N	20830303	20830755	2	69.2	84.6	62.1	79.6
chr11	Abcc3	Intron3	N	94212417	94213112	3	65.7	91.3	46.7	63.7
chr15	Fbxo43	Intron3	N	36087793	36091441	18	56.7	78.8	55.9	72.9
chr8	Msr1	Intron3	N	40693493	40696985	17	45.4	62.3	45.9	62.7
chr2	Fam171b	Intron3	N	83695687	83698374	13	56.8	83.1	80.4	96.8
chr10	Gm20125	Intron3	N	16675852	16679253	14	76.7	93.3	83.6	100.0
chr4	Agtrap	Intron3	N	147456486	147458067	8	43.1	64.7	44.3	60.1
chr11	Shroom1	Intron3	N	53277833	53278621	4	62.4	89.4	74.3	90.1
chr10	Vmn2r84	Intron3	N	129826403	129827730	6	50.0	69.6	54.1	69.2
chr2	Capn3	Intron3	N	120307965	120310226	11	27.2	64.6	11.1	26.2
chr10	Sbno2	Intron29	N	79545472	79546985	8	5.1	57.6	17.9	51.3
chr1	Kif21b	Intron29	N	138065901	138066565	3	51.9	74.5	51.5	73.7
chr2	Sec16a	Intron29	N	26297591	26300594	13	31.4	51.6	39.5	55.0
chr12	Kidins220	Intron28	N	25732792	25735959	16	2.8	48.0	5.1	30.4
chr10	Lrp1	Intron28	N	126989366	126989766	2	84.6	100.0	70.5	87.2
chr9	Col6a6	Intron27	N	105665244	105669353	21	54.9	70.4	61.5	96.1
chr7	Abcc6	Intron27	N	53274061	53275507	7	63.6	93.3	58.1	75.5
chr15	Rgs22	Intron27	N	36069663	36070082	1	26.5	42.4	30.8	46.4
chr15	Lrrk2	Intron26	N	91572776	91576128	17	67.5	100.0	71.6	100.0
chr1	Aox3	Intron26	N	58229193	58233317	20	32.4	72.7	35.4	61.1
chr9	Sorl1	Intron26	N	41830533	41832378	9	47.5	64.4	48.7	68.2
chr2	Cep152	Intron26	N	125446127	125446908	4	44.3	71.7	52.8	71.3
chr4	Arhgef10l	Intron25	N	140166480	140167141	3	59.3	79.5	56.1	77.5
chr2	Osbpl6	Intron25	N	76431524	76432820	6	59.7	76.8	57.7	73.9
chr1	Cspp1	Intron25	N	10117645	10120066	12	77.5	96.5	79.6	94.9
chr2	Duox2	Intron24	N	122120416	122120820	2	57.5	83.8	23.8	83.0
chr5	Ube3b	Intron24	N	114862919	114865210	11	56.0	76.1	57.9	77.1
chr2	Duox2	Intron23	N	122119830	122120324	2	69.0	89.4	54.7	88.4
chr11	Itgb4	Intron23	N	115853224	115854368	5	45.4	66.8	47.6	70.0
chr13	Mtr	Intron23	N	12330316	12331915	6	68.8	90.8	72.5	93.5
chr7	Tjp1	Intron23	N	72482033	72485035	15	49.7	97.4	72.6	91.9
chr5	Ube3b	Intron23	N	114862497	114862859	2	63.7	82.0	65.8	84.1
chr2	Atp9a	Intron23	N	168514851	168516465	8	58.1	77.2	56.0	72.8
chr9	Als2cl	Intron22	N	110798390	110799140	4	44.7	64.5	47.9	68.3
chr17	Abca3	Intron22	N	24535554	24536829	6	47.8	77.5	66.2	84.4
chr9	5830418K08Rik	Intron22	N	15151481	15155254	19	67.9	89.5	69.4	86.4
chr7	Muc5ac	Intron21	N	148986222	148986848	3	45.0	61.6	61.1	90.3

chr5	Ncor2	Intron21	N	125517289	125517643	3	80.6	95.8	71.9	100.0
chr7	Trpm5	Intron21	N	150272948	150273605	3	52.8	80.9	45.6	66.6
chr1	Sec16b	Intron21	N	159491108	159491646	3	31.1	46.6	38.0	58.7
chr10	Myo1a	Intron21	N	127153419	127155567	11	67.8	85.8	61.3	81.5
chr11	Atp2a3	Intron21	N	72803050	72805190	12	58.8	79.2	69.3	89.4
chr9	Slc44a2	Intron21	N	21156996	21158125	6	58.8	77.8	59.3	75.5
chr7	Abcc6	Intron21	N	53264249	53267863	18	52.8	73.8	61.4	77.5
chr1	Ankrd44	Intron21	N	54819318	54820488	6	67.6	89.8	62.6	78.3
chr11	Nf1	Intron20	N	79255477	79257270	9	35.6	65.4	35.7	90.7
chr5	Plb1	Intron20	N	32605083	32605679	3	12.9	56.2	15.5	46.2
chr1	Sec16b	Intron20	N	159489653	159490813	6	56.6	86.9	46.1	73.6
chr16	Slc15a2	Intron20	N	36781794	36782357	3	57.7	84.1	56.7	83.1
chr11	Abcc3	Intron20	N	94226544	94228903	12	6.3	35.4	36.6	60.6
chr7	Tjp1	Intron20	N	72474652	72480917	31	80.8	96.5	72.8	93.5
chr1	Pik3c2b	Intron20	N	134988246	134988848	3	44.0	67.0	53.8	72.8
chr8	Lsm1	Intron2	N	26902698	26904148	7	49.5	92.0	31.6	100.0
chr11	Slc13a2	Intron2	N	78211931	78212533	3	41.7	92.4	32.6	95.0
chr2	Duoxa2	Intron2	N	122125942	122126300	2	21.1	81.2	24.1	70.9
chr7	2310010J17Rik	Intron2	N	97275555	97278381	14	1.3	23.7	47.2	90.0
chr5	Zfp513	Intron2	N	31503196	31503955	4	45.1	71.7	47.6	90.3
chr14	Timm23	Intron2	N	33002441	33004527	10	0.0	92.7	46.0	86.6
chr2	Gpsm1	Intron2	N	26178156	26179027	4	58.8	95.7	55.7	95.9
chr11	Dnah2	Intron2	N	69234923	69235063	1	38.0	60.7	31.2	67.3
chr9	Gm5627	Intron2	N	102649949	102651625	8	40.0	60.9	22.1	58.1
chr5	Brap	Intron2	N	122112171	122113304	6	54.5	93.8	52.8	88.8
chr6	Vgll4	Intron2	N	114812898	114813901	5	62.5	86.7	47.4	81.3
chr11	Clec10a	Intron2	N	69981507	69982084	3	42.4	76.3	43.8	76.4
chr19	Ifit2	Intron2	N	34644861	34647556	12	54.7	81.7	49.9	82.2
chr6	Crebl2	Intron2	N	134799319	134801097	9	50.1	82.4	63.1	95.4
chr4	Rcc2	Intron2	N	140258351	140263865	22	48.9	79.9	50.7	82.2
chr2	Pex16	Intron2	N	92216064	92216783	3	1.7	41.5	45.3	76.4
chr7	Mrpl23	Intron2	N	149721033	149721974	5	50.0	71.3	40.0	70.8
chr18	Pmaip1	Intron2	N	66620653	66622923	10	66.3	95.2	67.1	97.5
chr2	Slc20a1	Intron2	N	129025801	129025918	1	79.0	94.7	64.6	94.8
chr17	Bag6	Intron2	N	35273181	35275316	10	48.8	86.0	58.6	88.6
chr11	Tm4sf5	Intron2	N	70323377	70323716	2	22.5	53.2	17.0	46.7
chr2	Fastkd5	Intron2	N	130454372	130455651	5	0.0	34.0	48.1	77.0
chr14	Slc39a2	Intron2	N	52513895	52514130	1	44.4	77.1	41.6	69.1
chr8	Zdhhc2	Intron2	N	41531159	41532404	6	25.0	56.7	34.1	61.4
chr18	Pdgfrb	Intron2	N	61219844	61220858	5	72.5	91.8	66.1	92.9
chr17	Ccnd3	Intron2	N	47731899	47734368	12	21.8	50.5	19.7	46.1
chr3	Spry1	Intron2	N	37539731	37541476	5	13.7	34.5	16.5	42.6
chr3	Gm11548	Intron2	N	36401217	36403213	10	74.3	100.0	74.2	100.0
chr14	Fam107a	Intron2	N	9132181	9133572	7	65.8	84.0	42.4	68.1
chr2	Wfdc3	Intron2	N	164557694	164559679	10	32.8	48.0	30.8	56.4
chr17	Cldn6	Intron2	N	23816789	23818014	5	53.2	80.0	55.0	79.8
chr6	Il17ra	Intron2	N	120422135	120422943	4	56.4	85.2	59.6	83.7
chr5	Speer7-ps1	Intron2	N	15205067	15211265	29	35.8	63.8	38.3	62.4
chr5	AI839979	Intron2	N	31873293	31873556	1	26.3	41.5	26.9	50.7

chr5	Mnx1	Intron2	N	29801457	29804124	11	28.7	45.6	24.5	48.0
chr7	Lrrc28	Intron2	N	74674632	74676500	9	54.4	72.2	50.0	73.3
chr11	Patz1	Intron2	N	3193347	3194458	6	53.8	73.7	61.4	84.7
chr2	Btbd3	Intron2	N	138104994	138105484	2	55.7	79.2	46.7	69.6
chr15	Gm16576	Intron2	N	79573661	79574942	7	37.8	66.6	69.7	92.5
chr10	Gcc2	Intron2	N	57718631	57719567	4	0.0	38.9	0.0	22.6
chr5	Rasl11b	Intron2	N	74592185	74593291	6	36.8	56.1	25.3	47.3
chr1	Dars2	Intron2	N	162973820	162975089	6	46.2	89.1	71.6	93.1
chr12	Ifi27l2b	Intron2	N	104690178	104690680	3	59.1	76.0	67.7	88.5
chr14	Ajuba	Intron2	N	55188382	55189081	4	60.6	81.4	62.3	82.9
chr7	Knop1	Intron2	N	125992089	125994134	10	35.5	68.7	33.6	54.2
chr16	Hira	Intron2	N	18894930	18896546	8	33.3	53.8	73.4	93.9
chr12	Tmem179	Intron2	N	113741532	113742744	6	53.3	68.7	62.5	82.9
chr14	Oxa1l	Intron2	N	54981153	54982965	8	67.5	85.8	70.4	90.8
chr15	Xrcc6	Intron2	N	81847629	81849410	9	80.3	98.2	77.2	97.4
chr11	Pdk2	Intron2	N	94889293	94889792	3	53.8	71.4	49.0	68.9
chr17	Capn11	Intron2	N	45767734	45768736	5	14.0	45.2	32.7	52.5
chr4	Grhpr	Intron2	N	44995882	44996745	4	41.5	59.3	37.6	56.6
chr3	Abca4	Intron2	N	121755914	121757109	6	57.0	80.5	53.9	72.8
chr1	BC055402	Intron2	N	57261453	57263557	11	25.4	66.3	40.8	59.7
chr10	Wisp3	Intron2	N	38873148	38874742	8	71.5	92.0	66.1	84.9
chr12	Fos	Intron2	N	86816137	86816541	1	52.6	70.1	45.8	64.5
chr8	Psmb10	Intron2	N	108460048	108460595	3	10.0	34.7	13.3	31.7
chr16	Zfp597	Intron2	N	3871277	3872058	3	40.9	65.4	42.9	61.3
chr1	Zfp451	Intron2	N	33822755	33826565	18	23.5	65.3	43.3	61.4
chr11	Ehbp1	Intron2	N	21907201	21913486	28	33.0	57.3	37.2	55.1
chr7	Csrp3	Intron2	N	56088026	56089108	5	72.9	96.7	65.3	83.1
chr5	Fbxo21	Intron2	N	118428003	118429749	7	43.9	69.2	49.8	67.3
chr7	Rras	Intron2	N	52275558	52275672	1	36.0	57.1	42.5	60.0
chr5	Plod3	Intron2	N	137464079	137464204	1	38.2	63.2	44.0	61.4
chr11	Wnk4	Intron2	N	101124210	101125082	3	31.1	50.2	28.0	45.1
chr1	Ercc5	Intron2	N	44213958	44214988	5	63.4	95.3	71.1	88.0
chr9	Snx22	Intron2	N	65915388	65915586	2	47.6	65.8	43.6	59.8
chr14	Fbxl3	Intron2	N	103488691	103491520	14	67.6	83.1	70.3	86.3
chr1	Ppp1r12b	Intron2	N	136668626	136670002	7	21.3	67.1	26.1	41.8
chr19	5830416P10Rik	Intron2	N	53519835	53520830	5	28.8	66.3	30.3	46.0
chr11	Arrb2	Intron2	N	70249075	70249664	3	57.5	82.5	67.1	82.8
chr12	Serpina1b	Intron2	N	104967592	104968459	4	38.1	81.8	75.2	90.7
chr4	Cldn19	Intron2	N	118929656	118929756	1	56.1	78.0	72.1	87.5
chr9	Nlrx1	Intron2	N	44062327	44063513	6	58.3	75.0	55.0	70.4
chr16	Ccdc14	Intron2	N	34695730	34696898	6	0.0	29.3	9.3	24.7
chr5	Shh	Intron2	N	28788124	28793000	11	28.4	57.7	34.6	49.9
chr8	Zfp423	Intron2	N	90210444	90211854	7	19.2	83.6	67.4	82.7
chr18	Slc4a9	Intron2	N	36688458	36688869	2	75.0	94.3	74.6	89.7
chr4	Slc25a33	Intron2	N	149123439	149126512	15	67.1	92.1	74.8	89.8
chr11	Brip1	Intron19	N	86012497	86014610	9	61.9	100.0	50.0	100.0
chr2	Pkp4	Intron19	N	59186099	59188550	12	56.6	82.0	54.8	83.6
chr19	Myof	Intron19	N	38009412	38010336	5	52.9	76.0	58.0	80.4
chr1	Ptprc	Intron19	N	139990880	139996059	21	70.0	85.5	65.2	85.6

chr1	Igsf9	Intron19	N	174427878	174428127	1	74.3	94.9	76.1	91.9
chr7	Mical2	Intron18	N	119490423	119492907	12	64.0	88.8	68.8	96.6
chr8	Gpr124	Intron18	N	28231009	28231237	1	50.0	73.0	44.0	71.0
chr2	Madd	Intron18	N	91003071	91003655	3	34.1	55.4	38.2	64.9
chr8	Nek5	Intron18	N	23231341	23234015	13	64.4	96.5	74.1	92.1
chr10	Ankrd24	Intron18	N	81109179	81109537	2	56.1	72.9	58.3	75.2
chr17	Thada	Intron18	N	84822530	84825478	14	42.6	80.4	60.6	77.2
chr2	Prpf6	Intron17	N	181384936	181385790	4	12.7	66.7	14.5	46.3
chr9	Stt3a	Intron17	N	36572766	36575134	9	45.7	61.4	61.6	84.8
chr16	Dlg1	Intron17	N	31838313	31842855	23	66.7	91.5	72.5	92.2
chr17	C3	Intron17	N	57354613	57355151	3	55.7	71.4	54.0	71.9
chr1	Epha4	Intron17	N	77508326	77511515	8	16.1	52.9	58.7	76.1
chr18	Aldh7a1	Intron17	N	56720484	56721686	6	43.3	71.8	55.1	72.3
chr9	Xylb	Intron17	N	119297887	119299547	8	53.5	74.2	49.8	64.9
chr8	Glg1	Intron16	N	113702887	113704834	10	31.9	86.0	52.0	95.6
chr5	9330182L06Rik	Intron16	N	9445554	9446893	7	46.9	65.6	49.6	77.0
chr9	Sema3b	Intron16	N	107506532	107507299	4	40.1	61.1	38.3	65.3
chr14	Cdhr1	Intron16	N	37910544	37911274	3	41.3	67.9	52.6	75.3
chr6	Ccdc37	Intron16	N	90377538	90378372	4	8.3	37.9	27.1	47.2
chr6	Srgap3	Intron16	N	112725715	112727013	6	45.5	96.3	73.9	92.0
chr14	Ndrg2	Intron15	N	52531222	52532997	6	30.6	53.3	37.2	65.0
chr9	Nek11	Intron15	N	105295491	105297525	10	9.8	50.3	29.2	55.4
chr1	Sgk3	Intron15	N	9876224	9878950	14	29.4	96.2	40.6	63.3
chr6	Cftr	Intron15	N	18220327	18223698	16	45.7	63.4	50.8	69.9
chr11	Camta2	Intron15	N	70495271	70495962	3	39.4	71.4	44.1	62.4
chr14	Piwil2	Intron15	N	70809193	70810188	5	33.3	83.3	70.2	86.1
chr1	Glb1l	Intron14	N	75205413	75205630	1	15.9	37.8	15.6	42.0
chr14	Ero1l	Intron14	N	45925008	45926186	6	52.2	76.5	54.7	76.4
chr10	Itga7	Intron14	N	128383459	128383854	2	31.1	73.9	61.4	80.4
chr1	Npas2	Intron14	N	39391658	39392839	6	16.1	79.8	62.2	80.6
chr5	Pan3	Intron14	N	148348117	148350818	14	60.9	86.9	71.2	89.3
chr10	Rfx6	Intron14	N	51441425	51442806	7	12.6	48.6	9.8	25.2
chr11	Rpa1	Intron14	N	75153278	75153791	3	71.5	93.4	78.9	94.1
chr11	Vps54	Intron14	N	21206551	21206911	2	30.0	61.9	37.0	52.1
chr13	Phactr1	Intron13	N	43228430	43229295	4	49.2	80.1	53.7	81.4
chr10	Dctn2	Intron13	N	126715279	126715372	1	69.6	95.5	65.0	88.6
chr5	Gpr133	Intron13	N	129648411	129649841	7	52.6	74.0	54.6	77.1
chr2	Lpin3	Intron13	N	160727613	160728298	3	63.8	100.0	66.2	88.6
chr11	Zmynd15	Intron13	N	70279002	70279416	1	46.6	62.4	42.1	64.2
chr14	Bmp1	Intron13	N	70904605	70905138	3	53.9	95.4	63.9	84.9
chr13	Tert	Intron13	N	73784287	73785394	6	70.8	94.9	76.8	96.3
chr11	Mybbp1a	Intron13	N	72259760	72260473	4	73.9	91.2	79.2	98.4
chr7	Mvp	Intron13	N	134145190	134145322	1	65.6	82.0	76.5	94.9
chr9	Kirrel3	Intron13	N	34832732	34835916	16	67.5	90.5	63.7	81.7
chr17	Tiam2	Intron13	N	3453413	3454087	3	56.1	91.4	76.8	94.5
chr3	Dclk2	Intron13	N	86634794	86635654	4	66.6	83.8	72.4	89.3
chr15	D15Ertd621e	Intron13	N	58264183	58264448	1	42.3	74.3	39.7	56.6
chr13	Ssbp2	Intron13	N	91828472	91830464	10	48.5	76.4	51.5	66.8
chr18	Dsg4	Intron12	N	20621185	20623317	11	23.5	49.5	23.4	95.2

chr4	Ift74	Intron12	N	94327680	94329277	7	5.1	44.3	11.1	68.6
chr8	Tacc1	Intron12	N	26310679	26311593	1	45.4	66.9	42.5	75.1
chr4	Wdr65	Intron12	N	118261743	118265602	19	54.5	86.7	62.6	90.1
chr11	Slc38a10	Intron12	N	119999296	120000299	5	72.5	92.5	56.1	80.6
chr1	Pign	Intron12	N	107484649	107485625	5	48.6	72.1	67.1	90.4
chr3	Chd1l	Intron12	N	97391145	97392162	5	73.6	91.0	56.9	79.9
chr8	Galns	Intron12	N	125128114	125129039	5	66.4	88.4	67.6	90.5
chr12	Exoc3l4	Intron12	N	112667748	112668879	2	17.7	44.0	20.2	42.8
chr6	Slc6a11	Intron12	N	114194905	114195599	3	51.0	73.4	57.3	79.0
chr9	Snx1	Intron12	N	65952744	65953327	3	21.8	45.2	28.3	49.5
chr9	Vipr1	Intron12	N	121577919	121578491	2	15.4	61.2	25.1	46.2
chr3	D930015E06Rik	Intron12	N	83727020	83727909	4	66.4	84.2	68.3	89.3
chr11	B3gntl1	Intron12	N	121532488	121534298	7	76.2	96.8	66.1	86.0
chr14	Hacl1	Intron12	N	32444225	32446081	9	72.2	100.0	55.1	74.5
chr10	Tjp3	Intron12	N	80742317	80742638	2	37.4	65.8	48.9	67.7
chr17	Foxp4	Intron12	N	48015704	48017211	5	36.7	63.8	42.3	60.6
chr2	Ttc17	Intron12	N	94202935	94204422	6	79.3	96.8	79.8	97.5
chr4	Rad54b	Intron12	N	11537713	11539518	9	62.9	81.3	70.8	87.9
chr2	Slc9a8	Intron12	N	167293268	167294463	6	69.6	94.4	73.1	89.6
chr11	Itgb4	Intron12	N	115846413	115847930	8	71.3	89.3	71.0	87.3
chr11	Grb10	Intron12	N	11851663	11854746	15	43.2	63.0	54.2	69.9
chr11	Alox15	Intron12	N	70163909	70164215	2	51.7	69.2	56.3	71.9
chr4	Rap1gap	Intron11	N	137273710	137273829	1	62.0	86.2	49.7	78.4
chr4	Plk3	Intron11	N	116805420	116805509	1	61.6	89.3	61.6	89.7
chr4	Casz1	Intron11	N	148313432	148315390	8	69.4	90.1	60.7	86.5
chr6	Dysf	Intron11	N	84020198	84020872	3	43.2	64.2	47.3	72.7
chr4	Rps6ka1	Intron11	N	133419986	133420939	5	40.0	70.4	47.9	72.4
chr2	Src	Intron11	N	157293347	157294218	4	40.0	68.5	68.7	90.5
chr7	Anpep	Intron11	N	86981293	86983127	9	41.3	82.5	71.8	92.2
chr7	Far1	Intron11	N	120705020	120709898	23	57.2	73.4	57.1	77.3
chr14	Cdh24	Intron11	N	55258499	55260057	6	53.6	78.0	53.4	72.8
chr12	Scin	Intron11	N	40808362	40810871	13	58.2	73.5	58.4	77.6
chr2	Gyltl1b	Intron11	N	92210141	92210333	1	52.9	75.6	60.2	78.7
chr5	Nrbp1	Intron11	N	31552055	31552261	1	70.0	90.4	77.8	95.2
chr9	Anxa2	Intron11	N	69335921	69337458	8	55.3	81.3	57.7	75.1
chr6	St7	Intron11	N	17854970	17856407	7	46.0	62.4	61.4	78.2
chr10	Hmha1	Intron11	N	79487597	79488153	3	41.3	65.1	44.6	60.8
chr11	Evpl	Intron11	N	116091438	116091729	1	43.0	66.3	38.3	54.3
chr6	Wee2	Intron11	N	40414282	40415814	8	9.5	31.0	75.0	90.9
chr17	Fam179a	Intron11	N	72054182	72055118	5	20.7	40.9	30.0	45.8
chr10	Tbc1d15	Intron11	N	114657465	114658638	6	51.6	89.6	72.9	87.9
chr12	Rps6kl1	Intron10	N	86490859	86492112	4	16.7	68.4	27.3	72.1
chr17	Abhd16a	Intron10	N	35237344	35237583	1	23.6	48.6	20.8	54.1
chr15	Gga1	Intron10	N	78719541	78720145	3	37.9	67.2	26.8	59.6
chr4	Cyp4x1	Intron10	N	114799211	114800045	4	64.4	81.7	58.6	90.0
chr15	Col22a1	Intron10	N	71650515	71652359	9	69.6	91.5	67.3	96.8
chr2	Grb14	Intron10	N	64774549	64776425	9	46.7	70.0	49.0	76.6
chr4	Lck	Intron10	N	129234700	129234869	1	63.8	93.2	62.8	90.1
chr10	Stat6	Intron10	N	127090129	127090262	1	69.2	85.9	62.4	88.5

chr9	Vps13c	Intron10	N	67724094	67725903	9	64.4	85.8	62.6	88.6
chr14	Fam35a	Intron10	N	35122281	35123352	4	55.7	71.0	52.3	76.8
chr1	Vil1	Intron10	N	74469655	74470248	3	63.2	85.9	62.3	85.0
chr1	Acsl3	Intron10	N	78693605	78695570	10	60.4	75.5	60.3	82.5
chr6	Calcr	Intron10	N	3664762	3667091	12	65.7	93.9	64.7	84.8
chr15	Nrbp2	Intron10	N	75920096	75920177	1	61.3	84.8	65.4	84.6
chr11	Dnah2	Intron10	N	69242995	69243975	5	16.1	95.2	45.2	63.5
chr12	Ppp1r13b	Intron10	N	113079309	113081755	12	43.1	62.8	45.1	63.3
chr19	Pde6c	Intron10	N	38233049	38234202	6	59.2	77.4	55.4	73.0
chr2	D630003M21Rik	Intron10	N	158035959	158037355	7	47.6	65.2	45.0	62.1
chr9	Sema7a	Intron10	N	57805513	57808061	13	66.4	86.6	67.4	84.2
chr7	Syce1	Intron10	N	147966431	147966773	2	59.5	75.5	45.9	62.5
chr11	Stat5b	Intron10	N	100658020	100658377	2	63.6	85.3	67.7	84.2
chr12	Lrrc9	Intron10	N	73561910	73564311	12	40.1	63.3	48.7	63.9
chr5	Grid2ip	Intron10	N	144142183	144143261	5	59.2	85.0	58.3	73.5
chr10	Gls2	Intron10	N	127641784	127641916	1	36.4	62.1	46.7	61.8
chr1	Mptx1	Intron1	N	176260768	176262324	8	43.5	97.1	35.0	94.7
chr4	Cdkn2c	Intron1	N	109334232	109337622	17	18.0	88.2	34.0	88.1
chr15	Fam83h	Intron1	N	75835180	75835370	1	50.0	65.0	31.8	81.6
chr2	Abcb11	Intron1	N	69077342	69081471	21	6.7	32.8	6.0	46.7
chr10	1600002K03Rik	Intron1	N	79635920	79636953	3	0.0	90.7	45.3	83.9
chr8	Rhou	Intron1	N	126178117	126179391	4	48.4	74.3	41.2	78.1
chr18	Pmaip1	Intron1	N	66618467	66620496	6	44.0	73.9	37.3	73.8
chr1	Cnot11	Intron1	N	39593183	39594260	4	33.0	63.1	33.9	67.9
chr11	Dlx4	Intron1	N	95002784	95003102	2	58.3	75.0	57.1	90.9
chr19	5830416P10Rik	Intron1	N	53518038	53519739	9	45.4	86.9	56.5	89.3
chr6	Vwf	Intron1	N	125503218	125504470	7	40.2	63.6	21.0	53.0
chr5	Mrpl1	Intron1	N	96639213	96642805	16	27.0	52.4	32.5	64.0
chr17	Slc22a1	Intron1	N	12841951	12843734	9	59.1	88.5	28.3	59.4
chr9	Bcl9l	Intron1	N	44307357	44307530	1	47.5	71.8	49.2	79.8
chr9	9230112J17Rik	Intron1	N	60373772	60374189	2	46.5	74.0	43.0	72.4
chr15	Sh3bp1	Intron1	N	78730392	78731575	4	27.0	65.7	29.4	58.7
chr8	4933405L10Rik	Intron1	N	108232416	108232695	2	60.2	93.9	62.6	90.8
chr4	Cdkn2b	Intron1	N	88953224	88956574	14	12.5	43.4	9.8	37.7
chr11	Ehbp1	Intron1	N	21906875	21907085	1	54.6	90.9	61.2	88.2
chr19	Hhex	Intron1	N	37509724	37511678	7	41.7	74.2	49.2	76.0
chr1	Ormdl1	Intron1	N	53354033	53355815	7	34.2	56.8	71.0	97.6
chr4	Acot11	Intron1	N	106421090	106421866	5	57.2	89.4	65.3	91.9
chr13	Irx4	Intron1	N	73398325	73402295	9	26.5	61.5	32.6	57.9
chr13	Wrnip1	Intron1	N	32894914	32897206	10	28.5	74.7	52.0	77.1
chr19	Klc2	Intron1	N	5108689	5109102	2	12.8	56.5	11.4	36.4
chr2	Duoxa2	Intron1	N	122124924	122125883	4	27.5	69.9	28.9	52.9
chr13	Hist1h2bp	Intron1	N	21879761	21880892	6	57.8	96.3	52.1	76.1
chr10	Ascc1	Intron1	N	59465656	59467542	6	7.0	52.7	34.2	57.9
chr2	Bmi1	Intron1	N	18599097	18603419	15	34.3	58.7	49.4	73.0
chr2	Catsper2	Intron1	N	121220224	121222688	12	49.7	66.5	14.6	37.6
chr16	Aifm3	Intron1	N	17489985	17491385	5	60.0	77.7	60.1	83.0
chr11	Wdr81	Intron1	N	75255592	75257756	11	64.3	79.9	63.2	85.5
chr7	1810026B05Rik	Intron1	N	80689269	80694482	25	46.2	67.0	73.5	95.5

chr1	Adamts4	Intron1	N	173181601	173182667	5	32.0	48.8	26.5	48.4
chr10	Rab32	Intron1	N	10266250	10270476	21	32.3	65.6	55.6	77.3
chr5	Acad12	Intron1	N	122048972	122049203	1	70.3	91.0	68.1	89.7
chr16	Tnp2	Intron1	N	10788187	10788357	1	64.4	85.9	71.4	92.9
chr11	Atp6v0a1	Intron1	N	100870879	100873070	8	34.0	60.3	27.3	48.8
chr11	Pctp	Intron1	N	89846071	89847406	7	12.8	60.1	40.4	61.8
chr19	Sec31b	Intron1	N	44591979	44592129	1	59.2	79.5	63.9	85.1
chr17	Dync2li1	Intron1	N	85025917	85027624	7	53.5	70.0	40.3	61.4
chr14	Arhgef40	Intron1	N	52604667	52607077	7	45.5	61.5	50.4	71.4
chr2	Pacsin3	Intron1	N	91097084	91100024	12	47.9	76.8	50.7	71.5
chr11	Gm11961	Intron1	N	4525507	4526421	5	70.5	88.9	75.4	96.2
chr6	Hoxa3	Intron1	N	52120742	52122119	5	69.9	90.1	65.0	85.7
chr11	Hes7	Intron1	N	68934018	68935011	2	7.1	48.0	16.9	37.5
chr11	Hand1	Intron1	N	57643209	57644745	6	66.2	81.8	63.1	83.4
chr15	Fam109b	Intron1	N	82171702	82173488	8	34.8	79.3	52.0	72.3
chr7	Pkp3	Intron1	N	148264334	148265804	8	65.8	83.5	49.0	69.0
chr3	Sfrp2	Intron1	N	83570966	83573247	11	49.8	68.2	43.7	63.5
chr19	Snx15	Intron1	N	6119955	6120485	3	29.6	51.0	26.7	46.5
chr7	Tpcn2	Intron1	N	152440604	152441355	4	65.7	88.5	70.3	90.0
chr5	Pdx1	Intron1	N	148082224	148085955	18	43.9	60.2	37.5	57.1
chr4	Mxra8	Intron1	N	155214000	155214886	4	41.6	64.3	52.1	71.5
chr14	Jph4	Intron1	N	55727233	55728122	4	45.8	69.2	27.5	46.6
chr11	Pemt	Intron1	N	59784378	59784685	2	61.8	94.0	65.2	83.8
chr16	Upk1b	Intron1	N	38774259	38776299	10	63.4	85.5	74.1	92.6
chr6	Tmem52b	Intron1	N	129463123	129464231	6	0.0	37.9	77.4	95.7
chr8	Mtss1l	Intron1	N	113245691	113250093	18	61.8	82.5	62.5	80.7
chr4	Eno1	Intron1	N	149611450	149613581	8	8.8	38.8	14.8	32.9
chr17	Gm7325	Intron1	N	45738635	45738959	2	18.3	36.7	19.7	37.7
chr6	Snrpg	Intron1	N	86321609	86325670	18	44.9	63.1	39.6	57.7
chr3	Wls	Intron1	N	159503012	159535872	143	46.3	64.6	46.2	64.2
chr14	Akap11	Intron1	N	78895754	78898641	13	79.2	94.4	73.3	91.3
chr16	5-Sep	Intron1	N	18622890	18623007	2	64.1	89.1	67.8	85.7
chr3	Nes	Intron1	N	87775911	87776913	3	16.8	41.8	31.6	49.6
chr7	Gm15413	Intron1	N	103941338	103944027	12	40.5	58.3	61.7	79.6
chr7	Ppp1r14a	Intron1	N	30074555	30076528	8	69.5	95.8	72.5	90.4
chr5	4930568K20Rik	Intron1	N	124680035	124680474	2	30.8	56.3	34.1	51.7
chr11	Slc47a1	Intron1	N	61158031	61162160	21	64.1	79.1	57.7	75.2
chr11	Stat3	Intron1	N	100750075	100750791	5	45.8	73.9	61.1	78.5
chr10	1810043G02Rik	Intron1	N	77441527	77442497	3	25.3	40.8	32.8	50.2
chr5	2410018M08Rik	Intron1	N	130371723	130375052	15	41.1	71.2	57.6	74.5
chr18	4930592I03Rik	Intron1	N	83088623	83089542	5	76.4	94.2	72.5	89.4
chr4	Tlr4	Intron1	N	66488956	66494927	28	21.1	57.1	56.8	73.5
chr5	Hpd	Intron1	N	123622098	123622178	1	22.4	47.8	11.8	28.4
chr14	Ccdc25	Intron1	N	66456322	66459284	12	2.7	61.7	45.0	61.6
chr4	Myom3	Intron1	N	135315722	135318416	13	61.6	82.7	64.0	80.4
chr7	Th	Intron1	N	150079096	150079912	4	37.1	71.2	49.8	66.1
chr16	Morc1	Intron1	N	48431516	48437387	28	66.8	83.2	71.2	87.5
chr17	Yipf4	Intron1	N	74889135	74891685	11	53.6	79.2	43.0	59.2
chr4	Fam46b	Intron1	N	133036479	133041998	23	51.3	74.8	50.3	66.4

chr10	Smpdl3a	Intron1	N	57514591	57520716	28	48.3	70.7	30.8	46.9
chr19	Cabp2	Intron1	N	4082868	4084877	12	64.5	81.6	67.2	83.1
chr13	C230035I16Rik	Intron1	N	23519922	23520173	1	79.4	97.1	78.7	94.4
chr7	Gm9999	Intron1	N	54232077	54238951	34	51.2	69.4	64.2	79.5
chr7	4933406J10Rik	Intron1	N	89892294	89892983	3	45.3	72.0	62.7	78.0
chr1	Stat4	Intron1	N	52065573	52068626	16	27.8	48.0	20.8	36.0
chr10	Nfic	Intron1	N	80863817	80867568	20	61.6	77.6	40.0	55.2
chr1	Hmcn1	Exon90	N	152603653	152603748	1	50.9	76.0	54.7	71.6
chr9	Zmynd10	Exon9	N	107452829	107452950	2	43.1	81.0	46.4	78.9
chr4	Fgr	Exon9	N	132553883	132553959	1	16.0	63.2	31.3	59.6
chr2	Pdk1	Exon9	N	71735704	71735817	1	62.5	91.4	67.5	90.5
chr14	Piwil2	Exon9	N	70797951	70798091	1	40.2	67.5	28.4	50.7
chr3	Fndc7	Exon9	N	108684244	108684504	1	46.6	84.2	51.4	73.1
chr17	Ddr1	Exon9	N	35825731	35825814	1	58.1	82.6	66.7	88.4
chr11	Pitpna	Exon9	N	75438967	75439032	2	56.6	75.9	60.9	80.7
chr15	Nrbp2	Exon9	N	75920040	75920095	2	61.3	84.8	65.4	84.6
chr10	Adarb1	Exon9	N	76822312	76822480	2	28.6	48.9	10.5	27.7
chr9	Vwa5a	Exon9	N	38535579	38535723	1	38.5	54.2	56.4	73.3
chr11	Myh13	Exon9	N	67151143	67151281	1	68.3	87.5	71.0	87.5
chr15	Dennd3	Exon9	N	73373150	73373246	1	44.0	60.0	46.2	61.2
chr2	Src	Exon8	N	157290365	157290514	1	47.8	72.6	37.0	72.7
chr12	Ptprn2	Exon8	N	118114463	118114845	2	23.8	58.7	58.2	88.3
chr7	Sigirr	Exon8	N	148280681	148281216	4	17.5	52.0	22.0	50.5
chr14	Sorbs3	Exon8	N	70591240	70591382	2	23.0	50.1	17.8	41.5
chr5	Pcgf3	Exon8	N	108924853	108924990	1	69.8	87.8	60.7	82.9
chr11	Wnk4	Exon8	N	101130376	101130434	1	70.0	86.8	71.9	92.9
chr4	Grhl3	Exon8	N	135110428	135110522	1	16.9	41.7	18.1	38.0
chr5	Agfg2	Exon8	N	138105444	138105600	1	50.0	84.1	48.5	67.7
chr5	Mlxipl	Exon8	N	135608185	135608731	3	57.3	80.5	54.4	73.6
chr4	Acot11	Exon8	N	106430911	106431030	1	48.9	68.0	52.2	71.2
chr6	Gnb3	Exon8	N	124789682	124789768	2	23.0	56.9	45.1	63.5
chr8	Ces2e	Exon8	N	107455742	107455886	1	45.7	66.7	61.1	78.6
chr18	Zfp608	Exon8	N	55147265	55148360	3	3.6	33.3	9.4	26.5
chr18	Megf10	Exon8	N	57412488	57412700	1	30.8	48.9	25.9	42.2
chr16	Prkdc	Exon75	N	15816844	15816987	1	53.4	91.5	57.1	79.7
chr16	Mgrn1	Exon7	N	4918866	4918913	1	38.5	72.0	29.0	65.7
chr8	Col4a2	Exon7	N	11402163	11402234	1	22.7	75.3	18.4	47.0
chr7	Myh14	Exon7	N	51870715	51870876	1	56.4	78.7	58.4	84.4
chr5	Mlxipl	Exon7	N	135604383	135604567	1	58.2	89.9	66.7	92.0
chr17	Rgs11	Exon7	N	26342053	26342134	1	71.9	94.3	67.1	90.7
chr11	Card14	Exon7	N	119187369	119187494	1	77.4	95.6	73.0	95.3
chr4	Serinc2	Exon7	N	129941505	129941695	1	51.5	71.7	54.4	76.3
chr7	Ap3b2	Exon7	N	88609517	88609697	1	32.2	55.4	40.5	60.2
chr4	Padi2	Exon7	N	140488662	140488765	1	57.5	76.6	61.7	81.1
chr3	Larp7	Exon7	N	127249394	127249487	2	53.1	89.7	61.9	80.9
chr4	Foxj3	Exon7	N	119291889	119292069	1	60.7	82.2	67.2	85.4
chr4	Catsper4	Exon7	N	133777435	133777536	1	61.2	79.2	58.3	76.3
chr10	Mfsd12	Exon7	N	80825678	80825772	2	45.3	60.6	39.9	57.5
chr5	Acacb	Exon7	N	114642083	114642193	1	37.2	61.9	41.5	56.9

chr8	Zdhhc1	Exon7	N	108000917	108001018	1	58.1	75.3	58.8	74.1
chr7	Myh14	Exon6	N	51869663	51869791	1	34.7	82.1	42.6	75.3
chr1	Sec16b	Exon6	N	159463210	159463309	1	63.3	91.3	57.4	85.7
chr15	St3gal1	Exon6	N	66960115	66960169	2	43.8	64.8	51.5	79.0
chr17	Runx2	Exon6	N	44951118	44951241	2	41.5	60.5	40.9	65.7
chr7	Ush1c	Exon6	N	53457003	53457044	1	59.6	82.9	58.9	81.8
chr2	Nr4a2	Exon6	N	56965362	56965483	2	64.4	84.7	65.2	85.6
chr17	Dennd1c	Exon6	N	57209536	57209607	1	61.7	88.1	62.7	82.1
chr4	Phc2	Exon6	N	128389303	128389603	2	28.4	59.3	26.5	45.3
chr4	Pink1	Exon6	N	137876653	137876937	1	53.2	73.4	50.0	68.3
chr7	Phldb3	Exon6	N	25404401	25404484	1	26.3	52.0	32.8	50.8
chr17	Arid1b	Exon6	N	5279203	5279382	1	66.4	85.9	72.0	89.9
chr2	Ptpra	Exon6	N	130357825	130357915	1	69.4	84.6	59.5	76.9
chr1	Stk16	Exon6	N	75210151	75210272	1	78.3	100.0	83.3	100.0
chr17	Smoc2	Exon6	N	14485711	14485785	1	37.2	68.4	58.8	75.4
chr12	Numb	Exon6	N	85152236	85152310	1	60.3	84.6	72.2	88.4
chr7	Tmc5	Exon6	N	125783434	125783521	1	10.0	26.7	25.0	40.9
chr7	Ate1	Exon6	N	137650996	137651213	1	64.7	82.6	66.2	81.7
chr9	Vps13c	Exon56	N	67793631	67793895	1	49.7	66.7	42.8	65.2
chr18	Gnpda1	Exon5	N	38497745	38497876	1	11.8	46.7	10.5	48.8
chr2	Prdm11	Exon5	N	92853922	92854046	2	11.4	40.8	20.9	57.1
chr12	Fam228b	Exon5	N	4775196	4775327	2	42.5	90.5	63.3	95.7
chr5	Atraid	Exon5	N	31356334	31356431	2	52.6	87.1	54.6	86.7
chr10	Tac2	Exon5	N	127166170	127166223	1	12.5	58.1	30.2	60.0
chr7	Flt3l	Exon5	N	52390492	52390545	1	36.1	78.6	28.6	57.4
chr17	Mocs1	Exon5	N	49588760	49588871	1	48.3	80.9	47.0	75.0
chr7	Ilk	Exon5	N	112889474	112889557	1	57.3	77.6	56.5	82.2
chr1	Lamc2	Exon5	N	154977780	154977929	1	41.1	76.7	56.9	79.9
chr1	Als2cr12	Exon5	N	58724573	58724672	1	53.8	70.2	42.7	64.9
chr4	Faah	Exon5	N	115673709	115673808	1	68.2	88.6	59.7	81.8
chr2	Rapgef4	Exon5	N	71979210	71979229	1	56.6	74.7	64.6	85.6
chr19	Exoc6	Exon5	N	37651426	37651630	1	25.7	52.5	20.5	39.9
chr13	Slc25a48	Exon5	N	56566316	56566449	1	39.1	71.4	49.6	68.6
chr8	Dync1li2	Exon5	N	106949859	106949970	1	65.1	89.5	73.6	92.1
chr15	Lmbr1l	Exon5	N	98738325	98738402	1	37.3	57.7	30.2	48.5
chr11	Ttll6	Exon5	N	96000944	96001087	1	36.6	63.2	28.9	46.4
chr2	Bpi	Exon5	N	158093467	158093530	1	56.5	84.0	72.7	90.0
chr1	Pm20d1	Exon5	N	133699297	133699416	1	50.0	78.2	58.2	75.4
chr6	Cpa2	Exon5	N	30499242	30499340	1	56.8	77.7	62.5	79.3
chr18	Spink5	Exon5	N	44140786	44140849	1	56.7	80.9	51.4	67.7
chr15	Tmem184b	Exon5	N	79200328	79200418	2	49.2	66.3	43.8	59.9
chr13	Erbb2ip	Exon5	N	104620268	104620411	1	39.6	75.6	66.7	82.5
chr15	Apobec3	Exon5	N	79735874	79736030	1	55.6	75.9	57.6	73.0
chr9	Dock6	Exon48	N	21651629	21651716	1	42.5	79.4	33.9	74.8
chr4	Tceb3	Exon4	N	135564961	135565058	1	42.9	92.0	31.4	87.5
chr11	Trim7	Exon4	N	48661527	48661642	1	50.0	79.4	34.9	76.4
chr4	Epb4.1l4b	Exon4	N	57086134	57086243	1	38.2	62.0	32.8	65.1
chr8	BC068157	Exon4	N	4216182	4216555	1	35.8	63.0	39.0	67.9
chr14	Ecd	Exon4	N	21148105	21148211	1	18.1	55.4	32.9	61.7

chr4	Yipf1	Exon4	N	107009042	107009129	1	61.9	80.0	52.3	78.8
chr4	Clic4	Exon4	N	134794777	134794886	1	26.6	47.4	35.0	60.6
chr19	Aldh1a7	Exon4	N	20782640	20782824	1	55.1	73.6	55.1	80.3
chr9	Slc24a1	Exon4	N	64778987	64779078	1	62.2	87.2	66.3	90.8
chr6	Foxp1	Exon4	N	98894613	98894714	1	48.1	65.2	43.0	66.0
chr2	Gata5	Exon4	N	180067001	180067176	2	65.5	83.3	64.3	86.8
chr19	Ostf1	Exon4	N	18664891	18664998	1	31.3	48.6	26.8	49.3
chr16	5-Sep	Exon4	N	18624204	18624305	1	60.9	90.6	64.6	86.4
chr17	Ccdc64b	Exon4	N	23802909	23803055	1	64.4	85.7	65.3	86.6
chr2	Macrod2	Exon4	N	140849761	140849877	1	47.9	69.2	51.9	73.1
chr17	Tfeb	Exon4	N	47924975	47925095	1	58.5	80.8	55.3	75.9
chr13	Jarid2	Exon4	N	44969652	44969821	1	16.4	42.0	18.8	37.9
chr5	Fndc4	Exon4	N	31597379	31597494	1	34.7	62.8	33.6	52.3
chr7	Isoc2b	Exon4	N	4802995	4803135	2	45.3	66.3	53.2	71.5
chr15	9130401M01Rik	Exon4	N	57863539	57863634	1	61.7	91.2	63.1	81.3
chr2	Nutm1	Exon4	N	112093659	112093787	1	35.0	62.4	35.7	53.8
chr5	C530008M17Rik	Exon4	N	77283960	77284142	1	64.0	88.3	59.3	76.8
chr19	Pten	Exon4	N	32874351	32874589	1	32.4	73.5	50.7	67.6
chr11	Gm11201	Exon4	N	78844363	78844642	1	17.5	39.5	24.6	41.4
chr3	Ppa2	Exon4	N	132993357	132993476	1	71.2	89.0	58.4	75.2
chr2	Vapb	Exon4	N	173601613	173601789	1	52.2	78.1	55.7	72.3
chr9	Snx22	Exon4	N	65916493	65916597	1	46.9	63.4	52.0	68.5
chr17	Foxp4	Exon4	N	48011584	48011685	1	64.0	80.7	59.8	76.0
chr4	Slc2a5	Exon4	N	149500235	149500395	1	54.8	80.7	48.0	64.1
chr5	Scfd2	Exon4	N	74793679	74793928	1	75.2	91.3	63.0	78.9
chr7	Flt3l	Exon4	N	52389743	52389886	1	54.8	77.0	61.2	76.7
chr11	Shroom1	Exon4	N	53278998	53279546	3	69.2	88.5	70.2	85.6
chr3	Lrba	Exon38	N	86343825	86343971	1	44.7	68.1	48.5	73.4
chr8	Fanca	Exon33	N	125830325	125830388	1	69.2	88.9	78.6	95.6
chr2	Myh7b	Exon32	N	155456747	155457096	2	48.6	68.6	48.8	71.0
chr2	Chd6	Exon32	N	160864930	160865079	1	55.0	72.8	60.3	78.2
chr14	Dock9	Exon30	N	122024378	122024467	1	65.1	100.0	54.2	87.5
chr15	Nrbp2	Exon3	N	75917951	75918004	1	10.5	48.2	14.4	50.0
chr10	Dctn2	Exon3	N	126711770	126711866	1	34.8	76.7	45.1	76.6
chr5	Wbscr25	Exon3	N	135472429	135472539	1	17.4	50.0	31.3	62.2
chr16	Pcyt1a	Exon3	N	32462087	32462203	1	51.4	71.7	50.8	76.8
chr9	Icam1	Exon3	N	20830756	20831043	1	72.7	89.5	63.8	88.8
chr7	Adck4	Exon3	N	28018960	28019026	1	56.8	79.3	59.7	80.4
chr13	Cetn3	Exon3	N	81930955	81931146	1	35.0	53.9	29.0	49.4
chr4	Ccdc171	Exon3	N	83195507	83195681	1	57.2	72.9	58.2	78.6
chr4	Sytl1	Exon3	N	132811649	132811727	1	78.0	95.5	73.7	93.9
chr7	Muc2	Exon3	N	148879035	148879128	1	37.8	56.9	40.0	60.0
chr16	Rogdi	Exon3	N	5010133	5010246	1	78.9	95.6	72.2	92.2
chr17	C3	Exon3	N	57344720	57344803	1	51.1	73.9	66.9	86.4
chr9	Rassf1	Exon3	N	107460101	107460398	1	55.3	75.0	59.4	77.0
chr19	Entpd7	Exon3	N	43778689	43778894	1	11.3	29.1	11.3	28.1
chr3	Manba	Exon3	N	135174767	135174937	1	50.5	67.7	45.2	62.0
chr1	Ccdc19	Exon3	N	174462212	174462356	1	35.8	53.1	35.8	51.8
chr10	Gstt4	Exon3	N	75283976	75284063	1	52.0	73.9	55.7	71.7

chr14	Fermt2	Exon3	N	46084399	46084620	1	62.0	82.8	53.4	69.2
chr2	Ppip5k1	Exon3	N	121152588	121152650	1	37.4	53.7	34.1	49.9
chr11	Itgb4	Exon29	N	115861857	115861994	1	72.7	93.7	72.2	94.4
chr9	Sorl1	Exon26	N	41832379	41832552	1	51.5	73.0	42.1	63.0
chr16	Dopey2	Exon26	N	93793332	93793476	1	50.9	70.7	44.1	62.8
chr10	Baz2a	Exon26	N	127562555	127562701	1	75.0	95.5	72.7	88.0
chr2	Duox2	Exon24	N	122120821	122120917	1	22.7	51.8	17.2	48.1
chr4	Map3k6	Exon24	N	132807526	132807688	1	27.5	70.0	35.5	50.8
chr7	Myh14	Exon23	N	51888305	51888374	1	43.1	79.7	45.7	79.7
chr2	Duox2	Exon23	N	122120325	122120415	1	40.5	80.0	44.2	75.4
chr2	Fbn1	Exon23	N	125166992	125167063	1	47.2	82.4	50.0	76.5
chr5	Ube3b	Exon23	N	114862860	114862918	1	49.4	69.1	48.4	67.0
chr12	Wdr35	Exon22	N	9028536	9028646	1	54.3	84.1	44.8	74.6
chr5	Myo1h	Exon22	N	114807928	114808087	1	28.2	66.9	42.6	68.6
chr5	Ube3b	Exon22	N	114862431	114862496	1	56.7	76.9	62.0	81.6
chr2	Atp9a	Exon22	N	168514799	168514850	1	57.1	76.3	62.9	79.5
chr19	Tjp2	Exon20	N	24209340	24209464	1	22.7	67.0	33.9	61.5
chr1	Als2	Exon20	N	59244579	59244710	1	64.5	80.8	63.9	84.6
chr14	Lect1	Exon2	N	80045170	80045323	1	39.6	60.9	18.4	65.6
chr15	Kcnh3	Exon2	N	99057412	99057546	1	53.7	83.3	35.1	80.0
chr2	Btbd3	Exon2	N	138105485	138105575	1	24.3	59.3	20.5	60.5
chr17	Mas1	Exon2	N	13040943	13040986	1	36.3	54.9	28.9	68.9
chr2	Spef1	Exon2	N	130997940	130997994	1	57.1	85.0	59.5	96.4
chr13	H2afy	Exon2	N	56185557	56185646	1	64.0	83.5	48.0	80.0
chr17	Lta	Exon2	N	35341503	35341607	2	16.7	41.2	15.8	47.2
chr1	Ppp1r12b	Exon2	N	136670003	136670056	1	13.9	36.0	12.5	43.4
chr3	Cyr61	Exon2	N	145311497	145311841	2	40.8	70.0	40.4	71.1
chr5	P2rx7	Exon2	N	123104170	123104238	1	0.0	50.0	19.2	49.2
chr2	Slc20a1	Exon2	N	129025919	129026059	1	64.8	86.9	59.8	87.1
chr19	Sorbs1	Exon2	N	40373953	40374011	1	80.6	100.0	74.4	100.0
chr5	Wbscr25	Exon2	N	135468443	135468861	2	22.1	51.9	14.9	38.8
chr2	Trp53tg5	Exon2	N	164298428	164298558	1	60.0	90.6	51.1	74.2
chr6	Fabp1	Exon2	N	71153081	71153173	1	55.7	77.4	60.7	83.3
chr6	Atp6v0e2	Exon2	N	48490039	48490165	2	39.4	80.1	65.1	87.7
chr3	Sh2d2a	Exon2	N	87652280	87652461	1	50.0	78.8	68.1	90.5
chr15	Nfam1	Exon2	N	82840805	82840903	1	52.3	68.6	50.6	72.9
chr4	Sepn1	Exon2	N	134096738	134096850	1	46.7	79.3	44.8	66.9
chr14	Fbxl3	Exon2	N	103491521	103491643	1	20.9	61.2	23.0	44.9
chr19	Rab11fip2	Exon2	N	60011509	60011977	2	56.1	71.2	62.1	84.0
chr19	1700019N19Rik	Exon2	N	58862025	58862211	1	59.2	78.0	64.2	86.1
chr7	Dusp8	Exon2	N	149269497	149269656	1	14.7	41.0	15.7	37.1
chr9	Ulk3	Exon2	N	57438468	57438588	1	59.3	80.0	54.4	75.8
chr8	Tmem66	Exon2	N	35228195	35228594	2	67.7	92.4	59.9	80.6
chr16	Tagln3	Exon2	N	45723036	45723210	1	63.4	90.2	66.7	86.8
chr3	Ankrd50	Exon2	N	38381613	38382906	7	53.5	75.4	60.3	80.4
chr8	Rad23a	Exon2	N	87361359	87361489	1	55.1	73.0	51.7	71.7
chr11	Trim65	Exon2	N	115988752	115988929	1	19.2	34.7	19.7	39.2
chr11	Bzrap1	Exon2	N	87576283	87576411	1	57.6	90.1	66.9	86.4
chr7	Chst15	Exon2	N	139439547	139439703	1	60.0	90.0	71.1	90.4

chr3	Them5	Exon2	N	94148331	94148469	1	78.4	94.8	74.2	92.6
chr12	Dnah11	Exon2	N	119118925	119119037	1	40.9	65.6	55.1	72.6
chr3	Dpyd	Exon2	N	118377407	118377489	1	73.8	90.3	68.2	85.7
chr8	Slc10a7	Exon2	N	81049061	81049197	1	65.4	82.5	65.5	82.7
chr6	Met	Exon2	N	17441227	17442437	7	52.4	68.8	45.6	62.7
chr12	Cfl2	Exon2	N	55962513	55962820	2	54.5	75.0	63.0	80.0
chr7	Mrpl23	Exon2	N	149721975	149722057	1	17.7	40.2	10.4	26.8
chr18	Ppargc1b	Exon2	N	61462273	61462394	1	52.7	84.8	59.6	75.2
chr6	Suclg1	Exon2	N	73210469	73210585	1	52.7	78.5	61.4	76.7
chr8	B930025P03Rik	Exon2	N	10876197	10876279	1	83.0	100.0	77.6	92.9
chr10	Rassf3	Exon2	N	120853122	120853344	1	53.1	81.4	57.3	72.6
chr11	Selm	Exon2	N	3416489	3416523	1	32.0	47.1	33.3	48.5
chr7	1600016N20Rik	Exon2	N	148396590	148396786	2	60.7	75.9	55.4	70.5
chr7	Abcc8	Exon19	N	53378228	53378312	1	16.7	33.3	15.1	38.1
chr11	Tanc2	Exon19	N	105771517	105771640	1	50.9	72.7	30.8	52.1
chr17	Dennd1c	Exon19	N	57214792	57214911	1	66.0	84.7	67.0	84.8
chr3	Pld1	Exon18	N	27987568	27987681	1	47.4	78.3	42.4	79.6
chr8	Arhgef10	Exon18	N	14974976	14975091	1	50.0	67.7	45.6	71.5
chr19	Myrf	Exon17	N	10294285	10294361	1	51.3	69.8	47.9	67.6
chr2	Plcb2	Exon17	N	118542948	118543063	1	52.4	73.5	52.8	68.4
chr4	Hectd3	Exon16	N	116675115	116675244	1	25.0	41.4	26.2	50.0
chr17	C2	Exon16	N	35018517	35018741	1	18.8	37.7	15.6	32.1
chr8	Jak3	Exon15	N	74208116	74208267	1	41.9	69.9	44.9	73.6
chr7	Gpatch1	Exon15	N	36092208	36092305	1	54.4	75.5	47.8	76.2
chr8	Nlrc5	Exon15	N	97014230	97014322	1	31.8	54.2	53.2	80.0
chr19	Sorbs1	Exon15	N	40444180	40444209	1	54.8	84.4	60.0	83.3
chr13	Mccc2	Exon15	N	100770205	100770271	1	40.0	76.9	55.6	76.5
chr5	Ccdc146	Exon15	N	20836358	20836567	1	62.3	78.1	57.6	74.3
chr5	Fryl	Exon14	N	73448963	73449103	1	39.6	69.9	48.3	65.2
chr3	Tnni3k	Exon14	N	154619904	154620053	1	55.0	82.1	80.0	95.2
chr5	Gpr133	Exon13	N	129649842	129649983	1	46.4	67.1	40.5	70.9
chr16	Itsn1	Exon13	N	91820924	91821064	1	62.5	85.0	65.1	90.8
chr14	Rabggta	Exon13	N	56339973	56340097	1	39.2	88.2	52.0	77.5
chr14	Dock9	Exon13	N	121982884	121983021	1	51.1	74.5	40.4	64.1
chr17	Tiam2	Exon13	N	3454088	3454351	1	44.3	62.4	42.0	65.4
chr8	Palld	Exon13	N	64181899	64182040	1	66.9	85.1	59.9	82.0
chr5	Card11	Exon13	N	141366940	141367022	1	33.7	69.3	40.4	61.3
chr2	Dnm1	Exon13	N	32191458	32191593	1	28.5	52.3	31.3	46.8
chr2	Stxbp1	Exon12	N	32667541	32667625	1	40.0	73.1	42.5	67.8
chr8	Gse1	Exon12	N	123098834	123099205	2	32.9	53.0	32.0	53.2
chr9	Cpne4	Exon12	N	104922096	104922147	1	70.4	86.7	80.0	96.6
chr10	Tjp3	Exon11	N	80742250	80742316	1	52.9	69.7	47.9	86.8
chr2	Stxbp1	Exon11	N	32666625	32666755	1	59.8	84.9	59.6	87.1
chr6	St7	Exon11	N	17856408	17856510	1	65.6	83.8	59.0	84.6
chr14	Lrch1	Exon11	N	75211407	75211522	1	46.6	76.5	50.4	76.0
chr4	Plk3	Exon11	N	116805510	116805639	1	66.7	88.4	61.4	84.5
chr8	Slc9a5	Exon11	N	107883227	107883353	1	14.1	52.1	20.6	42.0
chr11	Slc38a10	Exon11	N	119999171	119999295	1	33.0	61.1	35.6	56.7
chr8	Tacc1	Exon11	N	26310563	26310678	1	56.5	74.5	60.9	81.6

chr11	Zmynd15	Exon11	N	70278609	70278667	1	41.8	82.2	54.5	74.4
chr6	Met	Exon11	N	17490438	17490656	1	58.9	77.5	61.7	81.0
chr2	F2	Exon11	N	91475328	91475352	1	76.9	92.3	78.0	96.2
chr4	Kif2c	Exon11	N	116842690	116842744	1	2.8	40.0	9.0	26.6
chr13	Itga2	Exon10	N	115646339	115646480	1	41.1	66.7	22.2	70.4
chr11	Kcnab3	Exon10	N	69144764	69144858	1	44.6	73.6	38.6	70.2
chr17	Cpne5	Exon10	N	29313107	29313148	1	27.5	69.1	33.3	64.9
chr2	Cry2	Exon10	N	92267059	92267167	1	64.2	83.0	54.5	85.0
chr4	Whrn	Exon10	N	63122316	63122441	1	36.3	54.3	42.6	69.4
chr10	Myo1a	Exon10	N	127148140	127148226	1	55.7	75.9	53.9	75.8
chr4	Rap1gap	Exon10	N	137273609	137273709	1	37.3	59.1	31.2	52.9
chr15	Enpp2	Exon10	N	54697467	54697594	1	68.5	86.4	61.3	82.3
chr7	Dhx32	Exon10	N	140951083	140951596	4	62.2	94.0	54.8	75.6
chr11	Evpl	Exon10	N	116091283	116091437	1	47.1	67.4	44.8	65.1
chr15	Nrbp2	Exon10	N	75920178	75920261	2	61.3	84.8	65.4	84.6
chr9	Vipr1	Exon10	N	121577552	121577681	1	3.8	44.8	20.5	39.6
chr11	Wnk4	Exon10	N	101130884	101131000	1	23.9	48.2	25.6	43.8
chr11	Srebf1	Exon10	N	60017301	60017479	1	31.7	56.6	35.8	52.4
chr4	Plk3	Exon10	N	116805332	116805419	1	38.2	62.2	42.1	57.5
chr4	Olfr1339	Downstrear	N	118408084	118409083	5	61.0	93.2	25.5	90.5
chr17	Plin3	Downstrear	N	56417385	56418384	5	58.9	78.8	22.2	78.8
chr12	Gsc	Downstrear	N	105708419	105709418	4	24.0	71.1	20.3	70.0
chr1	Arid5a	Downstrear	N	36380875	36381874	5	56.2	82.3	41.4	85.3
chr11	Olfr1373	Downstrear	N	51957095	51958094	5	6.7	46.2	48.7	92.5
chr1	1700056E22Rik	Downstrear	N	185855911	185856910	5	54.1	95.1	51.7	94.8
chr13	Gm5083	Downstrear	N	44220549	44221548	5	13.6	100.0	53.2	95.2
chr7	Ovch2	Downstrear	N	114924058	114925057	5	3.6	41.5	30.8	72.4
chr2	Zcchc3	Downstrear	N	152236692	152237691	5	38.3	71.4	30.3	71.4
chr14	Pebp4	Downstrear	N	70459694	70460693	5	70.5	86.4	44.6	83.9
chr14	4931406H21Rik	Downstrear	N	26410148	26411147	5	26.0	61.6	26.6	65.3
chr18	Pcdhga1	Downstrear	N	38001525	38002524	5	20.9	52.4	23.9	62.3
chr18	Pcdhga3	Downstrear	N	38001527	38002526	5	20.9	52.4	23.9	62.3
chr18	Pcdhga8	Downstrear	N	38001527	38002526	5	20.9	52.4	23.9	62.3
chr18	Pcdhga9	Downstrear	N	38001518	38002517	5	20.9	52.4	23.9	62.3
chr18	Pcdhgb4	Downstrear	N	38001525	38002524	5	20.9	52.4	23.9	62.3
chr13	Calm4	Downstrear	N	3837918	3838917	5	29.4	100.0	43.3	81.5
chr7	Pou2f2	Downstrear	N	25875134	25876133	5	43.3	67.4	10.1	47.9
chr11	Ehbp1	Downstrear	N	21904829	21905828	5	65.0	84.6	52.4	90.1
chr14	Clybl	Downstrear	N	122801457	122802456	5	42.5	64.9	15.7	52.9
chr7	Spred3	Downstrear	N	29942848	29943847	5	10.2	59.6	22.7	59.1
chr8	Dda1	Downstrear	N	74000335	74001334	1	36.8	60.0	28.6	63.3
chr7	Syt8	Downstrear	N	149626304	149627303	5	25.0	60.7	43.0	76.8
chr16	Gm933	Downstrear	N	32803974	32804973	5	67.6	92.4	61.3	94.9
chr2	4921531C22Rik	Downstrear	N	179713719	179714718	5	18.8	55.7	18.3	51.9
chr19	Hectd2	Downstrear	N	36695626	36696625	5	51.4	86.6	61.5	94.0
chr3	Foxo1	Downstrear	N	52154032	52155031	5	51.3	76.9	48.9	79.9
chr15	Copz1	Downstrear	N	103130296	103131295	3	41.2	70.9	34.5	65.3
chr7	Rras2	Downstrear	N	121189296	121190295	5	4.6	24.9	39.4	69.8
chr19	Lgals12	Downstrear	N	7670151	7671150	5	40.3	80.0	45.8	75.9

chr19	Rcor2	Downstrear	N	7349716	7350715	5	47.9	68.1	38.3	68.1
chr9	Tcta	Downstrear	N	108204289	108205288	6	4.9	43.4	49.6	79.5
chr9	Lrrfip2	Downstrear	N	111128173	111129172	5	74.5	98.7	68.4	97.9
chr19	Stx5a	Downstrear	N	8830133	8831132	4	21.1	51.1	20.8	50.1
chr14	Ccdc25	Downstrear	N	66485442	66486441	5	50.4	70.1	52.2	81.1
chr15	Kifc2	Downstrear	N	76498627	76499626	4	44.4	74.5	47.6	75.5
chr15	Mir1943	Downstrear	N	79204658	79205657	5	59.3	78.7	53.3	80.8
chr8	1810019D21Rik	Downstrear	N	108662623	108663622	5	60.4	85.4	63.0	90.5
chr4	Fam151a	Downstrear	N	106420898	106421897	5	57.2	89.4	65.3	91.9
chr9	Ift46	Downstrear	N	44600798	44601797	5	65.6	81.2	50.7	76.4
chr9	Tmem25	Downstrear	N	44600862	44601861	5	65.6	81.2	50.7	76.4
chr4	Asap3	Downstrear	N	135802489	135803488	5	9.4	65.6	22.1	47.1
chr4	Polr1e	Downstrear	N	45046565	45047564	5	63.6	93.8	70.6	95.4
chr4	Fndc5	Downstrear	N	128821838	128822837	5	39.6	78.1	43.9	68.5
chr4	Wasf2	Downstrear	N	132754246	132755245	5	4.8	67.8	37.8	62.0
chr14	1700108J01Rik	Downstrear	N	122628127	122629126	5	24.9	75.4	51.1	74.7
chr13	Mcur1	Downstrear	N	43632775	43633774	5	57.3	80.7	53.9	77.3
chr16	Synj1	Downstrear	N	90935342	90936341	3	34.1	52.7	34.5	57.9
chr2	Atp9a	Downstrear	N	168458938	168459937	5	36.5	67.7	42.8	66.0
chr10	Smarcc2	Downstrear	N	127927231	127928230	6	39.6	59.4	41.4	64.5
chr3	Adad1	Downstrear	N	37010435	37011434	5	66.7	82.9	61.9	84.8
chr1	Pou2f1	Downstrear	N	167794285	167795284	5	25.8	68.6	59.4	82.2
chr7	Rgs9bp	Downstrear	N	36363013	36364012	5	24.0	82.3	68.4	90.8
chr2	Zfp663	Downstrear	N	165175797	165176796	5	51.6	75.2	57.8	80.2
chr2	Bmi1	Downstrear	N	18608257	18609256	5	58.5	88.7	69.5	91.9
chr4	Camk2n1	Downstrear	N	138016042	138017041	5	55.5	70.8	53.4	75.7
chr1	Pecr	Downstrear	N	72304747	72305746	5	66.7	90.2	25.4	47.6
chr2	Myo3a	Downstrear	N	22473773	22474772	5	22.8	70.7	21.6	43.6
chr15	Dpys	Downstrear	N	39599031	39600030	5	47.7	81.8	58.5	80.6
chr7	Sae1	Downstrear	N	16911403	16912402	5	22.5	45.2	35.6	57.6
chr3	Lrrc40	Downstrear	N	157731443	157732442	5	21.7	51.0	28.0	50.0
chr5	Oas1d	Downstrear	N	121371657	121372656	5	22.7	38.5	16.7	38.5
chr3	Ints3	Downstrear	N	90194302	90195301	4	42.7	57.9	41.4	63.1
chr4	Tnfsf8	Downstrear	N	63492858	63493857	5	50.0	89.2	63.3	84.8
chr8	Snord71	Downstrear	N	112363276	112364275	5	64.1	94.2	67.8	89.1
chr11	Dcakd	Downstrear	N	102854370	102855369	5	44.6	90.0	51.8	73.0
chr11	2310047M10Rik	Downstrear	N	68875079	68876078	5	27.3	48.5	21.2	42.1
chr18	8430422H06Rik	Downstrear	N	14437052	14438051	5	60.0	86.2	79.3	100.0
chr11	Gm11563	Downstrear	N	99518256	99519255	5	46.8	64.6	68.5	89.1
chr7	Lgi4	Downstrear	N	31855955	31856954	5	58.8	90.2	47.1	67.6
chr15	Foxh1	Downstrear	N	76497654	76498653	5	50.3	77.6	51.5	71.8
chr6	Tuba3a	Downstrear	N	125227292	125228291	5	58.3	92.5	75.8	95.9
chr7	Mir675	Downstrear	N	149761969	149762968	5	52.2	73.6	48.6	68.7
chr17	Hsf2bp	Downstrear	N	32080714	32081713	5	63.7	85.7	56.0	76.0
chr4	E330017L17Rik	Downstrear	N	129586241	129587240	5	29.9	58.6	34.4	54.4
chr5	D5Ertd605e	Downstrear	N	148234621	148235620	5	4.5	51.2	24.8	44.8
chr10	Arid3a	Downstrear	N	79417758	79418757	5	58.2	86.2	63.4	83.2
chr9	Tln2	Downstrear	N	67063892	67064891	5	51.1	80.6	50.1	70.0
chr10	Taar8c	Downstrear	N	23819649	23820648	5	58.9	74.8	74.4	94.2

chr4	1700013G24Rik	Downstrear	N	137011377	137012376	5	65.6	82.3	61.1	80.8
chr3	4930442L01Rik	Downstrear	N	96632749	96633748	5	61.8	79.8	55.9	75.6
chr8	Rbl2	Downstrear	N	93647744	93648743	5	62.4	78.3	60.2	79.8
chr15	Ank	Downstrear	N	27524663	27525662	5	38.0	65.1	51.9	71.6
chr19	Ccdc86	Downstrear	N	11014971	11015970	1	56.5	73.5	44.4	63.6
chr5	4933426D04Rik	Downstrear	N	24412668	24413667	4	2.7	55.3	76.3	95.5
chr17	Ergic1	Downstrear	N	26793879	26794878	5	71.0	90.8	68.4	87.5
chr7	Fut2	Downstrear	N	52902961	52903960	5	63.6	82.2	68.2	87.0
chr13	Hist1h3d	Downstrear	N	23668082	23669081	5	31.6	55.3	11.4	30.2
chr7	Zfp580	Downstrear	N	5005326	5006325	3	49.0	72.1	28.4	46.9
chr4	Triqk	Downstrear	N	12908633	12909632	3	80.5	97.8	68.6	87.0
chr2	Gm5535	Downstrear	N	143997886	143998885	5	21.4	36.5	21.2	39.4
chr6	2700086A05Rik	Downstrear	N	52163597	52164596	6	16.9	56.8	11.6	29.7
chr6	Mira	Downstrear	N	52163490	52164489	6	16.9	56.8	11.6	29.7
chr9	Ccr8	Downstrear	N	120004025	120005024	5	45.2	61.3	51.3	69.2
chr19	9130011E15Rik	Downstrear	N	45891634	45892633	5	55.3	75.9	62.6	80.4
chr7	Mrgpra1	Downstrear	N	54589245	54590244	4	59.5	78.3	29.4	47.2
chr15	Ddx17	Downstrear	N	79357126	79358125	5	50.0	75.0	56.0	73.8
chr17	Gpr116	Downstrear	N	43596507	43597506	5	56.1	86.2	67.5	85.2
chr13	1110046J04Rik	Downstrear	N	34052051	34053050	4	60.0	85.7	73.0	90.5
chr12	Mir1188	Downstrear	N	110850152	110851151	6	53.4	70.3	48.6	66.1
chr18	Camk2a	Downstrear	N	61147807	61148806	5	14.3	90.3	58.1	75.5
chr13	Hist1h2an	Downstrear	N	21877695	21878694	5	3.0	18.9	6.7	24.0
chr5	Fam185a	Downstrear	N	20987943	20988942	5	38.7	61.2	48.9	66.1
chr2	Foxa2	Downstrear	N	147867614	147868613	5	23.6	80.5	29.5	46.5
chr7	Rpl13a	Downstrear	N	52379933	52380932	3	8.0	28.7	17.1	34.0
chr2	Itpka	Downstrear	N	119576990	119577989	6	68.8	86.2	71.0	87.8
chr17	Slc25a41	Downstrear	N	57171195	57172194	2	50.9	66.9	51.2	67.8
chr10	4930404N11Rik	Downstrear	N	80825769	80826768	4	35.7	55.0	34.2	50.8
chr6	Mlf2	Downstrear	N	124886168	124887167	5	81.7	97.0	81.0	97.6
chr8	Dusp26	Downstrear	N	32207520	32208519	5	42.0	77.7	39.7	56.1
chr9	Acvr2b	Downstrear	N	119342625	119343624	5	47.0	72.2	46.6	62.9
chr6	Mir680-1	Downstrear	N	129641662	129642661	5	71.4	87.2	59.4	75.7
chr5	Otof	Downstrear	N	30668351	30669350	5	41.2	61.1	39.0	55.1
chr2	Mir219-2	Downstrear	N	29700151	29701150	3	60.7	84.1	62.7	78.9
chr2	Urm1	Downstrear	N	29700517	29701516	2	60.7	84.1	62.7	78.9
chr1	Esrrg	Downstrear	N	190038764	190039763	5	60.8	78.3	62.3	78.5
chr3	Fbxw7	Downstrear	N	84783121	84784120	5	73.5	100.0	67.2	83.2
chr7	Chst8	Downstrear	N	35458487	35459486	5	44.7	76.0	49.3	65.2
chr16	Hira	Downstrear	N	18970402	18971401	5	12.7	43.7	18.7	34.6
chr10	Ndufa4l2	Downstrear	N	126954211	126955210	5	40.2	81.1	59.8	75.5
chr10	A130077B15Rik	Downstrear	N	122001079	122002078	5	70.9	89.2	71.1	86.7
chr7	Xrra1	Downstrear	N	107066335	107067334	4	68.7	84.1	69.9	85.5
chr2	Lhx3	Downstrear	N	26054732	26055731	5	21.2	42.5	27.6	43.2
chr4	Zdhhc18	Downstrear	N	133161907	133162906	5	58.8	87.5	76.4	91.8
chr15	Peg13	Downstrear	N	72635030	72636029	5	54.7	80.6	64.4	79.6
chr2	Zcchc3	3-UTR	N	152237692	152239310	8	13.2	57.2	15.4	60.0
chr1	Dtl	3-UTR	N	193361244	193363121	9	29.0	45.0	39.5	83.2
chr2	Myo3a	3-UTR	N	22473690	22473772	1	17.1	46.8	13.2	56.3

chr2	Pdhx	3-UTR	N	102861213	102862167	5	54.4	79.7	39.3	79.5
chr19	Rab3il1	3-UTR	N	10109030	10110076	5	9.1	62.9	55.9	94.7
chr14	Slitrk5	3-UTR	N	112081042	112082357	7	30.5	51.0	2.5	40.2
chr16	5-Sep	3-UTR	N	18621904	18622832	3	66.3	85.5	49.7	87.3
chr11	Cd7	3-UTR	N	120898063	120898356	1	49.0	76.7	36.1	72.9
chr14	Amer2	3-UTR	N	60999103	60999840	4	38.2	74.7	44.8	80.4
chr3	Mab21l2	3-UTR	N	86349503	86350533	4	43.0	79.3	45.7	79.2
chr11	Vat1	3-UTR	N	101320062	101321506	7	33.5	57.2	33.2	65.4
chr9	Lars2	3-UTR	N	123370797	123371782	3	20.3	51.8	25.6	57.6
chr5	Drc1	3-UTR	N	30668834	30668993	2	20.0	48.2	22.9	54.6
chr4	Cdkn2b	3-UTR	N	88952198	88952962	3	49.2	92.5	54.1	85.6
chr7	Fzd4	3-UTR	N	96556871	96558620	9	60.6	92.0	45.7	76.2
chr11	Dusp14	3-UTR	N	83861547	83862118	3	20.0	81.3	29.2	59.4
chr1	Fzd5	3-UTR	N	64777132	64781416	21	37.4	58.1	28.9	58.5
chr2	Sp5	3-UTR	N	70315227	70315783	1	24.0	69.7	19.5	48.9
chr11	Arl5c	3-UTR	N	97850894	97851604	4	20.8	41.8	20.8	50.0
chr3	Slc50a1	3-UTR	N	89072168	89072418	1	63.8	86.7	42.9	72.0
chr5	Card11	3-UTR	N	141348953	141349278	1	22.9	59.8	30.1	59.1
chr18	Dsc3	3-UTR	N	20119431	20121872	12	57.6	91.7	62.5	90.7
chr19	Mark2	3-UTR	N	7349886	7351579	8	63.1	78.3	55.6	83.6
chr4	Kif12	3-UTR	N	62826671	62826865	1	40.0	55.4	44.3	71.8
chr3	Dkk2	3-UTR	N	131841085	131843268	11	72.3	100.0	71.3	98.5
chr2	Hoxd8	3-UTR	N	74544870	74545364	2	41.9	72.9	30.8	57.9
chr11	Wbp2	3-UTR	N	115939887	115940856	5	62.2	86.9	58.6	84.1
chr18	Wnt8a	3-UTR	N	34707303	34707715	2	57.8	90.0	57.6	82.9
chr14	Gm16617	3-UTR	N	52606064	52606514	2	44.1	91.9	69.2	94.0
chr3	Actrt3	3-UTR	N	30495995	30496755	4	50.6	71.7	37.4	62.1
chr11	Cygb	3-UTR	N	116506909	116508272	7	43.4	68.6	58.8	83.4
chr10	Gpr182	3-UTR	N	127186658	127186939	1	29.2	57.3	29.0	52.8
chr1	2010300C02Rik	3-UTR	N	37668521	37669101	3	13.4	52.1	30.4	53.7
chr17	Rnf151	3-UTR	N	24852785	24853180	2	45.9	71.9	43.8	66.7
chr11	Zfp735	3-UTR	N	73525939	73527310	5	48.7	66.7	34.6	57.1
chr9	Dnm2	3-UTR	N	21311907	21312203	2	21.6	43.2	24.5	47.0
chr9	Mmp13	3-UTR	N	7282119	7283333	6	75.6	91.9	73.9	96.2
chr11	Rhbdd3	3-UTR	N	5005981	5006094	2	66.7	92.3	73.7	95.9
chr8	Hpgd	3-UTR	N	58799003	58799843	4	65.5	84.6	58.3	80.5
chr17	Cd2ap	3-UTR	N	42929900	42933276	15	65.0	88.0	64.9	87.0
chr11	Sowaha	3-UTR	N	53290080	53291762	8	40.0	88.8	63.4	85.0
chr14	Dhrs4	3-UTR	N	56108771	56109177	2	61.9	80.1	61.2	82.6
chr10	Shmt2	3-UTR	N	126954179	126954844	5	40.2	76.6	50.3	70.8
chr2	Foxa2	3-UTR	N	147868614	147869249	3	30.4	84.3	29.7	50.0
chr7	Tmc7	3-UTR	N	125679358	125681650	11	41.2	62.5	70.3	90.6
chr7	1600016N20Rik	3-UTR	N	148395942	148395971	2	71.5	87.4	71.3	91.3
chr7	Msx3	3-UTR	N	147232056	147233638	7	58.8	75.5	50.3	70.0
chr11	Kdm6b	3-UTR	N	69212020	69213327	5	33.3	60.3	42.5	62.2
chr5	Tbx3	3-UTR	N	120133102	120134610	8	56.3	75.4	35.6	55.2
chr11	Peli1	3-UTR	N	21048527	21050330	7	45.7	64.4	40.3	59.8
chr19	Arl3	3-UTR	N	46605599	46605828	1	54.2	79.3	59.2	78.7
chr15	Kifc2	3-UTR	N	76497889	76498626	4	55.1	77.6	57.0	76.2

chr19	Mpeg1	3-UTR	N	12537833	12539775	10	9.2	30.8	28.2	47.2
chr15	Mkl1	3-UTR	N	80842711	80843714	6	71.2	92.3	75.4	94.4
chr7	Rpl13a	3-UTR	N	52380933	52381315	3	25.4	44.8	22.3	40.9
chr1	Arl4c	3-UTR	N	90594801	90597660	12	26.8	47.3	28.3	46.6
chr4	Cga	3-UTR	N	34854393	34854623	1	62.9	88.3	66.0	84.3
chr13	Foxq1	3-UTR	N	31651989	31652843	3	24.4	42.3	22.0	40.0
chr2	BC052040	3-UTR	N	115602768	115604504	9	45.6	96.3	53.1	70.9
chr9	Chrna3	3-UTR	N	54859150	54860607	7	61.5	86.7	65.3	83.1
chr3	Pias3	3-UTR	N	96509039	96509993	5	58.1	78.3	59.9	77.4
chr13	Tnpo1	3-UTR	N	99612036	99616410	22	76.2	92.7	74.2	91.7
chr15	Cyp2d13	3-UTR	N	82467180	82467428	1	46.4	62.4	45.4	62.9
chr19	Klc2	3-UTR	N	5107746	5108604	4	13.5	31.7	41.0	58.1
chr17	Pkmyt1	3-UTR	N	23873574	23873696	1	68.1	85.9	68.2	84.9
chr1	Fcer1g	3-UTR	N	173159703	173159995	1	44.0	60.5	45.2	61.6
chr18	Spire1	3-UTR	N	67647863	67650944	15	60.4	77.4	69.5	85.6
chr9	Fut4	3-UTR	N	14552903	14555138	11	64.8	81.0	61.8	77.9
chr9	1600029D21Rik	3-UTR	N	50312651	50313744	7	39.0	55.9	39.3	55.4
chr11	Ace	3-UTR	N	105850349	105851278	4	36.7	58.1	36.4	52.4
chr8	Mcm5	3-UTR	N	77651224	77652338	6	67.4	95.3	69.4	85.1
chr10	Ado	3-UTR	N	67007259	67010750	16	58.1	80.5	59.7	75.2
chr6	Creb3l2	3-UTR	N	37281021	37282424	7	76.9	92.0	69.0	84.5
chr11	Bcas3	3-UTR	N	85638853	85639560	4	65.0	90.3	64.6	80.1
chr8	Ccl17	3-UTR	N	97335754	97335936	1	64.5	80.6	67.0	82.4
chr7	Arnt2	3-UTR	N	91394785	91398565	19	43.3	76.9	48.9	64.2
chr5	Fzd1	3-UTR	N	4753839	4755651	9	29.1	58.3	74.6	89.9
chr4	Pgd	3-UTR	N	148524094	148524752	3	45.0	73.1	59.1	74.3
chr17	2300002M23Rik	3-UTR	N	35705762	35705890	1	13.2	36.9	18.0	33.1
chr14	Dok2	3-UTR	N	71177881	71178301	2	41.4	64.6	32.2	47.3
chr15	Fzd6	3-UTR	N	38868101	38869736	8	60.8	96.9	66.1	81.1

Group 7-mDMR genes associated with methylation loss at 5' non-CGI

							DNA Methylation Level (%)			
							ISCs		Diff. Cells	
**Chr**	**Gene**	**Region**	**CGI**	**Start**	**End**	**#Bins**	**P0**	**P21**	**P0**	**P21**
chr4	AI427809	5-UTR	N	53282951	53283104	1	60.8	10.5	85.1	0.0
chr5	Trafd1	5-UTR	N	121834096	121835624	4	78.7	38.9	73.0	2.5
chr5	Pf4	5-UTR	N	91201461	91201607	1	62.1	12.6	79.0	8.6
chr10	Vnn3	5-UTR	N	23571268	23571389	1	80.2	0.0	67.7	0.6
chr17	Xdh	5-UTR	N	74299469	74299522	1	73.1	0.6	50.5	0.0
chr9	Casp1	5-UTR	N	5298517	5298716	1	56.2	3.1	48.2	0.8
chr7	Tmem150b	5-UTR	N	4676578	4676853	2	40.3	3.1	48.5	1.7
chr2	Znf512b	5-UTR	N	181325689	181327166	5	95.0	6.5	46.5	0.9
chr5	Azgp1	5-UTR	N	138422749	138422787	1	38.1	0.0	48.7	4.7
chr14	Mtrf1	5-UTR	N	79797579	79801236	17	63.4	44.8	89.6	46.0
chr6	Tmem106b	5-UTR	N	13019759	13021743	7	96.0	47.9	58.3	14.8
chr1	Aox3	5-UTR	N	58169980	58170102	1	54.2	31.1	48.9	7.3
chr7	Ptprh	5-UTR	N	4554701	4555643	5	67.4	45.8	73.1	32.3
chr8	Irf8	5-UTR	N	123260276	123263717	15	52.5	21.6	55.5	15.1
chr13	F13a1	5-UTR	N	37139356	37142113	15	75.1	56.3	91.2	51.2

chr17	C030013G03Rik	5-UTR	N	12659710	12659888	1	65.2	20.8	53.1	13.6
chr18	4930592I03Rik	5-UTR	N	83089911	83089952	1	82.3	48.4	80.2	40.8
chr1	Pigr	5-UTR	N	132723261	132731005	39	57.3	19.0	60.4	21.0
chr16	Gm10791	5-UTR	N	84972456	84972997	3	65.1	30.3	80.5	41.5
chr10	Rdh9	5-UTR	N	127213461	127213540	1	81.5	14.7	76.3	40.0
chr14	Phf11d	5-UTR	N	59984247	59984327	1	72.7	50.5	89.2	52.9
chr1	Kcnj13	5-UTR	N	89285965	89291304	27	74.4	26.0	77.5	41.4
chr9	Nr2e3	5-UTR	N	59797692	59797886	1	89.4	40.6	78.8	43.1
chr8	Ces2g	5-UTR	N	107485618	107485687	1	35.6	0.0	36.2	0.7
chr17	Slc22a1	5-UTR	N	12868526	12868704	1	23.4	0.9	35.5	0.5
chr4	Cyp4b1	5-UTR	N	115320293	115320310	1	33.3	3.8	34.8	0.0
chr16	Mir802	5-UTR	N	93369965	93370061	1	57.1	21.4	68.5	34.2
chr1	Aqp12	5-UTR	N	94902911	94902979	1	30.4	10.0	44.9	11.4
chr6	Tigd2	5-UTR	N	59158864	59160143	3	48.3	22.8	50.9	17.4
chr19	5830416P10Rik	5-UTR	N	53539176	53539286	1	80.5	36.0	77.8	44.3
chr9	Mir3109	5-UTR	N	69304751	69304838	1	44.2	14.3	50.8	18.3
chr15	Vdr	5-UTR	N	97715371	97738727	113	75.6	43.2	73.4	41.2
chr19	Tmem216	5-UTR	N	10629065	10630728	5	32.7	1.1	30.7	0.0
chr4	Pdik1l	5-UTR	N	133840446	133843761	9	41.9	23.4	55.1	24.5
chr9	Spa17	5-UTR	N	37419575	37421305	6	91.2	48.4	87.4	58.2
chr9	Ubl7	5-UTR	N	57758793	57760473	7	81.4	64.6	68.2	39.3
chr7	Gm10619	5-UTR	N	80960823	80961389	3	82.7	61.1	85.5	57.6
chr1	Abca12	5-UTR	N	71461157	71461484	2	52.9	21.8	85.9	58.1
chr17	2010106C02Rik	5-UTR	N	86686241	86686518	1	29.8	1.7	29.3	1.6
chr11	Cant1	5-UTR	N	118272804	118280432	35	61.9	22.5	54.6	27.1
chr1	Slc9a4	5-UTR	N	40637072	40637360	1	17.0	1.0	30.3	3.0
chr12	Zfyve1	5-UTR	N	84935831	84938097	9	60.0	43.8	66.6	39.7
chr2	Myh7b	5-UTR	N	155436979	155437393	2	82.5	56.6	80.5	53.7
chr8	Tnfsf13b	5-UTR	N	10006633	10006842	1	47.2	10.0	53.7	27.1
chr11	Rnft1	5-UTR	N	86298159	86299613	4	48.8	28.5	34.4	8.3
chr14	Acox2	5-UTR	N	9089376	9091533	11	38.4	11.7	42.6	16.7
chr10	Adat3	5-UTR	N	80065625	80069074	14	49.8	15.1	57.3	31.7
chr2	Defb29	5-UTR	N	152365747	152365777	1	63.6	44.4	69.3	43.9
chr8	Trappc11	5-UTR	N	48616210	48618824	11	62.8	37.5	40.6	15.6
chr7	Il18bp	5-UTR	N	109165829	109166669	4	64.2	35.5	54.5	29.6
chr18	Sh3rf2	5-UTR	N	42213364	42213471	1	25.5	7.3	24.6	0.0
chr18	Gm9926	5-UTR	N	66663832	66664116	1	46.3	22.2	38.8	14.2
chr17	Wash	5-UTR	N	66460886	66463207	9	75.9	57.4	90.6	66.3
chr13	Cenpk	5-UTR	N	105018691	105020842	6	31.4	8.3	31.8	7.7
chr7	BC051019	5-UTR	N	116866800	116867285	2	77.9	62.2	62.4	38.5
chr7	Oat	5-UTR	N	139761788	139768081	27	75.7	25.2	58.3	34.6
chr6	Rep15	5-UTR	N	146981059	146981186	1	68.7	37.6	50.9	27.2
chr11	Scgb3a1	5-UTR	N	49477097	49477162	1	40.0	9.1	30.3	6.7
chr18	Zfp191	5-UTR	N	24176589	24179272	10	17.2	2.1	26.1	2.5
chr17	Btnl5	5-UTR	N	34634008	34634374	2	73.6	12.3	51.4	27.8
chr4	Stmn1	5-UTR	N	134024235	134026077	3	56.1	14.6	40.3	17.0
chr4	AU040320	5-UTR	N	126430799	126434170	13	51.9	34.8	46.0	23.2
chr1	Ivns1abp	5-UTR	N	153191628	153198128	25	51.8	24.7	43.5	20.7
chr16	Tmprss2	5-UTR	N	97820877	97832802	55	53.6	36.6	50.5	27.8

chr19	Aldh18a1	5-UTR	N	40660068	40662953	11	59.8	41.8	72.4	50.0
chr2	4930547E08Rik	5-UTR	N	103650630	103650779	1	63.6	30.1	62.1	39.8
chr10	Tmcc3	5-UTR	N	94038002	94041182	17	64.3	21.3	50.3	28.2
chr4	Sdr16c5	5-UTR	N	3943572	3946810	16	64.8	49.1	55.4	33.6
chr14	Rnase1	5-UTR	N	51765571	51766442	4	73.1	25.7	88.1	66.4
chr7	Arfip2	5-UTR	N	112787938	112788930	3	30.2	0.0	37.9	16.7
chr15	Gm15941	5-UTR	N	37362169	37362419	2	83.1	35.7	52.5	31.4
chr11	Il4	5-UTR	N	53432109	53432167	1	64.5	40.6	67.0	46.1
chr4	Aldh1b1	5-UTR	N	45811894	45815335	15	33.8	6.7	32.3	11.5
chr11	Tbrg4	5-UTR	N	6524224	6526070	7	42.4	19.6	47.2	26.5
chr5	Hps4	5-UTR	N	112772115	112774027	7	43.8	8.7	37.3	16.8
chr13	Mterfd1	5-UTR	N	67031140	67034008	12	90.1	61.9	91.2	70.8
chr5	Sult1d1	5-UTR	N	87995209	87998031	13	60.6	25.0	41.0	20.7
chr6	Tmem52b	5-UTR	N	129462575	129463056	2	52.3	36.4	65.4	45.4
chr4	Mir200a	5-UTR	N	155429005	155429094	1	24.5	0.7	20.7	0.7
chr2	Oser1	5-UTR	N	163241348	163245206	16	48.6	31.1	54.0	34.7
chr7	Cln3	5-UTR	N	133726560	133727794	3	36.5	21.4	35.8	16.7
chr6	Mgst1	5-UTR	N	138089058	138096210	36	57.7	29.5	52.9	33.9
chr11	Lgals9	5-UTR	N	78798319	78798426	1	35.2	0.0	20.0	1.3
chr18	Tslp	5-UTR	N	32975037	32975053	1	83.3	65.9	71.6	53.0
chr1	Neu2	5-UTR	N	89470602	89476079	28	59.7	42.4	51.4	32.9
chr1	Pikfyve	5-UTR	N	65233259	65236901	15	33.6	0.0	38.1	19.8
chr15	Eppk1	5-UTR	N	75943110	75950625	35	64.9	48.8	63.3	45.6
chr1	Faim3	5-UTR	N	132762354	132762436	1	88.9	50.0	70.4	52.7
chr11	Lypd8	5-UTR	N	58192534	58195834	16	33.8	16.2	28.4	11.1
chr4	Hdhd3	5-UTR	N	62160972	62163234	9	71.0	49.3	50.3	33.2
chr1	Fhl2	5-UTR	N	43210077	43220806	50	71.9	43.7	65.0	47.9
chr14	Dct	5-UTR	N	118451001	118451468	2	20.9	4.9	22.3	5.3
chr19	Hps1	5-UTR	N	42852729	42854466	6	83.8	68.5	84.0	67.0
chr5	Brca2	5-UTR	N	151325198	151325787	1	30.0	10.4	32.9	16.3
chr6	Cacna1c	5-UTR	N	119146250	119146427	1	85.0	66.7	82.6	66.0
chr11	Prr15l	5-UTR	N	96790638	96795859	26	43.4	25.2	32.8	16.5
chr18	Atp8b1	5-UTR	N	64765033	64820654	269	70.3	54.1	66.4	50.1
chr1	Adck3	5-UTR	N	182112370	182126151	66	58.7	39.2	55.0	38.9
chr10	Rdh1	5-UTR	N	127196819	127196993	1	32.4	0.0	19.0	3.0
chr15	Tmbim6	5-UTR	N	99223378	99232038	39	46.1	28.8	49.0	33.4
chr2	4930526D03Rik	5-UTR	N	181432361	181433147	4	79.0	56.1	70.1	54.6
chr11	Gm11545	5-UTR	N	94622226	94622496	1	17.0	0.0	20.0	4.8
chr8	Sf3b3	5-UTR	N	113368433	113370703	8	61.1	41.6	55.2	40.0
chr12	Slc25a29	Exon1	N	110065884	110065967	1	66.0	8.5	86.6	5.4
chr17	Rgmb	Exon1	N	15957634	15958142	3	81.0	18.9	84.1	15.7
chr8	Cyba	Exon1	N	124950086	124950167	1	71.4	18.2	73.9	14.1
chr7	Ccdc9	Exon1	N	16860605	16860724	2	87.5	48.1	78.4	20.4
chr8	Ccdc102a	Exon1	N	97429157	97429260	1	85.4	25.7	90.1	32.6
chr19	Ms4a18	Exon1	N	11074825	11074938	1	91.5	30.8	87.3	30.3
chr18	Grpel2	Exon1	N	61878408	61878489	1	66.2	26.3	74.7	20.4
chr8	Ctcf	Exon1	N	108167416	108167532	2	82.7	64.7	79.1	25.8
chr14	9230112D13Rik	Exon1	N	35327566	35327652	1	56.5	5.0	60.2	8.1
chr5	Pdgfa	Exon1	N	139455100	139455226	1	71.1	20.7	78.0	27.3

chr11	Nmt1	Exon1	N	102904482	102904590	1	87.4	42.0	87.8	38.2
chr2	Ap5s1	Exon1	N	131036999	131037209	2	35.7	9.7	59.8	11.4
chr4	Triqk	Exon1	N	12845063	12845212	2	56.5	0.0	61.9	14.6
chr5	Hps4	Exon1	N	112773561	112774068	4	54.4	5.1	59.2	13.5
chr7	Mrpl48	Exon1	N	107699054	107699143	1	42.1	14.3	54.7	9.2
chr9	Ifrd2	Exon1	N	107492234	107492353	1	91.4	36.7	74.6	30.3
chr11	Axin2	Exon1	N	108784466	108785416	5	23.4	1.3	45.3	1.7
chr8	Ces1b	Exon1	N	95581255	95581327	1	68.2	40.0	58.1	14.7
chr17	Foxn2	Exon1	N	88862053	88862603	4	41.8	7.2	47.0	3.8
chr2	Tm9sf4	Exon1	N	153004625	153004738	1	73.0	24.6	75.0	32.3
chr19	Ms4a8a	Exon1	N	11142865	11142978	1	51.4	27.8	49.3	6.9
chr1	Fam129a	Exon1	N	153483534	153483664	1	97.9	55.7	89.3	48.1
chr14	Nuggc	Exon1	N	66225540	66225600	1	62.6	17.1	69.1	30.2
chr5	5033403H07Rik	Exon1	N	53381828	53381976	1	21.1	1.7	43.3	4.8
chr7	Hsd3b7	Exon1	N	134944589	134944760	3	86.8	54.5	89.8	51.9
chr8	Slc25a42	Exon1	N	72712199	72712350	1	83.3	37.4	85.3	48.1
chr6	Mettl20	Exon1	N	149091128	149091237	2	47.8	14.7	48.9	12.4
chr2	Tubgcp4	Exon1	N	120999294	120999422	1	74.7	39.1	78.4	42.0
chr6	Tpi1	Exon1	N	124761535	124761622	1	52.3	20.7	56.9	21.1
chr10	Specc1l	Exon1	N	74693427	74693616	2	46.1	2.5	35.7	0.0
chr1	Tmem63a	Exon1	N	182876697	182876896	2	63.5	40.5	55.7	20.6
chr5	Vsig10	Exon1	N	117773509	117773589	1	36.4	6.3	42.3	7.4
chr1	Aldh9a1	Exon1	N	169282651	169282796	1	55.2	14.0	47.4	12.7
chr2	Garnl3	Exon1	N	32845026	32845152	1	66.2	32.9	60.4	25.9
chr6	Pzp	Exon1	N	128434882	128434923	1	65.2	45.2	86.0	51.4
chr13	Nnt	Exon1	N	120127183	120127298	1	96.5	42.7	91.5	57.1
chr1	Sec16b	Exon1	N	159437517	159437684	2	50.8	16.2	47.6	13.2
chr17	Igf2r	Exon1	N	12878004	12878073	1	67.9	19.5	72.1	38.1
chr4	Rps8	Exon1	N	116826904	116827033	1	38.9	15.4	56.3	22.4
chr4	Hivep3	Exon1	N	119669779	119669857	2	79.2	47.5	87.5	53.8
chr3	Dennd2d	Exon1	N	106289791	106289966	2	84.0	59.2	85.6	52.5
chr9	Vipr1	Exon1	N	121562917	121563022	1	76.6	41.0	75.3	42.7
chr16	Gpr156	Exon1	N	37947454	37947905	3	92.9	64.4	95.9	63.8
chr17	Airn	Exon1	N	13051973	13052118	2	45.4	13.7	54.4	22.5
chr19	Coro1b	Exon1	N	4149342	4149544	2	65.8	41.2	60.7	28.9
chr15	Stk3	Exon1	N	34874761	34874936	1	76.0	41.5	90.7	59.0
chr2	Itga6	Exon1	N	71654687	71654811	1	58.2	32.4	60.9	29.6
chr11	Krt39	Exon1	N	99377779	99377999	1	41.4	18.6	57.5	26.3
chr5	Plb1	Exon1	N	32657159	32657263	1	89.5	68.0	85.6	54.9
chr1	Fastkd2	Exon1	N	63777974	63778795	5	91.3	54.9	73.6	43.1
chr10	Cand1	Exon1	N	118643772	118643879	1	76.4	20.0	65.4	34.9
chr17	Mlst8	Exon1	N	24612991	24613154	2	88.0	69.0	100.0	70.0
chr13	Serpinb1b	Exon1	N	33177147	33177322	2	73.9	25.0	61.2	32.3
chr5	Daglb	Exon1	N	144227821	144227972	1	78.6	19.9	63.2	34.6
chr1	Gsta3	Exon1	N	21235019	21235113	2	82.0	51.2	82.5	54.4
chr17	Cyp4f16	Exon1	N	32673943	32674141	2	44.7	21.7	51.8	23.7
chr13	Fam169a	Exon1	N	97861773	97861907	2	44.3	1.8	37.3	9.5
chr14	Ldb3	Exon1	N	35349751	35349866	1	83.8	12.8	75.6	48.1
chr5	Hadha	Exon1	N	30446585	30446730	1	48.8	17.6	48.3	21.0

chr4	Selrc1	Exon1	N	108004816	108004956	1	33.8	4.3	36.0	8.8
chr4	Stmn1	Exon1	N	134026018	134026090	2	56.1	17.4	48.3	21.2
chr13	BC005537	Exon1	N	24896651	24896827	1	57.6	18.4	50.9	24.0
chr5	Cyth3	Exon1	N	144445265	144445347	2	89.0	61.5	89.4	62.6
chr18	Mbd1	Exon1	N	74429057	74429192	2	39.8	16.5	44.0	17.8
chr4	Aco1	Exon1	N	40110752	40110878	2	91.5	71.1	88.2	62.0
chr12	Hbp1	Exon1	N	32613449	32613590	1	21.4	4.4	33.7	8.0
chr6	Casp2	Exon1	N	42217498	42217648	1	66.2	28.7	53.4	28.4
chr1	Traf3ip3	Exon1	N	195004358	195004494	2	87.5	45.2	70.9	45.9
chr3	Slc2a2	Exon1	N	28604652	28604744	1	62.9	17.6	53.9	28.9
chr8	Cox4i1	Exon1	N	123193209	123193282	2	62.9	44.3	64.1	39.8
chr8	Lrrc8e	Exon1	N	4231662	4231804	2	54.5	34.7	51.6	27.8
chr10	Agap2	Exon1	N	126519420	126519478	1	31.9	10.2	40.7	16.8
chr7	Kcne3	Exon1	N	107326497	107326752	2	21.5	2.7	26.3	2.6
chr6	Slc15a5	Exon1	N	137935894	137936002	1	83.3	65.6	82.9	59.4
chr2	Cdca7	Exon1	N	72316963	72317085	1	94.7	60.3	91.3	68.5
chr12	4930465M20Rik	Exon1	N	108967706	108968049	2	54.7	12.1	43.1	20.3
chr7	Vrk3	Exon1	N	52007485	52007546	1	87.0	63.2	82.8	60.2
chr6	Slco1b2	Exon1	N	141581602	141581685	2	95.1	69.6	86.5	64.6
chr4	Alad	Exon1	N	62171139	62171268	1	88.9	73.8	88.6	67.2
chr5	Fam109a	Exon1	N	122300479	122300555	1	29.5	5.7	27.2	6.0
chr6	Rpl32	Exon1	N	115757004	115757185	1	26.7	6.3	28.7	7.8
chr5	1700007G11Rik	Exon1	N	98775251	98775381	1	74.5	53.7	74.0	53.2
chr8	5430403N17Rik	Exon1	N	36934240	36934466	1	81.7	58.5	76.2	55.6
chr16	Tmprss7	Exon1	N	45658156	45658292	1	35.9	3.6	24.3	3.7
chr2	Slc9a8	Exon1	N	167249619	167249797	1	78.4	63.0	81.8	61.6
chr5	Dtx2	Exon1	N	136472160	136472322	2	87.9	66.3	86.8	66.7
chr1	Dars2	Exon1	N	162973744	162973819	1	23.3	0.0	20.0	0.0
chr13	Gcnt2	Exon1	N	41048945	41049040	1	80.6	63.0	78.9	58.9
chr2	Acvr2a	Exon1	N	48725809	48726016	1	81.8	42.7	56.0	36.1
chr18	Dym	Exon1	N	75202733	75202923	2	87.5	67.5	82.7	62.8
chr15	4930415O20Rik	Exon1	N	98415701	98415823	1	22.3	2.3	22.0	2.2
chr14	Gnpnat1	Exon1	N	46000612	46000673	1	64.4	28.7	41.7	22.1
chr9	Mrps22	Exon1	N	98490454	98490562	1	87.1	55.2	83.0	63.6
chr3	Nhlrc3	Exon1	N	53257464	53257576	1	79.1	63.2	79.2	60.2
chr16	Mgrn1	Exon1	N	4907365	4907486	1	80.9	49.1	68.8	50.0
chr17	Dus3l	Exon1	N	56904948	56905215	1	46.7	25.4	48.8	30.4
chr7	Rab30	Exon1	N	99968493	99968593	2	59.0	25.6	45.2	26.8
chr11	Clint1	Exon1	N	45697212	45697316	1	51.2	25.4	44.6	26.3
chr17	4833427F10Rik	Exon1	N	35913821	35914168	2	23.4	1.1	26.0	7.9
chr6	Hrh1	Exon1	N	114358768	114358914	2	77.8	54.7	79.6	61.6
chr8	Mbtps1	Exon1	N	122034154	122034284	1	84.1	55.3	88.9	71.1
chr17	Mrpl28	Exon1	N	26261501	26261653	1	70.6	41.9	60.8	43.4
chr9	Slc35f2	Exon1	N	53645645	53645820	1	70.4	45.8	63.1	45.9
chr11	9530048J24Rik	Exon1	N	85581065	85581335	1	96.9	80.0	97.7	80.5
chr10	Pbld1	Exon1	N	62528532	62528631	1	33.3	4.5	19.7	2.5
chr13	Gprin1	Exon1	N	54843370	54843469	2	77.1	59.5	82.2	65.1
chr8	Nqo1	Exon1	N	109914945	109915046	1	74.8	51.9	67.6	50.8
chr1	Pm20d1	Exon1	N	133695173	133695259	1	69.4	44.0	70.3	53.5

chr17	Ly6g6c	Exon1	N	35205812	35205922	1	39.5	24.2	50.7	33.9
chr17	Pou5f1	Exon1	N	35646048	35646168	2	55.7	40.6	54.7	38.0
chr5	Tmem120a	Exon1	N	136211767	136211835	1	85.8	56.0	92.5	75.9
chr6	Zxdc	Exon1	N	90322536	90322688	1	55.3	30.3	54.0	37.6
chr13	Prelid1	Exon1	N	55424214	55424439	2	52.1	33.4	49.4	33.1
chr17	Bnip1	Exon1	N	26920390	26920482	1	91.3	67.4	86.8	70.6
chr4	Srsf10	Exon1	N	135414220	135414324	1	64.7	39.6	62.8	46.9
chr9	Gm10684	Exon1	N	44918068	44918842	4	84.5	65.1	86.5	70.6
chr9	Taf1d	Exon1	N	15112172	15112265	2	57.3	25.0	60.2	44.3
chr3	Arhgap29	Exon1	N	121676797	121677033	2	67.0	36.7	57.3	41.5
chr13	Vps41	Exon1	N	18815057	18815095	1	76.6	56.6	75.0	60.0
chr1	Pigr	Exon1	N	132723261	132723384	1	71.9	7.7	70.8	2.7
chr10	Vnn3	Exon1	N	23571268	23571602	2	80.2	0.0	67.7	0.6
chr15	Apol10a	Exon1	N	77307477	77307561	1	86.2	11.0	87.1	24.8
chr13	Naip1	Exon1	N	101222753	101222819	1	51.7	6.1	64.6	6.5
chr17	Slc22a1	Exon1	N	12868112	12868704	3	55.3	11.9	70.3	14.7
chr17	Xdh	Exon1	N	74299418	74299522	2	69.6	0.3	55.5	0.0
chr12	Agmo	Exon1	N	37968226	37968754	3	28.8	9.7	58.6	7.4
chr8	Snx20	Exon1	N	91159885	91160027	1	74.6	26.1	73.4	24.1
chr9	Casp1	Exon1	N	5298517	5298723	2	56.2	3.1	48.2	0.8
chr8	Ces2c	Exon1	N	107370968	107371108	2	53.8	10.7	52.9	6.1
chr7	Tmem150b	Exon1	N	4676510	4676853	3	40.3	3.1	48.5	1.7
chr11	Gm11437	Exon1	N	83980829	83980978	2	50.9	4.0	85.1	39.0
chr5	Azgp1	Exon1	N	138422749	138422848	2	38.1	0.0	48.7	4.7
chr5	Hsd17b13	Exon1	N	104406125	104406407	2	93.9	10.7	84.4	41.1
chr8	Ces2g	Exon1	N	107485618	107485763	2	40.5	0.0	42.9	0.4
chr1	Aox3	Exon1	N	58169980	58170156	2	54.2	31.1	48.9	7.3
chr10	Rdh1	Exon1	N	127196819	127197306	3	58.8	0.0	64.4	23.4
chr10	Mettl7b	Exon1	N	128397497	128398044	3	76.4	4.2	47.5	6.9
chr1	Apobec4	Exon1	N	154597681	154597894	2	44.1	3.3	50.5	10.3
chr12	Numb	Exon1	N	85183168	85183308	2	72.8	40.8	82.9	44.2
chr17	Acer1	Exon1	N	57121412	57121549	2	42.6	23.5	59.6	21.5
chr7	Ceacam20	Exon1	N	20550761	20550888	2	41.7	0.0	38.1	0.0
chr6	Aqp1	Exon1	N	55286293	55286868	3	79.5	9.2	56.5	18.5
chr4	Cyp4b1	Exon1	N	115320113	115320310	2	61.6	18.4	61.1	23.4
chr7	Dmpk	Exon1	N	19669198	19669503	2	39.5	5.4	41.5	7.0
chr3	Arl14	Exon1	N	69026341	69027540	7	26.8	10.2	38.9	6.0
chr18	Sh3rf2	Exon1	N	42213364	42213849	3	25.5	7.3	43.2	10.6
chr7	Cln3	Exon1	N	133727688	133727794	1	52.7	20.2	46.0	13.6
chr7	Mrgprd	Exon1	N	152500740	152501188	3	91.1	70.3	91.9	59.6
chr1	Abca12	Exon1	N	71461088	71461484	3	52.9	21.8	61.7	30.0
chr14	Acox2	Exon1	N	9091336	9091533	2	66.1	9.8	33.0	1.4
chr13	Naip6	Exon1	N	101085939	101086571	4	65.1	11.5	52.6	21.0
chr13	Tubal3	Exon1	N	3923941	3923983	2	86.7	26.1	31.4	0.0
chr8	Tnfsf13b	Exon1	N	10006633	10007160	2	46.4	21.3	58.7	27.8
chr9	Nr2e3	Exon1	N	59797595	59797886	2	54.3	23.1	52.3	21.5
chr1	Slc9a4	Exon1	N	40637072	40637616	2	17.0	1.0	33.7	3.0
chr4	Tnfrsf18	Exon1	N	155400451	155400646	2	33.4	16.4	44.5	13.9
chr3	P2ry1	Exon1	N	60806717	60808521	4	62.2	13.6	57.3	26.9

chr8	Il17c	Exon1	N	124945985	124946305	2	88.3	37.1	81.9	52.1
chr5	1700018F24Rik	Exon1	N	145803859	145804037	2	76.2	50.7	69.1	40.3
chr2	4833423E24Rik	Exon1	N	85358585	85359092	3	72.7	49.5	77.8	49.2
chr7	Apoc4	Exon1	N	20266654	20266809	2	34.6	9.1	73.7	45.4
chr19	Kcnk7	Exon1	N	5704476	5704794	2	61.4	44.6	53.5	25.2
chr17	Nlrc4	Exon1	N	74858346	74858448	1	28.0	3.1	27.9	0.8
chr10	Rdh16	Exon1	N	127238209	127238566	3	88.4	50.9	90.5	64.5
chr7	Muc2	Exon1	N	148876239	148876343	2	34.8	14.6	35.6	9.8
chr3	Fabp2	Exon1	N	122597990	122598397	3	41.4	0.3	32.6	6.8
chr7	Gm7092	Exon1	N	26400682	26400911	2	43.4	7.9	30.6	6.7
chr3	Ctss	Exon1	N	95330708	95330847	2	42.3	10.8	39.2	15.4
chr7	BC051019	Exon1	N	116866918	116867285	2	77.9	62.2	62.4	38.5
chr7	Zfp541	Exon1	N	16657291	16657597	2	43.4	11.5	34.1	10.4
chr11	Scgb3a1	Exon1	N	49477097	49477217	1	40.0	9.1	30.3	6.7
chr10	Lyz1	Exon1	N	116729759	116729924	2	39.0	17.0	52.3	28.8
chr6	Akr1b7	Exon1	N	34362362	34362475	2	67.6	37.5	58.6	36.1
chr19	Pnliprp2	Exon1	N	58834213	58834366	2	63.2	24.5	50.4	28.1
chr17	Slc3a1	Exon1	N	85427687	85428198	2	48.6	28.4	81.1	59.0
chr1	Mars2	Exon1	N	55294021	55296902	7	45.8	3.2	38.6	17.1
chr10	Ros1	Exon1	N	51914637	51915050	2	69.6	39.1	63.2	41.7
chr6	H2afj	Exon1	N	136756769	136758595	3	42.4	12.5	26.7	5.5
chr10	Gm19402	Exon1	N	77152702	77153502	4	52.5	4.1	45.6	24.6
chr7	Leng9	Exon1	N	4099785	4101474	3	69.0	52.4	54.3	33.3
chr2	Tldc2	Exon1	N	156912791	156912823	1	26.1	0.0	20.4	0.0
chr16	Gpr128	Exon1	N	56795660	56795971	2	16.2	0.6	20.2	0.0
chr6	Tmem52b	Exon1	N	129462575	129463122	3	52.3	36.4	65.4	45.4
chr11	Krtap3-1	Exon1	N	99427341	99427944	3	88.8	52.2	73.2	53.2
chr6	Cml5	Exon1	N	85770901	85770966	1	65.7	30.4	60.7	41.6
chr4	Olfr1337	Exon1	N	118454241	118455191	6	77.4	60.0	85.3	66.1
chr7	Olfr644	Exon1	N	111216599	111217543	5	77.6	54.9	68.8	50.0
chr11	Lgals9	Exon1	N	78798280	78798426	2	62.4	9.6	20.0	1.3
chr2	Ifih1	Exon1	N	62483535	62484312	1	59.6	29.2	36.4	18.0
chr11	Hoxb3	Exon1	N	96184440	96184759	2	88.1	63.8	88.5	70.2
chr7	Mia	Exon1	N	27965941	27966168	2	57.3	39.6	48.9	30.7
chr9	Slc51b	Exon1	N	65270554	65270580	1	58.5	15.3	40.2	22.1
chr19	Olfr1502	Exon1	N	13936285	13937235	5	96.9	60.0	64.8	46.9
chr19	Naaladl1	Exon1	N	6105798	6105997	1	38.5	18.6	34.6	16.8
chr6	Vmn1r4	Exon1	N	56906507	56907400	4	78.7	62.7	76.4	58.6
chr1	Faim3	Exon1	N	132762354	132762473	2	88.9	50.0	70.4	52.7
chr5	Gm5108	Exon1	N	68332908	68333099	2	51.3	31.2	35.6	18.2
chr8	F7	Exon1	N	13026034	13026131	2	70.8	45.0	57.1	40.0
chr8	Stox2	Exon1	N	48437385	48437702	2	20.3	0.0	18.7	1.7
chr7	Crebzf	Exon1	N	97591291	97596553	18	59.1	24.8	40.7	24.0
chr11	Lpo	Exon1	N	87639244	87639616	2	33.4	15.0	35.9	19.1
chr6	Cacna1c	Exon1	N	119146111	119146427	2	85.0	66.7	82.6	66.0
chr3	Rbm15	Exon1	N	107133133	107136207	8	38.2	18.6	43.9	27.4
chr6	Olfr441	Exon1	N	43065743	43066675	5	73.3	51.0	73.5	57.3
chr7	Olfr661	Exon1	N	111836531	111837490	5	88.2	70.2	90.9	75.0
chr2	Bpifa1	Exon1	N	153968616	153968662	1	73.1	56.9	73.2	57.4

chr2	Olfr1129	Exon1	N	87415243	87416187	5	97.4	66.7	88.9	73.1
chr10	Taar9	Exon1	N	23828294	23829340	5	91.3	58.4	77.3	61.8
chr14	Sftpd	Exon1	N	41998414	41998487	1	74.6	50.7	63.7	48.5
chr8	Proz	Exon1	N	13060908	13061099	2	73.2	38.5	43.1	27.9
chr14	Mat1a	Exon1	N	41918322	41918549	2	67.6	52.1	70.3	55.3
chr9	Casp1	Upstream	N	5297517	5298516	5	47.9	6.5	85.6	2.9
chr15	Apol10a	Upstream	N	77306477	77307476	5	64.0	3.0	82.9	8.5
chr3	Hsd3b3	Upstream	N	98567052	98568051	6	91.1	26.9	92.9	26.2
chr9	Zw10	Upstream	N	48862686	48863685	4	89.1	1.8	59.3	0.0
chr1	Pigr	Upstream	N	132722261	132723260	5	77.8	26.1	78.8	21.6
chr9	Ctnnb1	Upstream	N	120841518	120842517	2	33.5	3.9	63.0	6.5
chr7	Olfr713	Upstream	N	114178650	114179649	5	70.3	43.2	87.8	33.2
chr2	Fam107b	Upstream	N	3629730	3630729	3	66.7	32.8	63.5	9.4
chr16	Mir802	Upstream	N	93368965	93369964	5	71.8	10.0	74.0	20.4
chr6	Avl9	Upstream	N	56663899	56664898	3	64.6	25.0	72.1	18.9
chr5	Ccl26	Upstream	N	136039440	136040439	5	37.6	14.3	68.6	15.9
chr2	Prrg4	Upstream	N	104690008	104691007	4	55.5	12.1	57.2	4.8
chr3	Il21	Upstream	N	37131541	37132540	4	82.6	56.0	81.0	28.6
chr11	Gm12250	Upstream	N	57996345	57997344	5	81.6	12.4	69.7	17.8
chr16	Dnaja3	Upstream	N	4683070	4684069	3	28.0	9.9	53.3	3.5
chr14	Usp54	Upstream	N	21437577	21438576	5	75.9	33.3	78.4	29.1
chr19	E030003E18Rik	Upstream	N	20566205	20567204	5	67.7	23.1	72.3	23.1
chr7	C5ar2	Upstream	N	16829504	16830503	5	85.1	48.5	96.1	47.3
chr7	Olfr690	Upstream	N	112478793	112479792	5	72.9	17.2	76.0	27.5
chr11	Slc22a5	Upstream	N	53705206	53706205	2	35.4	13.7	56.9	8.9
chr19	Ifit3	Upstream	N	34657019	34658018	5	72.0	52.2	83.1	37.1
chr5	Niacr1	Upstream	N	124315526	124316525	5	90.9	71.0	91.0	45.2
chr2	Cd302	Upstream	N	60122476	60123475	5	47.3	25.3	52.7	6.9
chr9	Tmem123	Upstream	N	7763077	7764076	2	47.1	29.0	69.5	24.2
chr11	Atp10b	Upstream	N	42962379	42963378	5	57.8	21.6	47.7	2.4
chr9	Nnmt	Upstream	N	48413183	48414182	5	37.1	6.1	55.7	10.5
chr15	Krt81	Upstream	N	101294197	101295196	5	58.6	17.8	58.5	13.5
chr17	Cyp4f14	Upstream	N	33054288	33055287	5	64.8	4.9	58.0	13.8
chr14	Diap3	Upstream	N	87540922	87541921	3	58.9	13.1	63.2	19.1
chr12	Tomm20l	Upstream	N	72211415	72212414	4	60.9	15.1	71.9	28.8
chr11	E030025P04Rik	Upstream	N	109005684	109006683	5	55.7	26.8	69.0	26.0
chr10	Utp20	Upstream	N	88289560	88290559	4	43.9	0.8	43.8	0.8
chr17	Kif6	Upstream	N	49753497	49754496	3	79.6	33.1	42.3	0.0
chr6	Pzp	Upstream	N	128476739	128477738	5	80.0	25.0	56.0	14.0
chr11	Hoxb9	Upstream	N	96131644	96132643	2	34.3	10.6	46.5	4.6
chr18	Commd10	Upstream	N	47117530	47118529	3	44.6	0.8	43.4	1.5
chr14	Epsti1	Upstream	N	78303046	78304045	5	54.1	5.8	50.8	9.5
chr4	Gem	Upstream	N	11630594	11631593	3	67.3	37.4	69.1	28.3
chr9	Gorasp1	Upstream	N	119846677	119847676	3	59.2	36.2	80.7	39.9
chr18	Ttr	Upstream	N	20822751	20823750	5	42.9	27.3	40.6	0.0
chr4	Mup21	Upstream	N	61811876	61812875	5	54.9	38.5	77.0	36.7
chr1	Stx6	Upstream	N	157004833	157005832	3	84.5	54.0	95.8	56.0
chr7	Tmem86b	Upstream	N	4582085	4583084	5	65.6	1.7	48.8	9.7
chr7	Gde1	Upstream	N	125849253	125850252	4	78.9	57.0	91.5	52.5

chr16	Cpox	Upstream	N	58669321	58670320	3	54.5	12.0	56.6	18.0
chr3	Adh6a	Upstream	N	137975250	137976249	5	81.3	48.6	86.9	48.5
chr3	Lrrc39	Upstream	N	116264891	116265890	5	83.6	28.9	74.1	35.8
chr11	Dusp18	Upstream	N	3794243	3795242	1	56.6	39.5	64.6	26.4
chr5	Fam175a	Upstream	N	101249955	101250954	3	51.3	31.8	54.2	16.2
chr15	Adck5	Upstream	N	76405789	76406788	4	64.7	22.7	62.2	24.5
chr11	Chac2	Upstream	N	30886366	30887365	5	33.7	16.8	48.5	10.7
chr13	Txndc5	Upstream	N	38620330	38621329	1	74.5	39.2	72.8	35.3
chr19	As3mt	Upstream	N	46780933	46781932	4	87.3	42.6	40.6	3.5
chr17	H2-M5	Upstream	N	37126450	37127449	5	57.8	39.0	71.2	34.3
chr7	Trim6	Upstream	N	111366309	111367308	4	88.6	50.8	49.7	12.8
chr7	Sult1a1	Upstream	N	133819872	133820871	5	37.5	6.9	41.0	4.2
chr11	Stat5a	Upstream	N	100719665	100720664	5	73.4	47.8	80.5	43.7
chr5	5830473C10Rik	Upstream	N	90989235	90990234	5	84.7	57.4	84.4	47.8
chr7	Rccd1	Upstream	N	87469341	87470340	4	74.7	53.2	70.6	34.1
chr10	Rdh9	Upstream	N	127212461	127213460	5	80.6	34.1	38.6	2.9
chr17	Xdh	Upstream	N	74299523	74300522	5	54.2	28.2	56.4	20.7
chr16	Il1rap	Upstream	N	26580791	26581790	2	87.5	57.1	82.5	47.2
chr6	Gabarapl1	Upstream	N	129482210	129483209	4	60.4	29.6	40.1	5.2
chr14	Rnase12	Upstream	N	51676918	51677917	5	55.7	29.5	61.3	26.5
chr15	Csf2rb	Upstream	N	78155420	78156419	5	61.8	38.0	60.7	26.1
chr11	Gas7	Upstream	N	67345500	67346499	5	36.7	4.8	43.1	8.8
chr11	A430071A18Rik	Upstream	N	119695680	119696679	5	64.7	3.3	35.5	1.4
chr7	Dmpk	Upstream	N	19668198	19669197	5	59.7	35.3	57.7	24.0
chr6	Cftr	Upstream	N	18119687	18120686	2	44.5	14.1	46.2	12.6
chr4	Rgs3	Upstream	N	62279706	62280705	5	77.8	51.4	78.5	45.0
chr5	Tlr1	Upstream	N	65324798	65325797	5	39.7	5.6	81.5	48.2
chr7	Klk1b11	Upstream	N	51250249	51251248	5	36.6	7.4	43.1	10.0
chr17	Cyp4f40	Upstream	N	32795428	32796427	5	78.8	33.6	75.7	42.7
chr6	A2m	Upstream	N	121585191	121586190	5	91.3	66.0	56.7	23.8
chr17	Ppm1b	Upstream	N	85372392	85373391	5	82.2	52.9	78.0	45.1
chr16	Zfp263	Upstream	N	3743099	3744098	2	40.3	2.1	38.2	5.4
chr3	Gstm7	Upstream	N	107734664	107735663	5	34.9	4.7	35.4	2.9
chr10	9230102K24Rik	Upstream	N	110036706	110037705	5	28.9	3.7	33.1	1.1
chr12	Acyp1	Upstream	N	86621386	86622385	4	35.3	5.9	69.1	37.1
chr2	BC039771	Upstream	N	145525931	145526930	5	73.1	14.9	66.6	34.7
chr8	Nat3	Upstream	N	70046753	70047752	5	88.4	22.2	62.1	30.2
chr7	Psg23	Upstream	N	19201851	19202850	5	34.7	4.7	34.1	2.4
chr17	Fbxl17	Upstream	N	63849930	63850929	3	94.0	68.9	86.6	55.1
chr18	Htr4	Upstream	N	62482858	62483857	2	57.6	29.8	65.4	34.0
chr11	St6galnac2	Upstream	N	116555975	116556974	3	26.3	9.5	40.6	9.4
chr11	Dhrs11	Upstream	N	84642506	84643505	2	91.6	59.7	83.7	52.6
chr17	2010106C02Rik	Upstream	N	86686519	86687518	5	100.0	55.7	67.2	36.8
chr5	Btc	Upstream	N	91832021	91833020	5	47.8	0.0	30.9	0.7
chr13	Naip2	Upstream	N	100972048	100973047	5	61.8	38.5	68.7	38.5
chr8	Mir138-2	Upstream	N	96847211	96848210	5	73.0	56.5	84.4	54.2
chr4	Isg15	Upstream	N	155574928	155575927	5	68.3	38.2	70.0	40.0
chr5	Cxcl5	Upstream	N	91187324	91188323	5	63.4	37.2	46.4	16.6
chr4	Acot7	Upstream	N	151551209	151552208	1	31.8	5.2	35.8	6.0

chr7	Ceacam12	Upstream	N	18650278	18651277	5	61.5	27.7	72.6	42.9
chr8	Tnfsf13b	Upstream	N	10005633	10006632	5	54.1	14.4	47.8	18.1
chr15	5430437J10Rik	Upstream	N	5445318	5446317	5	82.8	64.9	74.4	44.8
chr1	Cd55	Upstream	N	132359318	132360317	4	64.5	43.3	76.5	46.9
chr2	Patl2	Upstream	N	122011926	122012925	3	25.6	5.8	38.1	8.5
chr17	Lhfpl5	Upstream	N	28711664	28712663	2	55.1	29.9	78.6	49.4
chr7	Atp1a3	Upstream	N	25790915	25791914	3	77.0	50.0	77.8	48.6
chr8	Snapc2	Upstream	N	4252102	4253101	3	40.9	20.4	48.6	19.4
chr14	Adamdec1	Upstream	N	69200130	69201129	5	100.0	45.0	94.1	65.0
chr11	BC018473	Upstream	N	116612481	116613480	5	34.6	15.0	45.9	17.0
chr2	8030442B05Rik	Upstream	N	11320128	11321127	5	44.5	17.5	48.6	19.8
chr19	Lipn	Upstream	N	34140848	34141847	5	69.7	31.9	68.6	40.1
chr1	Smyd2	Upstream	N	191746168	191747167	2	45.3	4.0	34.2	5.8
chr19	Pdcd4	Upstream	N	53965721	53966720	5	54.7	25.0	53.0	24.8
chr14	Il17rb	Upstream	N	30822083	30823082	3	25.9	3.7	28.2	0.0
chr15	Efr3a	Upstream	N	65617603	65618602	3	59.5	38.5	58.1	30.2
chr4	Rnu11	Upstream	N	131826102	131827101	3	53.6	9.6	54.5	26.7
chr3	Gbp2	Upstream	N	142282627	142283626	5	27.4	11.3	40.8	13.1
chr19	Tmem252	Upstream	N	24747498	24748497	5	74.1	6.0	45.5	17.9
chr17	Prr18	Upstream	N	8532271	8533270	5	90.5	63.7	68.3	40.7
chr19	C730002L08Rik	Upstream	N	20626821	20627820	5	60.1	27.7	62.8	35.2
chr4	Pnrc2	Upstream	N	135429762	135430761	3	42.6	26.9	50.9	23.5
chr13	Nup153	Upstream	N	46823219	46824218	2	50.9	5.5	32.2	4.9
chr2	Rnf114	Upstream	N	167317145	167318144	1	61.5	23.8	56.0	28.8
chr11	Sectm1b	Upstream	N	120924884	120925883	5	75.6	32.0	56.4	29.3
chr17	Mep1a	Upstream	N	43639723	43640722	5	24.2	0.0	27.1	0.0
chr16	Ephb3	Upstream	N	21203868	21204867	3	29.8	6.1	33.1	6.0
chr5	Gbp6	Upstream	N	105722719	105723718	5	54.0	3.0	26.9	0.0
chr12	Gm5434	Upstream	N	36815966	36816965	5	55.1	38.2	70.2	43.5
chr8	Tmem192	Upstream	N	67470084	67471083	3	46.4	26.2	63.2	36.8
chr1	Nuak2	Upstream	N	134211702	134212701	3	73.1	57.1	98.3	72.0
chr1	Sp100	Upstream	N	87545625	87546624	5	46.3	28.0	53.1	26.8
chr1	BC055402	Upstream	N	57271841	57272840	5	66.1	21.5	58.1	32.2
chr6	Tuba3a	Upstream	N	125236061	125237060	3	94.7	66.0	96.8	70.9
chr9	Snrk	Upstream	N	122025384	122026383	3	51.4	12.9	38.1	12.3
chr5	Cldn15	Upstream	N	137442739	137443738	5	18.9	1.6	25.6	0.0
chr8	Ces5a	Upstream	N	96059608	96060607	5	48.9	31.7	54.0	28.5
chr11	Cobl	Upstream	N	12364964	12365963	1	67.3	40.5	80.5	55.1
chr17	Apobec2	Upstream	N	48572054	48573053	3	51.7	34.4	56.3	31.0
chr11	Ddc	Upstream	N	11798148	11799147	5	68.7	47.4	78.9	53.6
chr8	Fam89a	Upstream	N	127275710	127276709	1	78.1	47.7	75.8	50.7
chr3	Alpk1	Upstream	N	127483446	127484445	4	90.1	45.5	89.1	64.1
chr18	Gm9926	Upstream	N	66662832	66663831	5	68.3	24.9	40.8	15.8
chr19	Capn1	Upstream	N	6015826	6016825	4	33.0	9.1	34.8	9.9
chr3	2010016I18Rik	Upstream	N	106288832	106289831	5	42.9	17.1	35.1	10.3
chr16	Gm10791	Upstream	N	84971456	84972455	5	60.0	36.5	70.8	46.0
chr8	Mtmr7	Upstream	N	41720147	41721146	3	54.5	0.0	25.6	1.0
chr14	Ctsb	Upstream	N	63740299	63741298	4	73.3	55.4	46.3	21.7
chr17	Slc9a3r2	Upstream	N	24787251	24788250	3	44.2	10.8	39.0	14.5

chr17	Btnl6	Upstream	N	34654298	34655297	5	90.0	66.7	84.6	60.3
chr4	AI427809	Upstream	N	53283105	53284104	5	41.7	8.7	72.7	48.5
chr11	Tcn2	Upstream	N	3832082	3833081	6	43.7	28.7	24.4	0.3
chr17	Crip3	Upstream	N	46564891	46565890	5	83.3	53.6	71.1	47.1
chr10	Sumo3	Upstream	N	77067979	77068978	1	86.4	63.4	92.3	68.3
chr12	Kidins220	Upstream	N	25658797	25659796	4	55.4	35.4	60.0	36.1
chr3	1700007F19Rik	Upstream	N	57967730	57968729	5	49.3	6.4	39.6	15.8
chr19	Naaladl1	Upstream	N	6104798	6105797	5	86.3	26.0	51.1	27.5
chr14	Pcca	Upstream	N	122932550	122933549	3	17.9	1.0	26.8	3.4
chr4	Prdx1	Upstream	N	116357204	116358203	4	80.2	44.6	43.3	20.3
chr11	Olfr332	Upstream	N	58306002	58307001	5	65.2	49.4	71.8	48.8
chr13	Zfp389	Upstream	N	21598394	21599393	4	68.0	31.0	50.0	27.1
chr12	Mir1193	Upstream	N	110952881	110953880	6	88.6	57.4	73.0	50.1
chr12	Mir679	Upstream	N	110952787	110953786	6	88.6	57.4	73.0	50.1
chr9	Olfr884	Upstream	N	37853809	37854808	5	88.4	47.2	90.2	67.7
chr15	Mroh1	Upstream	N	76209943	76210942	3	83.7	42.9	69.4	47.0
chr7	Scgb1b27	Upstream	N	34805586	34806585	5	93.3	75.1	90.9	68.6
chr16	Slc15a2	Upstream	N	36785049	36786048	5	60.1	28.5	61.0	38.8
chr19	Lrrn4cl	Upstream	N	8924275	8925274	5	67.0	50.6	52.4	30.2
chr1	A230020J21Rik	Upstream	N	192848230	192849229	2	40.5	17.1	45.3	23.2
chr11	Mir3064	Upstream	N	106644074	106645073	5	83.3	55.8	76.0	54.0
chr10	Mir1931	Upstream	N	92624530	92625529	3	41.7	25.3	44.5	22.6
chr9	Trak1	Upstream	N	121275076	121276075	5	60.9	40.6	61.9	40.1
chr12	Mir494	Upstream	N	110952528	110953527	5	88.6	38.1	75.8	54.0
chr8	Slc20a2	Upstream	N	23586172	23587171	2	44.5	17.2	51.4	29.7
chr15	Cyp11b1	Upstream	N	74672074	74673073	5	82.6	63.6	82.0	60.3
chr15	Apol7a	Upstream	N	77229541	77230540	5	51.6	7.8	29.9	8.3
chr14	Rft1	Upstream	N	31466561	31467560	4	91.7	58.7	81.5	59.8
chr11	Mir21	Upstream	N	86397661	86398660	5	23.3	2.7	21.6	0.0
chr16	Ppp1r2	Upstream	N	31275364	31276363	3	40.0	23.2	58.9	37.3
chr14	Pibf1	Upstream	N	99497652	99498651	4	57.5	36.7	57.5	35.9
chr11	Pik3ip1	Upstream	N	3229734	3230733	5	80.4	63.8	64.6	43.4
chr2	Defb29	Upstream	N	152365778	152366777	5	24.7	2.0	27.0	5.8
chr11	Tmem100	Upstream	N	89890662	89891661	5	53.9	31.2	51.2	30.2
chr19	Aldh1a1	Upstream	N	20675472	20676471	5	36.7	7.7	23.8	2.9
chr4	Serinc2	Upstream	N	129952831	129953830	3	69.7	44.8	73.7	53.0
chr2	Rbm45	Upstream	N	76207041	76208040	3	63.6	42.6	56.2	35.6
chr1	Slc26a9	Upstream	N	133639599	133640598	5	46.3	30.8	52.4	32.0
chr6	Mios	Upstream	N	8158227	8159226	3	73.7	57.3	72.4	52.1
chr8	Rasa3	Upstream	N	13677588	13678587	3	80.0	28.1	43.4	23.1
chr17	Rnf39	Upstream	N	37078996	37079995	2	53.7	28.3	51.0	30.7
chr1	Chit1	Upstream	N	136006819	136007818	5	46.6	6.5	34.8	14.7
chr14	Sftpd	Upstream	N	41998488	41999487	5	70.2	49.1	75.4	55.3
chr8	Nfat5	Upstream	N	109816370	109817369	2	58.1	35.3	74.5	54.5
chr7	Slc8a2	Upstream	N	16714649	16715648	3	19.5	2.9	22.6	2.7
chr9	Car12	Upstream	N	66560493	66561492	4	36.3	14.4	35.0	15.2
chr1	Pyhin1	Upstream	N	175559990	175560989	4	86.0	62.7	63.8	44.0
chr15	Csf2rb2	Upstream	N	78136034	78137033	5	76.7	41.8	57.9	38.1
chr7	Nsmce1	Upstream	N	132635057	132636056	2	55.4	14.3	44.2	24.5

chr7	Gramd1a	Upstream	N	31936070	31937069	1	55.4	40.2	51.6	31.9
chr11	Gm2a	Upstream	N	54910487	54911486	2	33.3	11.7	25.5	5.9
chr5	Cd38	Upstream	N	44259066	44260065	5	72.9	57.5	80.7	61.2
chr12	Mir453	Upstream	N	110972829	110973828	5	77.3	41.5	86.7	67.1
chr14	Itih1	Upstream	N	31756476	31757475	5	76.8	43.3	40.0	20.4
chr4	Snhg3	Upstream	N	131909602	131910601	4	73.5	45.3	71.1	51.5
chr11	Hoxb4	Upstream	N	96178581	96179580	3	57.1	33.1	54.9	35.3
chr4	Pde4b	Upstream	N	101926544	101927543	6	39.0	4.0	23.6	4.1
chr13	Slc35b3	Upstream	N	39052745	39053744	4	24.5	3.5	19.4	0.0
chr9	E030011O05Rik	Upstream	N	96653251	96654250	5	95.2	62.2	68.2	49.0
chr11	Iba57	Upstream	N	58977248	58978247	3	86.4	59.3	80.1	60.9
chr5	Ppargc1a	Upstream	N	51945161	51946160	5	48.2	27.7	41.2	22.0
chr10	Hddc2	Upstream	N	31032211	31033210	3	26.1	4.5	22.8	3.6
chr1	5033404E19Rik	Upstream	N	187320185	187321184	5	65.4	45.8	63.1	44.0
chr18	Gm4841	Upstream	N	60432922	60433921	5	85.3	51.4	62.3	43.2
chr12	Gpx2	Upstream	N	77896542	77897541	5	27.9	10.0	65.8	46.7
chr7	Sycn	Upstream	N	29324904	29325903	5	31.8	16.6	43.1	24.2
chr15	Pus7l	Upstream	N	94373939	94374938	1	59.7	24.1	50.5	31.8
chr16	Tctex1d2	Upstream	N	32418788	32419787	4	69.5	52.2	83.3	64.7
chr17	BC004004	Upstream	N	29404733	29405732	4	67.5	42.5	36.1	17.5
chr1	Kcnb2	Upstream	N	15301533	15302532	5	91.0	68.2	85.2	66.7
chr17	9830107B12Rik	Upstream	N	48283258	48284257	5	81.9	60.3	66.7	48.1
chr8	Mir1969	Upstream	N	73448424	73449423	5	89.5	69.7	84.6	66.1
chr10	Ccdc170	Upstream	N	5836670	5837669	5	53.8	20.3	48.2	29.7
chr17	Slc44a4	Upstream	N	35050411	35051410	5	60.7	9.5	52.8	34.3
chr12	Mir1936	Upstream	N	103923231	103924230	5	45.0	18.7	42.2	23.9
chr19	Fas	Upstream	N	34364149	34365148	4	44.4	14.6	40.8	22.5
chr1	Apcs	Upstream	N	174825186	174826185	5	78.5	59.5	67.1	48.9
chr5	Rabl5	Upstream	N	137383020	137384019	4	84.0	58.3	72.0	53.8
chr3	4930564D02Rik	Upstream	N	104868762	104869761	5	66.5	48.6	72.0	53.8
chr10	Plekhg1	Upstream	N	6606165	6607164	4	32.8	7.2	30.4	12.2
chr10	Chpt1	Upstream	N	87966716	87967715	2	39.0	15.4	43.9	25.7
chr5	1700027F09Rik	Upstream	N	64859182	64860181	5	68.0	44.2	62.6	44.5
chr6	Setmar	Upstream	N	108014039	108015038	5	37.7	19.7	21.0	2.9
chr12	Mir3070b	Upstream	N	110825802	110826801	5	80.5	53.0	75.8	57.8
chr11	Wdr81	Upstream	N	75268220	75269219	4	36.4	17.7	34.9	16.9
chr10	Avil	Upstream	N	126436765	126437764	5	87.5	62.8	73.7	55.8
chr11	Mir196a-1	Upstream	N	96125478	96126477	5	31.2	0.9	19.1	1.2
chr2	Nutm1	Upstream	N	112099449	112100448	5	66.7	49.0	69.9	52.0
chr7	Ap3b2	Upstream	N	88638812	88639811	4	74.5	55.7	86.7	69.1
chr13	Rfesd	Upstream	N	76156161	76157160	4	68.0	43.6	81.9	64.3
chr11	Krt17	Upstream	N	100122304	100123303	5	70.3	51.3	68.3	50.7
chr11	Gm12298	Upstream	N	66760589	66761588	5	59.2	7.1	58.3	40.7
chr11	2210015D19Rik	Upstream	N	5661153	5662152	3	31.7	13.6	31.2	13.7
chr10	Col6a1	Upstream	N	76188790	76189789	5	95.8	32.1	50.5	32.9
chr14	Prss52	Upstream	N	64722160	64723159	5	82.0	63.0	81.6	64.1
chr17	Gm9705	Upstream	N	32757264	32758263	5	69.9	40.1	76.9	59.5
chr6	Gm8579	Upstream	N	3237519	3238518	5	73.9	49.4	76.0	58.7
chr2	Upp2	Upstream	N	58606596	58607595	5	62.2	32.8	43.9	26.5

chr2	Srxn1	Upstream	N	151930260	151931259	2	51.4	16.7	32.4	15.0
chr12	Pacs2	Upstream	N	114251719	114252718	1	48.0	25.4	53.3	36.1
chr8	Snx20	Upstream	N	91160028	91161027	5	73.2	51.8	78.8	61.6
chr5	Hspb8	Upstream	N	116872874	116873873	5	37.2	18.2	23.9	6.8
chr16	Chodl	Upstream	N	78930193	78931192	4	64.2	45.8	51.3	34.3
chr4	Mir200a	Upstream	N	155429095	155430094	6	24.1	1.0	17.4	0.4
chr5	Gsap	Upstream	N	20691085	20692084	3	61.7	40.4	60.4	43.5
chr10	Nedd1	Upstream	N	92185164	92186163	3	64.6	29.2	40.7	23.9
chr11	Purb	Upstream	N	6376080	6377079	3	38.3	1.9	55.8	39.0
chr18	Zfp474	Upstream	N	52774569	52775568	5	81.6	50.6	72.3	55.6
chr7	Trim21	Upstream	N	109713984	109714983	5	62.4	47.1	61.5	44.8
chr7	Fut1	Upstream	N	52871976	52872975	2	52.4	32.3	50.7	34.2
chr11	Pex12	Upstream	N	83112480	83113479	3	83.5	66.7	65.3	48.8
chr2	AU019990	Upstream	N	132422932	132423931	5	87.4	71.7	91.1	74.5
chr2	Lmo2	Upstream	N	103797152	103798151	5	75.4	57.7	62.2	45.7
chr10	Rdh1	Upstream	N	127195819	127196818	5	32.3	15.6	31.9	15.5
chr19	Tut1	Upstream	N	9027340	9028339	3	58.6	43.0	60.0	43.7
chr11	Snord1a	Upstream	N	116534911	116535910	5	47.2	26.6	48.8	32.5
chr11	Alox8	Upstream	N	69011346	69012345	5	75.9	49.9	59.8	43.6
chr19	Dagla	Upstream	N	10379368	10380367	1	53.2	32.6	56.5	40.3
chr13	Wdr41	Upstream	N	95745299	95746298	3	31.0	6.3	17.6	1.5
chr12	Sdc1	Upstream	N	8777202	8778201	2	65.1	46.1	60.5	44.5
chr18	Atp5a1	Upstream	N	78011507	78012506	4	90.5	68.7	76.0	60.0
chr18	Dsc2	Upstream	N	20218007	20219006	5	26.2	8.8	17.8	1.8
chr9	Cdcp1	Upstream	N	123125157	123126156	4	64.7	24.2	64.8	49.0
chr15	1700011A15Rik	Upstream	N	101277176	101278175	5	70.8	51.5	69.2	53.5
chr11	Gm16515	Upstream	N	60727295	60728294	2	40.7	23.1	41.5	25.8
chr13	Vmn1r202	Upstream	N	22594115	22595114	5	83.8	64.2	83.3	67.6
chr7	Prss8	Upstream	N	135073628	135074627	5	22.4	3.2	19.6	3.9
chr2	Psd4	Upstream	N	24239917	24240916	5	23.4	6.4	23.4	7.8
chr18	Dnajc18	Upstream	N	35862799	35863798	3	76.9	55.2	66.1	50.5
chr4	Gm13037	Upstream	N	141537907	141538906	5	36.6	16.8	32.1	16.5
chr2	Sardh	Upstream	N	27102824	27103823	5	25.0	2.2	37.6	22.1
chr17	Gm8363	Upstream	N	5483070	5484069	5	32.0	5.3	26.9	11.4
chr17	Sult1c2	Upstream	N	53985284	53986283	5	63.6	20.4	31.1	15.7
chr7	Sec11a	Upstream	N	88092437	88093436	2	16.7	0.0	15.4	0.0
chr4	Tnfrsf18	Upstream	N	155399451	155400450	5	66.8	49.1	61.8	46.5
chr5	Plb1	Upstream	N	32654953	32655952	5	81.3	59.8	83.5	68.1
chr4	Luzp1	Upstream	N	136024676	136025675	3	21.3	6.1	19.1	3.8
chr5	Lrrc17	Upstream	N	21048345	21049344	5	92.6	34.0	60.6	45.3
chr8	Spcs3	Upstream	N	55615352	55616351	3	53.6	27.6	41.9	26.8
chr11	Hoxb5	Upstream	N	96163826	96164825	2	73.4	38.4	57.0	41.9
chr12	Zfp410	Upstream	N	85656809	85657808	3	39.2	16.7	36.4	21.3
chr10	Utrn	Upstream	N	12581534	12582533	5	77.9	42.4	59.3	44.3

Group 8-mDMR genes associated with methylation loss at gene body and 3' non-CGI

							DNA Methylation Level (%)			
							ISCs		Diff. Cells	
Chrom	Gene	Region	CGI	Start	End	#Bins	P0	P21	P0	P21

chr11	4933402P03Rik	3-UTR	N	69630068	69630127	1	49.3	0.0	59.4	1.6
chr5	Sel1l3	3-UTR	N	53498322	53499273	5	80.0	22.9	82.9	29.2
chr4	AI464131	3-UTR	N	41442634	41444510	8	87.0	37.7	89.7	37.2
chr3	4930425O10Rik	3-UTR	N	138374499	138375636	6	88.9	64.6	87.3	36.5
chr3	Arl14	3-UTR	N	69027023	69027540	3	71.2	16.5	75.7	25.1
chr17	Fsd1	3-UTR	N	56136176	56136304	2	88.9	45.8	89.7	40.6
chr1	Cnot11	3-UTR	N	39602112	39603722	8	92.0	46.0	70.2	22.4
chr17	Wdr90	3-UTR	N	25981679	25982012	3	55.1	17.2	66.3	18.7
chr2	Trib3	3-UTR	N	152163161	152163942	4	40.1	9.4	51.6	5.3
chr9	Cxcr5	3-UTR	N	44319870	44321316	7	77.6	39.7	77.2	31.9
chr3	Trim46	3-UTR	N	89038099	89038820	4	58.4	33.2	68.5	23.7
chr11	1700003D09Rik	3-UTR	N	98212032	98213308	6	57.4	9.9	52.1	9.1
chr1	Lefty2	3-UTR	N	182827945	182829233	6	62.5	25.6	61.7	19.9
chr10	Socs2	3-UTR	N	94874124	94875286	6	74.0	29.0	63.8	22.7
chr10	Txlnb	3-UTR	N	17563287	17565469	10	67.1	48.8	63.6	23.5
chr19	Tmem223	3-UTR	N	8846831	8846965	2	78.6	27.5	75.0	35.2
chr8	Cul4a	3-UTR	N	13146628	13147939	6	59.3	16.5	60.8	21.3
chr11	Usp43	3-UTR	N	67668025	67668982	5	98.1	73.5	76.2	36.7
chr15	Krt8	3-UTR	N	101827142	101827370	1	39.4	19.4	54.2	15.5
chr12	Kcns3	3-UTR	N	11097008	11098026	5	95.7	77.1	100.0	61.9
chr4	D830031N03Rik	3-UTR	N	123080844	123084602	19	82.5	29.6	62.9	25.0
chr4	Mpl	3-UTR	N	118115022	118116033	5	60.5	39.5	65.0	27.1
chr8	Hook2	3-UTR	N	87526911	87527263	2	91.4	70.4	77.8	40.0
chr11	Sap30bp	3-UTR	N	115825707	115826848	6	67.3	30.0	57.6	20.8
chr15	Myc	3-UTR	N	61821464	61821916	2	74.6	27.8	79.4	42.9
chr14	Fam160b2	3-UTR	N	70983102	70984851	8	86.4	36.5	84.5	48.3
chr7	Cars	3-UTR	N	150743135	150743776	3	97.1	79.3	67.7	31.6
chr1	Tnni1	3-UTR	N	137706311	137707566	6	50.6	9.5	56.3	20.5
chr17	Cdsn	3-UTR	N	35693207	35694125	5	47.7	10.1	44.9	9.3
chr3	Tifa	3-UTR	N	127500055	127501307	6	77.1	8.9	78.7	43.3
chr17	Fam195a	3-UTR	N	26000643	26000913	1	45.7	28.8	56.4	21.3
chr6	1700101I11Rik	3-UTR	N	129482202	129482960	5	60.4	29.6	40.1	5.2
chr7	Slx1b	3-UTR	N	133832441	133835139	14	83.5	63.2	86.8	52.1
chr4	Hcrtr1	3-UTR	N	129807461	129808084	3	66.6	19.6	57.4	23.1
chr12	Arl4a	3-UTR	N	40759878	40762730	12	44.8	9.2	33.9	0.0
chr19	Ifit3	3-UTR	N	34662758	34663472	4	87.0	32.0	74.5	40.6
chr17	Wdr46	3-UTR	N	34086361	34086640	2	51.3	19.4	52.4	18.9
chr11	Ramp3	3-UTR	N	6576742	6577478	4	50.1	4.9	63.1	30.3
chr6	2310001H17Rik	3-UTR	N	129182641	129182965	2	20.7	4.8	35.7	2.9
chr12	Cfl2	3-UTR	N	55959806	55962111	12	70.5	44.2	90.3	57.8
chr1	Pm20d1	3-UTR	N	133712688	133714692	10	76.3	53.8	83.6	51.1
chr17	Ntn3	3-UTR	N	24340791	24343542	5	53.7	34.0	60.9	28.6
chr11	Rasd1	3-UTR	N	59776685	59777311	2	68.9	36.1	59.9	27.6
chr9	Bco2	3-UTR	N	50341192	50341510	2	21.1	0.0	46.0	14.1
chr3	Impa1	3-UTR	N	10313540	10315185	8	82.9	0.7	76.6	44.8
chr9	Oxsr1	3-UTR	N	119147551	119150259	14	67.1	34.2	77.9	46.5
chr5	5830416I19Rik	3-UTR	N	64438901	64441465	13	51.9	33.4	62.5	31.8
chr4	Tspan1	3-UTR	N	115834486	115835592	6	77.4	30.2	76.2	45.6
chr17	Tbl3	3-UTR	N	24837598	24837617	1	76.8	55.8	72.3	42.5

chr9	Ccr5	3-UTR	N	124040166	124041922	9	33.8	18.0	46.5	16.8
chr3	Dclre1b	3-UTR	N	103604528	103606890	13	61.1	3.0	60.3	30.7
chr7	Pwwp2b	3-UTR	N	146452385	146453152	4	41.1	22.1	57.5	27.9
chr6	B230319C09Rik	3-UTR	N	83397070	83398316	6	95.7	69.1	94.1	64.7
chr11	Hoxb4	3-UTR	N	96181643	96182952	4	44.7	28.0	68.1	38.9
chr18	Atg12	3-UTR	N	46892071	46894103	10	49.7	34.1	58.8	29.9
chr9	Rasgrf1	3-UTR	N	89921602	89921817	1	46.0	23.0	57.2	29.4
chr17	Gpank1	3-UTR	N	35261610	35261760	1	36.6	2.2	34.6	7.2
chr7	Ifitm3	3-UTR	N	148195489	148195623	1	77.7	53.2	82.1	54.8
chr11	Eif1	3-UTR	N	100182676	100183410	4	42.5	4.1	36.4	9.2
chr17	Ehmt2	3-UTR	N	35050766	35050992	2	29.2	9.5	58.7	31.5
chr9	Cox5a	3-UTR	N	57379590	57380233	3	18.0	0.0	44.8	18.1
chr7	Ppfibp2	3-UTR	N	114891112	114892097	6	81.3	55.2	75.4	49.0
chr14	Lrrc18	3-UTR	N	33827036	33828478	6	58.2	22.8	62.6	36.3
chr1	Kcne4	3-UTR	N	78814725	78816600	9	39.2	13.4	55.2	29.2
chr2	4930500J02Rik	3-UTR	N	104411352	104411586	1	19.1	0.0	27.4	1.4
chr6	Clec9a	3-UTR	N	129372450	129374782	12	57.0	11.7	43.7	17.9
chr14	Atg14	3-UTR	N	48160568	48162511	10	73.5	44.5	77.4	51.6
chr17	Noxo1	3-UTR	N	24837301	24837474	2	20.0	1.8	26.6	1.0
chr4	2410114N07Rik	3-UTR	N	34857038	34857857	4	78.4	62.3	81.2	56.0
chr6	Usp5	3-UTR	N	124765037	124765583	3	34.1	1.9	41.2	16.2
chr4	Slc44a1	3-UTR	N	53634482	53635350	4	89.8	33.5	65.5	41.1
chr6	Gadd45a	3-UTR	N	66985090	66985652	4	35.1	6.0	33.9	9.6
chr15	Ccdc134	3-UTR	N	81971422	81972632	6	75.3	53.4	82.0	58.4
chr8	Defa-ps12	3-UTR	N	19212659	19212760	1	78.6	50.0	70.4	47.4
chr15	Zfp572	3-UTR	N	59142493	59143568	5	96.8	67.2	89.0	66.4
chr12	Gpx2	3-UTR	N	77893322	77893636	2	91.3	73.0	93.0	70.5
chr9	4833428L15Rik	3-UTR	N	45224709	45225478	4	63.7	44.0	71.2	49.0
chr2	Ada	3-UTR	N	163552307	163553295	5	47.2	2.0	43.7	21.6
chr1	Gls	3-UTR	N	52220294	52223001	14	100.0	70.6	91.0	69.2
chr1	D630023F18Rik	3-UTR	N	65153887	65155270	7	97.0	80.0	93.8	72.2
chr19	Pitpnm1	3-UTR	N	4113485	4113965	3	32.0	0.0	30.8	9.4
chr6	H2afj	3-UTR	N	136757251	136758595	3	42.4	12.5	26.7	5.5
chr9	AI593442	3-UTR	N	52481147	52485780	19	38.4	11.9	48.1	26.9
chr11	4933422H20Rik	3-UTR	N	115309535	115309582	2	74.3	18.8	59.6	38.4
chr5	Htt	3-UTR	N	35251495	35255170	18	90.7	58.0	79.2	58.2
chr17	Gm16197	3-UTR	N	29194419	29197303	14	87.6	59.2	83.4	62.6
chr4	Hrct1	3-UTR	N	43740564	43740982	2	75.6	60.4	68.9	48.2
chr7	E230029C05Rik	3-UTR	N	97186459	97188790	12	79.2	57.1	80.2	59.6
chr11	Aatf	3-UTR	N	84236358	84236457	1	31.8	6.7	28.3	8.0
chr1	A130010J15Rik	3-UTR	N	195001328	195004026	14	85.3	66.3	74.2	54.1
chr5	3110082I17Rik	3-UTR	N	139835693	139836498	4	53.3	34.8	48.9	29.0
chr10	Rps26	3-UTR	N	128061585	128061656	1	93.3	76.6	97.9	78.1
chr13	Dmgdh	3-UTR	N	94522421	94522778	2	23.1	2.4	54.4	34.7
chr2	F830045P16Rik	3-UTR	N	129284095	129285028	5	85.0	28.6	86.3	66.7
chr3	Mme	3-UTR	N	63184255	63186153	9	76.5	45.7	76.8	57.5
chr4	Gpatch3	3-UTR	N	133139748	133140157	1	33.3	15.0	30.8	11.8
chr2	Pmpca	3-UTR	N	26251109	26252641	8	96.7	37.0	75.4	56.7
chr15	Rpl37	3-UTR	N	5068637	5069140	4	87.0	64.0	77.1	58.8

chr9	Kank2	3-UTR	N	21571217	21573394	11	72.6	56.4	68.2	49.9
chr2	Astl	3-UTR	N	127182511	127183387	4	88.3	43.2	70.8	52.6
chr13	2310005E17Rik	3-UTR	N	99194065	99194871	4	20.2	2.9	21.4	3.3
chr4	Gnb1	3-UTR	N	154931387	154933378	10	73.6	55.5	76.2	58.1
chr11	Map3k14	3-UTR	N	103081078	103082338	6	75.2	50.6	67.0	49.0
chr9	Msl2	3-UTR	N	101004493	101007129	13	83.8	48.2	71.6	53.7
chr11	5730522E02Rik	3-UTR	N	25516846	25517872	5	94.7	76.4	94.9	77.3
chr10	Hs3st5	3-UTR	N	36553318	36554200	4	100.0	67.2	93.2	75.6
chr2	Inpp5e	3-UTR	N	26251769	26253358	8	89.7	46.0	73.3	56.0
chr16	Hmgn1	3-UTR	N	96343195	96343982	4	89.2	69.2	88.8	71.6
chr3	Dennd2d	3-UTR	N	106303525	106305948	13	91.0	74.9	87.2	70.2
chr14	Adam28	3-UTR	N	69223055	69224631	8	87.2	61.6	77.9	60.8
chr7	Crebzf	3-UTR	N	97592602	97596553	18	59.1	24.8	40.7	24.0
chr7	B230206H07Rik	3-UTR	N	148545077	148547935	12	86.6	64.6	92.9	76.1
chr9	1700104A03Rik	3-UTR	N	54116387	54117050	3	96.2	77.1	80.8	64.0
chr7	Pycard	3-UTR	N	135134887	135136225	7	65.2	37.2	45.8	29.1
chr15	Trmt12	3-UTR	N	58705651	58708336	13	86.0	63.8	81.6	65.0
chr14	Mdp1	3-UTR	N	56276716	56277819	6	39.7	23.6	37.5	21.2
chr5	Igfbp7	3-UTR	N	77778265	77778504	2	65.3	42.9	76.5	60.4
chr14	Slc39a2	3-UTR	N	52515207	52516420	6	36.7	13.9	40.2	24.2
chr16	1810007I06Rik	3-UTR	N	13739404	13739564	1	34.0	15.4	29.6	13.6
chr8	Slc18a1	3-UTR	N	71561607	71562693	5	35.0	14.1	40.7	24.8
chr12	2410004P03Rik	3-UTR	N	17011764	17012621	5	92.1	71.1	94.4	78.6
chr4	E130308A19Rik	3-UTR	N	59765912	59767175	6	92.1	53.1	84.4	68.6
chr8	Retn	3-UTR	N	3657387	3659818	13	64.8	41.1	73.6	57.9
chr11	Hoxb5	3-UTR	N	96166452	96167435	4	78.6	46.7	52.7	37.0
chr4	Uts2	3-UTR	N	150375790	150375919	1	77.2	56.1	75.9	60.2
chr4	Mxra8	3-UTR	N	155217440	155218211	4	87.7	48.4	83.5	67.9
chr6	Ube2h	3-UTR	N	30161289	30164958	18	75.8	44.5	74.9	59.7
chr4	Gm13032	3-UTR	N	140372611	140373426	4	84.0	47.4	72.5	57.3
chr3	Setd7	3-UTR	N	51319240	51325228	30	63.1	40.6	55.7	40.6
chr13	F2rl2	3-UTR	N	96471514	96472723	6	63.4	36.6	74.3	59.3
chr11	Sun3	Downstreai	N	8915057	8916056	5	95.2	69.5	89.3	20.3
chr9	Gm10684	Downstreai	N	44914259	44915258	5	94.4	5.0	87.0	26.1
chr4	Zc3h12a	Downstreai	N	124794658	124795657	5	68.0	7.8	69.6	10.4
chr16	Mir3081	Downstreai	N	44557159	44558158	3	28.1	2.1	55.7	0.7
chr9	Gm19299	Downstreai	N	67011596	67012595	5	61.7	4.1	73.2	18.8
chr2	Pkig	Downstreai	N	163551895	163552894	6	41.7	2.6	56.7	2.7
chr17	Stap2	Downstreai	N	56135499	56136498	5	88.9	16.5	67.5	14.6
chr3	4933431E20Rik	Downstreai	N	107690768	107691767	5	65.1	44.2	81.4	28.6
chr9	Cmtm8	Downstreai	N	114697463	114698462	5	58.1	12.5	78.8	26.5
chr13	4930401O12Rik	Downstreai	N	31304279	31305278	5	88.0	53.3	77.1	25.0
chr14	Scel	Downstreai	N	104012564	104013563	3	97.6	61.6	100.0	48.0
chr8	Med26	Downstreai	N	75017458	75018457	6	76.6	46.4	54.5	3.7
chr6	Hoxa4	Downstreai	N	52138686	52139685	5	55.6	14.7	59.3	10.0
chr5	Pisd	Downstreai	N	33077962	33078961	5	43.3	20.3	65.2	17.5
chr6	Ptpro	Downstreai	N	137413155	137414154	5	68.2	15.4	60.9	13.9
chr2	Adamtsl2	Downstreai	N	26964134	26965133	5	62.4	25.7	63.8	17.0
chr9	Fam81a	Downstreai	N	69936117	69937116	5	70.7	46.7	53.6	7.5

chr17	Pgp	Downstrea	N	24608542	24609541	5	44.1	6.4	52.9	7.6
chr18	Ablim3	Downstrea	N	61958047	61959046	5	63.4	13.9	78.6	34.0
chr13	Gm16907	Downstrea	N	63389462	63390461	5	71.0	45.4	79.1	34.7
chr12	F730043M19Rik	Downstrea	N	33795576	33796575	5	56.1	17.7	67.8	24.4
chr7	Syne4	Downstrea	N	31104065	31105064	5	36.4	2.2	48.9	7.1
chr14	Pnp	Downstrea	N	51573088	51574087	5	59.6	15.0	61.7	21.1
chr10	9530003J23Rik	Downstrea	N	116669809	116670808	5	57.9	32.4	69.8	29.3
chr7	Clrn3	Downstrea	N	142702139	142703138	5	72.2	41.3	63.8	23.4
chr1	Dyrk3	Downstrea	N	133024018	133025017	5	71.7	41.2	75.1	34.9
chr11	Hoxb7	Downstrea	N	96151478	96152477	5	62.9	23.4	64.9	24.7
chr1	2900060B14Rik	Downstrea	N	120354246	120355245	5	72.2	54.2	74.4	34.2
chr17	B3galt4	Downstrea	N	34085857	34086856	5	39.8	12.9	51.3	11.3
chr7	Ppp6r1	Downstrea	N	4582097	4583096	6	65.6	1.7	48.8	9.7
chr15	Xpnpep3	Downstrea	N	81285319	81286318	5	75.3	38.9	70.9	32.2
chr16	Gm10818	Downstrea	N	32664309	32665308	5	90.0	74.2	97.0	58.7
chr18	Gm6277	Downstrea	N	11978597	11979596	5	70.0	0.0	84.5	46.4
chr3	Intu	Downstrea	N	40508697	40509696	5	63.5	29.8	72.2	34.3
chr2	Tbc1d20	Downstrea	N	152138327	152139326	5	53.0	26.2	59.3	21.5
chr8	Clec4g	Downstrea	N	3715071	3716070	5	86.7	63.9	83.3	45.8
chr4	Glipr2	Downstrea	N	43991991	43992990	5	81.4	36.7	67.4	30.2
chr16	Lztr1	Downstrea	N	17526424	17527423	5	100.0	57.5	86.4	49.8
chr7	Unc45a	Downstrea	N	87469179	87470178	4	74.7	53.2	70.6	34.1
chr11	Gm11944	Downstrea	N	3220726	3221725	5	49.5	17.2	54.7	19.1
chr16	BC016579	Downstrea	N	45625961	45626960	5	97.0	48.0	62.8	27.2
chr9	Prss45	Downstrea	N	110743815	110744814	5	72.2	42.6	59.7	24.5
chr2	Itih2	Downstrea	N	10015218	10016217	5	41.8	3.1	34.4	0.0
chr13	Pdlim7	Downstrea	N	55597848	55598847	5	44.4	1.5	67.2	33.0
chr7	Dmwd	Downstrea	N	19668125	19669124	6	59.7	35.3	57.7	24.0
chr14	Mrps16	Downstrea	N	21209453	21210452	5	79.2	14.4	68.4	35.1
chr6	Prh1	Downstrea	N	132522420	132523419	5	90.3	67.8	95.5	62.3
chr14	Ncoa4	Downstrea	N	32992550	32993549	5	67.5	38.3	52.7	20.0
chr14	Timm23	Downstrea	N	32992352	32993351	4	67.5	38.3	52.7	20.0
chr14	Dlgap5	Downstrea	N	48006454	48007453	5	67.3	42.4	61.4	28.9
chr9	Tcaim	Downstrea	N	122745451	122746450	5	30.3	3.8	39.6	7.2
chr14	Klf5	Downstrea	N	99712629	99713628	5	75.1	35.3	67.8	35.4
chr11	Med9	Downstrea	N	59776809	59777808	1	68.9	36.1	59.9	27.6
chr19	Rela	Downstrea	N	5648131	5649130	3	66.1	45.8	67.3	35.2
chr18	Ptpn2	Downstrea	N	67824155	67825154	5	86.4	69.3	90.4	58.3
chr5	Pom121	Downstrea	N	135851010	135852009	5	83.0	53.3	78.5	46.7
chr6	Slc6a12	Downstrea	N	121315792	121316791	5	71.8	39.2	44.2	12.5
chr15	Slc52a2	Downstrea	N	76372561	76373560	5	69.9	27.8	56.0	24.6
chr3	Arl14	Downstrea	N	69027541	69028540	5	72.2	37.2	65.5	34.2
chr1	A130010J15Rik	Downstrea	N	195004027	195005026	5	70.3	51.0	55.5	24.8
chr3	Naa15	Downstrea	N	51279908	51280907	5	97.1	46.0	95.2	64.8
chr13	Jarid2	Downstrea	N	45017013	45018012	6	64.1	41.8	81.0	50.9
chr17	Sox8	Downstrea	N	25701838	25702837	5	55.1	38.3	65.1	35.2
chr17	Mtch1	Downstrea	N	29468021	29469020	5	81.0	49.4	56.2	26.9
chr11	Snord96a	Downstrea	N	48615612	48616611	6	33.8	12.1	51.7	22.5
chr11	Mrpl38	Downstrea	N	115992131	115993130	3	79.4	48.4	34.6	5.6

chr5	Rbm33	Downstrear	N	28745783	28746782	5	61.5	33.8	55.3	26.3
chr18	Elac1	Downstrear	N	73893692	73894691	5	68.3	14.9	54.9	26.3
chr11	Kat2a	Downstrear	N	100565060	100566059	6	60.7	10.0	66.0	37.7
chr2	Gdf5	Downstrear	N	155765761	155766760	5	84.8	55.3	75.7	47.4
chr17	Wtap	Downstrear	N	13162886	13163885	5	76.9	39.7	65.5	37.2
chr8	Exosc6	Downstrear	N	113581565	113582564	5	71.7	50.8	78.8	50.6
chr8	Klkb1	Downstrear	N	46353807	46354806	5	90.9	63.3	85.9	57.9
chr2	D330050G23Rik	Downstrear	N	116738528	116739527	5	68.0	43.7	73.1	45.1
chr1	Sctr	Downstrear	N	121960110	121961109	5	79.8	41.9	72.0	44.2
chr3	Ccdc169	Downstrear	N	54976858	54977857	5	51.2	29.6	69.9	42.1
chr9	Nr2e3	Downstrear	N	59789578	59790577	5	51.1	18.1	47.7	19.9
chr4	Mir200b	Downstrear	N	155428790	155429789	6	24.1	5.8	28.1	0.4
chr19	Nmrk1	Downstrear	N	18726675	18727674	4	85.4	45.6	76.9	49.2
chr8	Clgn	Downstrear	N	85950729	85951728	5	35.5	3.8	39.5	11.8
chr3	Efna4	Downstrear	N	89136315	89137314	5	50.5	8.7	33.0	5.5
chr4	Mllt3	Downstrear	N	87414829	87415828	5	66.1	48.0	57.8	30.4
chr7	Sephs2	Downstrear	N	134414395	134415394	5	75.3	33.5	60.7	33.7
chr3	Phgdh	Downstrear	N	98116094	98117093	5	49.3	26.9	41.1	14.0
chr18	Atg12	Downstrear	N	46891071	46892070	5	54.1	36.7	88.4	61.7
chr4	Casz1	Downstrear	N	148329002	148330001	5	70.5	49.2	68.8	42.1
chr4	Gnb1	Downstrear	N	154933379	154934378	5	58.7	22.8	61.9	35.3
chr18	Fbxo15	Downstrear	N	85150785	85151784	5	82.3	54.4	70.1	43.6
chr13	Larp4b	Downstrear	N	9172337	9173336	5	84.5	64.1	83.1	56.6
chr1	3110035E14Rik	Downstrear	N	9617224	9618223	5	62.3	42.9	35.9	9.5
chr8	Trhr2	Downstrear	N	124879867	124880866	5	73.2	50.3	63.4	37.0
chr11	Rnf167	Downstrear	N	70464917	70465916	6	27.2	10.1	34.0	7.9
chr2	Alx4	Downstrear	N	93521497	93522496	5	58.2	33.5	53.2	27.3
chr2	Gm14207	Downstrear	N	119145935	119146934	5	82.2	61.9	79.1	53.2
chr15	Ccdc134	Downstrear	N	81972633	81973632	5	61.1	37.0	62.1	36.4
chr6	Parp11	Downstrear	N	127444258	127445257	5	44.5	7.2	34.8	9.4
chr7	Rcn3	Downstrear	N	52337284	52338283	4	51.1	26.3	50.1	24.8
chr19	1700054A03Rik	Downstrear	N	53158883	53159882	5	68.2	47.9	69.7	44.7
chr12	Fam161b	Downstrear	N	85685267	85686266	6	85.5	70.1	95.2	70.6
chr12	Zfp410	Downstrear	N	85685390	85686389	5	85.5	70.1	95.2	70.6
chr11	Uqcr10	Downstrear	N	4600971	4601970	5	51.7	27.2	50.2	25.6
chr2	Manbal	Downstrear	N	157222500	157223499	5	61.9	2.8	55.9	31.5
chr4	Aldh1b1	Downstrear	N	45817481	45818480	5	85.8	68.5	82.8	58.4
chr12	Exoc3l4	Downstrear	N	112669395	112670394	5	64.5	26.5	38.1	13.8
chr10	Prf1	Downstrear	N	60767012	60768011	5	75.8	53.2	71.3	47.0
chr11	Gm53	Downstrear	N	96125799	96126798	6	31.2	0.7	27.1	2.8
chr6	Gm15612	Downstrear	N	88797269	88798268	5	85.7	67.9	89.4	65.2
chr12	Mir668	Downstrear	N	110973008	110974007	5	70.9	55.1	78.7	54.5
chr1	Elf3	Downstrear	N	137149151	137150150	5	36.9	6.8	28.6	4.5
chr11	Sectm1b	Downstrear	N	120913737	120914736	5	71.0	51.1	74.6	50.6
chr5	Polr2b	Downstrear	N	77778354	77779353	5	65.3	42.9	84.2	60.4
chr7	Zfp566	Downstrear	N	30861356	30862355	5	65.3	42.1	66.5	43.1
chr15	D930007P13Rik	Downstrear	N	102952501	102953500	5	59.6	43.4	50.2	26.8
chr4	Gem	Downstrear	N	11642141	11643140	5	71.0	11.5	66.0	42.7
chr4	S100pbp	Downstrear	N	128827069	128828068	5	56.8	36.0	61.1	37.8

chr5	Piwil1	Downstrear	N	129261350	129262349	5	81.3	12.9	50.9	27.6
chr10	Cnksr3	Downstrear	N	3227480	3228479	5	87.6	53.5	75.7	52.5
chr2	Tmem230	Downstrear	N	132064228	132065227	5	40.1	18.3	43.6	20.5
chr13	2310005E17Rik	Downstrear	N	99193065	99194064	5	89.1	43.0	63.5	40.4
chr17	Hspa1l	Downstrear	N	35116174	35117173	5	87.7	55.6	77.3	54.4
chr10	Tbc1d30	Downstrear	N	120699876	120700875	5	66.0	40.0	63.0	40.1
chr5	Pms2	Downstrear	N	144692626	144693625	5	50.0	11.8	28.6	5.7
chr12	Mir3070a	Downstrear	N	110826242	110827241	6	80.5	53.0	83.6	60.7
chr2	Vps18	Downstrear	N	119124190	119125189	5	76.8	61.2	69.0	46.2
chr7	Trappc6a	Downstrear	N	20101495	20102494	5	47.1	28.4	63.6	40.9
chr2	4921530L18Rik	Downstrear	N	13990960	13991959	5	54.9	7.0	22.7	0.0
chr19	Gm9895	Downstrear	N	29143994	29144993	5	30.5	0.0	25.8	3.1
chr7	Ccnd1	Downstrear	N	152114836	152115835	5	57.5	28.2	54.7	32.1
chr1	Neurl3	Downstrear	N	36320447	36321446	5	30.4	9.0	49.3	26.7
chr7	Spib	Downstrear	N	51780365	51781364	4	35.2	12.4	33.7	11.2
chr5	Limch1	Downstrear	N	67448399	67449398	5	90.0	70.0	75.5	53.1
chr2	Acvr1	Downstrear	N	58297849	58298848	5	68.7	46.5	61.6	39.3
chr14	Timm8a2	Downstrear	N	122437645	122438644	5	84.5	36.1	78.1	56.0
chr12	Rab15	Downstrear	N	77897950	77898949	5	24.3	5.4	25.4	3.3
chr2	Foxs1	Downstrear	N	152756634	152757633	5	74.9	47.3	63.5	41.6
chr5	Zcchc8	Downstrear	N	124147311	124148310	5	56.9	29.6	54.9	32.9
chr11	Erbb2	Downstrear	N	98299031	98300030	3	50.7	29.1	41.6	19.8
chr1	Traf3ip3	Downstrear	N	195000698	195001697	5	78.7	55.1	78.6	56.9
chr1	1700019A02Rik	Downstrear	N	53214421	53215420	5	65.4	47.3	70.7	49.0
chr6	Ogg1	Downstrear	N	113284181	113285180	5	67.3	47.7	68.8	47.3
chr6	H2afj	Downstrear	N	136758596	136759595	5	30.5	11.5	39.4	17.9
chr19	Tmem179b	Downstrear	N	8846012	8847011	5	86.0	13.8	77.5	56.2
chr4	AI464131	Downstrear	N	41441634	41442633	5	86.6	46.5	71.3	50.0
chr11	Alkbh5	Downstrear	N	60372015	60373014	5	52.0	10.7	28.1	6.9
chr19	Fam178a	Downstrear	N	45058278	45059277	6	67.9	51.7	84.2	63.1
chr11	Serpinf2	Downstrear	N	75244238	75245237	5	91.4	74.0	87.9	66.9
chr16	Gsk3b	Downstrear	N	38246166	38247165	5	47.3	28.6	51.0	30.0
chr12	Mir345	Downstrear	N	110075279	110076278	5	36.3	14.5	42.0	21.0
chr11	4933422H20Rik	Downstrear	N	115309583	115310582	5	67.8	22.8	54.0	33.2
chr12	Tspan13	Downstrear	N	36740142	36741141	5	51.7	32.5	62.9	42.1
chr1	Lefty2	Downstrear	N	182829234	182830233	5	65.7	46.6	72.1	51.5
chr1	Syt14	Downstrear	N	194722501	194723500	5	96.0	80.6	87.8	67.5
chr13	Nr2f1	Downstrear	N	78327236	78328235	5	71.4	52.0	57.3	37.1
chr11	Rnf112	Downstrear	N	61260944	61261943	5	65.0	44.2	72.4	52.2
chr10	Irak3	Downstrear	N	119577710	119578709	5	74.1	58.8	67.6	47.4
chr1	Ugt1a10	Downstrear	N	90116578	90117577	5	38.0	9.1	34.3	14.3
chr1	Ugt1a5	Downstrear	N	90116578	90117577	5	38.0	9.1	34.3	14.3
chr2	Ap5s1	Downstrear	N	131039251	131040250	5	64.1	37.7	71.6	51.7
chr3	Nup210l	Downstrear	N	90015940	90016939	6	51.8	23.4	32.6	13.0
chr11	Mir10a	Downstrear	N	96178589	96179588	3	57.1	33.1	54.9	35.3
chr7	Cyb5r2	Downstrear	N	114890969	114891968	5	62.6	32.8	68.4	49.0
chr17	Jmjd8	Downstrear	N	25968789	25969788	1	52.3	36.6	55.4	36.1
chr12	Serpina3m	Downstrear	N	105632468	105633467	5	81.0	65.0	78.8	59.5
chr8	Myo16	Downstrear	N	10633951	10634950	5	75.0	26.8	51.6	32.4

chr15	Ttll8	Downstrea	N	88743328	88744327	5	80.8	62.5	73.4	54.2
chr4	Cited4	Downstrea	N	120340426	120341425	5	83.8	66.0	85.6	66.4
chr18	Pcdhb7	Downstrea	N	37504862	37505861	5	97.1	58.9	77.5	58.3
chr5	5033403H07Rik	Downstrea	N	53363251	53364250	5	64.8	35.8	76.5	57.5
chr12	Pomc	Downstrea	N	3960644	3961643	5	88.7	71.1	71.6	52.7
chr19	Atg2a	Downstrea	N	6262305	6263304	5	27.3	9.7	26.1	7.1
chr17	Gm6623	Downstrea	N	36318310	36319309	5	54.4	17.1	48.6	29.6
chr17	Ntn3	Downstrea	N	24339791	24340790	5	27.0	2.0	24.0	5.2
chr1	Nuak2	Downstrea	N	134230066	134231065	5	23.5	3.6	23.6	4.8
chr1	C030007H22Rik	Downstrea	N	91255917	91256916	5	84.4	58.6	77.3	58.6
chr11	Gemin4	Downstrea	N	76023073	76024072	5	76.3	60.2	75.6	57.0
chr5	Rhbdd2	Downstrea	N	136122247	136123246	5	84.7	62.3	83.9	65.3
chr10	Ctdsp2	Downstrea	N	126437032	126438031	5	90.9	56.0	72.5	54.1
chr8	Cdkn2aip	Downstrea	N	48793698	48794697	5	41.3	26.0	36.7	18.5
chr5	Sdad1	Downstrea	N	92712036	92713035	5	45.2	8.7	38.4	20.5
chr11	4933402P03Rik	Downstrea	N	69629068	69630067	5	85.0	64.3	89.9	72.1
chr17	Oard1	Downstrea	N	48556592	48557591	5	66.1	43.1	58.5	40.7
chr5	Niacr1	Downstrea	N	124312579	124313578	5	39.3	14.3	45.2	27.5
chr8	Cd209c	Downstrea	N	3939222	3940221	5	100.0	77.2	89.5	71.9
chr9	Apoa4	Downstrea	N	46051542	46052541	5	46.1	18.4	43.1	25.5
chr15	Troap	Downstrea	N	98913841	98914840	5	26.7	1.4	30.1	12.6
chr2	Nsfl1c	Downstrea	N	151337041	151338040	5	79.8	61.6	74.0	56.5
chr7	Calca	Downstrea	N	121773992	121774991	5	97.6	79.2	79.3	61.9
chr8	Emc8	Downstrea	N	123176814	123177813	5	88.5	52.9	57.3	39.9
chr7	Psg22	Downstrea	N	19312598	19313597	5	77.2	49.6	65.1	47.8
chr6	Gimap3	Downstrea	N	48713463	48714462	5	70.8	42.2	69.1	51.7
chr4	Pomgnt1	Downstrea	N	115832450	115833449	5	62.7	42.9	68.4	51.1
chr6	Pex26	Downstrea	N	121146204	121147203	5	74.0	44.2	84.3	67.1
chr7	Hsd17b14	Downstrea	N	52822692	52823691	5	43.3	22.6	41.4	24.3
chr3	Pde5a	Downstrea	N	122562293	122563292	5	66.5	48.2	64.2	47.2
chr4	Lrrc42	Downstrea	N	106905119	106906118	5	95.7	68.5	90.3	73.4
chr15	Mtss1	Downstrea	N	58771789	58772788	6	86.7	67.3	82.5	65.7
chr13	Slc28a3	Downstrea	N	58653669	58654668	5	93.3	76.5	90.5	73.7
chr16	Olfr190	Downstrea	N	59072981	59073980	5	90.5	75.0	100.0	83.5
chr9	Acy1	Downstrea	N	106334312	106335311	5	76.9	56.7	86.3	69.9
chr19	Fads3	Downstrea	N	10134162	10135161	5	36.9	9.1	24.8	8.4
chr4	Mir200a	Downstrea	N	155428005	155429004	6	34.8	8.6	36.0	19.7
chr9	Sdhd	Downstrea	N	50403445	50404444	5	49.1	2.6	56.9	40.6
chr4	1700042G07Rik	Downstrea	N	115846903	115847902	5	30.7	9.2	35.3	19.0
chr18	Mbp	Downstrea	N	82755030	82756029	5	84.1	65.9	87.1	70.8
chr16	Fopnl	Downstrea	N	14298337	14299336	5	93.0	72.6	98.5	82.2
chr2	Id1	Downstrea	N	152563147	152564146	5	17.6	1.3	19.4	3.6
chr4	Rpp25l	Downstrea	N	41658065	41659064	5	78.6	43.0	67.8	52.1
chr17	Clps	Downstrea	N	28694159	28695158	5	56.7	38.7	65.6	49.9
chr17	Tbl3	Downstrea	N	24836598	24837597	5	20.0	1.8	16.2	0.6
chr5	Fam109a	Downstrea	N	122304609	122305608	5	90.6	63.7	80.0	64.4
chr2	Cst8	Downstrea	N	148631321	148632320	5	88.4	70.6	82.1	66.6
chr2	Trp53i11	Downstrea	N	93041915	93042914	6	91.1	71.9	91.0	75.5
chr11	Fasn	Downstrea	N	120666272	120667271	5	41.0	20.4	43.2	27.7

chr2	Agbl2	Downstrear	N	90656389	90657388	5	30.0	2.5	28.3	12.9
chr10	Bloc1s1	Downstrear	N	128355970	128356969	6	61.0	37.0	62.7	47.3
chr15	Snord72	Downstrear	N	5068481	5069480	5	88.5	67.4	81.6	66.2
chr11	Chmp6	Downstrear	N	119780867	119781866	5	83.7	66.3	73.7	58.4
chr17	Prss32	Downstrear	N	23996744	23997743	5	91.4	59.4	83.7	68.4
chr4	1700125G02Rik	Downstrear	N	124567132	124568131	5	63.6	45.3	64.5	49.3
chr1	Ahctf1	Downstrear	N	181674035	181675034	5	100.0	41.9	71.9	56.7
chr2	Bcas1	Downstrear	N	170171491	170172490	5	81.0	29.7	22.6	7.4
chr4	E130309F12Rik	Downstrear	N	49353134	49354133	5	88.9	57.4	76.9	61.8
chr12	Ptgr2	Downstrear	N	85656783	85657782	4	39.2	16.7	36.4	21.3
chr14	Myh6	Downstrear	N	55559758	55560757	5	71.2	55.0	58.8	43.7
chr3	5330417C22Rik	Downstrear	N	108259987	108260986	5	59.9	44.1	66.7	51.6
chr8	Tnpo2	Downstrear	N	87581482	87582481	5	97.4	77.1	92.2	77.1
chr12	Crip2	Downstrear	N	114383718	114384717	4	79.7	64.4	66.2	51.2
chr5	Trafd1	Exon10	N	121834049	121834113	2	68.5	2.9	57.8	1.6
chr15	Baiap2l2	Exon10	N	79113856	79113917	1	29.2	0.0	50.0	0.0
chr9	Pfkfb4	Exon10	N	108930034	108930163	1	57.4	17.5	57.0	10.1
chr13	Msh3	Exon10	N	93044685	93044872	1	30.7	5.9	37.7	2.3
chr1	Camk1g	Exon10	N	195185913	195186041	1	77.4	40.4	77.6	43.8
chr16	Mylk	Exon10	N	34899447	34899599	1	52.5	31.3	71.9	39.7
chr7	Sox6	Exon10	N	122805786	122805958	1	55.6	18.1	63.8	32.0
chr2	Entpd6	Exon10	N	150592695	150592832	1	82.2	64.8	85.2	55.3
chr1	Sdccag8	Exon10	N	178804695	178804829	1	82.5	52.1	86.0	57.8
chr4	Rad54b	Exon10	N	11536441	11536616	1	25.7	0.0	28.9	1.9
chr3	Rapgef2	Exon10	N	78889667	78890050	2	85.0	67.9	91.3	65.2
chr2	Tnks1bp1	Exon10	N	84912253	84912325	2	76.9	5.1	30.6	5.9
chr10	Enpp1	Exon10	N	24376911	24377038	1	71.1	43.5	60.7	37.0
chr9	Hexa	Exon10	N	59410674	59410857	1	74.4	47.1	63.2	40.0
chr4	Lepre1	Exon10	N	118917562	118917712	1	74.6	46.3	63.1	40.1
chr19	Sorbs1	Exon10	N	40424389	40424503	1	79.4	47.6	75.5	54.7
chr13	Rreb1	Exon10	N	38038726	38039532	4	38.0	14.7	35.4	14.9
chr14	Ercc6	Exon10	N	33373895	33374011	1	96.7	63.6	91.4	71.2
chr1	Hnrnpu	Exon10	N	180264018	180264157	1	23.2	5.3	24.8	6.0
chr17	Vav1	Exon10	N	57440604	57440699	1	69.9	51.4	73.5	54.8
chr7	Vasp	Exon10	N	19849754	19849919	1	71.1	54.8	65.9	47.3
chr6	Lrp6	Exon10	N	134420707	134420909	1	37.7	20.2	43.1	25.4
chr13	Larp4b	Exon10	N	9150261	9150470	1	32.6	7.0	23.4	7.5
chr8	Fcer2a	Exon10	N	3691119	3691221	2	80.0	62.5	72.8	57.1
chr9	Myzap	Exon11	N	71427418	71427504	1	67.3	14.8	68.4	12.3
chr12	Slc26a3	Exon11	N	32146573	32146668	1	87.3	40.6	75.0	23.4
chr15	Baiap2l2	Exon11	N	79114437	79114523	1	53.5	5.0	46.7	10.2
chr2	Cacnb2	Exon11	N	14905878	14906066	1	64.0	10.9	44.3	15.0
chr10	Tcf3	Exon11	N	79881511	79881563	1	86.2	56.7	79.0	50.6
chr11	Tbrg4	Exon11	N	6525152	6525216	1	32.4	3.7	29.0	3.4
chr12	Baz1a	Exon11	N	56022025	56022132	1	89.2	71.1	84.5	59.2
chr1	Stau2	Exon11	N	16499423	16499489	1	75.0	54.3	69.8	46.8
chr4	E230008N13Rik	Exon11	N	45918881	45918969	1	81.5	61.6	72.2	50.5
chr5	Naa25	Exon11	N	121873544	121873751	1	78.9	47.1	75.7	55.4
chr8	Capn9	Exon11	N	127131855	127131891	1	81.0	52.9	76.0	56.4

chr5	Tacc3	Exon11	N	34012127	34012296	1	82.9	56.6	89.3	71.6
chr5	Pde6b	Exon11	N	108854252	108854398	1	72.5	47.6	75.0	58.2
chr17	Cyp4f13	Exon11	N	33083750	33083948	2	34.0	17.2	31.4	15.6
chr17	Notch4	Exon11	N	34710694	34710853	1	64.5	45.5	73.1	57.7
chr3	Clca4	Exon12	N	144510127	144510267	1	80.0	34.8	93.1	41.4
chr3	Ilf2	Exon12	N	90291149	90291239	1	88.6	58.3	92.3	48.5
chr14	Lmo7	Exon12	N	102297774	102297916	1	43.9	18.6	57.1	14.0
chr18	Arhgap26	Exon12	N	39387022	39387087	1	77.3	38.1	91.7	54.4
chr15	Plec	Exon12	N	76017041	76017193	1	83.2	25.5	64.2	27.9
chr6	Aak1	Exon12	N	86913987	86914219	1	76.3	58.0	82.4	46.2
chr15	Mtss1	Exon12	N	58886573	58886646	1	45.8	6.5	46.8	11.3
chr12	Ak7	Exon12	N	106999678	106999806	1	48.5	9.6	45.5	14.4
chr14	Mphosph8	Exon12	N	57315496	57315579	2	82.4	50.1	83.0	52.3
chr2	Cacnb2	Exon12	N	14905878	14906066	1	64.0	10.9	44.3	15.0
chr7	Mical2	Exon12	N	119466030	119466177	1	73.6	48.1	74.0	46.0
chr6	Copg1	Exon12	N	87847947	87848042	1	23.1	3.6	28.1	3.2
chr17	Abca3	Exon12	N	24520845	24520988	1	87.9	46.2	81.3	60.3
chr2	Znfx1	Exon12	N	166885532	166885642	2	66.7	0.0	21.2	1.2
chr4	Emc1	Exon12	N	138919572	138919694	1	95.8	69.8	89.6	69.9
chr7	Dmkn	Exon13	N	31561098	31561142	1	53.3	29.6	62.9	12.9
chr10	Aim1	Exon13	N	43699287	43699413	1	74.8	42.5	76.8	31.0
chr14	Gnl3	Exon13	N	31830994	31831052	1	34.7	17.2	46.3	8.5
chr17	Wdr46	Exon13	N	34086018	34086131	2	28.2	6.5	39.4	3.6
chr11	Stk10	Exon13	N	32512671	32512800	1	48.1	21.6	59.8	24.9
chr7	Unc45a	Exon13	N	87479581	87479749	1	34.4	6.7	36.0	2.3
chr13	Naip7	Exon13	N	101085939	101086630	5	65.1	11.5	52.6	21.0
chr18	Nars	Exon13	N	64674980	64675059	1	60.3	34.4	61.8	33.3
chr17	Trerf1	Exon13	N	47465967	47466042	1	36.2	6.5	31.0	2.7
chr14	Uggt2	Exon13	N	119428185	119428264	1	69.3	32.1	63.6	43.0
chr8	Myo16	Exon13	N	10400484	10400589	1	55.8	30.2	51.8	31.3
chr1	Aox3l1	Exon13	N	58361273	58361457	1	28.0	3.5	21.9	2.9
chr2	Cubn	Exon13	N	13239784	13239971	1	68.6	38.6	68.3	50.0
chr6	Osbpl3	Exon13	N	50296327	50296430	1	87.5	65.0	90.9	73.5
chr13	Dbn1	Exon13	N	55584748	55584803	1	69.0	51.7	73.5	56.2
chr2	Kif5c	Exon13	N	49585637	49585843	1	30.4	13.2	29.4	14.2
chr18	Me2	Exon14	N	73964706	73964823	2	98.4	4.8	54.8	1.9
chr6	Krba1	Exon14	N	48363362	48363502	1	69.5	23.0	70.2	18.6
chr15	Arfgap3	Exon14	N	83173452	83173570	1	26.9	0.9	48.4	1.2
chr11	Gemin5	Exon14	N	57959895	57960034	1	22.0	0.0	46.6	0.6
chr9	Pls1	Exon14	N	95696181	95696285	2	20.6	0.0	35.5	5.0
chr11	Tmc8	Exon14	N	117653403	117653481	1	30.7	11.3	34.0	6.6
chr2	Gsn	Exon14	N	35160101	35160178	1	68.9	39.6	73.9	49.5
chr16	4930562C15Rik	Exon14	N	4866204	4866284	1	34.0	0.0	27.6	3.7
chr16	Sidt1	Exon14	N	44269961	44270032	1	59.6	40.0	57.9	35.0
chr5	Baz1b	Exon14	N	135713789	135713936	1	86.2	69.1	83.0	65.3
chr9	Glb1l2	Exon14	N	26599367	26599475	1	91.3	60.7	93.8	76.2
chr3	Fnbp1l	Exon14	N	122293046	122293161	1	83.1	34.2	75.0	58.5
chr10	Ptprb	Exon14	N	115783855	115784118	1	51.7	19.6	51.7	35.9
chr1	Lamc2	Exon14	N	154991644	154991862	1	46.3	27.9	41.1	25.5

chr3	Ahcyl1	Exon14	N	107477929	107478072	1	75.4	50.0	62.1	46.7
chr9	Gramd1b	Exon15	N	40125094	40125178	1	78.0	4.2	85.7	3.2
chr5	Sdad1	Exon15	N	92731665	92731722	1	68.0	5.4	70.2	3.8
chr17	Cbs	Exon15	N	31769773	31770099	2	25.8	0.5	63.4	1.6
chr7	Eml2	Exon15	N	19787858	19787945	1	80.6	34.8	76.9	33.0
chr2	Kif5c	Exon15	N	49590953	49591141	1	51.1	17.5	47.1	17.2
chr4	Eif4g3	Exon15	N	137714306	137714425	1	98.1	82.8	91.2	70.1
chr7	Eps8l2	Exon15	N	148546221	148546326	2	88.6	58.5	88.2	69.2
chr5	Tctn2	Exon15	N	125075692	125075817	1	45.2	16.3	32.3	15.1
chr5	Anapc5	Exon15	N	123269617	123269696	1	44.7	24.0	50.0	34.8
chr16	Itsn1	Exon16	N	91837306	91837433	1	67.7	42.3	70.4	33.2
chr12	Mthfd1	Exon16	N	77401375	77401451	1	74.6	43.5	82.6	49.7
chr8	Palld	Exon16	N	64191609	64191675	1	42.1	20.9	52.2	22.4
chr2	Cep110	Exon16	N	34999734	34999941	1	91.4	46.5	82.5	58.0
chr7	Adam8	Exon16	N	147174710	147174778	1	50.8	11.8	56.8	33.3
chr9	Kri1	Exon16	N	21091023	21091128	1	80.8	65.0	82.6	59.4
chr6	Eogt	Exon16	N	97097871	97098252	1	34.8	5.7	28.5	8.6
chr11	Ap1b1	Exon16	N	4937635	4937780	1	78.7	61.0	84.4	65.9
chr17	Tiam2	Exon17	N	3506767	3506892	1	85.0	63.8	78.3	35.0
chr2	Pla2g4f	Exon17	N	120138043	120138179	1	57.1	39.8	62.8	26.3
chr2	Armc3	Exon17	N	19222099	19222296	1	45.9	15.6	56.6	22.5
chr17	Abcc10	Exon17	N	46459086	46459242	1	92.4	35.4	89.5	55.6
chr8	Tnks	Exon17	N	35930748	35930839	1	54.9	38.2	66.0	35.4
chr9	Megf11	Exon17	N	64540162	64540290	1	49.2	0.0	28.6	0.0
chr3	Sema6c	Exon17	N	94975256	94975311	1	22.9	0.0	21.3	0.0
chr14	Piwil2	Exon17	N	70820447	70820557	1	75.4	53.3	70.8	51.6
chr11	Ccdc40	Exon17	N	119124389	119124547	1	60.7	43.9	70.6	54.5
chr19	Plce1	Exon18	N	38820256	38820385	1	60.0	13.1	78.1	17.3
chr5	Wdr19	Exon18	N	65622517	65622627	1	84.6	54.5	94.7	44.9
chr13	Naa35	Exon18	N	59730279	59730346	1	93.7	64.9	91.1	45.0
chr7	Ptprh	Exon18	N	4554650	4554815	2	58.6	17.4	54.5	9.3
chr18	Adamts19	Exon18	N	59184101	59184236	1	85.4	53.3	72.7	39.3
chr4	Rere	Exon18	N	149990050	149990272	1	35.1	1.7	31.0	1.1
chr11	Caskin2	Exon18	N	115673201	115673696	4	63.6	31.7	59.5	32.1
chr2	Cwc22	Exon18	N	77774650	77774779	2	79.1	46.8	77.4	52.3
chr9	Dync2h1	Exon18	N	7016730	7016795	1	86.1	70.3	79.5	55.2
chr17	Pde10a	Exon18	N	9167588	9167699	1	63.3	29.3	54.3	30.9
chr7	Eif4g2	Exon18	N	118223696	118223836	1	63.4	32.4	64.0	43.4
chr15	Tnrc6b	Exon18	N	80747114	80747290	1	88.0	69.5	85.7	65.9
chr5	Gsap	Exon18	N	20763688	20763741	1	35.3	11.1	21.7	3.7
chr7	Ctr9	Exon19	N	118194459	118194594	1	51.4	19.4	64.0	19.3
chr6	Plekha5	Exon19	N	140518842	140518991	1	72.5	27.7	58.1	36.9
chr18	Spink5	Exon19	N	44166025	44166118	1	58.5	43.2	68.9	48.1
chr9	Dlec1	Exon19	N	119043587	119043740	1	50.8	32.0	36.5	18.6
chr13	Arhgef28	Exon19	N	98740270	98740325	1	88.9	60.0	80.6	64.9
chr14	Ldb3	Exon2	N	35355054	35355174	1	60.0	0.0	80.5	1.4
chr5	Ugt2b35	Exon2	N	87433868	87433999	1	72.7	7.5	86.7	12.3
chr14	Dock9	Exon2	N	121945778	121945878	1	86.4	7.6	84.2	12.5
chr11	BC018473	Exon2	N	116615648	116615803	1	80.8	4.8	84.3	15.4

chr8	Bag4	Exon2	N	26881551	26881805	1	79.1	6.4	78.4	11.6
chr10	Rdh1	Exon2	N	127201738	127201901	1	81.1	0.0	63.3	10.3
chr2	Gata5	Exon2	N	180062465	180062552	1	60.5	0.0	61.0	8.1
chr13	Cap2	Exon2	N	46626346	46626446	1	72.0	11.0	67.8	17.3
chr6	Cd9	Exon2	N	125412132	125412221	1	27.8	0.0	52.2	2.9
chr17	Msrb1	Exon2	N	24877025	24877139	1	39.7	2.7	54.5	5.9
chr7	Tmem150b	Exon2	N	4675911	4675978	1	75.0	44.0	70.7	22.2
chr11	Gabarap	Exon2	N	69806084	69806202	1	62.9	12.5	57.8	9.6
chr17	Taf8	Exon2	N	47630511	47630653	1	85.6	24.0	73.9	26.2
chr11	Krt19	Exon2	N	100002837	100002998	1	48.7	19.9	62.3	14.7
chr9	Acaa1a	Exon2	N	119251841	119251898	1	91.0	31.3	91.9	44.8
chr13	Ercc8	Exon2	N	108956584	108956685	1	70.5	53.0	76.2	29.3
chr16	Drd3	Exon2	N	43807491	43807633	1	69.2	30.0	66.3	20.2
chr11	Gm11978	Exon2	N	6551368	6551453	1	54.7	19.1	60.5	15.1
chr9	C430002N11Rik	Exon2	N	96670370	96670515	1	47.4	8.8	47.3	3.7
chr15	4930415O20Rik	Exon2	N	98418984	98419128	1	77.8	46.2	96.6	54.5
chr6	Plxnd1	Exon2	N	115906613	115906753	1	68.7	32.4	73.2	31.2
chr7	Syne4	Exon2	N	31101249	31101392	1	79.5	41.4	85.4	44.9
chr3	Pdzk1	Exon2	N	96655414	96655663	1	77.9	50.7	83.2	43.8
chr3	Phf17	Exon2	N	41392856	41392944	1	56.1	24.1	60.0	21.9
chr10	Specc1l	Exon2	N	74703810	74703963	1	59.8	11.3	70.5	32.5
chr9	Fbxo9	Exon2	N	77933714	77933807	1	75.4	28.3	69.0	31.6
chr7	Ears2	Exon2	N	129187816	129187946	1	62.4	28.3	69.4	32.0
chr12	4933406C10Rik	Exon2	N	33605688	33605768	1	78.3	50.0	81.6	44.4
chr5	Hadha	Exon2	N	30447228	30447342	1	89.2	54.2	66.0	29.0
chr1	Soat1	Exon2	N	158362978	158363113	1	70.5	42.0	73.2	36.3
chr2	Ssfa2	Exon2	N	79479414	79479463	1	36.8	10.0	45.3	9.8
chr17	Gm17830	Exon2	N	49447554	49447729	1	61.7	34.4	61.5	27.7
chr2	Med22	Exon2	N	26764513	26764593	1	54.6	35.3	56.1	23.6
chr14	Slc7a7	Exon2	N	54992624	54992773	1	50.0	21.9	49.2	17.3
chr17	Nfya	Exon2	N	48531201	48531374	1	76.5	49.2	71.5	40.3
chr10	Itga7	Exon2	N	128376324	128376403	1	27.8	3.6	30.7	0.0
chr11	Krt40	Exon2	N	99401186	99401311	1	53.3	30.8	62.2	32.2
chr6	Rpusd3	Exon2	N	113366783	113366906	1	93.5	63.1	84.4	54.5
chr15	Slc2a13	Exon2	N	91106798	91106919	1	65.0	25.7	51.1	21.6
chr5	Rpl21	Exon2	N	147646024	147646085	1	62.5	25.0	63.5	34.6
chr3	Dennd2d	Exon2	N	106290769	106290881	2	86.4	35.6	73.5	45.1
chr11	Arsg	Exon2	N	109347020	109347071	1	78.6	55.9	76.7	48.6
chr2	Obp2b	Exon2	N	25593519	25593589	1	56.7	33.3	62.5	34.4
chr1	Dis3l2	Exon2	N	88641916	88642073	1	93.7	65.2	84.3	56.2
chr7	Slx1b	Exon2	N	133835442	133835569	1	68.2	45.8	89.5	61.5
chr11	Cntd1	Exon2	N	101145035	101145206	1	24.0	3.3	32.7	5.1
chr12	Atxn7l1	Exon2	N	34026786	34027060	1	88.2	54.8	89.8	62.6
chr10	Arhgap9	Exon2	N	126762579	126762686	1	39.7	12.1	37.7	10.9
chr13	Dbn1	Exon2	N	55576221	55576897	3	62.5	4.3	71.0	44.3
chr17	Safb	Exon2	N	56733113	56733177	1	57.5	40.7	65.7	39.3
chr19	E030003E18Rik	Exon2	N	20627589	20627690	2	78.9	48.9	88.3	61.9
chr6	Clec4f	Exon2	N	83596782	83596933	1	49.5	20.2	46.5	20.8
chr13	Slc12a7	Exon2	N	73922492	73922614	1	77.1	59.1	80.2	54.5

chr11	Peli1	Exon2	N	21040567	21040696	1	66.7	47.8	71.1	46.1
chr1	Hnrnpu	Exon2	N	180260296	180260477	1	29.4	4.0	33.6	8.6
chr17	Abca17	Exon2	N	24402368	24402558	1	69.6	37.0	62.9	38.8
chr3	Pex11b	Exon2	N	96441054	96441255	1	68.2	40.2	65.3	41.1
chr5	Mapk10	Exon2	N	103357242	103357305	1	42.9	10.6	31.0	7.1
chr19	Ppp1r14b	Exon2	N	7051348	7051380	1	25.0	7.4	29.3	5.4
chr12	1700086L19Rik	Exon2	N	75389700	75389921	1	54.1	32.0	44.8	21.3
chr11	Gnb2l1	Exon2	N	48615775	48615922	2	33.8	12.1	39.9	16.6
chr7	Pddc1	Exon2	N	148595230	148595323	1	62.3	30.2	51.8	29.2
chr8	Ido2	Exon2	N	25651066	25651117	1	74.3	50.6	85.3	62.7
chr2	Rin2	Exon2	N	145648001	145648101	2	34.5	0.0	22.7	0.2
chr5	Reln	Exon2	N	21402565	21402762	1	35.1	16.3	35.6	15.3
chr11	Dnajc7	Exon2	N	100445525	100445677	1	70.9	55.3	81.7	61.4
chr19	Fam178a	Exon2	N	45009423	45010156	4	73.2	34.5	71.5	51.3
chr1	Nek2	Exon2	N	193646371	193646611	1	22.5	3.5	25.3	5.3
chr5	Hnrpdl	Exon2	N	100465500	100465670	1	35.6	14.9	40.4	20.5
chr8	Pbx4	Exon2	N	72388211	72388449	1	58.7	35.9	57.5	37.9
chr5	Rplp0	Exon2	N	116010750	116011013	1	34.0	19.0	37.3	17.9
chr6	Retsat	Exon2	N	72551470	72551711	1	75.7	47.9	74.7	55.6
chr11	4732490B19Rik	Exon2	N	113070300	113070508	1	29.9	4.6	22.9	3.9
chr15	Tomm22	Exon2	N	79502327	79502444	1	35.6	11.8	26.3	7.3
chr2	Bpifa2	Exon2	N	153835503	153835644	1	56.9	38.6	63.4	44.4
chr11	Lig3	Exon2	N	82598817	82598960	1	65.7	22.5	67.8	49.1
chr13	Mak	Exon2	N	41127894	41127968	1	77.8	57.4	65.5	46.9
chr2	Ckap2l	Exon2	N	129098233	129098293	1	65.5	27.3	61.2	43.6
chr7	Ccdc101	Exon2	N	133807443	133807518	1	60.8	28.0	48.4	31.4
chr9	Taf1d	Exon2	N	15112969	15113359	2	64.2	46.3	62.1	45.1
chr2	Wfdc6b	Exon2	N	164442776	164442946	1	66.7	45.1	70.0	53.0
chr2	Tgm7	Exon2	N	120921591	120921751	1	83.3	61.0	76.2	59.5
chr5	Tmem120a	Exon2	N	136211940	136212004	2	85.8	56.0	92.6	75.9
chr12	Ifrd1	Exon2	N	40930884	40931012	1	90.7	72.9	91.7	75.2
chr2	Psmc3	Exon2	N	90895136	90895261	1	76.9	57.7	78.1	61.9
chr7	Axl	Exon2	N	26546424	26546583	1	88.0	72.1	92.5	76.4
chr9	Ncapd3	Exon2	N	26844570	26844724	1	82.0	57.8	80.7	64.7
chr17	Zfp760	Exon2	N	21846172	21846263	2	76.5	60.0	81.4	65.4
chr3	Skil	Exon2	N	30996192	30996387	2	85.4	52.2	66.5	50.7
chr9	Usp2	Exon2	N	43897215	43897265	1	46.9	17.5	25.0	9.2
chr19	Pcgf6	Exon2	N	47114534	47114632	1	43.1	20.0	27.8	12.0
chr10	Poc1b	Exon2	N	98587465	98587636	1	86.6	67.1	83.7	68.2
chr15	Mrpl13	Exon2	N	55371678	55371764	1	27.3	9.8	15.4	0.0
chr12	Fam49a	Exon2	N	12310987	12311128	2	100.0	48.4	83.0	67.6
chr1	Unc50	Exon2	N	37489396	37489516	1	85.0	69.6	87.2	72.0
chr11	Cacng1	Exon2	N	107567091	107567165	1	60.0	43.3	60.3	45.2
chr9	Acpl2	Exon2	N	96739505	96739631	1	44.3	26.6	40.2	25.2
chr1	Astn1	Exon2	N	160432179	160432572	2	50.7	24.1	61.2	46.2
chr19	Capn1	Exon20	N	6014201	6014468	4	41.6	16.9	66.0	14.5
chr7	Herc2	Exon20	N	63376915	63377099	1	88.1	58.3	76.0	54.0
chr9	Trank1	Exon20	N	111296262	111296360	1	67.8	35.6	54.1	33.0
chr9	Sorl1	Exon20	N	41812069	41812200	1	80.9	56.2	84.3	64.3

chr14	Ttc18	Exon20	N	21251146	21251245	1	28.7	5.4	21.2	1.2
chr15	Rapgef3	Exon20	N	97589756	97589816	1	90.0	61.5	75.0	57.6
chr10	Cabin1	Exon21	N	75200066	75200260	1	51.1	0.0	68.4	0.5
chr19	Plce1	Exon21	N	38833305	38833436	1	43.9	20.0	63.3	11.5
chr8	Slc12a4	Exon21	N	108483258	108483389	1	44.5	2.7	45.6	2.7
chr1	Pik3c2b	Exon21	N	134990233	134990362	1	50.0	11.1	65.4	25.6
chr15	Col14a1	Exon21	N	55254936	55255085	1	77.3	61.9	74.6	54.2
chr10	Stab2	Exon21	N	86335342	86335488	1	56.2	39.2	54.5	35.6
chr8	Pkd1l3	Exon21	N	112172404	112172638	1	86.4	46.2	78.4	61.3
chr1	Adcy10	Exon22	N	167486880	167487111	1	57.9	38.9	63.8	23.1
chr8	Slc12a4	Exon22	N	108484576	108484670	1	73.1	41.8	91.1	52.9
chr5	Pitpnm2	Exon22	N	124602553	124602728	2	47.4	13.6	55.7	27.2
chr13	Itga2	Exon23	N	115667829	115667977	1	67.1	17.8	63.1	25.0
chr10	Mon2	Exon23	N	122469579	122469805	1	75.0	37.8	68.1	32.7
chr1	Als2	Exon23	N	59253035	59253215	1	76.9	57.1	85.4	57.1
chr8	Ank1	Exon23	N	24222282	24222360	1	60.5	40.4	66.3	50.0
chr10	Stab2	Exon24	N	86341575	86341694	1	50.6	6.4	54.6	1.8
chr2	Itgav	Exon24	N	83636243	83636401	1	96.4	56.7	87.1	59.8
chr14	Abcc4	Exon24	N	119028840	119028965	1	79.4	52.8	77.9	54.0
chr19	Abcc2	Exon24	N	43901074	43901273	1	58.9	33.2	53.0	33.0
chr11	Myh10	Exon24	N	68605365	68605536	1	29.9	5.8	23.0	5.6
chr9	Nfrkb	Exon24	N	31227399	31227534	1	78.0	59.0	77.4	60.9
chr6	Vwf	Exon24	N	125587398	125587554	1	81.7	62.7	82.3	66.7
chr8	Kcnu1	Exon25	N	27044877	27044989	1	63.3	2.9	57.7	5.2
chr7	Usp47	Exon25	N	119250293	119250423	1	76.2	58.3	76.4	53.6
chr2	Plcb1	Exon26	N	135196157	135196337	1	45.2	3.9	58.7	7.3
chr5	Stag3	Exon26	N	138745270	138745418	1	56.9	26.9	57.5	27.6
chr11	Abca9	Exon26	N	110006805	110006980	1	86.0	57.8	82.5	54.3
chr13	Zcchc6	Exon26	N	59922921	59923471	4	53.8	16.5	68.3	40.4
chr7	Kndc1	Exon26	N	147118742	147118892	1	90.9	72.7	97.2	81.3
chr12	Greb1	Exon27	N	16730097	16730240	1	22.0	0.0	26.3	0.0
chr1	Aox3l1	Exon27	N	58403069	58403197	1	19.3	0.0	21.5	0.0
chr19	Col17a1	Exon27	N	47738293	47738346	1	83.9	63.8	83.5	63.9
chr10	Utrn	Exon27	N	12360725	12360842	1	87.5	40.7	87.1	69.7
chr4	Fhad1	Exon27	N	141538063	141538305	2	36.6	16.8	32.1	16.5
chr1	Cacna1s	Exon28	N	138001147	138001203	1	55.4	13.8	62.7	33.7
chr7	Herc2	Exon29	N	63390856	63391051	1	40.7	0.8	53.9	2.5
chr1	Clasp1	Exon29	N	120456563	120456706	1	65.9	36.8	80.6	41.1
chr17	Itpr3	Exon29	N	27243151	27243351	1	48.3	5.2	41.5	5.3
chr9	Col5a3	Exon29	N	20590017	20590070	1	79.5	63.4	79.6	62.9
chr19	Fads2	Exon3	N	10140742	10140838	1	85.0	4.3	73.8	0.0
chr9	Nr2e3	Exon3	N	59796170	59796327	1	72.4	4.7	78.4	14.3
chr13	Pde8b	Exon3	N	95802785	95802905	1	65.0	8.2	65.7	4.4
chr12	Nfkbia	Exon3	N	56592107	56592317	1	67.4	12.5	61.0	8.8
chr7	Kcne3	Exon3	N	107331243	107331333	1	45.2	16.5	64.0	13.2
chr11	Chmp6	Exon3	N	119777339	119777425	1	65.4	35.1	73.2	23.1
chr8	Col4a1	Exon3	N	11206226	11206403	1	87.1	50.0	90.0	43.5
chr17	Epcam	Exon3	N	88039628	88039868	1	79.4	21.5	68.3	23.5
chr5	9130230L23Rik	Exon3	N	66391444	66391694	1	17.6	1.1	44.8	2.5

chr7	Nlrp6	Exon3	N	148108309	148110055	3	59.4	5.6	50.9	9.9
chr15	Twf1	Exon3	N	94413181	94413306	1	52.0	25.0	58.7	23.9
chr16	Rpl35a	Exon3	N	33058765	33058909	1	77.3	46.1	74.4	40.1
chr15	Krt71	Exon3	N	101568377	101568502	1	68.0	24.6	62.8	29.2
chr11	Plekhm1	Exon3	N	103232176	103232369	1	87.5	45.7	84.2	50.8
chr7	Asb7	Exon3	N	73826591	73826694	1	73.1	32.4	70.3	37.0
chr7	Ptpre	Exon3	N	142843436	142843529	2	61.0	7.8	35.7	3.1
chr3	Ctso	Exon3	N	81748784	81748951	1	88.2	52.5	88.3	56.4
chr1	Vil1	Exon3	N	74464934	74465130	1	90.1	74.8	87.2	56.5
chr2	Tnfaip6	Exon3	N	51906363	51906591	1	67.5	41.9	63.5	32.8
chr1	Cacybp	Exon3	N	162136744	162136840	1	63.6	40.4	71.4	42.4
chr10	Vnn3	Exon3	N	23584144	23584435	1	85.3	59.0	86.6	58.5
chr4	Txn1	Exon3	N	57964669	57964773	1	36.8	0.0	27.8	0.0
chr5	Lrrc66	Exon3	N	74020770	74021249	3	40.8	6.0	29.2	1.6
chr3	Rps3a1	Exon3	N	85945161	85945348	2	33.2	6.7	36.9	10.1
chr11	4930401O10Rik	Exon3	N	71753075	71753281	1	82.8	60.7	78.3	52.4
chr4	Lck	Exon3	N	129229192	129229268	1	56.0	38.2	83.3	57.9
chr7	Rpl18	Exon3	N	52975290	52975388	1	71.4	44.0	74.0	49.6
chr17	Fez2	Exon3	N	78799970	78800238	1	89.3	52.9	83.4	59.0
chr4	Esrp1	Exon3	N	11280442	11280610	1	25.2	2.7	30.3	5.9
chr2	Rnf114	Exon3	N	167332547	167332697	1	84.1	50.3	81.8	57.4
chr1	Nek7	Exon3	N	140412202	140412309	1	16.7	0.0	27.9	3.8
chr16	App	Exon3	N	84971653	84971706	2	68.9	53.7	79.3	56.2
chr13	4933433G19Rik	Exon3	N	67104075	67104205	1	92.9	56.5	82.4	59.3
chr8	Chd9	Exon3	N	93480282	93480613	2	36.9	12.9	35.3	12.5
chr11	Smurf2	Exon3	N	106689818	106689938	1	45.4	1.2	34.5	11.8
chr4	Slc26a7	Exon3	N	14437294	14437394	1	36.5	13.0	35.6	14.6
chr10	Tpd52l1	Exon3	N	31066417	31066518	1	92.8	71.8	92.6	71.6
chr7	Tjp1	Exon3	N	72447630	72448104	2	77.5	36.8	68.9	48.1
chr6	Cd27	Exon3	N	125184533	125184712	1	33.3	14.0	32.0	12.0
chr3	Gpr89	Exon3	N	96679551	96679646	1	91.4	28.8	66.9	47.2
chr3	Setd7	Exon3	N	51336897	51336978	1	85.3	49.2	79.5	59.9
chr11	Cldn7	Exon3	N	69780722	69780806	2	27.7	9.0	31.4	12.4
chr18	Rpl17	Exon3	N	75161320	75161454	2	27.1	9.1	26.7	8.0
chr15	Mapk11	Exon3	N	88975526	88975605	1	80.5	47.9	78.0	59.6
chr4	Pla2g2c	Exon3	N	138290177	138290321	1	46.7	5.3	24.8	6.3
chr7	Ccp110	Exon3	N	125861481	125861609	1	92.1	75.7	92.3	74.1
chr9	Bsn	Exon3	N	108006275	108006406	1	52.1	33.8	62.5	44.3
chr9	Ldlr	Exon3	N	21537935	21538318	2	85.2	64.2	68.0	50.3
chr18	Slc14a2	Exon3	N	78352246	78352393	1	65.6	49.6	56.3	38.6
chr17	Sod2	Exon3	N	13206299	13206478	1	50.0	24.1	48.6	31.6
chr1	Mrpl15	Exon3	N	4774032	4774186	1	84.1	55.3	76.3	59.3
chr10	Arhgap9	Exon3	N	126762900	126762958	1	87.5	66.7	79.2	62.5
chr3	Efna1	Exon3	N	89080052	89080347	2	82.8	59.4	78.8	62.2
chr6	Cd9	Exon3	N	125412345	125412443	1	93.2	71.7	92.5	75.9
chr5	Trmt44	Exon3	N	35908010	35908193	1	50.6	7.0	27.7	11.4
chr11	Rpl38	Exon3	N	114533048	114533170	1	77.8	37.1	50.8	34.7
chr8	Adam18	Exon3	N	25736259	25736339	1	29.5	0.0	30.2	14.1
chr4	H6pd	Exon3	N	149369854	149370490	3	72.0	47.7	67.0	51.4

chr7	Ccdc101	Exon3	N	133808430	133808502	1	37.9	11.9	25.5	9.9
chr11	Nipsnap1	Exon3	N	4784025	4784119	1	28.6	4.3	17.2	1.8
chr5	AW549542	Exon3	N	120024531	120025670	6	60.3	37.5	51.0	35.7
chr15	Parvb	Exon3	N	84113192	84113294	1	73.8	55.9	76.2	60.9
chr6	Mat2a	Exon3	N	72385805	72386023	1	91.9	75.7	86.6	71.4
chr6	Atn1	Exon3	N	124694738	124695434	1	97.4	73.2	85.1	70.0
chr9	Sorl1	Exon30	N	41843526	41843657	1	60.8	34.7	71.4	28.6
chr6	Pzp	Exon30	N	128472034	128472202	1	78.9	62.7	70.7	43.8
chr10	Ptprq	Exon30	N	107104397	107104579	1	84.5	51.1	85.4	65.0
chr7	Tenm4	Exon31	N	104051355	104051497	1	47.3	13.8	50.0	9.0
chr10	Mon2	Exon32	N	122489241	122489368	1	40.4	0.0	23.5	0.0
chr2	Cubn	Exon33	N	13279746	13279856	1	33.3	3.8	51.0	13.5
chr19	Myof	Exon33	N	38034204	38034328	1	66.0	43.6	79.6	43.0
chr14	Tep1	Exon34	N	51465091	51465215	1	67.6	13.2	60.4	7.7
chr16	Urb1	Exon34	N	90800265	90800361	1	87.9	72.1	87.8	70.7
chr2	Ubr3	Exon35	N	69856450	69856629	1	74.2	50.0	83.1	51.0
chr4	Unc13b	Exon36	N	43272334	43272481	1	36.7	5.9	20.8	2.3
chr14	Wdfy4	Exon38	N	33914689	33914800	1	82.6	53.1	77.0	53.6
chr10	Syne1	Exon38	N	5274338	5274504	1	82.7	58.8	76.7	58.7
chr7	Muc5ac	Exon38	N	149000359	149000449	1	85.2	62.5	78.1	61.8
chr19	Fads2	Exon4	N	10141005	10141102	1	61.0	17.7	59.5	1.6
chr9	Cep63	Exon4	N	102498452	102498649	1	43.9	7.3	57.3	0.0
chr13	2010111I01Rik	Exon4	N	63257906	63258095	1	48.2	28.1	67.8	12.6
chr7	Sphk2	Exon4	N	52968630	52969098	3	59.6	14.5	65.2	16.6
chr2	Il1b	Exon4	N	129195439	129195487	1	82.7	46.6	75.5	30.3
chr5	1700028K03Rik	Exon4	N	107975220	107975298	1	54.2	10.9	46.6	3.4
chr4	Ptch2	Exon4	N	116777839	116777930	1	86.5	42.9	80.0	37.2
chr4	Plekhg5	Exon4	N	151477860	151477951	1	75.7	35.9	83.0	40.8
chr5	Srpk2	Exon4	N	23024437	23024537	1	75.0	5.7	61.8	19.8
chr8	Plekha2	Exon4	N	26168205	26168273	1	85.6	24.3	87.3	46.1
chr8	Glt25d1	Exon4	N	74141974	74142178	1	78.1	42.9	86.9	46.5
chr10	Ostm1	Exon4	N	42417914	42418079	1	53.6	10.0	55.3	16.7
chr2	Gbgt1	Exon4	N	28357717	28357752	1	55.2	23.1	67.9	29.6
chr5	Plbd2	Exon4	N	120937681	120937776	1	60.3	34.5	69.0	31.4
chr8	Ifi30	Exon4	N	73288618	73288662	1	60.9	41.7	66.7	30.0
chr1	Atp1b1	Exon4	N	166383620	166383748	1	30.0	1.5	38.2	3.3
chr12	Serpina1f	Exon4	N	104932807	104932899	1	84.6	43.3	67.6	33.3
chr13	Sgtb	Exon4	N	104914270	104914369	1	58.0	11.3	61.7	27.8
chr2	9830001H06Rik	Exon4	N	156853123	156853374	1	81.0	57.8	84.0	51.3
chr17	Flot1	Exon4	N	35961511	35961654	1	90.9	68.6	90.6	59.3
chr18	Htr4	Exon4	N	62587677	62587830	1	31.1	14.4	45.1	14.1
chr17	Slc22a1	Exon4	N	12852412	12852626	1	61.8	3.2	32.6	2.8
chr1	Nit1	Exon4	N	173274419	173274670	2	66.4	48.6	58.8	29.9
chr1	2310007B03Rik	Exon4	N	95056178	95056927	5	50.0	10.1	40.5	13.5
chr11	Ppp1r1b	Exon4	N	98216641	98216823	3	77.7	51.8	75.1	48.6
chr8	Slc12a3	Exon4	N	96857433	96857572	1	30.4	1.8	28.7	2.3
chr4	Acer2	Exon4	N	86557520	86557657	1	51.2	7.8	47.8	22.7
chr8	B930025P03Rik	Exon4	N	10879782	10879985	1	30.2	9.8	31.3	7.1
chr10	Slc5a4a	Exon4	N	75619763	75619867	1	80.7	50.0	74.8	51.5

chr11	Hnrnph1	Exon4	N	50194961	50195099	1	41.8	11.4	32.9	10.5
chr4	Zc3h12a	Exon4	N	124803850	124804326	3	79.8	60.6	80.8	58.7
chr11	E230016K23Rik	Exon4	N	83424625	83424724	1	59.0	7.5	47.2	25.5
chr11	Sap30bp	Exon4	N	115818675	115818763	1	43.6	7.8	32.7	11.1
chr11	Eif5a	Exon4	N	69732653	69732838	2	67.6	44.0	72.4	51.1
chr16	Ets2	Exon4	N	95933693	95933893	1	85.7	65.6	76.2	55.1
chr1	Rpl7	Exon4	N	16093249	16093415	1	79.2	43.3	69.6	48.5
chr19	Cyb561a3	Exon4	N	10659672	10659880	1	27.5	1.4	23.9	2.9
chr1	Npl	Exon4	N	155362532	155362680	1	68.6	28.4	74.7	54.1
chr18	Stard4	Exon4	N	33368697	33368811	1	86.7	67.6	87.6	67.5
chr9	Sesn3	Exon4	N	14118940	14119176	1	91.1	72.3	90.2	70.3
chr2	Mapk8ip1	Exon4	N	92225570	92225679	1	71.2	46.7	67.6	48.2
chr14	Mettl6	Exon4	N	32306783	32307150	2	81.5	60.0	86.4	67.1
chr3	Schip1	Exon4	N	68421533	68421730	1	91.1	52.8	78.0	59.0
chr12	Entpd5	Exon4	N	85725169	85725270	1	98.0	67.2	92.7	74.3
chr1	Ilkap	Exon4	N	93279881	93279968	1	90.5	71.0	90.3	72.0
chr2	Acot8	Exon4	N	164628497	164628633	1	81.3	65.3	86.9	68.7
chr5	Pi4k2b	Exon4	N	53144335	53144488	1	83.6	47.6	81.5	63.6
chr7	Ech1	Exon4	N	29615021	29615069	1	95.7	68.3	93.1	75.6
chr11	Slfn5	Exon4	N	82773507	82774230	5	78.7	58.6	73.4	56.5
chr7	Mboat7	Exon4	N	3642864	3642990	1	54.5	27.3	35.8	19.0
chr11	Myo15	Exon4	N	60296539	60296648	1	78.1	56.4	76.7	59.9
chr10	Cpm	Exon4	N	117107223	117107407	1	90.0	73.8	87.8	71.1
chr2	Ncs1	Exon4	N	31139667	31139755	1	68.6	52.8	66.5	51.0
chr12	2700097O09Rik	Exon4	N	56160444	56160565	1	82.5	54.3	83.5	68.1
chr7	Muc2	Exon41	N	148938582	148938680	1	49.2	19.6	46.4	22.6
chr12	Eml5	Exon41	N	100120186	100120284	1	78.4	52.4	71.9	54.4
chr18	Lama3	Exon44	N	12683015	12683125	1	90.7	31.6	88.7	58.3
chr9	Sorl1	Exon45	N	41907228	41907353	1	92.5	75.9	88.0	66.7
chr5	Zan	Exon49	N	137867392	137867533	1	50.9	29.6	51.3	34.2
chr9	Nr2e3	Exon5	N	59796732	59796835	1	87.5	20.2	94.3	16.7
chr3	Car14	Exon5	N	95703663	95703729	1	55.6	11.6	67.3	8.7
chr4	Trit1	Exon5	N	122726012	122726123	1	32.9	0.0	56.5	0.0
chr1	Rgsl1	Exon5	N	155641218	155641356	1	30.0	7.1	58.0	9.5
chr7	Ceacam1	Exon5	N	26258817	26259071	1	54.5	12.6	57.5	10.4
chr9	Fbxo22	Exon5	N	55068829	55068994	1	89.6	26.9	82.3	36.1
chr3	Adam15	Exon5	N	89145859	89145997	1	87.1	54.2	91.7	47.2
chr13	Adtrp	Exon5	N	41923512	41923754	2	60.5	2.4	48.6	4.6
chr9	Arih2	Exon5	N	108512109	108512130	1	87.0	50.0	94.2	52.1
chr2	Tcp11l1	Exon5	N	104537667	104537887	1	74.4	33.8	65.9	27.8
chr13	Aldh5a1	Exon5	N	25011548	25011691	1	67.8	52.5	81.9	45.1
chr8	Tlr3	Exon5	N	46495150	46495344	2	61.9	8.3	53.8	17.7
chr1	Sccpdh	Exon5	N	181609716	181609846	1	63.6	39.6	74.0	38.7
chr4	Tspan1	Exon5	N	115836924	115837130	1	78.5	32.2	71.2	37.7
chr13	4933425L06Rik	Exon5	N	105908731	105908878	1	79.5	35.4	62.6	30.7
chr10	Mtrf1l	Exon5	N	4532364	4532500	1	55.5	25.6	75.8	45.2
chr17	BC004004	Exon5	N	29431220	29431359	1	38.1	4.0	33.1	3.1
chr7	4732471J01Rik	Exon5	N	26316310	26316481	1	86.6	52.0	82.0	52.8
chr9	Mns1	Exon5	N	72296958	72297174	1	89.1	70.3	93.4	65.8

chr8	Tex29	Exon5	N	11854976	11855038	1	84.0	44.4	82.8	55.6
chr3	Slc25a24	Exon5	N	108961416	108961562	1	20.8	5.5	26.9	1.0
chr7	Il18bp	Exon5	N	109166111	109166178	1	37.2	2.3	25.4	0.0
chr5	Fastk	Exon5	N	23948000	23948213	1	75.6	40.0	67.3	43.5
chr7	Sphk2	Exon5	N	52972363	52972433	1	67.5	9.1	54.8	31.0
chr1	Cyp27a1	Exon5	N	74783255	74783421	1	73.4	51.7	74.1	51.0
chr15	Zfpm2	Exon5	N	40927605	40927811	1	67.1	25.0	52.3	30.7
chr7	Wtip	Exon5	N	34904158	34904225	1	51.9	27.3	47.5	26.1
chr10	Pmel	Exon5	N	128152983	128153606	3	88.0	72.6	91.6	70.3
chr1	Arpc2	Exon5	N	74302532	74302625	1	72.7	17.9	49.1	29.3
chr3	Med12l	Exon5	N	58875737	58875847	1	64.5	42.9	62.1	42.7
chr8	Eri1	Exon5	N	36554255	36554433	1	74.9	42.4	73.8	54.3
chr10	Ankrd52	Exon5	N	127817637	127817724	1	73.9	38.6	69.9	50.6
chr7	Ech1	Exon5	N	29615233	29615297	1	91.0	67.1	74.2	55.6
chr2	Mavs	Exon5	N	131070939	131071405	2	82.1	54.5	85.1	66.5
chr7	Bcam	Exon5	N	20345396	20345537	1	20.7	5.4	20.2	1.7
chr9	Layn	Exon5	N	50881953	50882250	1	63.7	41.7	67.7	49.6
chr5	Amz1	Exon5	N	141224569	141224741	1	70.3	43.3	71.2	53.7
chr6	Glcci1	Exon5	N	8532580	8532790	1	77.0	43.4	74.3	57.2
chr11	Slc35b1	Exon5	N	95250394	95250520	1	50.0	19.4	43.9	27.5
chr5	Tmem132d	Exon5	N	128464611	128464794	1	82.8	67.7	69.9	54.8
chr1	Dnah7a	Exon54	N	53692838	53693021	1	61.1	33.3	74.7	9.7
chr5	Plb1	Exon55	N	32657159	32657263	1	89.5	68.0	85.6	54.9
chr12	Snx6	Exon6	N	55864607	55864712	1	83.8	4.9	81.6	9.1
chr13	Aldh5a1	Exon6	N	25015836	25015952	1	74.4	7.2	61.5	2.1
chr9	Pstpip1	Exon6	N	55971069	55971167	1	75.5	20.8	72.2	17.9
chr11	Ttc25	Exon6	N	100415178	100415385	1	49.1	17.0	64.2	13.5
chr15	Gsdmcl-ps	Exon6	N	63753956	63754003	1	72.7	25.0	71.4	20.9
chr2	Mertk	Exon6	N	128584779	128584962	1	96.9	59.1	92.8	44.9
chr5	Fbxo21	Exon6	N	118440812	118440948	1	68.2	33.9	78.1	31.2
chr9	Ifrd2	Exon6	N	107493263	107493441	1	61.6	15.8	66.7	21.2
chr2	Cat	Exon6	N	103307851	103308042	1	74.6	15.1	69.6	25.2
chr14	Ddhd1	Exon6	N	46230167	46230429	1	90.2	46.5	87.2	45.5
chr2	Ehf	Exon6	N	103119675	103119920	1	63.6	20.1	52.5	12.6
chr9	Mns1	Exon6	N	72297259	72297366	1	82.9	14.8	59.0	21.2
chr14	Asb14	Exon6	N	27724483	27724654	1	54.2	31.1	69.8	32.7
chr2	Hsd17b12	Exon6	N	93906566	93906630	1	83.8	43.1	78.4	41.4
chr17	Synj2	Exon6	N	6007938	6008110	1	88.6	67.6	93.8	59.9
chr8	Edc4	Exon6	N	108410219	108410323	1	84.4	64.4	91.7	58.3
chr7	Arrdc4	Exon6	N	75890031	75890097	1	64.6	43.5	65.8	32.5
chr3	Gstm2	Exon6	N	107788952	107789027	1	77.9	18.8	53.3	20.3
chr11	Mtmr3	Exon6	N	4392722	4392917	1	81.3	36.4	77.5	44.9
chr11	Tsr1	Exon6	N	74715511	74715668	1	53.8	27.7	58.2	27.6
chr14	Clu	Exon6	N	66598488	66598717	1	71.2	34.4	71.8	41.2
chr14	Tmem110	Exon6	N	31685736	31685885	1	79.2	28.3	72.4	43.5
chr11	Tmem106a	Exon6	N	101450412	101450455	1	90.5	50.0	100.0	71.4
chr12	Cep128	Exon6	N	92452005	92452197	1	79.0	63.0	82.9	54.3
chr11	Anxa6	Exon6	N	54804893	54804946	1	78.6	59.7	80.7	52.3
chr6	Alox5	Exon6	N	116365392	116365595	1	76.4	56.7	84.1	56.0

chr11	Nprl3	Exon6	N	32141798	32141935	1	77.9	28.8	64.2	37.0
chr13	Arsb	Exon6	N	94709182	94709304	1	21.6	5.4	39.9	12.6
chr1	Stk36	Exon6	N	74657346	74657439	1	33.9	11.5	37.1	10.0
chr5	Uvssa	Exon6	N	33734705	33734833	1	68.6	51.4	69.0	42.2
chr8	Ccdc79	Exon6	N	106996586	106996938	2	28.1	3.2	25.7	0.0
chr7	Tgfb1i1	Exon6	N	135395591	135395764	1	44.6	24.9	45.2	19.6
chr5	Gm17660	Exon6	N	104503724	104503792	1	27.1	3.2	31.8	7.7
chr2	Slc1a2	Exon6	N	102596064	102596297	1	65.7	14.8	68.5	44.6
chr17	Brpf3	Exon6	N	28955281	28955583	2	88.1	45.1	80.4	57.3
chr4	Gale	Exon6	N	135523086	135523171	2	78.4	62.8	79.0	56.7
chr5	Fkbp6	Exon6	N	135823747	135823836	1	83.0	65.0	89.3	67.7
chr7	Adamtsl3	Exon6	N	89614074	89614200	1	42.6	23.0	55.2	33.7
chr1	Idh1	Exon6	N	65217533	65217824	1	93.8	66.7	93.5	72.8
chr10	Specc1l	Exon6	N	74721713	74721888	1	83.0	59.5	80.6	61.0
chr7	Akap13	Exon6	N	82753371	82756446	15	71.9	43.6	61.3	42.0
chr16	Tango2	Exon6	N	18316739	18316827	1	97.1	81.5	80.9	61.9
chr8	Lphn1	Exon6	N	86455512	86455615	1	88.9	64.3	77.3	59.0
chr7	Mki67	Exon6	N	142896226	142896478	1	90.8	70.8	86.0	68.0
chr17	Mlst8	Exon6	N	24615056	24615107	1	44.9	20.0	39.2	21.2
chr17	Dnase1l2	Exon6	N	24579585	24579744	2	95.0	75.0	77.5	60.5
chr19	Rtn3	Exon6	N	7530790	7531950	6	60.1	42.1	71.8	54.9
chr10	Dazap1	Exon6	N	79743674	79743756	1	79.4	52.3	83.7	66.9
chr19	Acta2	Exon6	N	34326220	34326348	1	51.6	17.6	39.1	22.9
chr3	Tars2	Exon6	N	95549956	95550093	1	81.7	60.5	79.6	64.0
chr14	Lgi3	Exon6	N	70934488	70934652	1	83.8	58.1	72.7	57.1
chr14	Vdac2	Exon6	N	22657613	22657840	1	78.4	58.1	66.1	50.7
chr10	Lrp1	Exon65	N	127013496	127013693	1	29.1	9.4	26.9	6.8
chr9	Vps13c	Exon67	N	67809401	67809542	1	75.4	24.6	64.3	37.8
chr4	Cyp2j5	Exon7	N	96329803	96329965	1	63.9	9.5	72.5	3.3
chr5	Nsun5	Exon7	N	135851145	135851355	2	67.5	8.9	65.4	7.5
chr4	Akr1a1	Exon7	N	116318109	116318198	2	76.0	43.7	75.4	35.8
chr11	Hmmr	Exon7	N	40527480	40527694	1	58.6	38.7	70.1	30.7
chr11	Itgae	Exon7	N	72927084	72927235	1	57.0	27.1	55.6	16.8
chr17	Slc37a1	Exon7	N	31459044	31459210	1	53.3	6.0	52.7	15.3
chr7	C2cd3	Exon7	N	107548178	107548325	1	74.3	33.3	72.4	36.8
chr9	Poc1a	Exon7	N	106207596	106207664	1	62.8	16.5	59.1	23.6
chr16	Abcc5	Exon7	N	20364048	20364234	1	84.7	66.7	95.9	61.3
chr3	Setdb1	Exon7	N	95132470	95132639	1	45.9	16.4	55.6	21.8
chr4	Rap1gap	Exon7	N	137272000	137272078	1	41.5	10.6	54.8	21.4
chr6	Crbn	Exon7	N	106744182	106744331	1	85.3	54.7	85.0	53.4
chr18	Hmgxb3	Exon7	N	61306083	61306210	1	80.3	42.4	73.5	44.4
chr8	Rbm34	Exon7	N	129489282	129489522	1	34.1	9.0	38.7	10.1
chr7	Chrdl2	Exon7	N	107177162	107177356	1	50.0	29.2	63.8	35.6
chr10	Tmem194	Exon7	N	127132425	127132598	1	69.6	45.0	69.8	43.1
chr10	Ccdc162	Exon7	N	41273222	41273374	1	16.4	0.0	25.5	0.0
chr18	Wdr33	Exon7	N	32037678	32037804	1	45.8	28.6	69.4	45.2
chr8	Zswim4	Exon7	N	86749715	86750065	2	64.2	47.3	64.8	40.9
chr11	Eml6	Exon7	N	29652436	29652550	1	79.0	55.3	80.7	57.5
chr1	Usf1	Exon7	N	173347604	173347662	1	86.7	66.0	86.0	63.8

chr17	Btnl5	Exon7	N	34632864	34632960	1	73.9	20.5	69.9	48.1
chr17	Unkl	Exon7	N	25347749	25347831	1	90.5	72.1	94.0	73.1
chr10	Ap3d1	Exon7	N	80173434	80173577	1	81.4	62.5	77.6	56.8
chr9	Trim29	Exon7	N	43140393	43140469	1	87.5	71.4	69.8	50.0
chr9	Plscr4	Exon7	N	92384790	92384948	1	89.0	68.0	90.8	71.1
chr6	Phb2	Exon7	N	124665972	124666052	1	75.5	41.1	64.9	45.8
chr15	Krt8	Exon7	N	101831848	101832056	1	39.5	18.1	38.2	19.1
chr3	Gm21949	Exon7	N	68421533	68421730	1	91.1	52.8	78.0	59.0
chr17	Mlst8	Exon7	N	24615265	24615403	2	33.3	10.1	25.0	6.3
chr5	Exoc1	Exon7	N	76972903	76973035	1	73.4	51.4	69.8	51.8
chr2	Prom2	Exon7	N	127356841	127356933	1	81.1	63.6	89.3	71.9
chr11	E230016K23Rik	Exon7	N	83435142	83435307	1	78.0	60.2	76.6	59.4
chr13	Aoah	Exon7	N	21002986	21003056	1	50.4	8.9	47.5	30.7
chr2	Slc1a2	Exon7	N	102601172	102601366	1	90.7	71.4	94.8	79.3
chr15	Scn8a	Exon7	N	100800635	100800698	1	46.8	31.5	35.9	20.4
chr14	Ncoa4	Exon7	N	32989125	32990141	5	80.2	65.1	74.8	59.4
chr19	Cd6	Exon7	N	10870816	10870878	1	90.0	68.8	97.8	82.6
chr1	Gtf3c3	Exon8	N	54476227	54476398	1	78.3	14.3	83.8	11.8
chr3	Fhdc1	Exon8	N	84268194	84268296	1	64.6	10.4	71.3	8.1
chr6	Zyx	Exon8	N	42307312	42307432	1	65.1	20.8	80.1	18.8
chr6	Clcn1	Exon8	N	42249237	42249321	1	77.4	20.0	65.7	5.7
chr3	Sema4a	Exon8	N	88254433	88254549	1	91.0	32.9	89.2	35.0
chr7	Zfp541	Exon8	N	16675146	16675209	1	76.2	60.0	87.5	46.2
chr5	Sdad1	Exon8	N	92721679	92721755	1	60.2	14.7	48.0	13.0
chr5	Kdm2b	Exon8	N	123332411	123332497	1	77.8	25.5	64.2	29.4
chr11	1700023F06Rik	Exon8	N	103063060	103063072	1	38.9	10.2	42.0	7.5
chr6	Ubn2	Exon8	N	38431804	38431922	1	58.7	31.5	64.8	31.9
chr10	Shprh	Exon8	N	10884143	10884793	3	69.8	30.5	71.5	42.5
chr11	Med24	Exon8	N	98571377	98571476	1	78.8	62.2	92.8	64.0
chr19	Fads3	Exon8	N	10130481	10130577	1	87.0	28.4	58.4	32.2
chr6	Sfxn5	Exon8	N	85237044	85237069	1	85.1	63.0	87.4	61.3
chr10	Mcm9	Exon8	N	53335658	53335858	1	86.3	64.1	93.4	68.7
chr10	Sgk1	Exon8	N	21716365	21716477	1	86.3	58.3	85.4	62.3
chr15	Sema5a	Exon8	N	32492246	32492531	1	77.2	60.3	76.1	55.0
chr6	Dysf	Exon8	N	84017448	84017510	1	60.0	34.8	56.5	36.1
chr6	Arhgap25	Exon8	N	87442172	87442259	1	77.8	52.4	76.9	56.5
chr11	Llgl2	Exon8	N	115708903	115708957	1	100.0	79.2	88.2	68.0
chr9	Npsr1	Exon8	N	24117617	24117797	1	76.7	58.0	75.2	56.4
chr10	Mbd6	Exon8	N	126723850	126724012	1	43.2	10.0	27.2	8.9
chr16	Dnaja3	Exon8	N	4701169	4701284	1	92.6	63.0	80.3	64.2
chr12	Rad51b	Exon8	N	80868058	80868161	1	50.0	22.6	39.5	23.9
chr15	Oc90	Exon9	N	65723914	65724026	1	82.6	4.3	72.0	0.0
chr19	Fads3	Exon9	N	10130707	10130786	1	93.8	28.6	92.4	22.0
chr14	Cpne6	Exon9	N	56134056	56134115	1	76.5	36.0	70.8	9.1
chr6	Clcn1	Exon9	N	42249499	42249600	1	84.7	27.6	82.8	30.1
chr5	Fbxl13	Exon9	N	21049478	21049607	2	61.9	2.9	53.2	7.7
chr11	Pold2	Exon9	N	5776716	5776992	2	70.1	21.1	64.9	23.4
chr12	Numb	Exon9	N	85183168	85183308	2	72.8	40.8	82.9	44.2
chr15	Parp10	Exon9	N	76073494	76073672	2	30.5	12.2	45.1	7.9

chr19	Plce1	Exon9	N	38796302	38796410	1	53.4	8.0	47.4	11.8
chr2	Rapgef4	Exon9	N	72017977	72018160	1	90.8	44.8	84.7	50.7
chr7	Ano1	Exon9	N	151797519	151797720	1	85.9	55.3	81.4	48.1
chr1	Zranb3	Exon9	N	129884574	129884723	1	90.6	56.7	92.5	61.4
chr19	Cnnm1	Exon9	N	43568200	43568301	1	37.0	18.6	37.9	8.1
chr14	Zswim8	Exon9	N	21533105	21533790	4	87.3	37.0	77.4	50.0
chr2	Dnmt3b	Exon9	N	153496039	153496183	1	79.5	54.4	80.1	54.3
chr2	Ppp1r16b	Exon9	N	158582874	158583042	1	51.4	29.4	48.3	23.6
chr9	Gramd2	Exon9	N	59563730	59563834	1	93.6	76.7	88.9	67.7
chr13	Rnf44	Exon9	N	54785699	54785849	2	66.7	40.0	52.2	31.8
chr4	Srrm1	Exon9	N	134893719	134893993	1	26.4	6.9	23.4	5.0
chr7	Kat8	Exon9	N	135068623	135068777	2	64.1	48.4	56.5	39.0
chr2	Fam65c	Exon9	N	167814754	167815305	3	93.8	56.1	71.4	54.2
chr10	Metap2	Exon9	N	93349739	93349846	1	84.5	64.6	87.7	71.4
chr3	Tars2	Exon9	N	95551965	95552063	1	80.6	52.0	64.7	48.4
chr9	Megf11	Exon9	N	64508147	64508321	1	45.9	26.5	44.0	28.2
chr16	Lsg1	Exon9	N	30581045	30581131	1	85.4	66.0	82.6	67.3
chr13	Shc3	Exon9	N	51578251	51578314	1	47.5	7.6	33.0	17.8
chr17	Catsperd	Exon9	N	56791218	56791313	1	44.2	23.3	27.1	12.0
chr1	Dst	Exon90	N	34347204	34347392	1	62.1	30.8	66.3	25.2
chr14	Mtrf1	Intron1	N	79797844	79799498	9	53.0	3.0	86.7	1.8
chr9	Casp1	Intron1	N	5298724	5299279	3	85.9	2.7	80.8	1.3
chr12	Iah1	Intron1	N	21322374	21323236	2	47.5	7.4	64.2	10.9
chr8	Ces2a	Intron1	N	107258048	107258596	3	75.7	9.8	62.7	14.1
chr2	Mtch2	Intron1	N	90687543	90689527	7	70.5	44.2	91.4	45.7
chr3	Gstm3	Intron1	N	107767215	107769004	9	56.9	26.9	69.1	23.7
chr16	Ephb3	Intron1	N	21205337	21212962	33	49.7	15.1	57.3	13.5
chr19	Mrpl21	Intron1	N	3283172	3284759	7	49.7	34.1	52.3	9.7
chr1	Ctla4	Intron1	N	60966124	60969266	16	87.5	61.5	83.5	42.2
chr16	Gm10791	Intron1	N	84972998	84973792	4	56.3	32.4	68.5	27.3
chr8	Irf8	Intron1	N	123260310	123263716	15	52.5	21.6	55.5	15.1
chr17	Prss32	Intron1	N	23990894	23991345	2	63.4	23.3	61.0	21.0
chr1	Pigr	Intron1	N	132723385	132730949	39	57.3	19.0	60.4	21.0
chr9	Ccdc13	Intron1	N	121707358	121708143	4	80.9	45.5	39.1	0.0
chr3	4932438A13Rik	Intron1	N	36762271	36763308	4	50.3	7.5	40.9	1.9
chr7	Nlrp6	Intron1	N	148106986	148107539	2	49.5	0.9	39.4	0.6
chr6	Slc6a12	Intron1	N	121297016	121297420	2	48.3	32.8	57.1	18.5
chr19	Zdhhc16	Intron1	N	42008156	42009227	5	51.4	31.0	38.6	0.0
chr17	Ppp2r1a	Intron1	N	21082548	21088306	25	72.9	53.5	90.0	52.7
chr7	Epn1	Intron1	N	5032080	5033619	5	75.0	23.0	57.0	20.0
chr18	Slc12a2	Intron1	N	58039199	58055935	79	70.7	31.5	70.6	34.1
chr5	Il6	Intron1	N	30339751	30339915	1	41.4	1.7	43.7	8.0
chr11	Cox11	Intron1	N	90499867	90501690	7	54.8	36.4	91.8	56.3
chr2	Nkain4	Intron1	N	180669735	180671855	12	79.4	39.3	74.8	39.7
chr11	Uqcr10	Intron1	N	4602224	4604159	8	37.0	4.3	53.9	19.2
chr2	Ptpmt1	Intron1	N	90751477	90754140	13	80.0	44.4	75.3	41.4
chr17	Rgmb	Intron1	N	15944766	15957633	63	66.7	36.3	72.6	38.8
chr4	Gm13177	Intron1	N	144203778	144207726	20	83.5	43.7	73.0	40.1
chr9	Ttc21a	Intron1	N	119846869	119848284	6	62.5	38.8	74.8	42.1

chr5	Nudt9	Intron1	N	104476166	104479596	15	63.1	46.6	94.1	61.8
chr19	Pdzd7	Intron1	N	45102465	45102868	2	57.5	36.8	57.7	25.3
chr16	Col8a1	Intron1	N	57628931	57632423	15	71.2	48.7	79.4	47.1
chr11	4930502E09Rik	Intron1	N	84642915	84643403	2	91.6	59.7	83.7	52.6
chr5	Hadha	Intron1	N	30446501	30446584	1	73.5	50.0	63.4	32.6
chr3	Nhlrc3	Intron1	N	53256572	53257463	4	83.7	38.7	58.8	28.3
chr3	Adh1	Intron1	N	137940701	137942694	10	48.8	33.6	78.2	47.8
chr19	Slc35g1	Intron1	N	38470792	38474923	18	57.8	30.5	46.3	16.4
chr10	Mettl7b	Intron1	N	128396012	128397496	8	69.5	13.9	55.6	26.3
chr9	Ubl7	Intron1	N	57758867	57760444	7	81.4	64.6	68.2	39.3
chr19	Nhlrc2	Intron1	N	56623351	56626248	14	57.5	26.8	42.0	13.6
chr11	Mgat4b	Intron1	N	50039242	50044181	22	47.0	20.0	40.9	12.8
chr12	Gpx2	Intron1	N	77893988	77896140	11	23.2	0.8	28.6	0.8
chr8	B930018H19Rik	Intron1	N	35653640	35654811	6	60.9	34.4	66.4	38.7
chr5	Vsig10	Intron1	N	117769513	117773508	17	29.8	8.2	59.7	32.5
chr7	Pnpla2	Intron1	N	148641373	148643171	7	77.5	27.8	55.7	28.7
chr2	Myh7b	Intron1	N	155436998	155437322	2	82.5	56.6	80.5	53.7
chr16	Gm933	Intron1	N	32805131	32806285	6	58.4	26.1	67.4	40.9
chr3	Gstm1	Intron1	N	107815697	107817670	10	61.8	25.6	73.3	46.9
chr11	Rnft1	Intron1	N	86298302	86299562	4	48.8	28.5	34.4	8.3
chr7	Ebf3	Intron1	N	144388086	144390174	10	88.2	62.6	73.0	47.2
chr7	Kcnn4	Intron1	N	25155615	25159690	20	32.5	11.7	34.3	8.5
chr17	Cyp4f40	Intron1	N	32796629	32799062	12	68.7	40.2	64.2	38.5
chr10	Adat3	Intron1	N	80065695	80069058	14	49.8	15.1	57.3	31.7
chr15	Col2a1	Intron1	N	97806618	97807072	2	35.4	19.1	64.1	38.7
chr4	Hook1	Intron1	N	95634248	95641076	31	89.5	68.2	85.5	60.2
chr7	Pglyrp1	Intron1	N	19470323	19474626	22	66.6	38.4	65.9	40.7
chr8	Nqo1	Intron1	N	109913025	109914944	10	58.5	36.2	71.0	46.2
chr7	Dmbt1	Intron1	N	138175660	138178194	13	59.0	17.4	44.3	19.8
chr2	Golga2	Intron1	N	32143917	32147612	16	74.5	56.4	84.4	59.9
chr3	Rbm15	Intron1	N	107129223	107133132	17	73.3	30.0	55.0	30.6
chr7	Psenen	Intron1	N	31347154	31347342	1	54.3	39.3	60.0	35.7
chr17	Wash	Intron1	N	66460990	66463202	9	75.9	57.4	90.6	66.3
chr2	Zc3h15	Intron1	N	83484885	83493514	38	33.9	11.8	46.0	21.8
chr5	Cldn15	Intron1	N	137444194	137448189	20	82.7	46.0	77.7	53.5
chr17	Tmem8	Intron1	N	26250513	26253703	13	28.8	3.3	34.7	10.5
chr13	Cenpk	Intron1	N	105019083	105020808	6	31.4	8.3	31.8	7.7
chr12	Ddx1	Intron1	N	13226435	13227152	4	29.2	0.0	71.7	47.6
chr1	Ccl20	Intron1	N	83113478	83114373	5	53.0	22.7	27.1	3.0
chr11	Hoxb9	Intron1	N	96133373	96135937	7	53.5	35.1	52.5	28.4
chr18	1700065O20Rik	Intron1	N	49963182	49965434	10	72.1	55.0	79.0	55.0
chr11	Slc25a35	Intron1	N	68782540	68784225	7	93.1	56.5	90.6	66.7
chr3	Hmgcs2	Intron1	N	98084511	98094809	50	73.1	25.0	67.8	43.8
chr3	Alpk1	Intron1	N	127374050	127375277	6	74.3	57.1	72.4	48.6
chr2	Dpp4	Intron1	N	62170777	62172312	9	91.3	73.6	91.6	68.0
chr2	Agpat2	Intron1	N	26449283	26450299	5	37.8	14.8	36.6	13.1
chr11	LOC666331	Intron1	N	101472224	101472405	1	57.7	25.0	47.1	23.5
chr4	Stmn1	Intron1	N	134024345	134026017	3	56.1	14.6	40.3	17.0
chr11	Krt31	Intron1	N	99908375	99908982	3	46.9	25.0	40.3	17.1

chr11	Hoxb7	Intron1	N	96148442	96150690	8	56.6	24.1	52.6	29.4
chr6	Tpi1	Intron1	N	124761406	124761534	2	65.3	39.8	64.7	41.6
chr6	Herc6	Intron1	N	57531227	57533166	9	45.3	26.8	50.9	27.9
chr11	Engase	Intron1	N	118338419	118340196	6	46.3	23.3	41.9	18.9
chr7	Slc1a5	Intron1	N	17367825	17371024	15	17.2	0.3	24.5	1.6
chr4	AU040320	Intron1	N	126430907	126434142	13	51.9	34.8	46.0	23.2
chr5	Epgn	Intron1	N	91456592	91457325	3	27.9	10.0	26.1	3.3
chr15	0610007N19Rik	Intron1	N	32171525	32174136	13	34.9	9.5	38.8	16.1
chr6	Tfpi2	Intron1	N	3913448	3913805	2	24.5	2.4	24.7	2.1
chr12	Degs2	Intron1	N	109927298	109930103	15	30.3	10.6	32.6	10.0
chr14	Styx	Intron1	N	45971205	45974620	15	81.9	56.8	77.6	55.1
chr15	Klhl38	Intron1	N	58146672	58147781	6	88.7	59.4	67.5	45.0
chr10	Sumo3	Intron1	N	77069087	77072556	13	81.4	62.4	81.8	59.3
chr11	Abca9	Intron1	N	109963378	109963824	2	83.2	53.6	90.3	67.8
chr11	Sp2	Intron1	N	96815885	96817088	6	64.3	43.1	69.3	46.9
chr15	E430025E21Rik	Intron1	N	59163915	59165172	6	62.1	17.4	57.3	35.0
chr19	Gm10814	Intron1	N	6012685	6014228	10	92.4	71.1	74.8	52.7
chr2	Ada	Intron1	N	163552785	163553279	3	47.2	2.0	43.7	21.6
chr14	Rnase1	Intron1	N	51765595	51766390	4	73.1	25.7	88.1	66.4
chr2	Cbln4	Intron1	N	171863068	171864473	7	93.5	71.0	59.8	38.2
chr4	Nbl1	Intron1	N	138639518	138641155	8	75.3	49.7	78.9	57.4
chr3	4931419H13Rik	Intron1	N	54859359	54862196	12	77.8	59.1	57.3	35.9
chr10	Ano4	Intron1	N	88412528	88414934	12	46.4	25.6	43.5	22.2
chr2	Hnf4a	Intron1	N	163373156	163377312	21	47.1	23.8	42.5	21.3
chr4	Aldh1b1	Intron1	N	45812042	45815326	15	33.8	6.7	32.3	11.5
chr15	Gm20556	Intron1	N	84545570	84549126	18	91.8	67.6	73.2	52.3
chr2	Slmo2	Intron1	N	174291438	174292226	4	86.5	57.3	73.3	52.5
chr7	Orai3	Intron1	N	134913731	134917070	16	50.5	29.7	51.1	30.4
chr10	1700040L02Rik	Intron1	N	67894072	67899027	25	69.0	51.9	67.7	46.9
chr1	Aox3	Intron1	N	58170157	58171714	8	75.7	45.7	74.6	54.0
chr8	Zfp617	Intron1	N	74446872	74452100	22	98.7	75.8	90.8	70.3
chr11	Slc16a13	Intron1	N	70031424	70032094	3	35.8	1.2	26.4	6.0
chr3	P2ry1	Intron1	N	60808522	60810820	12	73.9	17.7	67.4	47.1
chr2	Sord	Intron1	N	122060695	122072217	56	64.9	43.1	61.6	41.3
chr7	Zg16	Intron1	N	134194114	134195140	5	78.0	56.5	77.9	57.7
chr2	Itga6	Intron1	N	71625378	71654686	142	60.5	45.4	61.2	41.0
chr6	Zfp282	Intron1	N	47827836	47829998	7	34.0	5.7	34.5	14.4
chr2	Acss2	Intron1	N	155344021	155347716	16	62.1	44.6	48.4	28.4
chr2	Ahcy	Intron1	N	154886317	154887884	8	96.1	76.6	82.4	62.5
chr6	Clcn1	Intron1	N	42236946	42240071	16	80.8	63.0	85.1	65.2
chr7	Taldo1	Intron1	N	148578218	148581947	17	58.2	32.1	61.4	41.5
chr17	Tbl3	Intron1	N	24837731	24837820	2	76.8	55.8	81.4	61.6
chr9	Fbxl22	Intron1	N	66359826	66362025	7	47.6	14.3	39.1	19.4
chr6	Akr1b7	Intron1	N	34362476	34365319	14	88.2	64.6	87.3	67.6
chr9	Gm684	Intron1	N	51079031	51080432	7	69.0	28.9	45.1	25.5
chr1	Ivns1abp	Intron1	N	153191886	153196505	17	43.4	3.8	24.9	5.4
chr11	B3gntl1	Intron1	N	121479167	121480948	9	81.9	65.9	86.8	67.4
chr5	Ddx55	Intron1	N	125003049	125004551	6	80.4	62.9	77.0	57.7
chr13	Akr1c18	Intron1	N	4132083	4134448	11	84.7	35.0	74.2	55.0

chr14	Mat1a	Intron1	N	41918550	41918635	1	69.2	37.8	64.0	44.8
chr15	Eppk1	Intron1	N	75943156	75949538	31	62.8	44.9	60.9	41.7
chr7	Kctd14	Intron1	N	104600057	104601821	9	59.3	40.7	50.5	31.4
chr11	4932414J04Rik	Intron1	N	21395128	21397043	10	87.8	59.6	74.9	55.9
chr7	Ifitm5	Intron1	N	148135348	148135916	3	64.0	47.2	69.7	50.7
chr6	Tmem43	Intron1	N	91423893	91427238	15	95.6	39.6	73.6	54.5
chr3	Chmp4c	Intron1	N	10367222	10385551	91	52.7	35.6	58.6	39.7
chr6	Mgst1	Intron1	N	138089128	138096179	36	57.7	29.5	52.9	33.9
chr3	Ctss	Intron1	N	95330848	95333355	13	84.6	64.3	81.2	62.3
chr17	Ly6g6c	Intron1	N	35204390	35205811	7	49.4	33.8	54.4	35.7
chr10	Rdh1	Intron1	N	127197307	127200020	14	90.1	66.9	89.9	71.3
chr6	Fam136a	Intron1	N	86315814	86316603	1	46.0	27.1	37.8	19.4
chr1	Neu2	Intron1	N	89470702	89476008	27	59.7	42.4	51.4	32.9
chr7	Picalm	Intron1	N	97279213	97308957	138	47.6	29.7	51.9	33.5
chr1	Pikfyve	Intron1	N	65233408	65236892	15	33.6	0.0	38.1	19.8
chr7	Mesdc2	Intron1	N	91040986	91044039	13	76.8	55.5	63.1	44.9
chr7	E430018J23Rik	Intron1	N	134536030	134536514	2	72.7	55.6	66.7	48.5
chr1	Acsl3	Intron1	N	78654679	78659715	23	31.3	15.6	35.4	17.4
chr7	Tmem86a	Intron1	N	54306172	54308219	8	43.9	23.9	36.1	18.2
chr18	Gm9926	Intron1	N	66664117	66664963	4	87.6	63.1	81.1	63.2
chr11	Pgam2	Intron1	N	5701843	5703001	6	54.4	31.1	40.1	22.2
chr4	Hmgcl	Intron1	N	135502456	135506464	16	70.9	52.6	66.9	49.1
chr11	Trim11	Intron1	N	58792147	58794742	10	61.7	45.8	58.9	41.2
chr10	Amdhd1	Intron1	N	92987248	92988590	7	88.7	66.0	85.0	67.3
chr13	Glrx	Intron1	N	75977624	75984617	35	50.8	30.4	43.2	25.5
chr5	Atp6v0a2	Intron1	N	125079872	125083459	15	72.3	45.2	57.1	39.6
chr16	Vgll3	Intron1	N	65816004	65828138	57	77.6	56.1	70.2	52.7
chr4	Rnf220	Intron1	N	116944981	116945657	3	27.5	2.2	26.4	8.9
chr2	Lrrc8a	Intron1	N	30093326	30096088	10	47.1	24.3	40.2	22.9
chr7	Fam83e	Intron1	N	52978025	52978810	4	28.8	4.9	21.5	4.2
chr3	Mgst2	Intron1	N	51465229	51468432	16	44.2	24.6	34.0	16.8
chr10	Lrig3	Intron1	N	125403768	125409176	24	56.2	25.5	35.2	18.0
chr3	Polr3gl	Intron1	N	96382512	96383720	6	92.9	63.7	76.8	59.7
chr4	Hdhd3	Intron1	N	62161128	62163191	9	71.0	49.3	50.3	33.2
chr2	Ndor1	Intron1	N	25101670	25101851	2	66.0	25.0	73.5	56.6
chr6	Aqp1	Intron1	N	55286869	55295399	43	63.6	43.0	59.1	42.2
chr2	Pdss1	Intron1	N	22751283	22756800	25	94.0	76.5	92.2	75.4
chr4	Gm438	Intron1	N	144368079	144369618	8	75.8	60.6	78.8	62.1
chr13	2310005E17Rik	Intron1	N	99194872	99199004	21	50.3	31.9	63.8	47.2
chr3	Gm15441	Intron1	N	96359984	96361208	6	31.7	0.0	45.7	29.1
chr5	Brca2	Intron1	N	151325472	151325749	1	30.0	10.4	32.9	16.3
chr11	2810442I21Rik	Intron1	N	16839651	16850884	56	84.8	65.2	84.8	68.2
chr10	Cand1	Intron1	N	118640262	118643771	18	72.8	47.4	62.7	46.3
chr3	Dennd2d	Intron1	N	106285632	106289790	19	23.2	7.5	21.3	5.0
chr13	Akr1c13	Intron1	N	4190557	4193279	13	51.0	10.4	22.1	5.8
chr2	Emc7	Intron1	N	112295458	112299616	17	96.8	26.1	80.8	64.5
chr10	Thop1	Intron1	N	80533024	80535897	13	76.9	46.6	78.2	61.9
chr1	Atp1a2	Intron1	N	174204861	174206048	6	84.0	64.0	89.4	73.2
chr8	Amfr	Intron1	N	96497215	96497958	4	90.7	62.1	86.1	70.0

chr10	Dna2	Intron1	N	62409918	62411930	8	80.9	48.6	75.4	59.3
chr6	Suclg1	Intron1	N	73198748	73206164	34	45.6	22.3	41.7	25.7
chr2	Hoxd10	Intron1	N	74530782	74532147	5	53.0	24.1	56.6	40.7
chr10	Socs2	Intron1	N	94875745	94877464	5	29.3	4.2	34.5	18.6
chr7	Cd81	Intron1	N	150238972	150248397	44	71.5	45.6	65.9	50.0
chr5	4930519H02Rik	Intron1	N	15369871	15373088	15	48.3	30.1	63.6	47.8
chr7	Fan1	Intron1	N	71491782	71493733	10	93.2	69.3	84.7	68.8
chr9	Taf1d	Intron1	N	15110728	15112171	5	57.3	37.5	60.2	44.3
chr5	Pgm1	Intron1	N	64484328	64488217	17	62.5	41.9	58.5	42.6
chr19	E030003E18Rik	Intron1	N	20567312	20567884	3	77.3	58.4	80.3	64.6
chr15	Bin2	Intron1	N	100471996	100475243	16	75.8	56.3	80.4	64.8
chr10	Oaz1	Intron1	N	80289651	80290981	4	31.9	5.6	30.5	14.8
chr3	Rtca	Intron1	N	116192257	116195900	18	81.5	64.1	82.1	66.6
chr14	1700092C10Rik	Intron1	N	69783075	69783820	4	68.6	48.3	70.8	55.3
chr3	Impa1	Intron1	N	10315302	10316102	4	42.4	26.4	36.9	21.6
chr6	Gm19434	Intron1	N	135138614	135138852	1	43.8	26.7	55.2	40.0
chr1	Elf3	Intron1	N	137150958	137151501	3	27.0	1.8	16.8	1.6
chr10	Syne1	Intron1	N	5152069	5153368	5	38.6	2.9	20.7	5.6
chr14	Pnp2	Intron1	N	51575904	51579189	16	63.9	48.0	69.3	54.2
chr4	Zdhhc18	Intron1	N	133164689	133166284	8	50.1	34.7	49.3	34.2
chr3	Clca4	Intron10	N	144507590	144509325	9	80.6	25.2	81.6	20.9
chr4	Cachd1	Intron10	N	100638940	100639550	3	65.5	19.6	66.3	11.4
chr14	Tmtc4	Intron10	N	123342580	123343895	7	91.7	63.8	67.1	18.0
chr12	Pum2	Intron10	N	8730986	8735439	20	95.6	56.3	75.9	27.6
chr10	D10Wsu52e	Intron10	N	85416250	85418463	11	70.0	29.9	77.3	40.0
chr4	Acap3	Intron10	N	155274520	155275776	6	87.3	53.6	74.9	39.1
chr15	BC024139	Intron10	N	75955212	75955491	1	45.5	7.7	42.4	7.5
chr18	Ss18	Intron10	N	14837973	14841175	11	78.6	38.3	89.3	55.9
chr9	Tinag	Intron10	N	76880297	76893155	59	83.2	65.1	80.7	52.6
chr1	Hspd1	Intron10	N	55141630	55143609	10	43.4	20.5	41.5	14.0
chr5	Dcun1d4	Intron10	N	73946703	73948492	9	81.1	57.0	86.1	58.6
chr19	Zp1	Intron10	N	10993556	10993869	2	80.7	57.5	70.2	43.0
chr7	Itgad	Intron10	N	135329565	135329744	1	44.7	26.6	44.8	18.9
chr7	Cblc	Intron10	N	20378015	20378157	1	87.1	69.4	89.6	64.0
chr2	Abcb11	Intron10	N	69112217	69115827	16	82.1	50.5	74.8	49.1
chr1	Tmbim1	Intron10	N	74340577	74341771	6	68.9	33.9	69.2	43.9
chr18	Bin1	Intron10	N	32581615	32584473	14	90.8	34.4	78.7	53.7
chr10	Creb3l3	Intron10	N	80555403	80555551	2	37.7	12.1	36.9	12.2
chr17	Slc22a1	Intron10	N	12860197	12868111	39	61.2	38.1	70.9	46.8
chr9	Prtg	Intron10	N	72704888	72706567	8	97.5	63.1	91.9	68.2
chr5	Tapt1	Intron10	N	44584562	44585486	5	89.1	73.6	73.3	49.6
chr9	Amica1	Intron10	N	44912357	44915461	17	84.6	40.0	84.1	61.3
chr1	Vps4b	Intron10	N	108688367	108692997	21	77.3	36.3	63.9	41.4
chr2	Osbpl2	Intron10	N	179888028	179889971	9	87.4	69.6	73.5	51.9
chr1	Plcd4	Intron10	N	74607068	74608628	9	92.6	73.8	85.2	64.3
chr2	F2	Intron10	N	91473454	91475055	8	61.0	43.7	45.9	25.1
chr11	Osbp2	Intron10	N	3617218	3617796	3	52.8	34.5	62.9	42.9
chr13	Aoah	Intron10	N	21006870	21008928	10	90.4	61.5	79.7	60.6
chr11	Luc7l3	Intron10	N	94171006	94183003	57	66.0	45.7	58.2	39.5

chr7	Relb	Intron10	N	20213320	20214539	2	42.6	21.7	38.7	20.1
chr10	Ank3	Intron10	N	69333948	69337537	17	55.4	38.1	58.6	40.3
chr8	Smarca5	Intron10	N	83235705	83237832	11	94.3	77.5	92.4	74.3
chr2	Lnp	Intron10	N	74407186	74408991	8	63.0	32.1	56.6	38.6
chr19	Cep78	Intron10	N	16050853	16053270	12	93.1	77.1	89.4	71.9
chr15	Oxr1	Intron10	N	41655042	41657435	11	95.7	75.2	89.7	72.3
chr5	Fbxl13	Intron10	N	21049608	21057910	42	78.1	60.6	77.6	61.4
chr9	Tex9	Intron10	N	72335883	72339545	18	72.4	33.2	55.7	39.7
chr4	Ikbkap	Intron10	N	56782088	56783852	9	87.8	65.4	83.3	68.0
chr1	Lgr6	Intron10	N	136899849	136900034	1	50.0	17.4	52.0	36.7
chr5	Trafd1	Intron11	N	121834114	121835533	4	78.7	38.9	73.0	2.5
chr1	Atg16l1	Intron11	N	89675603	89676699	5	61.1	15.3	45.0	1.5
chr11	Eif4enif1	Intron11	N	3134649	3135583	5	79.4	33.0	73.3	35.3
chr8	Ces1f	Intron11	N	95795864	95798011	11	80.7	43.6	78.3	43.9
chr9	Arih1	Intron11	N	59274514	59284577	46	90.1	74.3	82.7	50.4
chr4	Dnaic1	Intron11	N	41561381	41561682	2	78.3	50.0	58.5	27.7
chr11	Sec14l2	Intron11	N	4016760	4018627	7	65.4	44.7	55.0	25.4
chr8	Tmem161a	Intron11	N	72705426	72705781	2	63.0	26.2	59.7	30.4
chr5	Dcun1d4	Intron11	N	73948596	73948870	1	26.7	10.2	40.5	11.8
chr2	Duox2	Intron11	N	122111076	122112218	6	74.2	44.3	62.2	34.8
chr5	Cdk8	Intron11	N	147111153	147111267	1	67.7	50.7	77.4	50.0
chr12	Zfyve1	Intron11	N	84936273	84937946	7	46.5	18.1	44.2	17.6
chr14	Atxn7	Intron11	N	14933416	14935820	12	71.5	55.0	70.7	45.1
chr14	Ercc6	Intron11	N	33374012	33375741	9	92.6	62.0	86.8	61.3
chr7	Trpm4	Intron11	N	52570464	52572144	8	89.3	70.0	78.9	55.4
chr8	Ces1b	Intron11	N	95597244	95599957	14	85.0	67.4	85.2	62.5
chr4	Nfib	Intron11	N	82144674	82150634	25	69.2	47.9	84.6	63.0
chr9	Dapk2	Intron11	N	66116758	66119433	13	88.1	51.1	68.9	47.7
chr11	Nf1	Intron11	N	79226261	79232053	25	79.8	51.8	76.6	55.5
chr19	Abcc2	Intron11	N	43883206	43884960	9	91.2	71.4	53.3	32.2
chr14	Rnf31	Intron11	N	56215696	56217474	8	86.4	62.8	92.4	71.4
chr7	Zfp541	Intron11	N	16676261	16677634	7	70.7	25.9	67.3	46.5
chr7	Eed	Intron11	N	97125560	97128921	13	70.5	44.5	48.6	27.8
chr11	Tbrg4	Intron11	N	6524287	6525151	5	42.4	19.6	47.2	26.5
chr17	Plg	Intron11	N	12596099	12596878	4	79.7	50.0	66.2	45.8
chr6	Chn2	Intron11	N	54245916	54248038	11	100.0	27.7	88.5	68.8
chr19	Lrp5	Intron11	N	3610239	3612197	10	75.6	59.6	79.6	59.9
chr7	Rhpn2	Intron11	N	36166551	36168987	12	80.2	42.9	65.4	46.4
chr16	Tnk2	Intron11	N	32678072	32678754	3	75.4	41.6	64.3	46.1
chr7	Gltscr1	Intron11	N	16578495	16581749	16	75.7	40.1	69.0	51.6
chr5	Smarcd3	Intron11	N	24102668	24104409	7	68.9	49.5	52.9	35.7
chr14	Dock5	Intron11	N	68389828	68391108	6	98.2	78.4	80.3	63.2
chr11	Itk	Intron11	N	46167445	46169226	8	97.4	81.4	96.1	79.3
chr10	Enpp1	Intron11	N	24377039	24377644	3	28.7	7.1	29.1	12.8
chr15	St13	Intron11	N	81222753	81229914	34	63.6	41.3	54.1	38.0
chr9	Sema3f	Intron12	N	107590681	107592016	7	72.6	34.3	72.7	11.3
chr15	Plec	Intron12	N	76016945	76017040	1	80.0	12.5	71.5	13.6
chr18	Nars	Intron12	N	64671532	64671637	1	55.0	20.0	76.6	23.4
chr11	Ankrd13b	Intron12	N	77290248	77290792	3	39.7	13.9	69.3	16.2

chr2	Ggt7	Intron12	N	155330558	155331690	6	70.3	17.0	63.0	10.9
chr11	Scpep1	Intron12	N	88813869	88816607	13	85.3	54.4	89.9	46.0
chr3	Ilf2	Intron12	N	90291020	90291148	2	88.6	58.3	92.3	48.5
chr9	Plxnb1	Intron12	N	109008228	109008429	1	64.4	40.4	74.3	32.0
chr16	Ccdc14	Intron12	N	34721956	34723095	6	87.7	67.9	87.0	49.4
chr15	Baiap2l2	Intron12	N	79114524	79115004	2	50.8	14.2	53.0	15.6
chr1	Adhfe1	Intron12	N	9553975	9556861	14	49.4	19.8	51.9	19.0
chr9	Col6a5	Intron12	N	105794635	105797571	15	95.7	63.5	94.7	65.6
chr19	Eml3	Intron12	N	9011251	9011618	2	72.2	51.4	70.2	42.5
chr6	Mug-ps1	Intron12	N	122168735	122170878	11	69.7	33.0	59.0	31.4
chr8	Tpte	Intron12	N	23431394	23435967	23	95.7	63.5	90.5	64.3
chr9	Gk5	Intron12	N	96071974	96075108	15	75.3	54.9	79.7	53.7
chr7	Zdhhc13	Intron12	N	56071853	56072515	3	92.9	55.2	77.6	52.0
chr11	Tbrg4	Intron12	N	6525217	6525967	3	32.4	3.7	29.0	3.4
chr3	Wdr47	Intron12	N	108440982	108441543	3	97.0	76.6	83.8	59.6
chr18	Riok3	Intron12	N	12312530	12313728	6	74.4	52.4	84.2	60.2
chr15	Kcnh3	Intron12	N	99070358	99071317	5	86.1	71.0	86.6	63.3
chr15	Slc38a2	Intron12	N	96525941	96528352	12	42.9	25.3	39.5	16.2
chr10	Pcbp3	Intron12	N	76262298	76263504	6	94.6	77.5	84.9	61.9
chr5	Rfc1	Intron12	N	65670063	65670695	3	75.3	58.9	72.7	50.7
chr5	Radil	Intron12	N	142983043	142984023	5	50.4	4.0	66.6	44.6
chr9	Pfkfb4	Intron12	N	108930322	108931407	5	78.8	56.5	59.8	39.9
chr8	F11	Intron12	N	46338483	46340557	10	50.5	0.0	17.2	0.0
chr11	Dock2	Intron12	N	34154473	34156526	10	88.1	73.0	81.2	64.1
chr1	Npas2	Intron12	N	39387867	39390654	14	67.5	34.2	63.8	46.9
chr2	Tgm2	Intron12	N	157968911	157972026	13	80.4	59.0	79.2	62.5
chr4	Necab1	Intron12	N	15067397	15075964	42	53.3	33.2	55.5	39.4
chr5	Ccdc63	Intron12	N	122584903	122587872	16	75.6	55.9	74.9	59.7
chr1	Rfx8	Intron12	N	39767090	39768222	6	82.3	61.7	86.0	70.9
chr3	Clca4	Intron13	N	144510268	144512090	9	76.5	30.9	83.5	11.4
chr3	Clca1	Intron13	N	144422276	144423527	6	75.0	47.4	96.7	44.1
chr3	Ilf2	Intron13	N	90291240	90291380	2	88.6	58.3	92.3	48.5
chr11	Rap1gap2	Intron13	N	74228023	74229733	9	69.3	7.8	53.1	18.0
chr4	Fkbp15	Intron13	N	61985256	61987092	9	51.4	4.9	43.2	9.1
chr13	Gpr98	Intron13	N	81536574	81544162	38	87.4	63.7	86.5	54.3
chr2	Slc23a2	Intron13	N	131917066	131919877	14	85.7	43.3	60.5	28.5
chr7	Cd22	Intron13	N	31663127	31664213	6	66.7	45.2	82.4	53.2
chr4	Slc5a9	Intron13	N	111568958	111571154	11	87.7	41.5	70.0	41.6
chr17	Msln	Intron13	N	25890008	25890322	2	70.0	42.9	79.2	52.0
chr14	Cacna1d	Intron13	N	30888443	30888997	3	93.8	73.3	83.5	56.4
chr2	Cubn	Intron13	N	13236437	13239783	16	100.0	70.7	65.0	38.3
chr2	Kif16b	Intron13	N	142583685	142584525	4	76.5	43.6	60.4	35.2
chr8	Kat6a	Intron13	N	24036943	24039687	14	90.2	67.0	86.2	61.1
chr11	Nf2	Intron13	N	4716169	4718507	12	47.8	27.8	66.2	41.5
chr15	Baiap2l2	Intron13	N	79115081	79115767	4	38.9	20.0	41.8	18.0
chr16	Tmprss2	Intron13	N	97820930	97832625	55	53.6	36.6	50.5	27.8
chr10	Aim1	Intron13	N	43696640	43699286	13	43.9	20.5	41.7	20.1
chr1	Atic	Intron13	N	71622708	71623190	2	83.1	61.4	86.4	65.5
chr9	Ireb2	Intron13	N	54745430	54747194	9	63.7	40.6	53.1	33.1

chr2	Usp6nl	Intron13	N	6348858	6351471	13	84.0	62.5	78.7	59.3
chr15	Wdr67	Intron13	N	57783368	57784310	5	72.6	53.0	65.6	46.4
chr2	Fermt1	Intron13	N	132765252	132767672	11	85.2	68.6	76.4	57.4
chr17	Nrxn1	Intron13	N	91022964	91029224	31	93.2	33.3	81.3	64.4
chr7	Cln3	Intron13	N	133725257	133726277	5	59.0	27.9	58.1	41.2
chr15	Map3k12	Intron13	N	102336000	102339720	19	84.0	57.2	73.1	56.6
chr11	Map3k3	Intron13	N	106012455	106012931	2	56.0	30.7	45.3	29.3
chr17	Rgs11	Intron13	N	26344820	26344936	1	79.3	55.2	80.0	64.3
chr4	Map7d1	Intron13	N	125917382	125917957	3	100.0	81.8	93.2	77.5
chr11	Map3k14	Intron14	N	103102421	103103428	5	63.7	4.3	71.2	11.0
chr5	Zcwpw1	Intron14	N	138259121	138260191	5	27.8	0.0	65.5	9.3
chr9	Trip4	Intron14	N	65744738	65746183	8	67.3	16.4	88.1	38.1
chr19	Abcc2	Intron14	N	43887006	43887679	3	74.6	41.1	87.2	43.2
chr13	F13a1	Intron14	N	37139374	37142063	14	75.1	56.3	91.2	51.2
chr2	Samhd1	Intron14	N	156952581	156955585	15	45.8	29.7	52.6	14.3
chr14	Ccdc66	Intron14	N	28312076	28313426	7	48.6	28.4	56.0	21.6
chr13	Fam120a	Intron14	N	49027763	49029318	8	71.6	39.1	68.2	34.2
chr2	Dhtkd1	Intron14	N	5850879	5851820	5	50.7	24.3	87.1	53.2
chr10	Myb	Intron14	N	20874562	20876145	8	61.7	36.7	54.6	23.4
chr14	Lrch1	Intron14	N	75216940	75218298	7	59.2	36.2	85.0	55.6
chr14	Acox2	Intron14	N	9089450	9091335	10	38.4	11.7	49.1	20.0
chr11	Rars	Intron14	N	35646809	35647938	4	46.4	18.4	36.1	7.7
chr13	Sptlc1	Intron14	N	53469439	53472661	14	68.0	38.8	72.9	52.0
chr1	Col4a3	Intron14	N	82656862	82657400	3	80.6	56.8	71.2	51.0
chr18	Nars	Intron14	N	64675060	64676069	4	41.3	20.3	35.2	15.4
chr14	Parp4	Intron14	N	57221745	57226258	21	86.7	71.3	90.3	71.5
chr10	Slc5a4a	Intron14	N	75649346	75651803	12	52.0	34.5	55.0	36.6
chr8	Pkd1l3	Intron14	N	112160321	112162229	10	77.8	47.6	78.8	61.4
chr1	Adck3	Intron14	N	182112379	182126003	66	58.7	39.2	55.0	38.9
chr18	4833403I15Rik	Intron14	N	47049194	47050482	6	89.7	67.6	81.2	65.1
chr7	Cd177	Intron14	N	25543469	25544619	6	73.1	46.4	63.1	47.9
chr9	Gramd1b	Intron15	N	40124554	40125093	3	79.2	47.1	91.1	3.3
chr11	Sgsm2	Intron15	N	74680139	74680532	2	79.3	59.0	93.2	31.7
chr5	Sdad1	Intron15	N	92730913	92731664	4	68.2	5.0	63.3	10.2
chr4	2010015L04Rik	Intron15	N	154808168	154808400	1	90.0	56.0	95.5	52.2
chr13	Naip1	Intron15	N	101216303	101222752	32	79.4	59.4	79.6	41.3
chr15	Tg	Intron15	N	66523950	66524808	4	78.9	58.5	64.7	33.3
chr2	Samhd1	Intron15	N	156955653	156960614	23	92.6	75.6	70.0	39.2
chr2	Spg11	Intron15	N	121896802	121897790	5	75.9	38.8	72.7	43.2
chr2	Rbm39	Intron15	N	156003122	156004912	9	64.8	15.7	70.1	41.2
chr16	Mgrn1	Intron15	N	4932299	4934194	10	76.4	46.8	77.4	48.9
chr17	Mslnl	Intron15	N	25883969	25884837	5	86.6	60.0	81.7	59.0
chr9	Dixdc1	Intron15	N	50503689	50510024	26	75.9	53.4	58.6	36.6
chr3	Odf2l	Intron15	N	144812042	144813923	9	92.0	76.9	90.0	68.6
chr2	Lrp4	Intron15	N	91321027	91322014	5	64.0	45.0	68.0	48.1
chr4	Fhad1	Intron15	N	141484400	141485444	5	44.6	29.1	57.4	39.0
chr5	Atp2a2	Intron15	N	122921263	122923218	10	80.7	64.1	76.9	59.1
chr13	Nnt	Intron15	N	120185784	120193358	38	91.1	72.3	84.7	66.9
chr10	Ccdc41	Intron15	N	94251582	94252448	4	84.4	68.9	79.8	62.4

chr6	Atp6v0a4	Intron15	N	38036096	38037929	9	77.6	46.7	74.3	58.2
chr1	Stat4	Intron15	N	52153713	52155230	8	84.3	46.0	83.4	68.2
chr11	Larp1	Intron15	N	57866234	57868968	13	97.0	80.5	86.2	71.1
chr5	Ccdc146	Intron16	N	20836568	20838837	11	80.6	55.5	79.5	17.2
chr11	Sgsm2	Intron16	N	74680658	74681112	2	95.2	16.7	72.6	20.0
chr2	Znf512b	Intron16	N	181325691	181327125	5	95.0	6.5	46.5	0.9
chr16	Prdm15	Intron16	N	98038653	98039818	6	98.6	49.1	90.1	48.3
chr13	Fam65b	Intron16	N	24802161	24805490	16	53.5	3.2	60.9	23.6
chr6	Lrig1	Intron16	N	94580234	94604840	123	69.0	43.8	73.1	43.1
chr7	Eps8l2	Intron16	N	148546327	148546900	4	84.3	58.7	91.8	70.7
chr15	Matn2	Intron16	N	34362507	34362844	2	75.0	58.6	86.2	65.3
chr5	Sdad1	Intron16	N	92731723	92732691	5	78.3	42.0	88.5	68.9
chr7	Cln3	Intron16	N	133726962	133727687	3	36.5	21.4	35.8	16.7
chr9	Slc44a2	Intron16	N	21151574	21152326	4	56.0	11.6	28.8	10.1
chr7	Muc5ac	Intron16	N	148983836	148984372	3	80.0	64.9	42.9	25.0
chr11	Ewsr1	Intron16	N	4993909	4999011	21	59.8	43.6	37.9	21.4
chr9	Cep164	Intron16	N	45587304	45587464	1	96.0	68.2	91.9	75.6
chr4	Orc1	Intron16	N	108285762	108286885	6	86.9	63.7	91.2	75.1
chr12	Daam1	Intron16	N	73059761	73060792	5	82.0	54.5	82.0	66.1
chr8	Mau2	Intron16	N	72555458	72557077	8	64.1	27.9	65.0	50.0
chr11	Sec24a	Intron17	N	51545408	51546893	7	75.5	50.9	89.1	39.0
chr13	Adamts16	Intron17	N	70932104	70934327	11	29.0	4.0	45.8	1.9
chr3	Postn	Intron17	N	54184415	54187309	14	92.8	69.6	91.7	58.8
chr11	Cpd	Intron17	N	76626234	76626984	4	44.6	19.1	50.3	18.6
chr1	Col4a3	Intron17	N	82659525	82661151	8	100.0	62.9	68.4	40.7
chr5	Rbm33	Intron17	N	28737300	28739826	13	91.8	64.9	89.0	65.2
chr12	Hdac9	Intron17	N	35075218	35078147	11	79.8	64.2	87.3	65.3
chr12	Lamb1	Intron17	N	31986076	31987426	7	67.0	38.4	75.0	56.1
chr9	Ldlr	Intron17	N	21551751	21552584	4	83.3	67.3	76.3	59.3
chr6	Lrig1	Intron17	N	94604916	94613920	45	85.5	56.1	76.5	59.6
chr3	Vav3	Intron17	N	109366123	109377023	54	56.2	30.7	52.6	37.1
chr13	Kif2a	Intron17	N	107780173	107783242	15	43.5	20.0	68.2	52.7
chr12	Efr3b	Intron17	N	3983519	3984533	5	66.7	35.6	49.3	33.9
chr17	Baiap3	Intron17	N	25384139	25384254	1	69.2	54.0	71.8	56.5
chr11	Helz	Intron17	N	107493665	107496293	13	86.1	65.6	86.8	71.7
chr2	Siglec1	Intron17	N	130909282	130909526	1	71.7	48.7	63.6	48.6
chr10	Col18a1	Intron18	N	76532244	76532564	2	68.0	37.5	76.0	21.6
chr8	Nlrc5	Intron18	N	97015471	97016230	4	92.9	54.4	44.5	1.9
chr6	Lrig1	Intron18	N	94613993	94649455	172	35.6	4.5	46.9	6.3
chr10	Pan2	Intron18	N	127752714	127752831	1	59.4	31.8	59.2	23.3
chr3	Sema6c	Intron18	N	94975312	94975479	1	21.7	3.8	39.7	4.2
chr18	Jakmip2	Intron18	N	43735214	43736953	9	91.7	68.9	93.5	66.1
chr17	Abcc10	Intron18	N	46459243	46460300	5	75.0	26.0	51.3	28.1
chr1	Plekha6	Intron18	N	135181964	135182823	4	73.5	56.1	77.8	55.2
chr19	Aldh18a1	Intron18	N	40660683	40662799	8	59.8	41.8	72.4	50.0
chr17	Ppp4r1	Intron18	N	66187365	66188157	4	39.2	22.0	79.4	57.6
chr9	Megf11	Intron18	N	64540291	64541567	6	66.7	20.7	55.2	33.6
chr7	Clasrp	Intron18	N	20180358	20180807	2	83.8	30.3	51.4	32.7
chr19	Fam178a	Intron18	N	45050206	45053650	17	96.7	68.5	70.8	52.8

chr5	A330021E22Rik	Intron18	N	5640228	5644423	20	55.1	36.6	57.8	40.5
chr15	Tars	Intron18	N	11327017	11329240	10	41.5	25.6	34.5	17.3
chr9	Mlh1	Intron18	N	111170813	111173912	14	88.9	49.1	51.9	34.8
chr19	Sf3b2	Intron18	N	5293320	5294298	5	97.5	56.9	63.1	47.1
chr11	Cntrob	Intron18	N	69135436	69136191	4	65.5	36.2	52.7	36.9
chr7	Ptprh	Intron18	N	4553959	4554649	3	72.8	32.5	45.7	30.1
chr4	Rars2	Intron18	N	34604491	34606739	11	72.3	52.5	64.0	49.0
chr7	Ptprh	Intron19	N	4554816	4555388	4	67.4	43.5	75.6	32.3
chr16	Tmprss15	Intron19	N	79057965	79062381	20	88.3	70.6	65.3	27.4
chr6	Plekha5	Intron19	N	140518708	140518841	1	69.2	30.1	65.7	27.9
chr9	Ccdc33	Intron19	N	57965439	57966241	4	74.2	50.0	72.7	46.8
chr14	Itih3	Intron19	N	31735223	31736005	4	73.7	36.4	53.4	31.4
chr1	Pik3c2b	Intron19	N	134987439	134988137	3	16.2	0.0	22.7	3.3
chr2	Mroh8	Intron19	N	157080762	157082062	7	44.1	23.9	40.6	23.6
chr16	Usp7	Intron19	N	8700296	8702054	9	59.9	36.6	73.5	57.4
chr18	Dmxl1	Intron2	N	50000167	50003439	13	58.5	38.6	97.3	6.9
chr17	Rgmb	Intron2	N	15958143	15962975	21	70.4	5.6	74.9	6.9
chr3	Cp	Intron2	N	19864528	19866238	9	82.2	53.7	76.7	10.8
chr8	Slc25a42	Intron2	N	72712351	72712731	2	62.5	3.4	61.5	2.4
chr7	Ccdc9	Intron2	N	16860725	16861086	3	87.5	48.1	66.7	10.2
chr11	Fam196b	Intron2	N	34303306	34304277	5	95.5	36.5	65.7	10.9
chr19	Fads2	Intron2	N	10139302	10140091	4	70.8	52.5	77.3	23.8
chr3	Arhgef26	Intron2	N	62145306	62149067	19	63.4	29.0	83.6	30.7
chr15	Polr3h	Intron2	N	81747832	81748697	4	92.9	48.4	89.4	37.1
chr13	Nnt	Intron2	N	120127299	120128633	7	75.1	53.5	86.0	34.4
chr8	Cyba	Intron2	N	124950168	124950835	3	72.6	26.9	56.6	5.2
chr19	Taf6l	Intron2	N	8850009	8852576	13	91.6	64.5	88.2	38.6
chr5	Abcb1a	Intron2	N	8660733	8664545	18	65.0	2.6	50.4	1.7
chr17	Zfp760	Intron2	N	21845690	21846171	3	76.5	60.0	81.4	33.9
chr6	Ninj2	Intron2	N	120148153	120148660	3	76.3	50.0	61.8	15.1
chr6	Nop2	Intron2	N	125082337	125083536	4	77.7	58.5	86.9	41.7
chr1	4930444P10Rik	Intron2	N	16058937	16068648	49	65.2	37.7	79.8	35.5
chr11	Card14	Intron2	N	119178215	119178318	2	91.3	71.1	82.4	38.2
chr19	Fam45a	Intron2	N	60890712	60893433	14	82.7	62.3	91.3	47.6
chr6	Tmem106b	Intron2	N	13020415	13021741	7	96.0	47.9	58.3	14.8
chr17	Tmem8	Intron2	N	26253879	26253963	1	36.0	5.3	46.3	2.9
chr11	BC018473	Intron2	N	116614980	116615647	3	47.2	0.3	44.0	0.7
chr17	Msrb1	Intron2	N	24876593	24877024	2	52.5	5.8	57.9	15.9
chr6	Slco1b2	Intron2	N	141581686	141582904	6	95.1	46.9	79.4	38.6
chr5	5033403H07Rik	Intron2	N	53381977	53383452	6	29.3	9.8	43.6	3.1
chr6	Mfap5	Intron2	N	122464419	122465365	5	97.1	74.6	84.3	43.9
chr2	Fam210b	Intron2	N	172177125	172178085	5	52.1	18.3	47.3	7.0
chr19	Plcb3	Intron2	N	7028958	7029194	1	77.1	29.3	71.8	33.9
chr6	Plxnd1	Intron2	N	115906098	115906612	3	76.2	23.8	87.5	49.6
chr11	Mtfp1	Intron2	N	3994037	3994391	2	52.7	28.6	52.9	15.4
chr1	Kcnj13	Intron2	N	89285981	89291186	27	74.4	26.0	77.5	41.4
chr11	Axin2	Intron2	N	108785417	108792752	37	58.4	25.9	65.5	29.7
chr6	Ptpn6	Intron2	N	124671241	124671593	2	38.0	0.7	35.5	0.0
chr5	Tmem33	Intron2	N	67652273	67654968	11	93.2	47.4	96.1	61.4

chr4	Ak2	Intron2	N	128676504	128679394	14	62.8	44.5	58.0	23.3
chr4	Uqcrh	Intron2	N	115742630	115743341	4	61.8	1.5	36.2	1.6
chr5	Anapc5	Intron2	N	123238474	123241616	16	71.6	15.0	91.4	56.9
chr7	Zc3h4	Intron2	N	17002342	17002639	1	78.7	52.5	74.7	40.2
chr11	BC096441	Intron2	N	69497495	69497719	1	65.8	31.8	72.5	38.3
chr3	Dennd2d	Intron2	N	106289967	106290768	5	82.0	55.3	83.0	49.5
chr7	Prmt3	Intron2	N	57034084	57035678	5	54.6	17.9	59.9	27.5
chr10	Rdh1	Intron2	N	127200280	127201737	7	30.2	0.0	35.5	4.0
chr2	Cdca7	Intron2	N	72317086	72319747	13	89.7	60.9	86.7	55.2
chr6	2310001H17Rik	Intron2	N	129183172	129187756	19	59.5	23.7	66.5	35.0
chr11	Arsg	Intron2	N	109346888	109347019	2	78.6	55.9	73.9	42.9
chr7	Mrvi1	Intron2	N	118020491	118021655	6	90.7	14.3	50.0	19.0
chr4	Pdik1l	Intron2	N	133840463	133843541	9	41.9	23.4	55.1	24.5
chr4	Edn2	Intron2	N	119834633	119835994	7	85.7	18.8	43.7	14.1
chr9	Traip	Intron2	N	107858254	107858676	2	88.6	59.9	71.0	41.7
chr1	Imp4	Intron2	N	34496995	34499754	12	96.2	81.0	61.1	31.9
chr19	Ms4a18	Intron2	N	11074939	11076384	7	93.5	70.7	82.7	54.0
chr9	Ube2q2	Intron2	N	55010877	55016009	24	96.2	73.0	78.5	49.9
chr7	1700003G18Rik	Intron2	N	123230270	123232626	12	79.5	45.2	76.0	47.4
chr1	Bok	Intron2	N	95583247	95585733	11	80.8	48.0	73.4	44.9
chr2	Tm9sf4	Intron2	N	153004739	153008123	17	64.9	36.3	67.2	39.3
chr11	Asic2	Intron2	N	80695184	80696923	9	86.1	46.2	73.7	46.2
chr3	Scamp3	Intron2	N	88981990	88983016	5	57.7	41.5	33.7	6.9
chr7	Tmem80	Intron2	N	148517461	148518808	8	87.1	18.2	67.7	41.0
chr1	Utp14b	Intron2	N	78656966	78659715	15	63.8	25.3	57.5	30.9
chr19	Gm10814	Intron2	N	6014357	6014868	4	23.9	6.5	31.2	4.7
chr6	Rpusd3	Intron2	N	113366441	113366782	2	39.8	17.9	36.7	10.2
chr16	Ets2	Intron2	N	95928100	95931378	16	47.0	16.5	45.6	19.1
chr1	Hnrnpu	Intron2	N	180260071	180260295	1	41.0	8.1	39.2	12.8
chr7	Bccip	Intron2	N	140905946	140906581	3	60.8	3.8	54.0	27.8
chr7	Crym	Intron2	N	127333325	127333965	3	20.4	2.0	25.8	0.0
chr6	Cd9	Intron2	N	125411328	125412131	4	72.7	27.8	82.7	57.1
chr8	Nr2c2ap	Intron2	N	72655908	72656310	3	54.0	38.2	57.8	32.2
chr2	Gzf1	Intron2	N	148510705	148512249	8	76.7	59.6	90.5	65.1
chr1	Ralgps2	Intron2	N	158741668	158743568	10	84.2	27.6	59.7	34.3
chr19	Dtx4	Intron2	N	12547810	12552615	24	66.1	45.7	64.7	39.6
chr11	Gnb2l1	Intron2	N	48615337	48615774	4	38.8	6.0	37.8	12.7
chr9	Zfp653	Intron2	N	21860537	21860857	1	32.8	12.6	39.7	14.7
chr9	Slc24a1	Intron2	N	64774143	64774969	4	82.7	54.4	64.2	39.4
chr11	Cntd1	Intron2	N	101144782	101145034	1	51.0	22.5	48.9	24.1
chr13	Trim38	Intron2	N	23877842	23880079	11	86.8	65.5	77.5	53.1
chr4	Tmem54	Intron2	N	128785649	128787618	10	86.9	55.2	80.3	56.0
chr3	5730460C07Rik	Intron2	N	153454400	153454845	2	82.5	65.7	48.8	24.7
chr7	Aen	Intron2	N	86047717	86050728	15	74.6	47.8	81.4	57.3
chr1	Uhmk1	Intron2	N	172135335	172137244	10	94.2	77.6	96.1	72.1
chr11	Prr15l	Intron2	N	96792868	96795828	15	57.7	42.0	56.6	32.9
chr3	Snapin	Intron2	N	90294195	90294469	1	17.2	0.0	25.0	1.5
chr15	Faim2	Intron2	N	99341067	99342926	9	72.3	41.9	69.7	46.6
chr6	Tada3	Intron2	N	113320402	113320932	3	59.4	17.8	49.7	26.7

chr19	Capn1	Intron2	N	5990051	5990337	1	21.4	4.5	30.7	7.8
chr5	Rpl6	Intron2	N	121655673	121655762	1	46.2	18.2	36.4	13.8
chr8	Csnk2a2	Intron2	N	97977344	97979757	12	77.2	44.9	82.9	60.4
chr16	4930578N18Rik	Intron2	N	76154096	76155985	9	86.3	68.0	94.5	72.0
chr17	Noxo1	Intron2	N	24835354	24835462	1	16.1	0.0	22.5	0.0
chr8	Wdr17	Intron2	N	55717850	55720797	15	76.9	14.3	64.9	42.4
chr13	Akr1c12	Intron2	N	4269490	4271441	9	81.3	51.6	68.4	46.0
chr6	Tpi1	Intron2	N	124761623	124761899	2	72.4	53.7	76.7	54.4
chr10	Palm	Intron2	N	79269437	79269778	2	86.0	65.0	79.3	57.1
chr5	Acads	Intron2	N	115561160	115561327	2	70.0	53.8	93.0	71.0
chr11	Ddx5	Intron2	N	106644008	106645272	7	83.3	55.8	76.0	54.0
chr12	Brf1	Intron2	N	114199493	114199933	2	91.5	66.7	87.8	65.7
chr8	Bag4	Intron2	N	26880009	26881550	8	70.1	54.9	76.6	54.7
chr17	Nfkbie	Intron2	N	45695547	45696156	3	82.8	60.9	79.6	57.8
chr8	1810012K16Rik	Intron2	N	23509751	23509916	2	60.8	17.1	45.9	24.2
chr17	Akap8	Intron2	N	32443531	32446363	14	87.9	62.5	79.1	57.4
chr7	Stard5	Intron2	N	90781296	90781639	2	29.5	2.8	21.6	0.0
chr18	Tmco6	Intron2	N	36895155	36897110	9	60.1	40.0	59.2	37.7
chr19	Hells	Intron2	N	39006003	39009903	19	64.7	43.2	48.8	27.5
chr1	Pm20d1	Intron2	N	133695260	133697670	12	43.2	8.0	59.9	38.6
chr6	Ssbp1	Intron2	N	40422133	40424663	13	62.4	46.8	66.1	44.9
chr16	Ildr1	Intron2	N	36708507	36709564	5	34.2	17.3	45.7	24.8
chr15	Rad1	Intron2	N	10416472	10417744	6	53.6	37.7	78.7	57.9
chr1	Traf3ip3	Intron2	N	195004495	195005686	6	74.3	26.4	61.1	40.5
chr4	Rps8	Intron2	N	116827034	116827242	3	38.9	15.4	42.6	22.1
chr6	Zfml	Intron2	N	83880160	83884942	22	100.0	47.6	83.9	63.6
chr10	Cisd1	Intron2	N	70799168	70807471	39	64.2	41.8	71.0	50.9
chr7	Slc1a5	Intron2	N	17371068	17371697	3	85.0	57.5	82.4	62.4
chr11	Irf1	Intron2	N	53584878	53586340	7	89.9	66.8	84.1	64.3
chr18	4930592I03Rik	Intron2	N	83089662	83089910	2	86.6	67.0	80.5	61.1
chr11	Phykpl	Intron2	N	51399135	51400062	5	89.4	61.9	61.6	42.4
chr6	Cml5	Intron2	N	85770706	85770900	2	65.7	30.4	60.7	41.6
chr12	Plekhg3	Intron2	N	77661506	77663192	8	95.1	55.5	93.6	74.4
chr12	Hs1bp3	Intron2	N	8324757	8328324	18	52.4	35.0	63.7	44.8
chr4	Txn1	Intron2	N	57958085	57963710	28	66.8	45.2	49.6	31.0
chr10	Slc18b1	Intron2	N	23518692	23523563	23	59.8	43.6	53.5	35.1
chr17	Treml4	Intron2	N	48404352	48408751	22	62.4	43.6	60.2	41.8
chr19	Rbm14	Intron2	N	4804016	4811171	33	73.7	55.5	60.2	41.8
chr6	Cops7a	Intron2	N	124909969	124910108	1	73.6	30.6	59.7	41.5
chr15	Krt8	Intron2	N	101828150	101828283	1	78.9	62.1	83.9	65.7
chr4	Extl1	Intron2	N	133914062	133914999	5	78.1	46.0	68.9	50.9
chr12	Degs2	Intron2	N	109930847	109940370	45	48.3	20.9	35.5	17.5
chr11	Dlx3	Intron2	N	94983213	94984722	6	69.8	39.7	56.8	38.9
chr8	Irf8	Intron2	N	123263892	123267605	19	73.8	51.5	76.3	58.6
chr11	Gabarap	Intron2	N	69805946	69806083	1	17.9	1.4	19.1	1.4
chr1	Aldh9a1	Intron2	N	169282797	169284557	9	93.4	64.4	87.6	70.0
chr10	Dnajc14	Intron2	N	128244677	128251093	32	80.2	64.7	84.4	66.9
chr9	Acaa1a	Intron2	N	119251037	119251840	3	83.3	42.7	62.7	45.3
chr9	Hcrtr2	Intron2	N	76078534	76080706	11	84.1	66.7	82.0	64.9

chr14	Mapk1ip1l	Intron2	N	47928264	47930089	9	94.8	79.3	77.8	60.9
chr8	Rfwd3	Intron2	N	113797923	113800119	11	83.4	61.9	86.0	69.2
chr12	Apob	Intron2	N	7985058	7986058	5	84.4	51.4	66.4	49.5
chr9	Vipr1	Intron2	N	121563023	121567164	21	47.2	23.1	35.1	18.5
chr5	Tmem120a	Intron2	N	136211836	136211939	1	85.8	56.0	92.5	75.9
chr4	Ybx1	Intron2	N	118951805	118954174	12	84.6	64.7	81.8	65.3
chr11	Lypd8	Intron2	N	58193212	58195786	13	36.8	18.0	30.3	13.8
chr5	Cox19	Intron2	N	139818629	139821025	11	67.3	38.4	42.8	26.5
chr4	Fam131c	Intron2	N	140935709	140936233	3	61.5	29.3	48.2	31.8
chr2	Acbd5	Intron2	N	22925140	22931009	28	34.8	10.8	59.0	42.8
chr19	Rad9a	Intron2	N	4196383	4197112	4	51.9	27.9	26.2	10.1
chr11	Rnf167	Intron2	N	70461612	70462722	5	51.3	34.8	51.8	35.7
chr7	Vmn2r30	Intron2	N	7269907	7275760	15	81.6	65.4	76.9	60.9
chr14	Pdlim2	Intron2	N	70566015	70567467	7	72.3	51.1	72.0	56.0
chr4	Klhl21	Intron2	N	151386815	151388415	8	94.4	59.3	84.6	68.8
chr13	Eif4e1b	Intron2	N	54885924	54886169	1	58.4	35.7	55.2	39.5
chr8	Otud4	Intron2	N	82167737	82170196	11	88.3	65.0	91.9	76.3
chr7	Kcnn4	Intron2	N	25159787	25162301	11	91.0	51.3	74.9	59.3
chr11	Gga3	Intron2	N	115447511	115447629	1	83.6	63.4	78.2	62.6
chr6	Frmd4b	Intron2	N	97242490	97245569	15	56.6	27.5	41.3	25.8
chr13	Zscan26	Intron2	N	21539764	21541309	8	50.1	29.6	49.4	34.1
chr18	Gramd3	Intron2	N	56629307	56633672	22	49.6	19.7	45.1	29.8
chr8	Amfr	Intron2	N	96498144	96498635	2	94.7	54.3	65.7	50.6
chr8	Fto	Intron2	N	93925784	93933121	37	62.5	46.8	60.2	45.1
chr12	Sypl	Intron2	N	33639225	33650237	48	21.8	3.8	17.6	2.5
chr17	Myom1	Intron20	N	71436911	71439162	11	58.9	21.5	57.2	35.6
chr9	Usp28	Intron20	N	48844066	48845281	6	88.0	55.3	67.2	47.3
chr7	Nadsyn1	Intron20	N	151007138	151008563	6	70.9	31.5	61.0	45.1
chr6	C2cd5	Intron20	N	143026982	143028388	7	82.4	29.5	84.3	68.5
chr10	Pwp2	Intron20	N	77646916	77647815	3	55.0	21.0	39.6	23.9
chr15	Adcy6	Intron20	N	98431492	98434304	14	46.6	7.1	34.1	18.5
chr6	Ptpro	Intron21	N	137391243	137392038	4	70.4	30.1	81.9	33.4
chr1	Unc80	Intron21	N	66598825	66601500	12	90.0	65.5	92.9	56.8
chr6	Gucy2c	Intron21	N	136709293	136711518	11	59.3	25.7	53.5	21.7
chr16	Dgkg	Intron21	N	22599961	22600511	3	60.3	29.8	66.7	37.2
chr9	Usp28	Intron21	N	48845362	48845872	3	85.4	62.2	79.6	53.6
chr5	Hfm1	Intron21	N	107318969	107321716	13	42.7	20.1	48.6	23.8
chr1	Pik3c2b	Intron21	N	134988942	134990232	6	88.9	67.5	84.3	59.9
chr14	Appl1	Intron21	N	27778144	27783586	23	62.5	44.4	66.7	46.8
chr2	Ralgapb	Intron21	N	158291708	158292471	4	87.0	67.9	86.3	69.6
chr3	Arhgap29	Intron22	N	121715092	121717018	10	85.9	61.7	71.6	4.4
chr10	Cabin1	Intron22	N	75200261	75200955	3	82.1	30.6	81.6	34.7
chr7	Adam8	Intron22	N	147176248	147177244	5	59.6	14.6	63.4	30.6
chr7	Nphs1	Intron22	N	31259672	31262877	16	74.1	52.8	73.7	51.0
chr4	Abca1	Intron22	N	53080028	53081558	8	70.0	52.0	66.3	48.0
chr9	Adamts7	Intron22	N	90090916	90091837	5	62.5	30.6	50.5	32.8
chr16	Pdxdc1	Intron22	N	13887864	13903007	72	67.9	50.4	62.1	44.9
chr7	Abca14	Intron22	N	127426756	127432971	31	87.3	49.1	87.3	70.6
chr7	C2cd3	Intron22	N	107579337	107580480	6	40.0	22.6	65.3	49.3

chr1	Gigyf2	Intron22	N	89330492	89333343	14	67.3	39.9	78.8	63.4
chr15	Bai1	Intron23	N	74404599	74406268	8	63.0	5.4	70.4	20.2
chr2	Ccdc141	Intron23	N	77006691	77008441	8	97.8	49.0	95.8	61.5
chr12	Hdac9	Intron23	N	35118097	35122001	18	60.0	43.0	79.7	62.1
chr2	Ralgapa2	Intron24	N	146237896	146238469	3	32.1	10.6	59.2	9.0
chr1	Armc9	Intron24	N	88171131	88172234	6	62.7	10.3	73.4	51.6
chr16	Ttc3	Intron24	N	94647851	94648372	3	82.1	44.1	55.0	35.4
chr1	Col4a4	Intron24	N	82493913	82494402	2	81.8	54.5	80.6	64.1
chr11	Obscn	Intron24	N	58834071	58834921	4	82.7	62.0	78.5	62.9
chr11	Spag9	Intron25	N	93969941	93973346	16	92.2	63.5	95.0	56.1
chr8	Edc4	Intron25	N	108415034	108415118	1	51.4	14.3	61.4	31.1
chr1	Spta1	Intron25	N	176141787	176142668	4	47.4	9.0	53.0	26.0
chr1	Hdlbp	Intron25	N	95334961	95337256	11	93.9	69.2	89.6	67.0
chr4	Ptprd	Intron25	N	75784478	75785197	2	71.2	38.6	60.0	39.5
chr8	Sf3b3	Intron25	N	113368500	113370574	8	61.1	41.6	55.2	40.0
chr1	2310035C23Rik	Intron26	N	107642763	107646898	20	69.8	52.2	77.1	52.5
chr5	Nos1	Intron26	N	118399838	118402773	15	66.9	51.5	67.4	45.2
chr11	Cep112	Intron27	N	108720722	108721505	4	60.2	42.7	59.3	10.0
chr2	Kcnt1	Intron27	N	25765457	25765785	2	74.3	36.2	75.2	38.0
chr10	Syne1	Intron27	N	5235167	5235505	1	84.6	45.5	76.7	52.4
chr8	Pnpla6	Intron27	N	3537020	3537248	1	52.3	24.1	32.1	13.3
chr18	Atp8b1	Intron27	N	64765055	64820353	269	70.3	54.1	66.4	50.1
chr15	Col2a1	Intron28	N	97817272	97817723	2	82.5	62.3	85.4	66.2
chr2	Ubr3	Intron29	N	69831870	69838514	31	67.8	35.7	76.2	50.2
chr8	Trappc11	Intron29	N	48616232	48618672	11	62.8	37.5	40.6	15.6
chr17	Prrc2a	Intron29	N	35299325	35299816	2	58.3	42.4	48.7	27.2
chr8	Ddx60	Intron29	N	64479501	64488738	44	73.8	54.8	80.3	59.8
chr11	Itgae	Intron29	N	72954265	72959042	24	79.7	59.3	75.6	59.1
chr14	Ldb3	Intron3	N	35355175	35355612	2	62.3	6.7	84.8	1.3
chr17	Pkmyt1	Intron3	N	23871284	23871932	3	65.2	0.0	78.1	3.4
chr7	Mef2a	Intron3	N	74385327	74389406	19	76.5	54.7	81.0	11.6
chr1	Faim3	Intron3	N	132771731	132772539	4	55.6	23.0	81.2	14.7
chr9	Arhgap32	Intron3	N	32052840	32053502	3	38.9	0.0	56.5	0.0
chr19	Capn1	Intron3	N	5990455	5991538	5	66.7	25.0	86.6	32.8
chr15	Dazap2	Intron3	N	100448602	100449798	6	80.0	47.8	74.0	20.5
chr5	0610040J01Rik	Intron3	N	64287723	64289193	7	71.5	45.6	69.4	20.2
chr15	Krt71	Intron3	N	101567199	101568376	6	43.5	12.5	59.9	11.8
chr6	Cd9	Intron3	N	125412222	125412344	1	87.0	56.8	96.9	49.0
chr4	D4Wsu53e	Intron3	N	134481348	134482649	7	78.6	63.0	76.9	29.7
chr11	Rnft1	Intron3	N	86300235	86302568	12	82.1	61.6	87.4	43.4
chr5	Snx17	Intron3	N	31497287	31497787	3	89.6	23.5	89.2	45.5
chr9	Arih2	Intron3	N	108509688	108510239	3	79.5	52.2	89.6	47.3
chr1	Slc19a2	Intron3	N	166191149	166192177	4	96.4	53.1	78.0	36.8
chr4	1700024P16Rik	Intron3	N	104621438	104623457	10	64.1	44.0	69.2	29.8
chr19	Lzts2	Intron3	N	45098695	45099208	1	69.6	14.3	57.4	20.3
chr13	Slc25a48	Intron3	N	56550431	56552381	10	63.0	35.7	74.9	37.9
chr7	Kcne3	Intron3	N	107329785	107331242	9	47.6	16.3	45.1	9.2
chr12	2210039B01Rik	Intron3	N	74651229	74652142	5	33.7	6.0	38.7	3.0
chr1	Inpp1	Intron3	N	52851515	52853884	12	84.7	50.8	85.7	50.0

chr10	Arhgap18	Intron3	N	26569917	26574679	24	83.2	65.0	92.1	59.1
chr7	Etfb	Intron3	N	50708328	50709887	8	62.1	43.3	58.2	26.3
chr9	Taf1d	Intron3	N	15113360	15114302	5	25.0	1.9	31.8	0.0
chr11	Cntd1	Intron3	N	101145207	101146073	4	40.5	8.5	39.4	7.7
chr10	Arhgap9	Intron3	N	126762687	126762899	1	41.8	14.4	49.8	18.7
chr3	Ifi44l	Intron3	N	151424481	151425680	5	81.1	54.9	80.9	51.4
chr4	Kif12	Intron3	N	62827542	62827967	2	47.4	10.3	44.8	15.3
chr9	Nr2e3	Intron3	N	59795361	59796169	4	82.0	45.9	90.6	61.5
chr10	1500009L16Rik	Intron3	N	83220226	83220878	3	67.6	52.5	70.1	42.0
chr11	Vmp1	Intron3	N	86415572	86420677	26	44.8	8.7	27.8	0.0
chr8	Mylk3	Intron3	N	87852583	87860266	37	82.1	60.8	84.1	57.9
chr11	Mtmr3	Intron3	N	4388636	4389624	5	88.7	70.7	94.2	68.6
chr6	Fam115a	Intron3	N	42627030	42627328	1	72.0	49.0	75.6	50.0
chr7	Bccip	Intron3	N	140906663	140908543	9	71.6	36.4	74.7	49.2
chr5	1700028E10Rik	Intron3	N	152210643	152216201	28	61.0	33.4	62.8	37.8
chr9	Pkm	Intron3	N	59513762	59516355	13	72.8	46.7	77.7	52.9
chr2	Ube2c	Intron3	N	164596880	164597353	2	86.0	41.7	62.5	37.7
chr10	Madcam1	Intron3	N	79128503	79129083	3	56.7	36.9	63.7	39.1
chr11	Nhp2	Intron3	N	51436093	51436593	4	87.3	66.7	79.2	54.6
chr13	Dsp	Intron3	N	38259534	38260474	5	94.4	60.6	95.7	71.1
chr7	Tufm	Intron3	N	133631941	133632163	1	64.9	43.2	61.5	37.3
chr19	Fads2	Intron3	N	10140172	10140741	3	18.2	0.0	24.0	0.0
chr18	Zfp191	Intron3	N	24176670	24179212	10	17.2	2.1	26.1	2.5
chr11	Nufip2	Intron3	N	77519044	77523184	21	82.3	54.9	77.5	54.4
chr1	Nuak2	Intron3	N	134222807	134224273	7	69.6	54.0	77.0	54.6
chr1	Aldh9a1	Intron3	N	169284688	169285802	6	43.8	15.9	59.2	36.9
chr7	Fan1	Intron3	N	71494398	71494920	3	85.1	67.9	92.5	70.3
chr7	Eps8l2	Intron3	N	148528010	148528824	4	75.4	39.5	62.5	40.4
chr14	4930471C04Rik	Intron3	N	64722339	64722937	4	73.2	49.5	74.0	52.0
chr4	Extl1	Intron3	N	133915161	133915663	3	86.0	66.2	81.2	59.4
chr7	Lamtor1	Intron3	N	109058621	109058933	2	71.2	54.2	68.6	47.3
chr17	BC004004	Intron3	N	29419213	29419622	2	63.9	3.3	37.4	16.8
chr15	Pdzd2	Intron3	N	12297836	12298373	3	51.5	27.0	57.1	36.9
chr7	Uqcrc2	Intron3	N	127781512	127783747	11	96.8	71.7	94.0	74.3
chr12	Ifrd1	Intron3	N	40931013	40933575	13	66.7	49.2	67.7	47.9
chr3	Trim46	Intron3	N	89041842	89042819	5	44.3	18.2	33.9	14.2
chr4	Hmgcl	Intron3	N	135508394	135509771	7	95.2	38.1	57.6	38.3
chr2	Oser1	Intron3	N	163241392	163245147	16	48.6	31.1	54.0	34.7
chr16	Prodh	Intron3	N	18073747	18076334	12	70.3	55.1	62.4	43.1
chr1	Serpinb8	Intron3	N	109495615	109499376	19	82.3	64.7	85.2	65.9
chr10	Hsd17b6	Intron3	N	127431644	127434753	16	88.8	72.9	86.5	67.4
chr13	Trim27	Intron3	N	21275832	21279909	20	67.6	50.1	73.5	54.4
chr9	Rbpms2	Intron3	N	65497813	65498368	3	48.6	31.7	63.5	44.4
chr11	Sgsm2	Intron3	N	74665339	74666576	6	97.8	81.3	80.1	61.7
chr9	Ppcdc	Intron3	N	57268099	57268932	4	56.1	18.4	44.0	25.6
chr17	Themis3	Intron3	N	66942624	66943786	6	87.8	63.2	87.5	69.4
chr15	Fam173b	Intron3	N	31537989	31539320	7	29.2	4.6	21.9	4.1
chr11	Hsf5	Intron3	N	87445192	87449112	19	79.0	57.5	84.0	66.3
chr19	Slc22a29	Intron3	N	8237248	8243644	31	74.5	46.6	79.2	61.6

chr2	Bpifb5	Intron3	N	154052932	154053798	4	81.0	64.5	63.8	46.3
chr9	Atp2c1	Intron3	N	105320476	105320873	2	97.9	48.2	93.3	75.8
chr19	Cabp4	Intron3	N	4137217	4138548	7	74.4	58.9	80.7	63.2
chr15	Krt18	Intron3	N	101860411	101861049	3	89.7	74.0	90.0	72.7
chr12	Tcl1b1	Intron3	N	106403750	106404242	3	79.0	55.8	68.7	51.7
chr2	Stom	Intron3	N	35177207	35179183	10	81.4	55.8	65.6	48.7
chr8	Bag4	Intron3	N	26881806	26887894	29	79.1	59.3	77.3	60.6
chr15	4930447A16Rik	Intron3	N	37369371	37370232	6	77.4	54.4	64.1	47.5
chr11	Jmjd6	Intron3	N	116701860	116702382	3	84.7	13.0	69.4	53.1
chr2	Ppp1r14d	Intron3	N	119044584	119055277	53	83.7	61.9	82.1	65.9
chr2	9430008C03Rik	Intron3	N	158185068	158186199	7	41.6	19.6	37.5	21.3
chr7	EU599041	Intron3	N	50479332	50481081	8	87.0	66.5	90.1	74.0
chr3	D3Ertd751e	Intron3	N	41550651	41552594	10	95.2	75.9	87.5	71.4
chr6	Clec2e	Intron3	N	129045219	129048380	16	55.8	32.5	40.2	24.2
chr11	Chmp6	Intron3	N	119777034	119777338	2	28.9	2.2	17.8	1.8
chr16	Itsn1	Intron3	N	91784401	91785532	6	29.7	3.2	67.1	51.1
chr11	Axin2	Intron3	N	108792894	108800625	39	80.2	49.1	77.5	61.6
chr4	Plod1	Intron3	N	147290053	147291413	7	80.4	60.8	83.1	67.5
chr19	Fth1	Intron3	N	10058818	10059049	2	92.4	75.9	91.2	75.6
chr6	Gpr19	Intron3	N	134847232	134847849	2	18.5	2.3	16.4	0.8
chr2	4930526D03Rik	Intron3	N	181432369	181433111	4	79.0	56.1	70.1	54.6
chr4	Nadk	Intron3	N	154953504	154958245	24	83.0	67.2	69.2	53.7
chr5	Hadhb	Intron3	N	30490611	30493204	13	39.1	11.9	44.0	28.6
chr10	Atp5b	Intron3	N	127521528	127522146	5	89.5	46.5	54.2	38.8
chr5	Chchd2	Intron3	N	130360055	130363067	13	95.1	75.5	71.2	55.9
chr1	Rnasel	Intron3	N	155600078	155600736	4	73.3	29.7	72.7	57.5
chr3	Phf17	Intron3	N	41392945	41395604	13	67.5	48.3	64.6	49.6
chr1	Pik3c2b	Intron30	N	135000012	135000377	2	60.0	32.8	82.0	21.7
chr11	Cltc	Intron30	N	86547347	86550572	16	72.2	53.5	74.9	27.2
chr9	Atm	Intron30	N	53290549	53292177	7	96.2	65.0	92.2	67.1
chr17	Myom1	Intron30	N	71459181	71460046	4	77.7	34.9	81.8	58.8
chr9	Arhgef12	Intron30	N	42818337	42823985	26	72.2	47.6	70.8	50.9
chr6	Plxnd1	Intron30	N	115926497	115926587	1	66.7	50.0	66.1	48.8
chr14	Stab1	Intron30	N	31961118	31961203	1	92.6	42.9	80.0	63.2
chr3	Med12l	Intron31	N	59063636	59064665	6	52.6	25.2	64.3	35.9
chr11	2310067B10Rik	Intron31	N	115659293	115659588	1	63.6	30.7	46.0	24.0
chr14	Rnf17	Intron31	N	57128173	57131047	13	95.0	75.2	80.9	60.6
chr2	Eif2ak4	Intron32	N	118292176	118293897	9	100.0	67.2	83.5	50.0
chr10	Mon2	Intron32	N	122488825	122489240	2	46.6	21.4	41.2	11.8
chr17	Lrpprc	Intron32	N	85170891	85171432	3	62.5	32.4	63.0	43.9
chr11	Top2a	Intron32	N	98880256	98883024	14	85.4	56.4	89.7	74.3
chr19	Myof	Intron33	N	38031040	38034203	16	83.5	27.7	72.2	32.0
chr3	Notch2	Intron33	N	97949143	97949975	4	70.9	39.0	75.4	48.6
chr1	Rab3gap2	Intron33	N	187106390	187107260	4	62.2	29.0	54.6	33.6
chr9	Col6a4	Intron33	N	105974890	105977152	11	82.4	61.9	73.9	56.1
chr4	Myom3	Intron33	N	135364875	135366663	9	54.8	31.6	44.6	26.9
chr5	Ptpn13	Intron33	N	103997204	103998448	6	20.9	0.0	62.6	46.0
chr11	Abca6	Intron34	N	110105708	110106531	4	79.7	37.2	80.4	45.6
chr14	Tep1	Intron34	N	51464849	51465090	1	77.2	47.7	79.3	52.2

chr16	Ttc3	Intron34	N	94666158	94669605	13	48.3	30.7	65.9	40.0
chr1	Kif1a	Intron34	N	94959524	94960725	6	45.7	24.8	45.9	30.7
chr4	Unc13b	Intron36	N	43271822	43272333	3	79.3	45.3	74.8	29.8
chr15	Myh9	Intron36	N	77622777	77626390	18	85.3	65.0	80.9	61.0
chr9	Myo9a	Intron36	N	59753892	59755154	6	92.0	74.4	100.0	81.4
chr1	Kif1a	Intron37	N	94963712	94965061	7	90.1	25.4	65.0	40.6
chr2	Fbn1	Intron37	N	125187043	125188197	6	71.9	39.8	55.0	34.5
chr15	Myo10	Intron38	N	25736933	25737490	3	89.6	64.9	77.8	40.7
chr5	Fryl	Intron38	N	73486713	73487512	4	95.9	67.5	94.0	74.5
chr5	Hgfac	Intron4	N	35385584	35386208	3	92.3	0.0	88.2	0.0
chr16	Phldb2	Intron4	N	45757359	45758229	4	64.0	17.0	82.1	14.5
chr4	Slc30a2	Intron4	N	133903409	133904391	5	47.4	1.4	65.8	3.9
chr12	Tmx1	Intron4	N	71559895	71561331	7	92.2	61.8	67.6	11.4
chr3	Ppid	Intron4	N	79401796	79402713	5	35.4	6.9	70.7	15.1
chr7	Nlrp6	Intron4	N	148110056	148111014	5	83.1	9.6	79.6	26.2
chr19	Gm10814	Intron4	N	6016484	6017797	7	69.0	32.9	70.6	20.3
chr13	Grk6	Intron4	N	55551946	55552288	2	92.9	50.8	86.0	37.4
chr1	Slc23a3	Intron4	N	75125584	75125971	2	63.2	22.8	68.0	20.6
chr1	Arfgef1	Intron4	N	10132826	10134798	10	86.4	54.9	89.7	42.4
chr6	Clec2e	Intron4	N	129048507	129050803	11	74.8	27.5	84.7	37.6
chr4	Aldob	Intron4	N	49552669	49554002	7	60.0	40.0	86.1	41.4
chr13	Slc6a19	Intron4	N	73821848	73823212	7	64.6	38.1	68.2	26.4
chr6	Cald1	Intron4	N	34703589	34705633	9	82.0	47.7	81.2	40.5
chr1	Vil1	Intron4	N	74465131	74466142	5	55.9	17.3	57.5	18.1
chr7	Swap70	Intron4	N	117407622	117409349	9	51.6	26.2	65.4	26.7
chr17	Rrp1b	Intron4	N	32185584	32186370	4	65.0	47.2	67.6	31.4
chr7	Bnip3	Intron4	N	146089761	146090363	3	90.8	46.6	37.6	1.4
chr1	Lamc1	Intron4	N	155074841	155075864	5	91.5	65.2	95.7	59.6
chr11	Myh2	Intron4	N	66987071	66988444	7	85.3	63.9	89.4	53.3
chr2	Dok5	Intron4	N	170655689	170658381	13	90.9	51.8	91.1	55.0
chr12	Serpina1f	Intron4	N	104932233	104932806	3	84.6	49.9	67.6	33.3
chr13	Mcidas	Intron4	N	113787124	113787570	2	54.8	16.2	47.5	13.9
chr11	Gsdma2	Intron4	N	98510966	98512157	6	89.8	50.9	79.4	46.1
chr18	Aqp4	Intron4	N	15558512	15562108	18	55.4	31.3	52.8	20.1
chr8	Col4a1	Intron4	N	11206404	11207193	4	72.7	24.4	76.8	45.5
chr7	Sphk2	Intron4	N	52967976	52968629	3	34.0	2.0	33.6	2.6
chr8	Plekhg4	Intron4	N	107900414	107900493	1	52.2	16.4	50.8	20.5
chr17	Epcam	Intron4	N	88039869	88040773	5	43.9	9.6	41.4	11.2
chr11	Cant1	Intron4	N	118272826	118278887	31	66.7	26.0	58.1	28.6
chr9	Spa17	Intron4	N	37419604	37421140	6	91.2	48.4	87.4	58.2
chr4	Ldlrad1	Intron4	N	106888883	106890370	7	89.7	52.4	83.1	55.4
chr4	Txn1	Intron4	N	57964774	57969058	18	40.8	19.7	31.8	4.3
chr9	Cox5a	Intron4	N	57379600	57380088	3	18.0	0.0	44.8	18.1
chr17	Prss27	Intron4	N	24181499	24181825	2	29.2	3.1	28.9	3.0
chr11	Nbr1	Intron4	N	101417606	101420832	16	92.2	30.5	77.2	51.4
chr19	Capn1	Intron4	N	5991618	5991855	1	63.1	33.7	68.8	43.2
chr19	Fra10ac1	Intron4	N	38274169	38276036	9	61.9	46.8	85.7	60.3
chr1	Ppfia4	Intron4	N	136197116	136200181	15	77.9	56.8	70.4	46.5
chr9	Map4	Intron4	N	109928728	109930182	7	89.3	61.7	86.4	62.5

chr7	C230079O03Rik	Intron4	N	143540565	143541002	2	57.1	28.0	67.0	43.3
chr9	Snx1	Intron4	N	65938538	65940965	12	40.9	15.0	47.1	23.7
chr3	Phtf1	Intron4	N	103785879	103786868	5	55.0	2.8	77.8	54.5
chr4	Mpl	Intron4	N	118119120	118121150	9	33.1	3.8	53.9	30.8
chr12	Itgb1bp1	Intron4	N	21280764	21282680	10	100.0	84.6	90.5	67.7
chr2	Cdk5rap1	Intron4	N	154174518	154176553	10	92.3	77.1	86.3	63.6
chr4	Zc3h12a	Intron4	N	124799638	124803849	21	85.2	33.2	52.9	30.4
chr3	Gucy1a3	Intron4	N	81910170	81912515	12	83.2	60.7	81.6	59.3
chr15	Foxred2	Intron4	N	77778950	77782399	17	84.0	68.1	84.7	62.6
chr11	Cldn7	Intron4	N	69780807	69781056	2	30.3	12.4	36.2	14.2
chr8	Nek3	Intron4	N	23242666	23243464	5	52.8	27.0	46.8	24.8
chr4	Lzic	Intron4	N	148862838	148867307	22	84.8	65.3	78.3	56.6
chr9	Slc25a36	Intron4	N	96993597	96997521	19	85.0	69.2	70.2	49.2
chr3	Rabggtb	Intron4	N	153572424	153572509	1	58.3	42.5	71.2	50.5
chr11	Vmp1	Intron4	N	86420795	86424843	20	74.9	59.5	62.0	41.3
chr6	Phb2	Intron4	N	124664187	124664847	3	57.4	41.0	58.3	37.7
chr3	Adh5	Intron4	N	138110904	138113862	15	83.4	62.1	85.4	65.4
chr1	Serpinb5	Intron4	N	108771738	108772586	4	67.1	35.5	45.6	25.7
chr9	Fbxl2	Intron4	N	113893704	113894390	3	33.3	12.2	67.9	48.1
chr16	Ostn	Intron4	N	27347054	27350493	18	89.5	40.0	90.7	71.1
chr5	2210019I11Rik	Intron4	N	148050990	148052122	6	62.6	43.7	44.8	25.3
chr13	Sgtb	Intron4	N	104911617	104914269	13	66.9	40.9	67.6	48.2
chr17	Fez2	Intron4	N	78800239	78802079	9	53.9	23.4	56.9	37.6
chr18	Mro	Intron4	N	74031719	74032877	6	40.3	6.7	43.8	24.6
chr2	Armc3	Intron4	N	19160394	19165575	26	87.5	58.6	75.6	56.4
chr9	Acat1	Intron4	N	53395672	53397282	8	40.4	13.3	71.1	51.9
chr9	Fxyd6	Intron4	N	45198935	45199643	4	89.6	48.2	49.2	30.1
chr15	Tmbim6	Intron4	N	99232601	99234844	11	59.1	41.6	44.8	25.8
chr15	Ccdc65	Intron4	N	98549282	98551014	9	72.6	55.8	73.4	55.1
chr5	Sult1d1	Intron4	N	87988913	87992894	18	90.9	43.0	76.8	59.0
chr15	Slc30a8	Intron4	N	52153408	52157114	19	85.1	59.5	83.1	65.3
chr7	Izumo1	Intron4	N	52879592	52880031	2	77.1	60.6	80.2	63.2
chr13	Elovl7	Intron4	N	109060908	109061998	5	78.1	60.6	76.0	59.3
chr8	Slc35e1	Intron4	N	75013489	75015740	9	62.7	43.0	65.9	49.3
chr1	B230209K01Rik	Intron4	N	123014043	123016434	12	75.6	49.6	70.2	53.6
chr3	Fndc7	Intron4	N	108665906	108669979	20	62.9	42.5	74.5	58.0
chr17	Capn11	Intron4	N	45769037	45769329	1	69.1	43.8	58.9	42.5
chr5	Fam184b	Intron4	N	45925510	45928827	17	83.5	67.0	61.9	45.5
chr16	App	Intron4	N	84971707	84978308	34	77.8	59.2	85.6	69.4
chr4	Dhdds	Intron4	N	133547296	133548378	5	36.1	17.9	39.5	23.4
chr11	Otop2	Intron4	N	115186019	115187734	9	77.0	56.8	67.1	51.1
chr1	Mrpl15	Intron4	N	4774187	4775653	5	64.7	22.5	46.8	31.3
chr5	Msi1	Intron4	N	115880898	115883860	14	58.1	36.5	54.4	38.9
chr1	Rgs16	Intron4	N	155589260	155590763	8	78.7	54.1	74.5	59.0
chr18	Rell2	Intron4	N	38116810	38117242	3	90.8	71.4	73.1	57.7
chr6	Trim24	Intron4	N	37865298	37869373	19	100.0	75.2	95.1	80.0
chr2	Itgav	Intron4	N	83596078	83602024	30	86.7	61.1	89.2	74.1
chr5	Fryl	Intron40	N	73488809	73489397	3	87.4	49.4	83.2	46.5
chr6	Dnah6	Intron41	N	73073888	73074608	4	20.5	1.1	62.6	40.9

chr7	Muc5ac	Intron42	N	149001513	149001852	2	55.0	4.2	47.5	2.6
chr14	Flnb	Intron42	N	8771694	8774558	14	66.1	50.8	61.0	34.8
chr5	Zan	Intron43	N	137860489	137861121	3	40.4	19.9	49.2	19.7
chr2	Lrp2	Intron43	N	69326120	69327191	5	90.2	45.8	29.2	10.5
chr1	Fer1l5	Intron43	N	36474381	36474568	1	76.4	59.1	81.1	62.9
chr5	Ptpn13	Intron45	N	104020373	104022525	11	82.6	63.2	82.7	65.9
chr7	Nlrp6	Intron5	N	148111108	148112313	6	83.2	30.4	86.2	33.1
chr11	Etaa1	Intron5	N	17852707	17853565	2	45.0	0.0	50.0	0.0
chr9	Ifrd2	Intron5	N	107492994	107493126	1	64.1	42.9	74.4	27.2
chr2	Il1b	Intron5	N	129195488	129196019	4	82.7	46.6	75.5	30.3
chr17	BC004004	Intron5	N	29431107	29431219	1	31.6	0.0	42.6	0.0
chr8	Tlr3	Intron5	N	46494090	46495149	7	61.9	8.3	53.8	17.7
chr7	Siglec5	Intron5	N	50608040	50610345	12	38.9	4.4	40.6	4.8
chr12	Entpd5	Intron5	N	85725271	85726097	4	50.1	9.8	45.9	10.3
chr11	Eme1	Intron5	N	94509377	94509456	1	87.0	60.0	86.8	51.9
chr4	Zdhhc18	Intron5	N	133169854	133171094	6	66.9	49.4	82.4	48.2
chr3	Slc25a24	Intron5	N	108959924	108961415	7	32.6	0.7	34.5	2.1
chr19	Kdm2a	Intron5	N	4325047	4326808	9	79.2	61.9	88.7	56.6
chr10	Hsf2	Intron5	N	57219298	57221175	9	45.5	15.7	46.8	15.3
chr7	Ceacam1	Intron5	N	26257093	26258816	9	41.7	22.0	51.1	20.0
chr1	Suco	Intron5	N	163759472	163763546	19	84.9	57.7	90.6	60.0
chr1	Uck2	Intron5	N	169166831	169167619	4	60.3	30.0	64.2	34.1
chr13	Tdp2	Intron5	N	24930135	24932256	11	68.6	53.0	77.8	47.9
chr4	Gm12695	Intron5	N	96415935	96420746	24	62.8	28.2	35.2	5.4
chr9	Exph5	Intron5	N	53175318	53180347	25	56.0	31.8	51.3	22.8
chr15	Hnrnpa1	Intron5	N	103072730	103072810	1	50.0	23.3	33.3	5.6
chr9	Chrnb4	Intron5	N	54891733	54896250	22	50.5	34.7	43.8	16.5
chr4	Hmgcl	Intron5	N	135511651	135514637	15	39.4	13.4	55.4	28.3
chr19	Cep78	Intron5	N	16043691	16045022	7	94.3	58.3	93.5	66.5
chr7	Ears2	Intron5	N	129190247	129191615	7	70.1	40.0	86.4	59.5
chr5	Wbscr16	Intron5	N	134639676	134641208	8	83.6	63.3	82.3	55.5
chr8	Slc25a42	Intron5	N	72713988	72715759	9	64.5	20.4	50.6	23.9
chr1	Kmo	Intron5	N	177573738	177577243	17	60.1	15.3	57.8	31.5
chr14	Diap3	Intron5	N	87228964	87234783	26	79.5	53.4	90.1	64.5
chr2	Pigt	Intron5	N	164326019	164326580	3	85.6	67.7	86.7	61.1
chr7	Il18bp	Intron5	N	109165866	109166110	2	37.2	2.3	25.4	0.0
chr10	Agap2	Intron5	N	126520635	126522168	8	69.6	17.6	48.1	22.8
chr3	Adh6a	Intron5	N	137988283	137988879	3	88.7	71.2	92.5	68.8
chr3	Pdgfc	Intron5	N	81013184	81016383	15	76.0	32.1	60.7	37.1
chr13	Rslcan18	Intron5	N	67208990	67213123	17	76.2	52.2	66.3	42.9
chr10	Lss	Intron5	N	75999111	76001096	10	57.4	36.3	60.4	36.9
chr1	Arpc2	Intron5	N	74301589	74302531	5	70.7	42.7	80.4	57.1
chr11	Slfn5	Intron5	N	82774231	82774401	2	81.6	64.9	85.3	62.1
chr6	Camk1	Intron5	N	113287300	113288104	4	97.9	58.4	76.0	52.9
chr1	Nuak2	Intron5	N	134224774	134226534	9	64.1	36.6	53.7	30.8
chr1	Wdfy1	Intron5	N	79710539	79711448	5	71.6	47.2	77.7	55.3
chr1	Cd247	Intron5	N	167788610	167789349	4	88.9	58.9	69.8	47.6
chr14	Slc7a7	Intron5	N	54994002	54994231	1	83.3	60.4	81.3	59.7
chr12	Syne3	Intron5	N	106183421	106184775	7	74.7	25.8	72.6	51.4

chr10	Sgk1	Intron5	N	21715356	21715599	1	41.7	18.4	30.7	9.8
chr10	Gucd1	Intron5	N	74974902	74979898	23	61.3	40.8	52.9	32.2
chr10	Ppap2c	Intron5	N	78993774	78996368	10	32.0	14.1	32.2	11.7
chr7	Fcgrt	Intron5	N	52358042	52358372	2	66.4	41.3	55.8	35.3
chr5	Prkab1	Intron5	N	116470477	116471491	5	86.4	60.0	84.6	64.1
chr14	Rpgrip1	Intron5	N	52740248	52740667	2	61.9	41.2	57.3	37.3
chr8	Spg7	Intron5	N	125600891	125601717	4	55.3	33.9	47.9	28.2
chr6	Chn2	Intron5	N	54245916	54248038	11	100.0	27.7	88.5	68.8
chr1	Gdap1	Intron5	N	17150120	17151207	5	71.4	46.5	77.1	57.4
chr15	Fam134b	Intron5	N	25896473	25898161	8	92.6	48.7	69.6	50.0
chr17	Stub1	Intron5	N	25968907	25968990	1	52.3	36.6	55.4	36.1
chr12	Alkbh1	Intron5	N	88781365	88784591	15	49.5	30.2	42.1	23.4
chr15	Ugt3a1	Intron5	N	9240464	9241503	5	86.6	64.9	80.1	61.5
chr19	Pdlim1	Intron5	N	40324194	40326399	11	47.3	25.1	48.6	30.0
chr10	Ggt5	Intron5	N	75067546	75068000	2	48.7	7.7	70.9	53.1
chr11	Pigl	Intron5	N	62323354	62325639	11	81.5	46.4	67.4	49.6
chr1	Iqca	Intron5	N	91963500	91967086	18	67.4	44.1	61.6	43.9
chr3	BC021767	Intron5	N	94467742	94468897	6	72.3	52.0	71.8	54.5
chr1	Fhl2	Intron5	N	43210100	43220546	50	71.9	43.7	65.0	47.9
chr10	Pmel	Intron5	N	128152745	128152982	1	38.3	20.3	41.6	24.8
chr8	Nqo1	Intron5	N	109916913	109926965	50	80.3	59.7	74.1	57.3
chr9	Mlh1	Intron5	N	111135891	111138410	13	81.4	65.7	84.5	67.7
chr17	Rpl10a	Intron5	N	28467643	28467774	1	40.9	21.7	40.3	23.7
chr7	Relb	Intron5	N	20199235	20200907	8	67.0	41.0	69.8	53.2
chr11	Map3k14	Intron5	N	103086937	103088300	7	86.7	50.7	83.4	66.9
chr14	4930524C18Rik	Intron5	N	115256845	115260016	16	92.9	66.2	97.3	81.2
chr2	Rpl12	Intron5	N	32819104	32819260	2	20.8	5.8	25.4	9.7
chr16	Comt	Intron5	N	18424454	18426448	9	51.8	33.9	41.3	25.6
chr8	Fhod1	Intron5	N	107854478	107855028	3	86.8	66.0	83.1	67.6
chr10	Sarnp	Intron5	N	128283640	128285292	8	90.9	71.4	85.3	70.0
chr14	Dnah1	Intron50	N	32108105	32108397	1	49.0	33.3	49.5	30.6
chr14	Stab1	Intron53	N	31972506	31972682	1	65.2	40.0	84.0	46.0
chr18	Alpk2	Intron6	N	65453770	65453865	1	74.7	12.0	76.8	10.5
chr9	Nr2e3	Intron6	N	59796836	59796988	1	86.4	25.3	89.0	24.6
chr8	Plat	Intron6	N	23883283	23884088	4	75.9	53.2	64.5	5.2
chr7	Slc22a18	Intron6	N	150676830	150677650	4	75.9	2.9	64.6	8.8
chr11	Pnpo	Intron6	N	96803849	96805044	4	83.3	18.8	81.0	29.9
chr12	Entpd5	Intron6	N	85726160	85726746	3	83.6	36.8	87.2	37.2
chr16	Mina	Intron6	N	59486462	59487253	4	70.9	19.4	63.6	13.8
chr11	BC096441	Intron6	N	69498633	69500458	10	37.6	7.7	59.2	9.6
chr12	Prkar2b	Intron6	N	32657069	32660783	19	43.3	24.9	50.2	5.9
chr19	Kdm2a	Intron6	N	4326899	4328127	6	91.5	71.5	83.9	40.0
chr7	Ceacam1	Intron6	N	26259072	26259538	2	18.9	0.0	51.6	7.7
chr3	1700061I17Rik	Intron6	N	116770744	116771889	6	40.6	22.1	51.5	14.1
chr2	Ttc16	Intron6	N	32624504	32624617	1	55.6	8.7	52.8	17.0
chr2	Gm711	Intron6	N	26798722	26799396	3	80.0	40.4	73.9	38.7
chr10	Mtrf1l	Intron6	N	4532501	4533134	3	60.8	15.0	71.6	36.7
chr5	Hsd17b11	Intron6	N	104447301	104450548	16	69.2	44.8	64.9	30.4
chr15	Krt83	Intron6	N	101265108	101265911	4	93.1	45.4	84.3	50.2

chr6	Pparg	Intron6	N	115413304	115422878	47	75.6	46.3	74.3	41.3
chr4	Ugcg	Intron6	N	59230087	59231366	6	86.4	66.5	91.5	60.8
chr7	Ppp5c	Intron6	N	17592596	17593074	2	75.0	53.5	70.7	40.0
chr6	Clcn1	Intron6	N	42243018	42244076	5	42.5	12.4	59.3	30.0
chr2	Ivd	Intron6	N	118699041	118700400	7	62.8	32.9	70.7	41.9
chr8	Tcf25	Intron6	N	125912548	125913564	5	94.1	40.5	87.0	58.5
chr13	Slc17a2	Intron6	N	23909477	23910886	7	95.0	63.6	92.0	64.0
chr7	Arrdc4	Intron6	N	75889816	75890030	1	59.2	37.1	65.1	37.3
chr4	Tnfrsf14	Intron6	N	154300834	154301247	2	19.7	1.3	29.0	1.8
chr7	Vasp	Intron6	N	19844817	19845929	6	76.5	50.7	47.1	20.8
chr3	Ccdc144b	Intron6	N	35924364	35924830	2	70.3	45.8	80.7	54.5
chr7	Il18bp	Intron6	N	109166179	109166623	3	64.2	35.5	54.5	29.6
chr5	Rassf6	Intron6	N	91038019	91038740	4	37.6	12.8	32.2	7.4
chr15	Krt5	Intron6	N	101541063	101541169	1	77.8	52.9	64.4	40.4
chr13	Tgfbi	Intron6	N	56727542	56729274	9	89.4	63.9	78.3	54.4
chr6	Ogg1	Intron6	N	113283675	113283835	2	47.6	22.4	51.5	28.4
chr15	Gsdmc4	Intron6	N	63726515	63726711	1	82.8	36.7	73.0	50.8
chr16	Tnk2	Intron6	N	32670317	32670877	3	55.8	40.4	53.0	30.9
chr19	Pyroxd2	Intron6	N	42808646	42809912	6	87.2	58.9	64.1	42.1
chr18	Tmx3	Intron6	N	90690430	90692921	10	62.2	39.2	66.4	44.4
chr4	Sdr16c5	Intron6	N	3943586	3946595	16	64.8	49.1	55.4	33.6
chr8	Slc25a42	Intron6	N	72715866	72718022	11	90.1	60.8	62.1	40.4
chr8	Slc18a1	Intron6	N	71567903	71575178	36	76.7	61.2	57.9	36.4
chr6	Apobec1	Intron6	N	122546796	122550075	17	80.4	55.7	83.9	62.7
chr3	Casq2	Intron6	N	101930603	101931863	6	100.0	61.1	23.4	2.4
chr13	Ccnb1	Intron6	N	101553603	101555411	9	72.3	45.7	65.9	45.5
chr1	Slco6c1	Intron6	N	98972560	98977897	23	63.5	46.9	76.6	56.2
chr7	Deaf1	Intron6	N	148507075	148507824	4	82.9	54.0	80.3	60.0
chr2	Mavs	Intron6	N	131071406	131072099	3	87.8	58.7	91.7	71.6
chr9	Fbxo22	Intron6	N	55068995	55071046	12	81.0	64.9	87.7	67.6
chr10	Tube1	Intron6	N	38862183	38864180	10	82.9	67.6	84.0	64.0
chr19	Ms4a4d	Intron6	N	11630784	11632366	8	93.1	66.7	75.0	55.0
chr2	Cpne1	Intron6	N	155903426	155903510	1	83.7	57.6	68.8	49.1
chr7	Pop4	Intron6	N	39053974	39056165	10	76.9	49.8	76.4	57.3
chr19	Fads3	Intron6	N	10129157	10129596	2	67.9	52.3	52.7	34.1
chr9	Twf2	Intron6	N	106115395	106116431	5	90.6	67.4	79.0	60.7
chr13	Serpinb6e	Intron6	N	33933225	33934575	7	76.9	50.0	94.4	76.2
chr17	Mlst8	Intron6	N	24614971	24615055	1	44.9	20.0	39.2	21.2
chr17	Gpr110	Intron6	N	43433789	43435953	11	87.4	67.6	90.1	72.5
chr15	Map3k12	Intron6	N	102332614	102332745	1	57.1	15.0	40.0	22.5
chr11	Mpdu1	Intron6	N	69472379	69475970	17	80.0	41.8	80.9	63.5
chr2	Tlk1	Intron6	N	70560146	70562058	10	74.3	45.4	82.0	64.7
chr9	Gorasp1	Intron6	N	119839425	119841738	12	88.4	44.8	67.7	50.5
chr1	Casq1	Intron6	N	174145274	174145607	2	93.2	58.9	81.9	64.8
chr2	Optn	Intron6	N	4954271	4955199	5	77.0	58.1	78.2	61.4
chr2	Tmco5b	Intron6	N	113129961	113130932	5	80.0	45.8	56.3	40.0
chr8	Mmp2	Intron6	N	95360042	95360804	4	93.4	59.7	92.3	76.2
chr7	Trim12c	Intron6	N	111495259	111496441	6	76.6	59.3	79.6	63.7
chr15	Pde1b	Intron6	N	103352593	103354738	11	66.0	41.4	65.7	49.8

chr2	Frmd5	Intron6	N	121383145	121383559	2	59.7	43.9	64.2	48.5
chr11	Git1	Intron6	N	77314244	77314573	2	88.2	64.0	60.8	45.3
chr9	Rora	Intron6	N	69219144	69220895	9	76.7	59.7	76.5	61.2
chr1	2-Mar	Intron6	N	186657908	186665132	35	69.7	53.4	68.2	52.9
chr9	Dapk2	Intron6	N	66098193	66098881	3	58.6	30.4	75.3	60.1
chr6	Thumpd3	Intron6	N	113013165	113015537	12	75.7	45.5	68.2	53.2
chr17	Col11a2	Intron62	N	34201789	34201950	1	46.7	26.6	48.8	23.5
chr7	Ryr1	Intron65	N	29861284	29861819	3	63.3	38.1	71.4	28.0
chr5	Fras1	Intron66	N	97197511	97198524	5	72.2	49.8	53.6	3.2
chr5	Htt	Intron66	N	35250243	35251280	5	61.0	37.5	58.8	33.8
chr11	Tsr1	Intron7	N	74715669	74717321	7	63.5	25.3	65.4	16.8
chr10	Tmprss9	Intron7	N	80350301	80351030	3	23.8	0.0	49.1	1.5
chr2	Entpd8	Intron7	N	24939609	24939780	1	78.8	31.0	67.1	20.3
chr11	Polg2	Intron7	N	106638876	106640272	6	33.1	2.1	80.1	35.4
chr5	Gm17660	Intron7	N	104503793	104503879	1	66.8	27.2	80.4	35.9
chr17	Fkbp5	Intron7	N	28565412	28566165	4	70.3	39.3	65.2	22.5
chr17	Ubash3a	Intron7	N	31365176	31368356	15	54.8	33.9	75.0	34.1
chr1	Idh1	Intron7	N	65217825	65221804	20	81.0	46.9	90.5	51.4
chr9	Nr2e3	Intron7	N	59797116	59797594	2	63.3	23.1	61.0	24.8
chr7	Uevld	Intron7	N	54200720	54203282	13	91.7	56.9	88.0	52.6
chr4	Gale	Intron7	N	135523172	135523266	1	84.2	67.5	89.8	56.1
chr11	Arhgap27	Intron7	N	103195678	103196514	3	40.9	25.6	45.5	12.4
chr11	Mettl16	Intron7	N	74617403	74618754	7	94.3	70.4	79.9	47.0
chr12	Snx6	Intron7	N	55864713	55866570	9	78.3	47.9	80.8	48.8
chr6	Phb2	Intron7	N	124665678	124665971	1	52.5	35.7	56.1	25.0
chr9	Kdelc2	Intron7	N	53206130	53207791	8	82.2	61.9	81.8	51.0
chr1	Usf1	Intron7	N	173347461	173347603	1	82.0	56.9	76.5	45.9
chr4	Akr1a1	Intron7	N	116314583	116318108	18	88.7	65.4	82.2	52.4
chr17	Gm9992	Intron7	N	7581467	7583552	10	62.9	42.4	73.0	43.2
chr10	Dazap1	Intron7	N	79743757	79745768	10	51.5	9.8	41.7	12.1
chr3	Cth	Intron7	N	157573905	157576563	13	60.3	27.3	63.5	35.3
chr4	Trit1	Intron7	N	122726508	122726719	1	82.7	52.7	80.9	53.7
chr15	Twf1	Intron7	N	94416968	94418658	7	92.5	70.8	94.2	67.3
chr5	Csn2	Intron7	N	88126306	88127052	3	91.7	50.0	95.0	70.0
chr19	Gif	Intron7	N	11833630	11834789	6	75.3	52.0	74.3	50.0
chr19	Cyp2c69	Intron7	N	39955733	39955935	1	83.3	58.8	70.4	46.2
chr6	Fkbp4	Intron7	N	128384891	128385731	4	81.9	47.6	82.9	59.1
chr17	Btnl2	Intron7	N	34502407	34503328	5	69.2	47.6	69.6	45.9
chr11	4933422H20Rik	Intron7	N	115308135	115308977	5	41.8	12.5	26.8	5.3
chr7	Arfip2	Intron7	N	112787980	112788758	3	30.2	0.0	37.9	16.7
chr10	D10Bwg1379e	Intron7	N	18320417	18323212	14	34.4	13.2	66.0	45.4
chr13	Mterfd1	Intron7	N	67031160	67033870	12	90.1	61.9	91.2	70.8
chr5	Srpk2	Intron7	N	23031564	23032607	5	92.2	37.5	77.5	57.1
chr5	Sult1d1	Intron7	N	87995218	87997821	13	60.6	25.0	41.0	20.7
chr2	Sephs1	Intron7	N	4820610	4826517	29	71.3	48.5	83.7	63.8
chr2	Mtg2	Intron7	N	179818166	179818768	3	95.7	79.2	82.0	62.7
chr6	Clcn1	Intron7	N	42244156	42248797	23	64.4	49.2	61.5	42.3
chr2	Ehf	Intron7	N	103119921	103123677	19	29.0	9.8	27.8	9.1
chr17	Mlst8	Intron7	N	24615108	24615264	2	39.1	15.1	32.1	13.7

chr5	Hnrnpd	Intron7	N	100396407	100405552	45	74.5	46.8	43.6	25.4
chr10	Adamtsl5	Intron7	N	79806472	79807577	6	43.1	22.8	39.8	22.0
chr2	Abtb2	Intron7	N	103545890	103547733	9	74.9	57.8	56.0	38.2
chr13	Cdk7	Intron7	N	101487638	101489256	7	79.8	29.3	53.7	37.6
chr5	Klhl5	Intron7	N	65550248	65552280	10	79.6	63.6	80.4	64.5
chr15	Slc22a22	Intron7	N	57086811	57088029	6	96.8	81.4	87.7	71.8
chr5	Lrrc43	Intron7	N	123951340	123953184	9	79.1	50.7	71.8	56.0
chr9	4931406C07Rik	Intron7	N	15102386	15105810	16	82.0	56.3	50.7	34.9
chr17	Crisp2	Intron7	N	40927614	40928825	6	91.3	68.7	96.2	80.4
chr3	Tdo2	Intron7	N	81772194	81773411	5	89.7	69.0	95.0	80.0
chr12	Syne2	Intron70	N	77125145	77126543	7	86.8	66.5	86.1	71.1
chr7	Ryr1	Intron76	N	29875247	29875938	3	82.0	50.0	85.4	58.1
chr5	Tmem175	Intron8	N	109072223	109073186	5	95.7	31.8	96.6	9.1
chr1	Tnnt2	Intron8	N	137744350	137744557	1	75.3	43.8	78.2	25.0
chr1	Hibch	Intron8	N	52958178	52959690	8	62.8	16.7	75.8	23.7
chr2	Anapc2	Intron8	N	25134834	25135646	4	72.6	40.6	92.9	42.6
chr13	Pik3r1	Intron8	N	102459453	102460435	5	77.2	41.2	82.9	33.9
chr9	Fbxl2	Intron8	N	113898555	113899735	6	82.6	64.1	72.1	24.6
chr2	Slc1a2	Intron8	N	102601367	102607089	29	69.8	30.9	76.6	32.6
chr1	Als2	Intron8	N	59230158	59231767	8	97.4	54.0	90.5	47.1
chr17	BC051142	Intron8	N	34580404	34580982	3	81.0	53.1	93.5	51.4
chr1	Tnni1	Intron8	N	137706313	137707189	5	50.6	9.5	48.7	7.8
chr11	Wipi1	Intron8	N	109445482	109446408	5	67.7	10.0	76.4	37.7
chr11	Luc7l3	Intron8	N	94165301	94167499	11	44.4	12.1	57.8	21.2
chr16	Nfkbiz	Intron8	N	55818648	55818860	1	90.8	68.9	86.6	51.9
chr14	Gmpr2	Intron8	N	56296086	56296707	3	71.4	37.9	68.7	35.4
chr7	Stim1	Intron8	N	109574655	109575213	3	41.7	10.2	43.1	10.1
chr5	Bst1	Intron8	N	44231730	44233235	8	60.6	35.0	48.9	15.8
chr5	Nsun5	Intron8	N	135851356	135851424	2	76.7	40.4	80.3	47.4
chr2	Stam	Intron8	N	14052746	14055979	16	32.3	1.3	36.7	4.6
chr4	Trit1	Intron8	N	122726798	122729308	13	40.1	14.1	44.7	13.3
chr2	Meis2	Intron8	N	115885301	115886416	4	37.5	20.8	42.3	12.0
chr1	Lamb3	Intron8	N	195152268	195154087	9	76.0	46.7	57.9	30.1
chr5	Kdm2b	Intron8	N	123332234	123332410	1	91.2	44.4	73.8	46.2
chr11	Tmem106a	Intron8	N	101451303	101451646	2	45.3	13.7	39.7	13.1
chr11	Nf1	Intron8	N	79223054	79224997	9	93.8	73.8	100.0	74.3
chr8	Tecr	Intron8	N	86097043	86097118	1	73.1	42.3	74.6	50.0
chr12	Actr10	Intron8	N	72055642	72057259	8	84.0	66.9	82.4	58.4
chr4	Wdr31	Intron8	N	62124511	62124960	2	91.7	34.3	54.7	30.8
chr9	Trcg1	Intron8	N	57094563	57095691	6	32.1	5.1	52.2	28.4
chr7	Snrnp70	Intron8	N	52647696	52650028	12	40.3	19.9	45.5	21.7
chr17	Btnl5	Intron8	N	34632961	34634007	5	69.9	18.2	59.7	36.3
chr9	Amica1	Intron8	N	44909229	44911117	9	30.5	5.1	37.2	14.5
chr17	Chaf1a	Intron8	N	56201735	56201973	1	85.7	61.5	83.3	60.7
chr13	Tgfbi	Intron8	N	56731192	56731904	4	73.2	28.1	54.8	32.7
chr3	Psmd4	Intron8	N	94839847	94840526	3	100.0	83.3	94.5	72.4
chr11	Mpp2	Intron8	N	101924730	101925607	4	55.1	23.3	74.9	53.3
chr13	Hexb	Intron8	N	97953762	97955522	9	63.7	48.1	53.3	31.7
chr3	Ccdc144b	Intron8	N	35931868	35934197	12	79.2	60.9	81.3	60.2

chr6	Phb2	Intron8	N	124666053	124666621	3	77.6	32.0	60.5	40.1
chr10	Tmem194	Intron8	N	127132599	127133283	5	76.4	58.2	76.6	56.5
chr16	Jam2	Intron8	N	84819021	84821504	12	77.0	59.1	77.0	57.1
chr9	Pcsk7	Intron8	N	45723310	45724070	4	91.8	72.2	88.3	69.4
chr6	Mcm2	Intron8	N	88838260	88839007	4	79.2	56.4	67.7	49.1
chr7	Otoa	Intron8	N	128246249	128249385	16	84.0	36.5	85.8	67.6
chr10	Sgk1	Intron8	N	21716161	21716364	1	93.6	51.1	87.5	69.4
chr17	Decr2	Intron8	N	26225943	26226908	4	74.9	51.9	65.3	47.7
chr2	Lrp2	Intron8	N	69271150	69273059	10	90.2	51.7	64.3	47.3
chr4	Tle1	Intron8	N	71799723	71800722	5	84.9	48.8	85.1	68.1
chr1	Smarcal1	Intron8	N	72645504	72647942	12	74.6	56.5	78.6	62.2
chr17	Rasgrp3	Intron8	N	75900097	75902453	10	70.8	51.0	63.0	46.7
chr9	Neo1	Intron8	N	58747041	58749889	13	68.8	51.1	68.6	52.9
chr7	Oat	Intron8	N	139758974	139761588	12	91.8	70.6	94.1	78.4
chr11	Obscn	Intron83	N	58894466	58895296	4	96.2	76.7	89.4	67.2
chr7	Ryr1	Intron86	N	29885504	29886752	6	100.0	27.9	81.2	60.3
chr10	Lrp1	Intron87	N	127047537	127049712	11	59.9	39.2	65.1	35.9
chr4	Eif3i	Intron9	N	129276287	129277652	5	85.0	41.8	95.7	43.5
chr3	Fstl5	Intron9	N	76393031	76397364	20	82.9	41.6	91.9	44.9
chr10	Cpsf6	Intron9	N	116805045	116813894	34	44.0	8.4	49.7	3.6
chr1	Tnnt2	Intron9	N	137744675	137745387	4	82.3	66.3	83.6	38.3
chr2	Fnbp4	Intron9	N	90603703	90605986	11	68.7	51.4	80.8	35.7
chr3	Fhdc1	Intron9	N	84268297	84269343	5	90.5	46.7	90.0	46.7
chr12	Evl	Intron9	N	109919765	109921404	8	88.8	51.8	92.5	53.3
chr15	Vdr	Intron9	N	97718920	97738665	97	73.2	37.5	71.5	34.1
chr5	Kdm2b	Intron9	N	123332498	123333327	4	79.8	13.0	78.2	42.4
chr14	Ncoa4	Intron9	N	32990560	32991008	2	63.0	42.1	62.5	27.1
chr4	Bai2	Intron9	N	129685674	129686454	4	41.5	13.5	45.8	11.9
chr1	Dars	Intron9	N	130275455	130283693	41	79.2	46.1	78.1	46.9
chr9	Amica1	Intron9	N	44911179	44912272	5	28.6	2.7	34.6	3.7
chr2	Serinc3	Intron9	N	163465064	163470733	26	68.6	36.6	73.5	43.0
chr17	Mocs1	Intron9	N	49591657	49592119	2	27.6	8.5	44.9	15.4
chr10	Mknk2	Intron9	N	80132721	80134303	8	91.7	61.3	90.3	61.2
chr11	Ccdc104	Intron9	N	29145151	29147014	8	46.0	27.9	31.5	2.9
chr5	Acads	Intron9	N	115567738	115569233	6	89.3	57.7	88.9	61.0
chr16	Eif4a2	Intron9	N	23111666	23111889	2	48.6	31.6	54.5	27.7
chr4	Trit1	Intron9	N	122729419	122729726	2	60.0	30.8	46.7	20.0
chr19	Entpd1	Intron9	N	40811375	40813312	10	36.9	15.0	42.8	17.6
chr7	Ech1	Intron9	N	29616887	29617022	1	33.9	3.0	33.2	8.1
chr7	D430042O09Rik	Intron9	N	132956464	132957106	3	84.5	61.9	88.6	64.3
chr7	Oat	Intron9	N	139761824	139767977	27	75.7	25.2	58.3	34.6
chr11	Gfpt2	Intron9	N	49632757	49636713	20	87.5	72.1	74.2	51.2
chr4	Sepn1	Intron9	N	134101606	134103865	11	58.7	29.0	61.0	39.4
chr5	Sec31a	Intron9	N	100808021	100810178	11	62.5	34.2	48.6	27.3
chr9	Impdh2	Intron9	N	108466023	108466965	5	70.9	55.4	80.3	59.0
chr18	Nars	Intron9	N	64668970	64670106	6	85.6	66.7	89.1	68.1
chr18	Oacyl	Intron9	N	65897659	65899729	10	82.0	54.4	78.3	58.6
chr1	Usf1	Intron9	N	173347996	173348162	1	82.4	44.9	82.0	62.5
chr8	Vps4a	Intron9	N	109567032	109568354	7	85.0	47.0	67.3	48.2

chr8	Smarca5	Intron9	N	83234576	83235555	5	70.8	36.9	92.9	74.0
chr2	Man1b1	Intron9	N	25203747	25204090	2	78.7	57.2	81.1	62.8
chr1	Cflar	Intron9	N	58809691	58810627	5	100.0	69.2	83.9	65.9
chr8	Rfx1	Intron9	N	86611972	86614009	10	77.2	58.2	68.8	50.9
chr6	Slco1a5	Intron9	N	142202988	142207497	21	89.5	68.8	82.0	64.2
chr7	Kat8	Intron9	N	135068461	135068622	1	64.1	48.4	56.5	39.0
chr2	Tmc2	Intron9	N	130058147	130060513	12	74.9	58.0	79.1	61.9
chr6	Rasgef1a	Intron9	N	118037305	118038126	4	93.0	76.7	84.6	67.5
chr11	Ncor1	Intron9	N	62144427	62147164	14	91.8	75.3	94.4	77.8
chr9	Ubl7	Intron9	N	57770426	57770961	3	96.2	70.0	94.6	79.3
chr5	Sel1l3	LastExon	N	53498322	53499413	6	80.0	22.9	78.6	24.1
chr5	Khk	LastExon	N	31233335	31233619	2	61.9	27.3	67.9	17.5
chr15	Krt8	LastExon	N	101827142	101827564	2	39.4	19.4	65.7	15.5
chr17	Fsd1	LastExon	N	56136065	56136304	3	88.9	45.8	89.7	40.6
chr2	Il1a	LastExon	N	129125346	129126635	7	64.1	8.1	54.1	5.6
chr1	Cnot11	LastExon	N	39601914	39603722	9	92.0	46.0	70.2	22.4
chr17	Wdr90	LastExon	N	25981679	25982170	3	55.1	17.2	66.3	18.7
chr11	Sstr2	LastExon	N	113486903	113486950	1	72.6	50.5	74.3	29.4
chr8	Cul4a	LastExon	N	13146532	13147939	7	65.5	28.7	65.9	21.3
chr12	Gpx2	LastExon	N	77893322	77893987	4	83.7	47.8	85.6	43.9
chr10	Socs2	LastExon	N	94874124	94875744	8	74.0	29.0	63.8	22.7
chr4	AI464131	LastExon	N	41442634	41446723	10	88.3	46.5	89.0	48.6
chr2	Trib3	LastExon	N	152163161	152164423	6	51.7	15.4	58.9	18.6
chr2	Fap	LastExon	N	62338993	62339345	2	74.6	31.6	69.5	32.1
chr7	Siglec5	LastExon	N	50613953	50614840	4	80.3	34.9	82.2	45.1
chr14	Fam160b2	LastExon	N	70983102	70984990	9	86.4	36.5	84.5	48.3
chr2	Ada	LastExon	N	163552307	163552784	3	41.7	2.6	38.5	2.7
chr15	Mei1	LastExon	N	81957044	81957244	2	67.8	52.3	79.7	44.2
chr7	Slx1b	LastExon	N	133832441	133835169	14	83.5	63.2	86.8	52.1
chr9	Cxcr5	LastExon	N	44319870	44322384	9	81.7	57.5	81.8	48.9
chr8	Slc18a1	LastExon	N	71561607	71562804	6	35.0	14.1	57.4	24.8
chr3	Tifa	LastExon	N	127499487	127501307	10	71.5	29.9	76.8	44.2
chr1	Tnni1	LastExon	N	137707190	137707566	2	43.5	21.2	59.9	27.3
chr17	Ntn3	LastExon	N	24340791	24343892	5	53.7	34.0	60.9	28.6
chr11	Rasd1	LastExon	N	59776685	59777868	2	68.9	36.1	59.9	27.6
chr3	Impa1	LastExon	N	10313540	10315301	9	82.9	0.7	76.6	44.8
chr9	Oxsr1	LastExon	N	119147551	119150334	14	67.1	34.2	77.9	46.5
chr14	Mmrn2	LastExon	N	35216099	35217472	7	82.2	56.5	80.4	49.1
chr12	Arl4a	LastExon	N	40759878	40763420	16	34.5	6.9	31.0	0.3
chr4	Tspan1	LastExon	N	115834486	115835640	7	77.4	30.2	76.2	45.6
chr17	Tbl3	LastExon	N	24837598	24837730	1	76.8	55.8	72.3	42.5
chr7	Pwwp2b	LastExon	N	146452371	146453152	4	41.1	22.1	57.5	27.9
chr11	Hoxb4	LastExon	N	96181344	96182952	4	44.7	28.0	68.1	38.9
chr16	Klhl6	LastExon	N	19946586	19947386	4	80.6	38.6	75.8	46.7
chr18	Atg12	LastExon	N	46892071	46894163	11	49.7	34.1	58.8	29.9
chr17	Wdr46	LastExon	N	34086223	34086640	3	39.8	12.9	40.0	11.3
chr2	Nsfl1c	LastExon	N	151336643	151337040	2	87.0	57.1	70.6	42.0
chr9	Rasgrf1	LastExon	N	89921509	89921817	2	61.4	46.0	74.2	45.6
chr17	Gpank1	LastExon	N	35261165	35261760	3	36.6	2.2	47.6	19.7

chr17	Fam195a	LastExon	N	26000643	26000984	2	70.4	45.9	67.8	40.0
chr4	Hcrtr1	LastExon	N	129807461	129808248	4	47.9	15.0	45.2	17.9
chr14	Prkcd	LastExon	N	31408540	31409211	4	91.9	64.0	89.7	62.4
chr11	Eif1	LastExon	N	100182631	100183410	5	42.5	4.1	36.4	9.2
chr3	Trim46	LastExon	N	89038099	89039214	6	65.9	48.3	69.1	42.0
chr2	Entpd6	LastExon	N	150596365	150597410	6	19.3	1.8	34.0	6.9
chr1	Pm20d1	LastExon	N	133712564	133714692	11	78.6	53.8	83.6	56.6
chr8	Pbx4	LastExon	N	72395894	72396191	2	91.5	70.5	82.1	55.4
chr11	Sap30bp	LastExon	N	115825525	115826848	7	67.3	30.0	61.8	35.2
chr7	Ppfibp2	LastExon	N	114891061	114892097	6	81.3	55.2	75.4	49.0
chr14	Lrrc18	LastExon	N	33827011	33828478	7	58.2	41.2	62.6	36.3
chr15	Myc	LastExon	N	61820901	61821916	3	83.4	48.4	84.1	58.3
chr1	Lefty2	LastExon	N	182827581	182829233	8	68.9	51.3	71.2	45.4
chr6	Usp5	LastExon	N	124765037	124765677	4	34.1	1.9	41.2	16.2
chr12	Cfl2	LastExon	N	55959806	55962224	13	70.5	54.9	82.7	57.8
chr9	Rplp1	LastExon	N	61761090	61761328	2	46.6	29.3	45.7	21.0
chr11	4933402P03Rik	LastExon	N	69630068	69631443	8	69.8	45.4	79.2	54.5
chr13	Dmgdh	LastExon	N	94522175	94522778	3	23.1	2.4	60.7	36.1
chr17	Ehmt2	LastExon	N	35050585	35050992	3	60.7	9.5	70.2	45.7
chr4	Slc44a1	LastExon	N	53634467	53635350	5	89.8	33.5	65.5	41.1
chr6	Gadd45a	LastExon	N	66985090	66985766	4	35.1	6.0	33.9	9.6
chr4	Mpl	LastExon	N	118115022	118116285	6	68.9	39.5	73.4	49.0
chr4	Aldh1b1	LastExon	N	45815327	45817480	12	90.5	53.0	88.8	64.8
chr9	Msl2	LastExon	N	101002901	101007129	21	85.0	56.0	83.3	59.5
chr7	Dnajb13	LastExon	N	107651529	107651819	2	82.5	59.1	91.2	67.6
chr1	Ankar	LastExon	N	72689554	72689885	3	81.8	63.8	75.9	52.6
chr11	Car4	LastExon	N	84779113	84779556	2	76.9	48.6	82.4	59.1
chr6	Ogg1	LastExon	N	113283836	113284180	3	47.6	22.4	51.5	28.4
chr7	Alg8	LastExon	N	104540038	104540668	3	83.3	51.3	76.1	53.1
chr19	9930021J03Rik	LastExon	N	29788892	29794096	26	89.1	53.0	86.6	63.8
chr1	Gls	LastExon	N	52220294	52223158	15	100.0	70.6	91.0	69.2
chr11	Mien1	LastExon	N	98299022	98299459	4	50.7	29.1	41.6	19.8
chr10	Asf1a	LastExon	N	53327471	53329021	6	77.8	15.6	83.9	62.1
chr2	Pmpca	LastExon	N	26250939	26252641	9	96.7	49.1	78.4	56.7
chr5	Htt	LastExon	N	35251281	35255170	19	90.7	48.4	71.5	49.9
chr1	D630023F18Rik	LastExon	N	65153887	65155379	8	97.0	80.0	93.8	72.2
chr19	Tmem223	LastExon	N	8846538	8846965	3	86.0	27.5	77.5	56.2
chr5	Ttyh3	LastExon	N	141096532	141099485	15	80.2	57.4	80.6	59.5
chr5	3110082I17Rik	LastExon	N	139835693	139836684	5	53.3	34.8	59.3	38.9
chr9	AI593442	LastExon	N	52481147	52486622	20	26.7	8.2	42.5	22.3
chr6	Clec9a	LastExon	N	129372244	129374782	13	69.8	22.9	54.4	34.3
chr9	Kank2	LastExon	N	21571217	21573448	12	72.6	56.4	69.7	49.9
chr11	Ramp3	LastExon	N	6576486	6577478	5	50.1	4.9	66.9	47.0
chr17	Noxo1	LastExon	N	24837066	24837474	3	20.0	1.8	20.6	0.9
chr10	Cdk2	LastExon	N	128134995	128136266	7	64.6	42.3	65.2	45.6
chr14	Rnase1	LastExon	N	51764677	51765594	5	60.1	41.2	73.7	54.3
chr8	Kxd1	LastExon	N	73037295	73038067	4	98.8	63.1	79.5	60.3
chr12	Tex21	LastExon	N	77299677	77299938	2	84.8	67.5	82.9	63.8
chr16	Muc13	LastExon	N	33818961	33820013	5	91.2	73.0	85.0	66.0

chr1	Ap1s3	LastExon	N	79603451	79605777	10	91.4	59.1	83.0	64.3
chr19	Npas4	LastExon	N	4984355	4985096	2	34.2	10.0	66.0	47.4
chr7	Kcne3	LastExon	N	107332648	107333379	5	88.3	58.7	87.3	68.9
chr15	Rpl37	LastExon	N	5068567	5069140	4	87.0	64.0	77.1	58.8
chr14	Mrpl52	LastExon	N	55048419	55048587	2	58.1	39.4	72.4	54.2
chr3	Muc1	LastExon	N	89036946	89037291	3	53.8	28.0	58.3	40.2
chr2	Tubb4b	LastExon	N	25077678	25078888	7	86.5	66.8	77.9	59.9
chr4	Gnb1	LastExon	N	154931580	154933378	10	73.6	55.5	76.2	58.1
chr19	Banf1	LastExon	N	5364640	5365158	3	90.2	70.1	90.0	72.1
chr3	Cd1d1	LastExon	N	86799758	86800523	5	54.0	37.3	56.0	38.1
chr7	Pycard	LastExon	N	135134887	135136479	8	65.2	37.2	39.7	21.8
chr11	Map3k14	LastExon	N	103081078	103082503	7	77.3	54.3	69.8	52.0
chr10	Madcam1	LastExon	N	79130982	79131281	3	64.6	33.3	86.5	69.1
chr4	Cyp2j9	LastExon	N	96235120	96235593	2	82.4	63.4	87.3	70.0
chr16	Hmgn1	LastExon	N	96343195	96344024	5	89.2	69.2	88.8	71.6
chr3	Dennd2d	LastExon	N	106303454	106305948	13	91.0	74.9	87.2	70.2
chr14	Adam28	LastExon	N	69223055	69224653	8	87.2	61.6	77.9	60.8
chr17	Cdsn	LastExon	N	35691606	35694125	13	68.2	44.5	71.2	54.3
chr4	Orc1	LastExon	N	108286886	108287436	3	76.2	53.3	66.4	49.6
chr10	Rdh1	LastExon	N	127202377	127205355	15	94.7	77.2	94.9	78.2
chr1	Kcne4	LastExon	N	78814186	78816600	13	55.5	31.6	67.3	50.6
chr11	Slc13a5	LastExon	N	72055496	72057138	9	80.6	65.3	75.6	58.9
chr15	Atf4	LastExon	N	80087064	80087971	5	23.0	2.7	24.1	7.6
chr2	Cstad	LastExon	N	30463680	30464465	4	68.1	42.3	48.6	32.0
chr4	Tex38	LastExon	N	115452439	115453208	4	31.1	10.5	25.7	9.2
chr19	Ifit2	LastExon	N	34647557	34651024	16	76.5	56.9	75.0	58.5
chr9	Tmem202	LastExon	N	59366492	59367081	3	79.2	51.5	73.0	56.5
chr7	Fam24a	LastExon	N	138480013	138480230	2	62.7	37.8	57.6	41.1
chr14	Mdp1	LastExon	N	56276716	56277911	6	39.7	23.6	37.5	21.2
chr4	Zswim5	LastExon	N	116659076	116661710	13	55.3	37.7	53.0	36.7
chr5	Igfbp7	LastExon	N	77778265	77778524	2	65.3	42.9	76.5	60.4
chr11	Tmem106a	LastExon	N	101451647	101453099	8	94.3	63.4	50.4	34.7
chr11	Hoxb5	LastExon	N	96166204	96167435	4	78.6	46.7	52.7	37.0
chr4	Mxra8	LastExon	N	155217414	155218211	5	87.7	48.4	83.5	67.9
chr5	Grxcr1	LastExon	N	68557331	68557637	2	79.6	59.5	71.8	56.4
chr10	Lrriq1	LastExon	N	102525832	102526143	2	63.8	45.6	48.0	32.6
chr16	Abhd10	LastExon	N	45729838	45730106	2	34.9	14.1	34.4	19.0
chr5	Slc15a4	LastExon	N	128076036	128077142	6	94.1	71.3	88.4	73.0
chr6	Ube2h	LastExon	N	30161289	30165083	19	75.8	44.5	74.9	59.7
chr16	Parn	LastExon	N	13538057	13538995	5	39.3	6.1	44.4	29.4

Table S3: Significantly enriched GO categories associated with mDMR genes

Gene Cat.	CGI Status	Methylation	GO Category	Term	Genes	Count	%	P-Value	Benjamini
Gene body	CGI	Gain	Biological process	embryonic organ development		20	3.4	8.60E-05	9.50E-03
Gene body	CGI	Gain	Biological process	intracellular signaling cascade		48	8.1	1.10E-04	1.00E-02
Gene body	CGI	Gain	Biological process	regulation of RNA metabolic process		69	11.7	1.10E-04	1.00E-02
Gene body	CGI	Gain	Biological process	regulation of transcription, DNA-dependent		69	11.7	7.10E-05	1.10E-02
Gene body	CGI	Gain	Biological process	cell morphogenesis involved in differentiation		18	3.1	1.60E-04	1.40E-02
Gene body	CGI	Gain	Biological process	embryonic morphogenesis		24	4.1	3.60E-04	2.20E-02
Gene body	CGI	Gain	Biological process	branching morphogenesis of a tube		11	1.9	3.80E-04	2.30E-02
Gene body	CGI	Gain	Biological process	ion homeostasis		21	3.6	3.90E-04	2.30E-02
Gene body	CGI	Gain	Biological process	glycosphingolipid biosynthetic process		4	0.7	4.60E-04	2.60E-02
Gene body	CGI	Gain	Biological process	anterior/posterior pattern formation		14	2.4	5.50E-04	2.90E-02
Gene body	CGI	Gain	Biological process	regionalization		17	2.9	5.50E-04	2.90E-02
Gene body	CGI	Gain	Biological process	pattern specification process		20	3.4	6.90E-04	3.30E-02
Gene body	CGI	Gain	Biological process	regulation of cell development		14	2.4	7.90E-04	3.60E-02
Gene body	CGI	Gain	Biological process	dorsal/ventral neural tube patterning		5	0.8	9.90E-04	4.20E-02
Gene body	CGI	Gain	Biological process	regulation of small GTPase mediated signal transduction		17	2.9	1.10E-03	4.40E-02
Gene body	CGI	Gain	Cellular component	plasma membrane		139	23.6	3.20E-10	1.00E-07
Gene body	CGI	Gain	Cellular component	synapse		27	4.6	2.70E-06	2.80E-04
Gene body	CGI	Gain	Cellular component	cell junction		32	5.4	2.30E-05	1.80E-03
Gene body	CGI	Gain	Cellular component	myosin complex		9	1.5	3.90E-04	1.50E-02
Gene body	CGI	Gain	Cellular component	cytoskeleton		54	9.2	3.60E-04	1.60E-02
Gene body	CGI	Gain	Cellular component	cell projection		32	5.4	8.30E-04	2.60E-02
Gene body	CGI	Gain	Cellular component	clathrin-coated vesicle		11	1.9	9.60E-04	2.70E-02
Gene body	CGI	Gain	Cellular component	Golgi cisterna		5	0.8	7.80E-04	2.70E-02
Gene body	CGI	Gain	Cellular component	organelle subcompartment		5	0.8	7.80E-04	2.70E-02
Gene body	CGI	Gain	Molecular function	small GTPase regulator activity		24	4.1	1.10E-07	5.60E-05
Gene body	CGI	Gain	Molecular function	transcription regulator activity		59	10	5.40E-05	5.70E-03
Gene body	CGI	Gain	Molecular function	calmodulin binding		13	2.2	1.00E-04	8.80E-03
Gene body	CGI	Gain	Molecular function	voltage-gated ion channel activity		16	2.7	1.90E-04	1.50E-02
Gene body	CGI	Gain	Molecular function	lipid binding		23	3.9	3.90E-04	2.00E-02
Gene body	CGI	Gain	Molecular function	sequence-specific DNA binding		31	5.3	6.50E-04	3.10E-02
Gene body	CGI	Gain	Molecular function	guanyl-nucleotide exchange factor activity		13	2.2	1.10E-03	4.50E-02
Gene body	CGI	Loss	Molecular function	cation channel activity		15	4.4	3.60E-05	1.30E-02
Gene body	CGI	Loss	Molecular function	metal ion transmembrane transporter activity		16	4.7	5.20E-05	9.40E-03
Gene body	CGI	Loss	Molecular function	channel activity		17	5	2.00E-04	2.40E-02
Gene body	CGI	Loss	Molecular function	passive transmembrane transporter activity		17	5	2.00E-04	2.40E-02
Gene body	CGI	Loss	Molecular function	ion channel activity		16	4.7	4.00E-04	3.50E-02
Gene body	non-CGI	Loss	Cellular component	extracellular matrix part		20	1.2	1.30E-04	1.60E-02
Gene body	non-CGI	Loss	Cellular component	organelle lumen		128	7.6	1.20E-04	1.90E-02
Gene body	non-CGI	Loss	Cellular component	intracellular non-membrane-bounded organelle		198	11.8	2.20E-04	2.10E-02
Gene body	non-CGI	Loss	Cellular component	membrane-enclosed lumen		132	7.8	1.00E-04	2.40E-02
Gene body	non-CGI	Loss	Molecular function	purine nucleoside binding		187	11.1	3.90E-08	1.40E-05
Gene body	non-CGI	Loss	Molecular function	ATP binding		173	10.3	2.40E-07	5.10E-05
Gene body	non-CGI	Loss	Molecular function	purine nucleotide binding		204	12.1	1.40E-05	2.50E-03
Gene body	non-CGI	Loss	Molecular function	GTPase regulator activity		54	3.2	2.70E-05	4.10E-03
Gene body	non-CGI	Loss	Molecular function	cytoskeletal protein binding		59	3.5	4.70E-05	4.40E-03
Gene body	non-CGI	Loss	Molecular function	actin binding		45	2.7	5.00E-05	4.40E-03
Gene body	non-CGI	Loss	Molecular function	nucleoside-triphosphatase regulator activity		54	3.2	4.30E-05	4.50E-03
Promoter	non-CGI	Loss	Biological process	oxidation reduction		37	7.3	3.20E-06	5.20E-03
Promoter	non-CGI	Loss	Biological process	negative regulation of myeloid cell differentiation		6	1.2	1.00E-04	8.00E-02
Gene body	non-CGI	Gain	Biological process	vesicle-mediated transport		43	4.9	7.90E-06	2.00E-02
Gene body	non-CGI	Gain	Cellular component	cell junction		43	4.9	5.80E-06	2.10E-03
Gene body	non-CGI	Gain	Cellular component	plasma membrane		163	18.4	1.20E-04	8.50E-03
Gene body	non-CGI	Gain	Cellular component	anchoring junction		17	1.9	7.50E-05	9.10E-03
Gene body	non-CGI	Gain	Cellular component	internal side of plasma membrane		28	3.2	1.20E-04	1.10E-02
Gene body	non-CGI	Gain	Cellular component	cell projection		46	5.2	7.40E-05	1.30E-02
Gene body	non-CGI	Gain	Cellular component	cytoskeleton		72	8.1	6.00E-04	2.40E-02
Gene body	non-CGI	Gain	Cellular component	Golgi apparatus		47	5.3	1.70E-03	4.90E-02
Gene body	non-CGI	Gain	Molecular function	purine nucleotide binding		122	13.8	8.20E-07	6.20E-04
Gene body	non-CGI	Gain	Molecular function	ATP binding		96	10.8	8.10E-06	8.70E-04
Gene body	non-CGI	Gain	Molecular function	protein domain specific binding		23	2.6	2.30E-05	1.90E-03
Gene body	non-CGI	Gain	Molecular function	cytoskeletal protein binding		35	4	1.90E-04	1.40E-02
Gene body	non-CGI	Gain	Molecular function	protein kinase activity		44	5	3.00E-04	1.90E-02
Gene body	non-CGI	Gain	Molecular function	Rab guanyl-nucleotide exchange factor activity		4	0.5	3.00E-04	2.00E-02
Gene body	non-CGI	Gain	Molecular function	SH3 domain binding		12	1.4	4.90E-04	2.80E-02
Gene body	non-CGI	Gain	Molecular function	transcription activator activity		24	2.7	7.00E-04	3.70E-02
Gene body	non-CGI	Gain	Molecular function	motor activity		16	1.8	7.80E-04	3.80E-02

Table S4: Age-adjusted regression analysis of correlation between DNA methylation and gene expression

	Gene	Adjusted for age		FDR
		Correlation	*P* value	
Non-CGI	Rnf43	-0.45	0.09	0.13
	Zbtb22	-0.68	0.01	0.04
	Fam109a	-0.38	0.18	0.20
	Adamtsl5	-0.65	0.01	0.03
	Mif	0.37	0.20	0.20
CGI	B4galnt1	0.52	0.01	0.03
	Net37	0.50	0.01	0.03
	Lpar5	0.53	0.01	0.03
	Fkrp	0.42	0.03	0.10
	Phospho1	0.33	0.06	0.16

Table S5: Composition of diets

		GF vs. CNV studies			FMT studies	
		CNV-1<br>Harlan 2020	GF-1<br>Harlan 8656	GF-2<br>LabDiet 5021	CNV-2<br>LabDiet 5V5R	GF-3<br>LabDiet 5V0F
**Macronutrients**						
Protein	%	19.1	24.5	21.5	19.8	19.0
Fat (acid hydrolysis)	%	6.5	4.4	9.0	7.6	7.6
Fiber (Crude)	%	2.7	3.4	3.5	2.4	2.4
Ash	%	5.1	6.5	5.5	5.0	5.0
Metabolizable Energy	kcal/g	3.1	3.0	3.3	3.4	3.4
**Vitamins**						
Vitamin A	IU/g	15	37	30	18	30
Vitamin D3	IU/g	1.5	3.0	3.3	2.3	2.3
Vitamin E	IU/kg	110	134	59	100	140
Vitamin B1	mg/kg	17	121	86	120	120
Vitamin B2	mg/kg	15	17	8	14	27
Niacin	mg/kg	75	128	89	120	120
Vitamin B6	mg/kg	18	21	15	16	26
Vitamin B12	mg/kg	0.08	0.13	0.05	0.08	0.08
Biotin	mg/kg	0.4	0.9	0.3	0.4	0.9
Folic Acid	mg/kg	4	8	3	3.6	8.6
Choline	mg/kg	1200	2530	2200	2100	2100
**Minerals**						
Calcium	%	0.9	1.3	0.8	1.0	1.0
Phosphorus	%	0.7	1.0	0.8	0.6	0.6
Sodium	%	0.1	0.3	0.3	0.2	0.2
Potassium	%	0.4	0.9	0.8	0.5	0.5
Chloride	%	0.4	0.4	0.4	0.8	0.8
Magnesium	%	0.2	0.2	0.2	0.2	0.2
Zinc	mg/kg	60	87	140	110	110
Manganese	mg/kg	80	109	130	140	140
Copper	mg/kg	15	23	17	15	15
Iodine	mg/kg	6	2	2	1.5	1.5
Iron	mg/kg	200	347	220	240	240
Selenium	mg/kg	0.23	0.35	0.35	0.35	0.33
**Amino Acids**						
Threonine	%	0.6	1.0	0.8	0.6	0.6
Leucine	%	2.3	2.0	1.6	2.5	2.4
Isoleucine	%	0.7	1.3	0.9	0.8	0.8
Valine	%	0.9	1.3	1.0	0.9	0.8
Methionine	%	0.5	0.4	0.7	0.6	0.6
Cystine	%	0.3	0.4	0.3	0.4	0.4
Histidine	%	0.4	0.6	0.5	0.4	0.4
Arginine	%	0.8	1.9	1.2	0.7	0.7

Table S6. Primers and PCR conditions for genotyping assays

Gene	Primer Set	Sequence	Genotype	PCR Product (bp)	PCR Annealing (°C)
Lgr5-EGFP	8060 (common)	CTG CTC TCT GCT CCC AGT CT	WT	298	55
	8061 (WT reverse)	ATA CCC CAT CCC TTT TGA GC	Mut	174	
	oIMR9402 (mutant reverse)	GAA CTT CAG GGT CAG CTT GC			
Dnmt1$^{f/f}$	mDnmt1-1	GGG CCA GTT GTG TGA CTT GG	WT	334	55
	mDnmt1-2	CTT GGG CCT GGA TCT TGG GGA	F/F	368	
Dnmt 3a$^{f/f}$	Dnmt3a_WT Forward	TGAGTGGTGAGGCCCGGGCAT	WT	200	55
	Dnmt3a_WT Reverse	ATT CGA CCT GGT GAT TGG AG	F/F	400	
	Dnmt3a_MT Forward	CTG TGG CAT CTC AGG GTG ATG AGC A	WT	no band	55
	Dnmt3a_MT Reverse	AAG CCC AGG CCC TCT AGG CAA GAT	F/F	200	
Villin-Cre	oIMR1878(transgene)	GTG TGG GAC AGA GAA CAA ACC	Villin-Cre	1100	55
	oIMR1879(transgene)	ACA TCT TCA GGT TCT GCG GG	WT	200	
	oIMR8744(positive cntrl F)	CAA ATG TTG CTT GTC TGG TG			
	oIMR8745(positive cntrl R)	GTC AGT CGA GTG CAC AGT TT			

Table S7. Summary of WGBS results for genome-wide DNA methylation analysis

Samples	Number of Reads	Coverage Depth	Mapping Efficiency (%)	Bisulfite Conversion Efficiency (%)
P0 ISCs	1,143,947,860	21.0	66.6	98.4
P0 differentiated epithelial cells	1,131,900,850	20.8	72.0	98.4
P21 ISCs	1,161,799,014	21.3	66.5	98.3
P21 differentiated epithelial cells	1,104,562,844	20.3	69.5	98.4

Table S8. Bisulfite-pyrosequencing primers and sequenced regions for quantitative DNA methylation analysis

	Gene	Forward Primer	Reverse Primer (5' Biotinylated)	Sequencing Primer	Sequenced Region
Global	Line1	TGATAGTTTTTGGAATAGGTAGAAGT	CCCAAATAATACAAACTCTCACTTAA	TTGAGGTAGTATTTTGTGTG	GGTYGGGGAT AGTYGGTT
	IAP	AAGGAAAGGGGGAGATGTTGG	CRCCTAAAACRTATCACTCCCTAA	ATTTTATGTGTTTTGTTTTT	TTYGTGAYGT TAATTYGGTY GATGG
mDMR genes with methylation loss	Adamtsl5	GGAATGGGGTTTTAAAGTATATGTAG	AACTACCTATCTCCCTTCAATCTA	AGTAGTTTATATAATTGGGTG	T TYGGGGTAG TAYGTTTTTA
	Fam109a	TGGTTTGGAATTAGTTAAGTTAGGA	ACTCTCCCTACTACAAACATAAAAT	GTGTTTAGTTTTGTAGGT	GAGTTYGGGT AGAAGTAGGG
	Wnt11	GATTTTTGGGTTGGGTTGTTGGTAGAA	ATATCAAATATCCCTTTCATCTCCAAATCA	GTTGGTAGAAGGAAGTT	TYGGGATAGT TGTGTAGTGG GGGTAGGGAT TYGTTAGGGT
	Apc2	TTTTTGGAGGGGTTTGAGGAG	CCCCAAAACCTCTACTAC	TTGGTTTTGGTGAGG	TYGAGTTGGG TAGGGYGTGG YGAGTATTYG GGTTT
				ATTTTTTTTTAGGTGTTTATTTTA	GTTTYGTAGY GGGGGYGTAG
	Mif	GTAGAGGTTTTGTTTTAAGAAGGGATTTT	ATAAAACCACACCCTTTCCAACTAA	GTATAGAGATTAGTAAGTTGTTT	YGGGGGYGTA GAGTTTAGGT
	Rnf43	TGGGATGAAGGTAGAGGAGGTATA	AAACACCATCAACCCTTACC	GGAGTAGAAAGTTGGTT	YGGGTAGTTT GTATTAYGTG GGTAAGGGTT
	Zbtb22	GAAGTTTATGTGGAGGGATAGTTTTATG	CTCCACCCCCACTAAAATC	GGGTGTGGGTGGGGT	YGGATTTGGA TYGGGTTTTG GATTTGGATT
				TATAGTGGGTGGGGG	TAGTGGYGAT GAGGTAAATT YGGTTAYGTT TTYGTTTTAT AGGYGTGTTT
	Ipmk	GGGGAGGTTTTTTTTTAAAGGATT	CTTCTCCACCCCTTATCTTACTCCAAC	GGTTTGGAGTGTAATAATAATTTT	TATTTGTTYG GYGTTTYGTT TAAYGGGATG TYGGTGGGTA
				GGTGGGTAAGAGTTTAT	YGAAGGYGTA TTYGAGGTAT
mDMR genes with methylation gain	AI464131	GTTGGGAGGAAGTTGTGT	CAACCCAAATAAAAAAACACTAAATCATT	GAGTAAGAGATGTTTTTGGGAGA	TGTTGYGGYG TATTGGTATG GYGGYGTAGA
				ATTAGTTTAGATGGTTAGTAAGAAT	YGTAGTTATT TGTTAYGAGY GAYGTTTATT TATTTGAYGT
				GTATTAGGGGGTATTTTTGAG	YGTTATTGGT TATTTTTAYG YGTYGTTGTT
	B4galnt1	GGGGAGAGGGAAAAAGATGTTAGG	ACTCTCCTCCCCTACCAAAT	ATTTTTTTAGGGTTGGTT	YGTAGGTYGG AATTTGGYGG T
				GGTGTTTTAAGTAATTATTAAAT	AYGTGTTGTG GGTGGAYGAY GATTTTGTTT TTAYGGYGYG TA
				GGTTGGAGAAGTTTGTG	GAYGTGTTYG AGAAGAYGTT
	Lpar5	TGGTTTTTTTTAGGTTTTTTGTGTTAG	ACCTACCCTTCCACAATTC	GTGTGGGTTTTTATTTTGT	TGTTYGTTGT GTTYGTYGTT YGYGTGTATA GTTYGTTTTA
	Phospho1	GAAGTAGGAGTTTTGTTTGGTTATGAA	CTTCCCCCTACTCCCAAAA	TTGGTTATGAATTGTAATAAA	TYGTTTATGT TTGGYGATAG GGGGATGGTT TYGTAGATAG
				GTTGTATGTATTTATTGTAGTAGT	T TTYGYGATA GGTGGTAYGT AGGTTTTYGG GTAGTTGTTG
	Fkrp	AGTTTTTTTTTATGTGTTATGGTTATAATA	CCACTACCACCAACTAAACTAAATCCTACT	GTTATAATATTAGTTTAGAAATTTT	YGGGTTYGGG GTTTTYGTYG GATTTTTGTT
				GGATTTTTGTTATTGGTTTT	YGAGTTATYG TTTTGATTYG GGAATTTGAG
	Amn	TGAGAGTGGGGTTTTAAGTTTGT	CACCCCAAAAATCACAACATTATCCT	GAGGTGAGTGAGTTT	TGATYGGGTA TAGYGYGAGG GTYGGTTTYG GTT
				TTATTTTAGTGTTTTTATTTAGATG	TTGTYGTGGA TTTGYGYGTT
	Mab21l2	GTTATTGAGAGGTTGGTTAGGTAGTT	AATAACCTATACCCCACATCCC	AGGTAGTTTTAGGTGG	YGGTTTYGTA GYGTTTTTAG TATYGAGAGG
				GAGAGGTATTTGTTTTTATAGT	YGTTTATTAG TAAGYGGTTT TTYGTTTTAT YGAATTGTAG
				GGAGAGTAAGTTGAATTTT	TYGGTTTTGA TTTTYGTTAT TYGATTGGGG TYGGGTTAGG

Table S9. Real-time Taqman assays for gene expression analysis

Gene	Assay ID	RefSeq	Exon Boundary
Rnf43	Mm01307031_m1	NM_172448.3	9-10
Zbtb22	Mm00457884_s1	NM_020625.3	-
Fam109a	Mm01199886_m1	NM_175474.3	3-4
Adamtsl5	Mm01149530_g1	NM_001285435.1	-
Mif	Mm01611157_gH	NM_010798.2	2-3
AI464131	Mm01222105_m1	NM_001085515.2	1-2
B4galnt1	Mm00484649_g1	NM_001244618.1	1-2
Lpar5	Mm01190819_m1	NM_001163268.1	1-2
Phospho1	Mm00462190_m1	NM_153104.3	1-2
Fkrp	Mm00557870_m1	NM_173430.2	2-3
Dnmt1	Mm01151063_m1	NM_001199431.1	5-6
Dnmt3a	Mm00432881_m1	NM_007872.4	17-18
Dnmt3b	Mm01240113_m1	AF068627.2	19-20
β-Actin	mm00607939_s1	NM_007393.3	6-6
